# Proteomic analysis reveals that COP9 signalosome complex subunit 7A (CSN7A) is essential for the phase transition of migratory locust

**DOI:** 10.1038/srep12542

**Published:** 2015-07-27

**Authors:** Xi-Wen Tong, Bing Chen, Li-Hua Huang, Qi-Li Feng, Le Kang

**Affiliations:** 1 Guangdong Provincial Key Lab of Biotechnology for Plant Development, School of Life Sciences, South China Normal University, Guangzhou 510631, China; 2 State Key Laboratory of Integrated Management of Pest Insects and Rodents, Institute of Zoology, Chinese Academy of Sciences, Beijing 100101 China

## Abstract

The migratory locust displays a reversible, density-dependent transition between the two phases of gregaria and solitaria. This phenomenon is a typical kind of behavior plasticity. Here, we report that *COP9 signalosome complex subunit 7A* (*CSN7A*) is involved in the regulation of locust phase transition. Firstly, 90 proteins were identified to express differentially between the two phases by quantitative proteomic analysis. Gregaria revealed higher levels in proteins related to structure formation, melanism and energy metabolism, whereas solitaria had more abundant proteins related to digestion, absorption and chemical sensing. Subsequently, ten proteins including CSN7A were found to reveal differential mRNA expression profiles between the two phases. The *CSN7A* had higher mRNA level in the gregaria as compared with the solitaria, and the mRNA amount in the gregaria decreased remarkably during the 32 h-isolation. However, the mRNA level in the solitaria kept constant during the crowding rearing. Finally and importantly, RNA interference of *CSN7A* in gregaria resulted in obvious phase transition towards solitaria within 24 h. It suggests that *CSN7A* plays an essential role in the transition of gregaria towards solitaria in the migratory locust. To our knowledge, it’s the first time to report the role of CSN in behavior plasticity of animals.

The migratory locust (*Locusta migratoria*) is an important pest insect in Asia. When locust disaster breaks out, swarms of locusts gather at very high population densities, and then trigger the migration of whole population towards new areas with more food. The aggregation and migration of locusts definitely result in broader damage. In 1966, Uvarov brought forward the concepts of gregaria and solitaria to describe locust phases with high and low population densities, respectively^1^. The two phases distinguish each other in many aspects including morphology, behavior, coloration, reproduction, development, endocrine and immunity^2^. Their behavioral distinction is prominent: the gregaria is more active and easier to be attracted by other individuals, whereas the solitaria exhibits to be more isolated. These differences are usually used as key markers in behavioral assay to distinguish the two phases^3^,^4^,^5^,^6^. Locust phase can shift from one state to another in response to density changes. The phase transition is a continuous, cumulative, and easily reversible process^7^, and it can take place within a short period (from 4 h to 32 h) in both the migratory locust^3^ and the desert locust, *Schistocerca gregaria*^8^,^9^,^10^.

In recent years, many fruitful studies have been carried out to elucidate the intrinsic molecular mechanisms of phase transition in locusts from various aspects such as genomics, transcriptomics and metabolomics. A large scale of transcriptomic sequencing was carried out in the migratory locust using an expressed sequence tag (EST) technique in 2004^11^, and 532 differentially expressed unigenes were identified between the two phases. The transcriptome dynamics in the same species were further analyzed in 2010 based on a newly emerged next-generation sequencing technology^12^. A lot of genes related to neural pathway, such as dopamine receptor, adipokinetic hormone, neurotransmitter synthetase were found to be up-regulated in the gregarious locusts. Another transcriptomic analysis was performed in the desert locust^13^. The solitary locusts up-regulate genes related to antioxidant systems, detoxification and anabolic renewal, whereas gregarious locusts have a greater abundance of transcripts for genes involved in sensory processing and nervous system development and plasticity. After monitoring and comparing transcript profiles between the two phases at various developmental stages, Chen *et al*. found that a sharp rise in phase differences appeared during the 4^th^ instar and the high level difference was maintained in all the following stages. Therefore, the 4^th^ instar stage seems to be a turning point in the process of forming the phase differences in the migratory locust^12^. Some neuronal signaling and sensory activity related genes, such as *dopamine receptor*^5^, *chemosensory protein* (*CSP*) and *takeout*^3^ were proved to play roles during the phase transition. The successful assembly of the migratory locust genome is a milestone in the study of phase transition of locust^14^. The genome is 6.5 giga base pairs (Gb), the largest animal genome sequenced so far. Significant expansion of gene families associated with energy consumption and detoxification were found in the locust genome^14^. Besides, small RNA^15^ and metabolomics^4^ analysis also disclosed a lot of regulators contributing for the phase transition.

Proteomic researches have also been carried out, but few significant progresses have been made till now. In 1999, polypeptide maps were generated from hemolymph of the desert locust and twenty differential spots were identified between the two phases. However, detailed information about these peptides was not available^15^. Two proteins, a 6-kDa peptide and a serine protease inhibitor were identified to have different expression patterns between the two phases in the desert locust using a combined approach of high-performance liquid chromatography (HPLC) with matrix-assisted laser desorption/ionization time-of-flight mass spectrometry (MALDI-TOF MS)^16^. These proteomic studies moved very slowly because of the lack of locust genome information at that time.

The behavior plasticity makes locusts to be a good model in the epigenetic researches^17^,^18^. Two DNA methyltransferase genes were shown to be phase-specific in certain tissues of the desert locust^19^. Further analysis revealed that the methylome of the gregarious desert locust was characterized by CpG- and exon-specific methylation, and the overall methylation levels were substantially higher than other invertebrates^20^. These findings suggest that DNA methylation may be involved in the regulation of locust phase transition. Besides, a cAMP-dependent protein kinase (PKA) was reported to play a role in the transition from solitary to gregarious behavior in the desert locust^21^. Except for these reports, few studies have been further performed in recent years.

In general, large progresses have been made in exploring the mechanisms of phase transition in the migratory and desert locusts. A lot of differentially expressed genes and pathways have been identified based on DNA sequencing techniques. However, the researches in protein areas, such as protein identification and protein modification, have been largely lagged. One of the most key reasons is the lack of genome sequence information. Fortunately, the genome assembly of the migratory locust was just finished^14^, which provides much convenience for protein identification and will do great help for exploring the complex mechanism of phase transition in another viewpoint.

In the present study, we identified 90 differentially expressed proteins between the two phases in the migratory locust by a quantitative proteomic technique. Among them, CSN7A was found to play an essential role in the transition of gregaria towards solitaria.

## Results

### Proteins identified in the locust head

**Figure 1:**
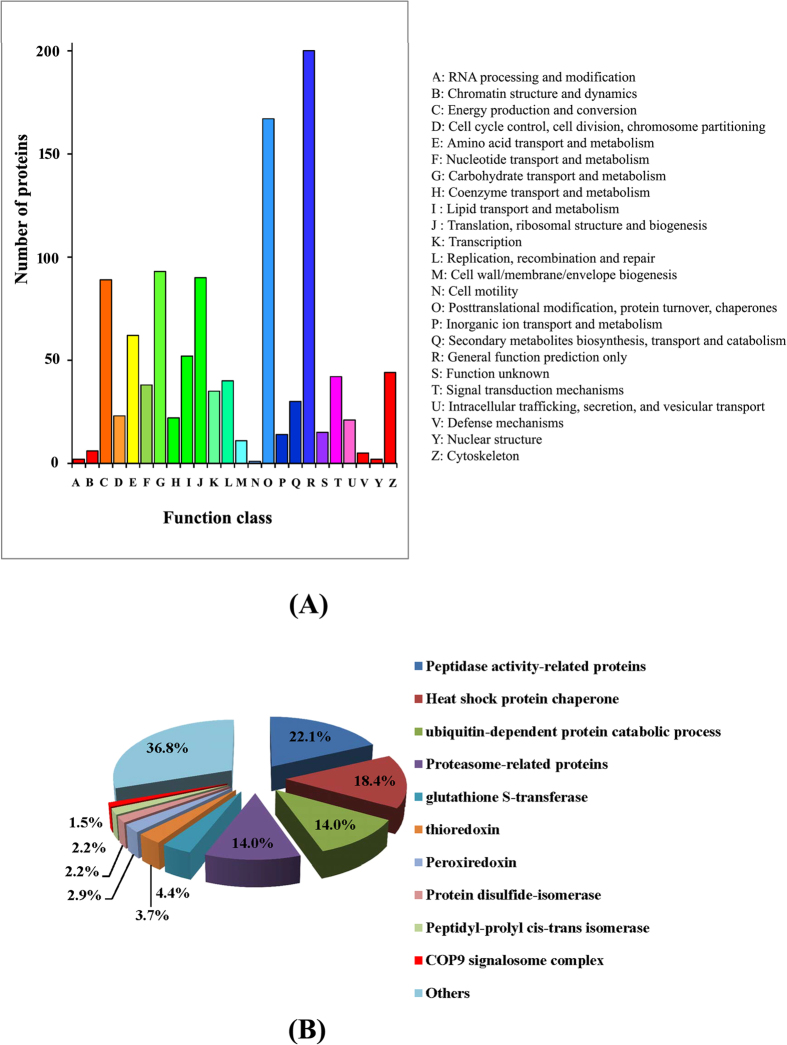
COG classification of identified proteins. Based on sequence homology, 1, 104 proteins were classified into 25 COG (Clusters of orthologous groups) categories (**A**). The “R” and “O” clusters represent the largest two groups. Proteins classified in the “O” cluster (Posttranslational modification, protein turnover, chaperones) were further assigned to different categories according to their molecular functions (**B**). Detailed information about these “O” cluster proteins can refer to Supplementary Table S1.

A total of 4, 895 peptides were identified by liquid chromatography coupled with tandem mass spectrometry (LC-MS/MS) from the locust head, and they were finally assembled into 1, 387 proteins. After COG classification, 1, 104 proteins were assigned to 25 COG categories, and the “R” cluster (General function predication) and “O” cluster (Posttranslational modification, protein turnover, chaperones) represent the largest two groups, and their amounts are 18% and 15% of the total identified proteins, respectively (Fig. 1). The “O” cluster proteins are mainly heat shock protein chaperones, ubiquitin-dependent proteins, proteasome-related proteins, peptidase activity-related proteins, glutathione S-transferase, protein disulfide-isomerase, and COP9 signalosome complex subunits (Supplementary Table S1). The top 70 most abundant proteins are listed in Table 1 and Supplementary Table S2. Most are the proteins related to structural construction, such as twitchin, spectrin alpha chain-like and microtubule-actin cross-linking factor 1. Many proteins, including pyruvate kinase (EC 2.7.1.40), malate dehydrogenase (EC 1.1.1.37), aconitate hydratase (EC 4.2.1.3), citrate synthase 2 (E.C. 2.3.3.1), succinyl-CoA ligase (EC 6.2.1.4), 2-oxoglutarate dehydrogenase E1 component (EC 1.2.4.2), and ATP-citrate synthase (EC 2.3.3.8), revealed high abundance. These proteins are involved in tricarboxylic acid cycle (TCA). It suggests that TCA is very active in the locust head. Besides, several heat shock proteins and hexamerins were also identified to be abundant in the locust head.

### Differentially expressed proteins between the two phases

Among the 1, 387 identified proteins, 90 proteins were shown to have different expression levels between the two phases. Sixty-four were up-regulated in the gregaria (as compared with the solitaria), and twenty-six were down-regulated (Table 2, Supplementary Table S3). Most of the up-regulated proteins are involved in the processes of structure formation (such as cuticle protein, beta-1 tubulin, profiling, and troponin), energy metabolism (electron transfer flavoprotein subunit alpha [ETFA], dehydrogenase/reductase SDR family member 11-like, 3-ketoacyl-CoA thiolase, V-type proton ATPase subunit B and isocitrate dehydrogenase NAD subunit beta), and environmental stress response (heat shock protein 60 [Hsp60] and heat shock protein 20.6 [Hsp20.6]). Besides, four hexamerin-like proteins are also abundant in the gregaria. The down-regulated proteins are mainly related to the processes of digestion and absorption (carboxypeptidase A-like, serine protease-like protein, and 1,4-alpha-glucan-branching enzyme-like) and chemical sensing (takeout-like). In addition, the differentially expressed proteins are also enriched in the class of “Regulation of gene expression” in both the gregaria and solitaria (Table 2). For example, wingless protein, 3′-phosphoadenosine 5′-phosphosulfate synthase (PAPSS), COP9 signalosome complex subunit 7A (CSN7A), and juvenile hormone binding protein (JHBP) were highly expressed in the gregaria, and splicing factor 3B subunit, ubiquitin-conjugating enzyme E2 variant 2-like isoform 1, proteasome subunit alpha type-4, and arginine/serine-rich-splicing factor RSP31 (RSP31) showed higher levels in the solitaria.

### Differential expression at mRNA levels

**Figure 2:**
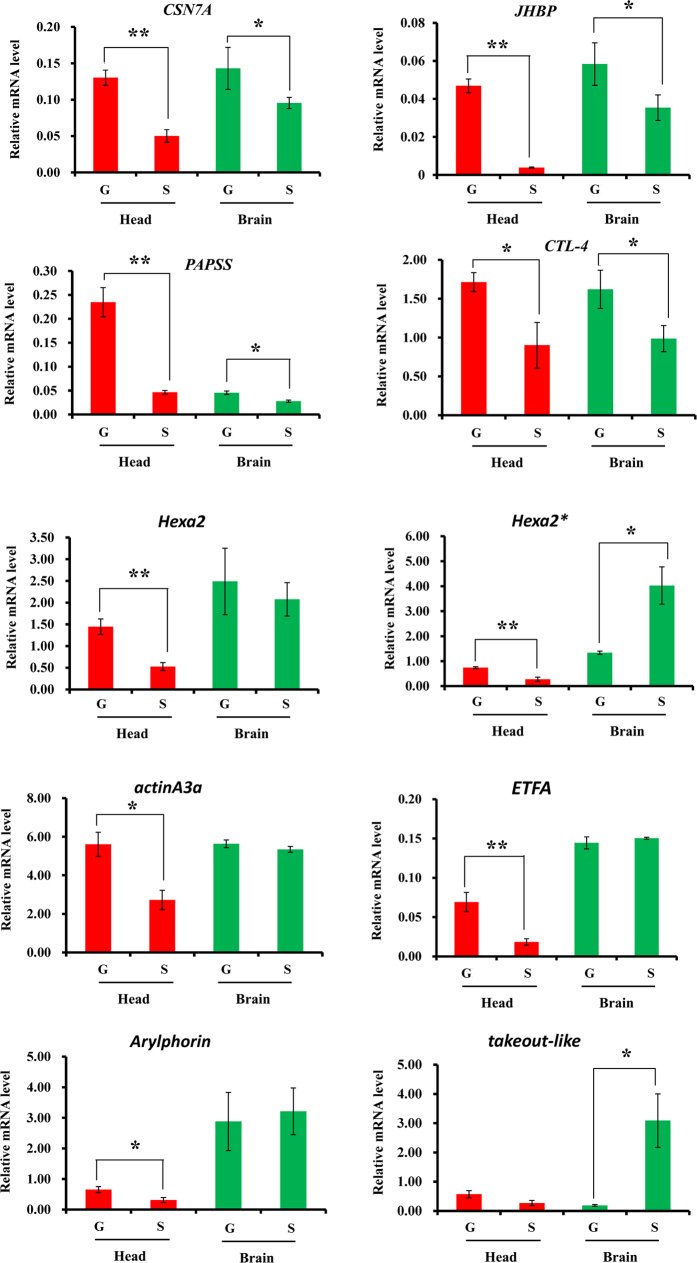
The mRNA expression profiles in the two-phase locusts. The mRNA expression profiles were examined by qRT-PCR in both the head and brain tissues. The mRNA levels were quantified by standard curves generated with serial (10×) dilutions of plasmid DNAs. The relative expression level of each target gene was normalized against a house-keeping gene (*RP49*). Differences between treatments were compared by Student’s *t*-test, and two levels (P < 0.05 or 0.01) were adopted to judge the significance of difference. Abbreviations: “G”, gregaria; “S”, solitaria. The abbreviation for gene names can refer to Table 3.

To validate the differential expression, fourteen representative proteins were selected according to their function categories in Table 2. Their mRNA expression profiles were examined in the whole head of the two-phase locusts. Nine protein genes, including *CSN7A*, *JHBP*, *PAPSS*, *choline transporter-like protein 4* (*CTL-4*), two *hexamerin-like protein 2* (*Hexa2* and Hexa2*), *cytoplasmic actin A3a* (*actinA3a*), *ETFA* and *Arylphorin* revealed higher mRNA level in gregaria (Fig. 2). It was in consistent with the protein profiles in Table 2. The brain tissues, the most important part of head, were also studied. Four genes, such as *CSN7A*, *JHBP*, *PAPSS*, *CTL-4* and *takeout-like*, showed similar expression patterns between the mRNA and protein levels. There were still four genes, including *V-ATPase subunit B* (*V-ATPase*), *ATPsyn-d*, *RSP31*, and *NADPH--cytochrome P450* (*P450*) revealed constant mRNA levels between the two phases (Supplementary Fig. S1).

### Time-dependent mRNA expression during phase transition

**Figure 3:**
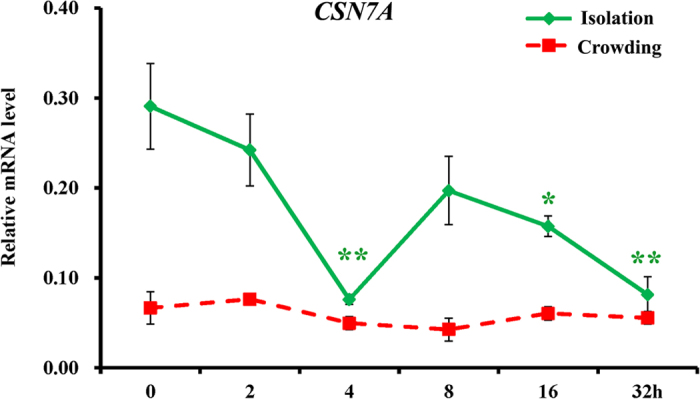
Time-dependent mRNA expression dynamics of *CSN7A* during phase transition. The mRNA expression dynamics were examined by qRT-PCR in the brain during phase transition. To make the gregarious behavior change towards solitaria, the 4^th^ instar gregarious nymphs were individually reared at the same condition as solitary ones. After 2, 4, 8, 16 and 32 h of isolation, the brains were dissected and sampled. Similar procedure was used to convert solitary individuals towards gregaria. The sampling and mRNA level detecting methods were as same as the isolation of gregaria. The untreated gregarious and solitary locusts were used as controls. Differences between each treatment and the corresponding control (untreated gregaria or solitaria) were compared by Student’s *t*-test, and two levels of significance (P < 0.05 or 0.01) were adopted to judge the significance of difference.

In order to further narrow target proteins that may play a role in the regulation of locust phase transition, *CSN7A* was chosen and time-dependent mRNA expression dynamics were examined in brain during the phase transition process. The *CSN7A* had higher mRNA level in the gregaria (Figs 2, 3), the level decreased significantly at 4, 16 and 32 h- isolation and was as low as that in the solitaria at 32 h (Fig. 3). However, the mRNA level did not change during the crowding of solitary locusts (Fig. 3).

### RNA interference (RNAi) and behavioral assay

**Figure 4:**
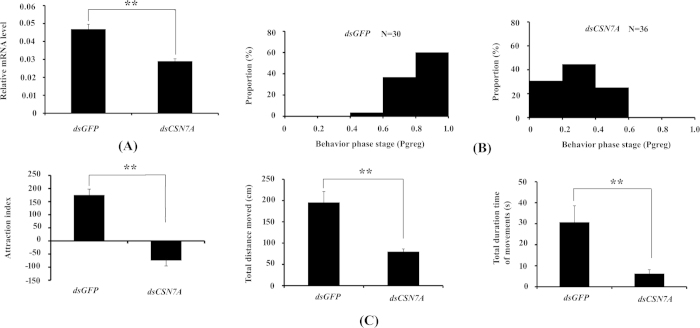
RNAi and behavioral assay in the gregarious locusts. RNAi of *CSN7A* in the gregaria (**A**). DsRNAs of *CSN7A* were injected directly into the brains of 4^th^ instar gregarious nymphs, and the individuals were used 24 h later. Half of the individuals were dissected and their brains were sampled for qRT-PCR, and the other half were used for behavioral assay. Possibility of gregaria (**B**). Three key behavioral parameters (attraction index, total distance moved, and total duration of movement) were compared between the dsCSN7A and dsGFP populations (**C**). Isolation of gregaria referred to the method in Fig. 3, and the behavioral assay was then performed in a rectangular arena monitored by EthoVision system. Eleven behavioral parameters (such as attraction index, total distance moved, total duration of movement, *etc*.) were collected to calculate the possibility of gregaria (P_greg_), which were used for criterion of phase type. The behavioral data were analyzed by the Mann-Whitney *U* test. The phase difference between two populations was highly significant (*P*_Mann-Whitney U test_ = 1.61 × 10^−12^). Differences between treatments were compared by Student’s *t*-test, and two levels of significance (P < 0.05 or 0.01) were adopted to judge the significance of difference. Individual numbers of gregaria and solitaria were marked directly on top of the figure.

To validate the function of *CSN7A* in locust phase transition, RNAi and behavioral assay were carried out. The mRNA level was suppressed by injection of *CSN7A* dsRNA in the gregaria (Fig. 4A), and the behavioral state shifted from gregaria (*dsGFP* population) to solitaria (*dsCSN7A* population) (Fig. 4B). The phase difference between two populations was highly significant (*P*_Mann-Whitney U test_ = 1.61 × 10^−12^). For example, 60% and 0% individuals fall into the P_greg_ interval of 0.8–1.0 in the *dsGFP* and *dsCSN7A* population, respectively. In addition, significant difference existed in the three key behavioral parameters (attraction index, total distance moved, and total duration of movement) between the two populations (Fig. 4C). These results revealed that phase transition did happen by RNAi of *CSN7A* in the gregarious locust.

## Discussion

The “O” cluster proteins are extremely abundant in the locust head. This phenomenon was also found in the antennae of *Batocera horsfieldi* based on cDNA library analysis^22^. However, similar phenomenon did not exist in the whole insect bodies^23^,^24^,^25^,^26^. It seems that “O” cluster proteins are mainly abundant in the head as compared with the other parts of insects. It suggests that the proteins related to post-translational modification, protein turnover and chaperone folding are highly involved in the regulation of head function in insects. Locust phase polyphenism is a typical phenomenon of epigenetics^17^,^19^,^26^. The existence of high abundant “O” cluster proteins suggests that post-translational modification may play important roles in the locust phase transition.

The two locust phases differ in many aspects, especially in the body color and behavioral activity. The gregaria is darker and more active, while the solitaria is shallower and quieter. The proteomic analysis revealed that proteins related to structure formation, melanism and energy metabolism have significantly higher expression level in the gregaria. This is consistent with the facts that gregarious locusts have stronger muscles, darker color and more frequent activity. As compared with the gregaria, the solitaria owns more abundant proteins related to digestion, absorption and chemical sensing. It’s apparently that the former two characteristics provide the solitary locusts with higher abilities in digestion and absorption, and the latter one gives them stronger olfactory sensation. This makes them have an advantage over the gregarious locusts in feeding and mating, and then results in higher reproductive capacity^14^.

In the present study, hexamerins and JHBP are abundant in the head of gregarious locust. Similar results have been revealed by EST library analysis in the same species^11^. Both hexamerin and JHBP have been suggested to play a role as juvenile hormone (JH) transporters, and even as regulators of JH levels and action^27^,^28^,^29^,^30^. This explains the involvement of hexamerins in JH-dependent differentiation of caste phenotype in some social insects, including termite *Reticulitermes flavipes*^31^,^32^,^33^,^34^, honey bee^35^ and wasp *Polistes metricus*^36^. Besides caste-related polyphenism in social insects, JH was also reported to mediate plasticity of aggregation behavior in adult desert locusts^37^. Surgical removal of the corpora allata to terminate JH secretion increased aggregation index and behavioral activity of adult locust. This effect was caused by repressing the responsiveness of olfactory interneurons in the antennal lobe to aggregation pheromone. Thus, hexamerins and JHBP can be involved in the phase plasticity of locust by mediating JH action.

Heat shock proteins (Hsps) are a kind of stress-induced proteins that can be synthesized rapidly in response to various environmental stress signals. Hsps usually function as molecular chaperones and participate in numerous cellular functions such as folding, assembly, intracellular localization, secretion, regulation and degradation of proteins^38^,^39^. Gregarious locusts live at high population density. Population density can alter the expression of Hsps. For example, the mRNA levels of five *Hsp*s (*Hsp20.5*, *Hsp20.6*, *Hsp20.7* and *Hsp90*) are significantly higher in the gregarious locust head as compared with those in the solitaria. The mRNA levels were up-regulated by crowding of the solitary locusts (for 32 h), and down-regulated by isolation of the gregarious locusts^40^. In the present study, Hsp60 and Hsp20.6 were identified to have higher protein levels in the gregarious locust head. The over-expression of Hsps in gregaria seems to be a direct response to high-population gather of locust. It’s hard to distinguish whether Hsps play a role to control the phase transition.

In the desert locusts, two phase populations display different sensitivity to aggregation pheromone^10^,^41^,^42^. Chemosensory protein (CSP) and takeout are important proteins for olfactory sensing^43^,^44^,^45^,^46^,^47^. RNA interference combined with olfactory behavioral experiments confirmed that six *CSP* genes (*CSP-1* to *6*) and one *takeout* gene, LmigTO1, are responsible for the formation of gregarious and solitary behaviors, respectively^3^. In our study, another CSP (CSP-7) and three new takeout proteins (TO 4 to 6) were identified from the head of *Locust migratoria* (Supplementary Fig. S2), and the CSPs revealed higher protein level in the gregaria, while the TOs showed higher protein levels in the solitaria. These protein expression patterns are consistent with the early report at mRNA levels^3^, and further confirm that both CSP and takeout are involved in the phase plasticity of locust.

The CSN, an eight protein complex (CSN1-8)^48^ was originally discovered as an essential regulator in light-induced development in *Arabidopsis thaliana*^49^. In *Drosophila melanogaster*, it also plays an essential role for development. Disruption of one of the subunits caused lethality at the late larval or pupal stages^50^. This role of CSN is partly due to its regulation on Hedgehog signaling by mediating proteolysis of some transcription factors^51^. In the same species, CSN was also reported to be involved in circadian rhythms by controlling the degradation of two clock proteins^52^. Interestingly, our study showed that *CSN7A* played a role in the phase transition from gregaria to solitaria in the migratory locusts. RNAi of *CSN7A* triggered the phase shift from gregaria to solitaria within 24 h (Fig. 4). Isolation (gregaria to solitaria) and crowding (solitaria to gregaia) may have different regulation mechanism. The former takes place within 4 h in the migratory locusts, whereas the latter cannot finish until 32 h^3^. In the present study, the mRNA amount of *CSN7A* in gregaria decreased during the isolation, however, the mRNA level remained constant during the crowding of solitaria (Fig. 3). It suggests that *CSN7A* may be only involved in one direction transition from gregaria to solitaria rather than in its reverse process.

It is the first time to disclose the role of *CSN* in behavior plasticity of animals. CSN has been reported to be involved in neural development, and regulates dendritic morphogenesis in *Drosophila* brain through Cullin-mediated protein degradation^53^. More and more evidences revealed that CSN plays an important role in protein degradation through Cullin-ubiquitin-proteasome pathway^54^,^55^,^56^. Therefore, CSN might be involved in the phase transition of locust by mediating ubiquitin-dependent proteolysis. Further studies need to be carried out to explore the detailed mechanism of CSN in the regulation of phase transition.

In conclusion, a total of 1,387 proteins were identified in the locust head in the present study, and a large proportion of proteins are involved in post-translational modification, especially in protein folding, phosphorylation and ubiquitylation. Ninety proteins were identified to differentially express between two phases in the head of the migratory locust. Gregaria reveals higher expression in proteins related to structure formation, melanism and energy metabolism, whereas solitaria owns more abundant proteins related to digestion, absorption and chemical sensing. This is consistent with their differentiation in morphology and physiology. JHBP, hexamerin, Hsp, CSP and takeout are suggested to play a role in behavior formation according to their differential expression profiles between two phases. The most interestingly, RNAi of *CSN7A* in gregaria made the behavior shift towards solitaria within 24 h. It is the first time to disclose the role of CSN in behavior plasticity of animals. These results provide important information for further exploration of the complex mechanism of locust phase transition, as well as for the study of behavior plasticity of animals.

## Methods

### Animals

The gregarious and solitary populations of the migratory locust are long-term maintained in our laboratory as the early reported method^5^. Briefly, gregarious nymphs were cultured in large boxes (40 × 40 × 40 cm^3^) at a density of 500–1000 insects per container. Solitary nymphs were obtained from the gregarious colony and cultured alone in white metal boxes (10 × 10 × 25 cm^3^) supplied with charcoal-filtered compressed air. The gregarious and solitary colonies were maintained under a 14 h light/10 h dark cycle at 30 ± 2 °C and fed on fresh wheat seedlings and bran.

### Sample preparation and iTRAQ labeling

When the locusts developed into the second day of 4^th^ instar, the heads of 3 to 5 gregarious or solitary nymphs were collected and thoroughly homogenized in 500 μL cold PBS buffer including 1 mM PMSF, 2 mM EDTA and 10 mM DTT. The samples were centrifuged for 20 min at 25,000 × g, and the supernatant was collected. A total of 100 μg of protein per sample was reduced, alkylated, and then digested by adding 2 μg trypsin (1 μg/μL) at 37 °C overnight. The digested samples were lyophilized and re-suspended in 100 μL of 0.5 M TEAB (triethylammonium bicarbonate). The method of isobaric tags for relative and absolute quantitation (iTRAQ) was adopted for sample labelling according to the protocol of iTRAQ^®^ Reagents—4plex Applications Kit (AB Sciex Pte. Ltd., Foster City, USA). Each sample was labeled with an isobaric tag. The iTRAQ-labeled peptide mixtures were pre-separated by strong cation exchange (SCX) column. For SCX chromatography, the LC-20AB HPLC Pump system (Shimadzu Corporation, Chiyoda-ku, Tokyo, Japan) was used, the peptide sample was reconstituted with 4 mLbuffer A (25 mM NaH_2_PO_4_ in 25% ACN, pH2.7) and then loaded onto a 4.6 × 250 mm Ultremex SCX column containing 5-μm particles (Phenomenex, Torrance, CA, USA). The peptides was eluted at a flow rate of 1 mL/min with a gradient of buffer A for 10 min, 5–35% buffer B (25 mM NaH_2_PO4, 1M KCl in 25% ACN, pH2.7) for 11 min, 35–80% buffer B for 1 min. The system was then maintained in 80% buffer B for 3 min before equilibrating with buffer A for 10 min prior to the next injection. Elution was monitored by measuring absorbance at 214 nm, and fractions were collected every 1 min. The eluted peptides were pooled as 12 fractions, desalted by Strata X C18 column (Phenomenex, Torrance, CA, USA) and vacuum-dried. Each fraction was resuspended in certain volume of buffer A (2% ACN, 0.1% FA).

### LC-MS/MS Analysis

A total of 5 μg of the above solution was loaded on a LC-20AD nanoHPLC (Shimadzu Corporation, Chiyoda-ku, Tokyo, Japan) equipped with a 2 cm C18 trap column, and the peptides were then eluted onto a resolving 10 cm analytical C18 column. The MS data acquisition was performed with Triple TOF 5600 System (AB SCIEX, Concord, ON) fitted with a Nanospray III source (AB SCIEX, Concord, ON) and a pulled quartz tip as the emitter (New Objectives, Woburn, MA). Data was acquired using an ion spray voltage of 2.5 kV, curtain gas of 30 PSI, nebulizer gas of 15 PSI, and an interface heater temperature of 150 °C. The MS was operated with a resolving power of greater than or equal to 30,000 FWHM (full width at half maximum). The MS/MS data collection and processing was done on Analyst® software (version 1.6, AB SCIEX, Concord, ON) with the method of Information Dependent Acquisition (IDA) according to the manual.

### Database searching for protein identification

The resulting MS/MS spectra were searched against the locust protein database generated from the newly assembled genome^7^ with MASCOT software (Matrix Science, London, UK; version 2.3.02). The carbamidomethylation of cysteine was considered a fixed modification, and the conversion of N-terminal glutamine to pyroglutamic acid and methionine oxidation were considered variable modifications. The minimal peptide length was seven amino acids, and a single missed cleavage maximum was used. A peptide mass tolerance of 10 ppm was allowed for intact peptide masses and 0.05 Da for fragmented ions. A stringent 0.01 false discovery rate (FDR) threshold was used to filter the candidate peptide and protein. Two thresholds were set up to filter the candidate proteins whose abundances were significantly different from others: <0.05 for a two-tailed *P*-value test and >1.5 (or <1/1.5) for the fold-change. For gene ontology (GO, http://www.geneontology.org/) mapping, BLAST2GO software (version 2.5.0, http://www.blast2go.org) was employed to deal with the BLASTx results and then to perform the functional annotation by GO vocabularies, enzyme classification codes, KEGG metabolism pathways^57^. The default settings of BLAST2GO were used in every annotation step.

### Quantitative real-time PCR (qRT-PCR)

Total RNAs were extracted from the whole head and dissected brain tissues, respectively using an RNAeasy mini kit (QIAGEN, Hilden, Germany). Three heads or eight brains were used for each RNA isolation, and five biological repeats were performed during sampling. PCR reactions were performed in a 20 μL volume and the final concentration of primers was 250 nM. PCR amplification was conducted on a Roche Light Cycler^®^ 480 system (*Roche* Applied Science, Penzberg, Germany) using SYBR green master mix (Roche Diagnostics Ltd. Shanghai, China). The PCR was initiated with a 10-min incubation at 95 °C, followed by 45 cycles of 10 s at 95 °C, 20 s at 58 °C and 20 s at 72 °C. Five biological replicates were performed for each sample. The standard curves for target genes and reference genes (*ribosomal protein 49*, *RP49*) were generated with serial (10×) dilutions of plasmid DNAs. Efficiency of qRT-PCR and correlation coefficients were determined for the primers of each gene. The relative expression level of each target gene was normalized against *RP49*. The specificity of amplification was ensured by both melting curve analysis and sequencing of PCR product. The primers for qRT-PCR were listed in Table 3.

### RNAi

Double-strand RNA (dsRNA) of the target gene and a negative control gene (*green fluorescent protein, gfp*) were prepared using the T7 RiboMAX Express RNAi system (Promega, Madison, USA) according to the manufacturer’s instruction. The primers for dsRNA preparation were listed in Table 3. A total of 35 ng dsRNA was injected directly into eight brains of the 4^th^ instar nymphs using Nanoject II nanoliter injector (Warner Instruments, Hamden, CT, USA). Twenty four hours later, the effects of RNAi on mRNA level were detected by qRT-PCR and behavioral assay. Four biological repeats were performed for qRT-PCR. For behavioral assay, the same injection was carried out in gregaria, and 30 and 36 individuals were used for dsGFP and dsCSN7A, respectively.

### Phase Transition

To make gregarious behavior change towards solitaria, the 4^th^ instar gregarious nymphs were individually reared at the same condition as solitary ones. After 2, 4, 8, 16 and 32 h of isolation, the brains were dissected and immediately placed in RNAlater Solution (Ambion, Austin, USA) for qRT-PCR analysis. The gregarious nymphs maintained in normal situation (high population density) were used as controls. To avoid the influences of circadian rhythm and sexual difference, all samples were collected at the same time point of a day with a sex ratio of 1:1. Each treatment included five biological replicates. To make a reverse phase transition (solitaria towards gregaria), ten solitary nymphs were marked and moved into an optic perplex-made box (10 × 10 × 10 cm^3^), and 20 gregarious individuals were then added to maintain high population density. The sampling, mRNA level detecting and other methods were as same as the isolation of gregaria.

### Behavioral assay

The behavioral assay was performed in a rectangular arena (40 × 30 × 10 cm^3^). The wall of the arena is opaque plastic and the top is clear. One of the separated chambers (7.5 × 30 × 10 cm^3^) contained 20 4^th^ instar gregarious locusts as the stimulus group, and the other end of the chamber with the same dimensions was kept empty. Both ends of the chamber were illuminated equally to prevent the formation of mirror images. The floor of the open arena was covered with filter paper during the behavioral assay. The locust nymphs were gently transferred by a tunnel to the arena. Each individual was recorded for 6 min using EthoVision system (Noldus Inc. Wageningen, the Netherlands). Eleven behavioral parameters (such as attraction index, total distance moved, total duration of movement, *etc*.) were collected to calculate the possibility of gregaria (P_greg_), which was used for criterion of phase type. Detailed information can refer to the early reported methods^3^,^6^.

### Statistical analysis

Differences between mRNA levels were compared by Student’s *t*-test. The relative mRNA levels were presented as mean ± SEM (standard error of the mean). Behavioral data were analyzed by the Mann-Whitney *U* test. Two levels of significance (P < 0.05 or 0.01) were adopted to judge the significance of difference. All the statistics was analyzed using SPSS 15.0 (SPSS Inc., Chicago, USA).

## Additional Information

**How to cite this article**: Tong, X.-W. *et al*. Proteomic analysis reveals that COP9 signalosome complex subunit 7A (CSN7A) is essential for the phase transition of migratory locust. *Sci. Rep*. **5**, 12542; doi: 10.1038/srep12542 (2015).

## Supplementary Material

Supplementary Information

## Figures and Tables

**Table 1:** Top 70 most abundant proteins identified in the locust head.

No.	ID number in thelocust genomedatabase (version 2.0)	Blast P			Peptide
		Protein name	Species	Accession No.	
1	LMI_GLEAN_10128031	twitchin	*Cerapachys biroi*	EZA52953	166
2	LMI_GLEAN_10163232	apolipophorin II	*Locusta migratoria*	Q9U943	103
3	LMI_GLEAN_10136968	myosin heavy chain, muscle-like	*Apis florea*	XP_003695415	77
4	LMI_GLEAN_10143205	spectrin alpha chain-like	*Apis mellifera*	XP_006558458	50
5	LMI_GLEAN_10182824	muscle M-line assembly protein unc-89	*Cerapachys biroi*	EZA59129	49
6	LMI_GLEAN_10088460	filamin-A isoform X3	*Tribolium castaneum*	XP_008199793	48
7	LMI_GLEAN_10187850	microtubule-actin cross-linking factor 1 isoform X6	*Nasonia vitripennis*	XP_008203191	45
8	LMI_GLEAN_10135516	alpha-actinin, sarcomeric isoform X2	*Tribolium castaneum*	XP_972324	36
9	LMI_GLEAN_10062054	paramyosin, long form	*Zootermopsis nevadensis*	KDR08790	33
10	LMI_GLEAN_10137418	spectrin beta chain	*Zootermopsis nevadensis*	KDR16227	31
11	LMI_GLEAN_10140538	titin	*Harpegnathos saltator*	EFN83273	28
12	LMI_GLEAN_10134774	elongation factor 2	*Schistocerca gregaria*	AEV89753	26
13	LMI_GLEAN_10128585	pyruvate kinase	Zootermopsis nevadensis	KDR19430	26
14	LMI_gi_37993866	heat shock protein 70	*Locusta migratoria*	AAP57537	25
15	LMI_GLEAN_10160912	glycogen phosphorylase-like	*Apis florea*	XP_003690485	25
16	LMI_GLEAN_10157178	staphylococcal nuclease domain-containing protein 1	*Tribolium castaneum*	XP_974879	23
17	LMI_GLEAN_10153094	coracle, partial	*Blattella germanica*	CCI09964	23
18	LMI_gi_93278396	heat shock protein 90	Locusta migratoria	AAS45246	21
19	LMI_gi_99867354	arginine kinase	*Locusta migratoria manilensis*	ABF68036	20
20	LMI_GLEAN_10097368	ATP-citrate synthase	*Zootermopsis nevadensis*	KDR07798	20
21	LMI_GLEAN_10043824	clathrin, partial	*Locusta migratoria*	AHC70342	19
22	LMI_GLEAN_10164084	heat shock 70 kDa protein cognate 5	*Zootermopsis nevadensis*	KDR08641	18
23	LMI_gi_241997152	ER protein gp78	*Locusta migratoria*	ACS75353	18
24	LMI_gi_256368118	hexamerin-like protein 2	*Locusta migratoria*	ACU78069	18
25	LMI_GLEAN_10109513	tropomyosin-1	Nasonia vitripennis	XP_001599003	17
26	LMI_GLEAN_10065878	60 kDa heat shock protein, mitochondrial	Zootermopsis nevadensis	KDR14060	17
27	LMI_GLEAN_10001937	myosin heavy chain, non-muscle-like isoform 2	*Bombus terrestris*	XP_003394420	17
28	LMI_gi_225194719	pro-phenoloxidase 2	*Locusta migratoria*	ACN81829	17
29	LMI_GLEAN_10141796	vinculin-like isoform 1	*Bombus impatiens*	XP_003493644	17
30	LMI_GLEAN_10143564	hexamerin-like protein 2	Locusta migratoria	ACU78069	17
31	LMI_GLEAN_10196247	beta-actin	*Diabolocatantops pinguis*	ACV32627	16
32	LMI_GLEAN_10097173	tropomyosin-1, isoforms 9A/A/B	*Camponotus floridanus*	EFN72212	16
33	LMI_GLEAN_10021933	tubulin beta-1 chain	*Tribolium castaneum*	XP_967267	16
34	LMI_GLEAN_10170791	fructose 1,6-bisphosphate aldolase	*Schistocerca gregaria*	AEV89754	16
35	LMI_GLEAN_10123052	malate dehydrogenase, mitochondrial	*Nasonia vitripennis*	XP_001600547	16
36	LMI_GLEAN_10097518	alpha tubulin	*Schistocerca gregaria*	AEV89775	16
37	LMI_GLEAN_10142607	alpha tubulin	*Schistocerca gregaria*	AEV89775	16
38	LMI_GLEAN_10096450	alpha tubulin	*Schistocerca gregaria*	AEV89775	16
39	LMI_GLEAN_10111205	aconitate hydratase, mitochondrial-like	*Megachile rotundata*	XP_003705474	16
40	LMI_GLEAN_10170835	nesprin-1	Zootermopsis nevadensis	KDR09330	16
41	LMI_GLEAN_10104518	pyruvate carboxylase, mitochondrial	*Zootermopsis nevadensis*	KDR22588	16
42	LMI_GLEAN_10168000	annexin-B9	*Zootermopsis nevadensis*	KDR08631	15
43	LMI_GLEAN_10143558	hexamerin-like protein 1	*Locusta migratoria*	ACU78068	15
44	LMI_GLEAN_10126189	citrate synthase 2, mitochondrial	*Zootermopsis nevadensis*	KDR22581	15
45	LMI_GLEAN_10189307	ubiquitin-like modifier-activating enzyme 1	*Zootermopsis nevadensis*	KDR20513	15
46	LMI_GLEAN_10113091	ATPase	*Homo sapiens*	AAA35578	15
47	LMI_GLEAN_10165425	glycogen debranching enzyme, partial	*Zootermopsis nevadensis*	KDR16306	15
48	LMI_GLEAN_10136778	neither inactivation nor afterpotential protein C	*Zootermopsis nevadensis]*	KDR20620	15
49	LMI_GLEAN_10056004	cytoplasmic A3a	*Helicoverpa armigera*	Q25010	14
50	LMI_GLEAN_10156223	transitional endoplasmic reticulum ATPase TER94	*Zootermopsis nevadensis*	KDR08983	14
51	LMI_GLEAN_10085205	transferrin	*Romalea microptera*	AAQ62963	14
52	LMI_GLEAN_10071372	14-3-3 protein zeta	*Zootermopsis nevadensis*	KDR15025	13
53	LMI_GLEAN_10081307	glutamate dehydrogenase, mitochondrial, partial	*Zootermopsis nevadensis*	KDR15400	13
54	LMI_GLEAN_10127881	tubulin alpha-3 chain, partial	*Anas platyrhynchos*	EOA98266	13
55	LMI_GLEAN_10183379	14-3-3 protein epsilon	*Schistocerca gregaria*	AEV8977	13
56	LMI_GLEAN_10181426	2-oxoglutarate dehydrogenase E1 component, mitochondrial	*Zootermopsis nevadensis*	KDR11185	13
57	LMI_GLEAN_10042900	mitochondrial F1-ATP synthase alpha subunit	*Locusta migratoria manilensis*	AGO59887	13
58	LMI_GLEAN_10122197	bifunctional purine biosynthesis protein PURH	*Zootermopsis nevadensis*	KDR19778	13
59	LMI_GLEAN_10154078	hexamerin-like protein 2	*Locusta migratoria*	ACU78069	13
60	LMI_GLEAN_10173735	phosphoglycerate mutase 2	*Zootermopsis nevadensis*	KDR20387	13
61	LMI_GLEAN_10053226	Rab GDP dissociation inhibitor alpha	*Zootermopsis nevadensis*	KDR21130	13
62	LMI_GLEAN_10105168	titin	*Tribolium castaneum*	XP_008191512	13
63	LMI_gi_329564865	glutathione S-transferase delta	*Locusta migratoria*	ADR30117	12
64	LMI_GLEAN_10096104	calcium-transporting ATPase sarcoplasmic/endoplasmic reticulum type-like	*Megachile rotundata*	XP_003707160	12
65	LMI_GLEAN_10109919	Hrp65 protein	*Zootermopsis nevadensis]*	KDR15347	12
66	LMI_GLEAN_10123367	protein disulfide-isomerase	*Schistocerca gregaria*	AEV89748	12
67	LMI_GLEAN_10192650	moesin/ezrin/radixin homolog 1	*Riptortus pedestris*	BAN21261	12
68	LMI_GLEAN_10051280	malate dehydrogenase, putative	*Pediculus humanus corporis*	XP_002424808	12
69	LMI_GLEAN_10154080	hexamerin-like protein 2	*Locusta migratoria*	ACU78069	12
70	LMI_GLEAN_10108970	succinyl-CoA ligase [ADP-forming] subunit beta, mitochondrial	*Tribolium castaneum*	XP_970725	12

Note: Detailed information about these proteins can refer to Supplementary Table S2.

**Table 2:** Differentially expressed proteins in the locust head.

No.	ID number in thelocust genomedatabase (version 2.0)	G/S (protein level of gregaria over solitaria)	BlastP			Function category
			Protein name	Accession No.	Species	
Up-regulated proteins in gregaria						
1	LMI_GLEAN_10019610	16.62	cuticle protein 1	XP_970381	*Tribolium castaneum*	Structure formation
2	LMI_GLEAN_10050722	5.56	cuticular protein 49Ae	XP_002033546	*Drosophila melanogaster*	
3	LMI_GLEAN_10021933	4.48	similar to beta1-tubulin	XP_967267	*Tribolium castaneum*	
4	LMI_GLEAN_10107594	4.45	cuticular protein RR-1 motif 45 precursor	BAB32485	*Bombyx mori*	
5	LMI_GLEAN_10196247	4.30	beta-actin	ACV32627	*Diabolocatantops pinguis*	
6	LMI_GLEAN_10170245	4.18	similar to Cuticular protein 62Bc CG1919-PA	XP_967979.1	*Tribolium castaneum*	
7	LMI_GLEAN_10056004	3.53	cytoplasmic actin A3b	AAL89657	*Helicoverpa zea*	
8	LMI_GLEAN_10105042	3.00	Endocuticle structural glycoprotein SgAbd-3	Q7M4E9	*Apis florea*	
9	LMI_GLEAN_10140130	2.31	profilin	NP_001011626	*Apis mellifera*	
10	LMI_GLEAN_10061897	2.11	actin-interacting protein 1-like isoform 1	XP_001943831	*Acyrthosiphon pisum*	
11	LMI_GLEAN_10172557	1.97	lambda-crystallin homolog	XP_001601340	*Nasonia vitripennis*	
12	LMI_GLEAN_10074080	1.51	Troponin I	EFN61242	*Camponotus floridanus*	
13	LMI_GLEAN_10124366	1.65	troponin t, invertebrate	XP_001655223	*Aedes aegypti]*	
14	LMI_GLEAN_10123370	4.61	electron transfer flavoprotein subunit alpha, mitochondrial-like	XP_003700429	*Megachile rotundata*	Energy metabolism
15	LMI_GLEAN_10127658	3.53	dehydrogenase/reductase SDR family member 11-like	XP_001947617	*Manduca sexta*	
16	LMI_GLEAN_10164303	3.28	3-ketoacyl-CoA thiolase, mitochondrial-like	XP_003488365	*Bombyx mori*	
17	LMI_GLEAN_10124634	2.8	V-type proton ATPase subunit B	P31401	*Acyrthosiphon pisum*	
18	LMI_GLEAN_10106737	2.26	probable isocitrate dehydrogenase [NAD] subunit beta, mitochondrial-like	XP_001607423	*Nasonia vitripennis*	
19	LMI_GLEAN_10137288	1.71	H+transporting ATP synthase subunit e	ABF51335	*Bombus impatiens*	
20	LMI_GLEAN_10120585	1.55	ATP synthase, subunit d	XP_002096273	*Bombus terrestris*	
21	LMI_GLEAN_10088301	1.53	probable pyruvate dehydrogenase E1 component subunit alpha, mitochondrial-like isoform 1	XP_003399781	*Drosophila yakuba*	
22	LMI_GLEAN_10187224	2.02	wingless protein	EDS27053	*Culex quinquefasciatus*	Regulation of gene expression
23	LMI_GLEAN_10002441	2.02	nucleoplasmin isoform 1-like protein	ABM55590	*Maconellicoccus hirsutus*	
24	LMI_GLEAN_10160841	1.93	Four and a half LIM domains protein 2	EGI61543	*Acromyrmex echinatior*	
25	LMI_GLEAN_10099907	1.77	3′-phosphoadenosine 5′-phosphosulfate synthase	XP_970563	*Tribolium castaneum*	
26	LMI_GLEAN_10172624	1.75	Putative beta-carotene-binding protein	P82886	*Drosophila willistoni*	
27	LMI_GLEAN_10131973	1.68	COP9 signalosome complex subunit 7A	EEB19483	*Pediculus humanus corporis*	
28	LMI_gi_1710156	1.66	juvenile hormone binding protein	AAC47391	*Locusta migratoria*	
29	LMI_gi_85816368	6.21	heat shock protein 20.6	ABC84493	*Locusta migratoria*	Environmental stress response
30	LMI_GLEAN_10065877	3.37	heat shock protein 60	ACO57619.	*Pteromalus puparum*	
31	LMI_GLEAN_10110738	1.54	heat shock protein 60	AEV89752	*Schistocerca gregaria*	
32	LMI_GLEAN_10190806	3.43	aspartate aminotransferase	EEB15916	*Pediculus humanus corporis*	
33	LMI_GLEAN_10095175	1.80	aspartate ammonia-lyase	EAT40064	*Aedes aegypti*	Digestion and absorption
34	LMI_GLEAN_10116472	1.56	alpha-amylase	ABC68516	*Blattella germanica*	
35	LMI_GLEAN_10043969	1.54	aspartate aminotransferase	AEX97005	*Allonemobius socius*	
36	LMI_GLEAN_10078839	1.65	chemosensory protein	CAJ01464	*Locusta migratoria*	Chemical sensing
37	LMI_gi_484000	4.23	choline transporter-like protein 4	NP_001086000	*Xenopus laevis*	Transporter
38	LMI_GLEAN_10154078	9.96	hexamerin-like protein 2	ACU78069	*Locusta migratoria*	
39	LMI_GLEAN_10057778	8.50	hypothetical protein TcasGA2_TC001323	EEZ98759	*Tribolium castaneum*	Others
40	LMI_GLEAN_10164915	5.57	predicted protein	EEH59002	*Micromonas pusilla*	
41	LMI_GLEAN_10175054	5.32	similar to ribosomal protein S28e	CAJ01883	*Tribolium castaneum*	
42	LMI_GLEAN_10098768	5.30	hypothetical protein	XP_00242276	*Pediculus humanus corporis*	
43	LMI_GLEAN_10126245	4.82	arylphorin hexamerin-like protein 2	AAX14951	*Romalea microptera*	
44	LMI_GLEAN_10154080	4.09	hexamerin-like protein 2	ACU78069	*Locusta migratoria*	
45	LMI_GLEAN_10025235	3.63	peroxiredoxin-like protein	ABV44727	*Phlebotomus papatasi*	
46	LMI_GLEAN_10042129	3.50	similar to conserved hypothetical protein	XP_970222	*Tribolium castaneum*	
47	LMI_GLEAN_10102266	3.24	AGAP006260-PD	EDO63843	*Anopheles gambiae str. PEST*	
48	LMI_GLEAN_10042900	2.80	similar to AGAP005134-PA isoform 1	EFA07428	*Tribolium castaneum*	
49	LMI_GLEAN_10080939	2.43	GK13357	EDW79295	*Drosophila willistoni*	
50	LMI_GLEAN_10061914	2.16	46 kDa FK506-binding nuclear protein, putative	EEB13519	*Pediculus humanus corporis*	
51	LMI_GLEAN_10097536	2.09	acidic ribosomal protein	CAA72658	*Ceratitis capitata*	
52	LMI_GLEAN_10118445	2.08	major allergen Bla g 1.02	AAD13531	*Blattella germanica*	
53	LMI_GLEAN_10113524	2.06	c-1-tetrahydrofolate synthase, cytoplasmic-like	XP_003700207	*Megachile rotundata*	
54	LMI_GLEAN_10147206	2.00	hypothetical protein LOC100160882	XP_001951692.	*Acyrthosiphon pisum*	
55	LMI_GLEAN_10095481	1.98	myosin 1 light chain	AAV91412	*Lonomia obliqua*	
56	LMI_GLEAN_10124558	1.82	GF22728	EDV33005	*Drosophila ananassae*	
57	LMI_GLEAN_10130423	1.72	maternal protein exuperantia-like	XP_003697468.	*Apis florea*	
58	LMI_GLEAN_10154525	1.61	transketolase-like protein 2-like isoform 1	XP_003493512	*Bombus impatiens*	
59	LMI_GLEAN_10119040	1.57	pentatricopeptide repeat-containing protein 2-like	XP_001946785	*Acyrthosiphon pisum*	
60	LMI_GLEAN_10073596	1.56	hypothetical protein	CAJ01469	*Locusta migratoria*	
61	LMI_GLEAN_10181062	1.55	hypothetical protein LOC100169018	XP_001946070.	*Acyrthosiphon pisum*	
62	LMI_GLEAN_10135691	1.54	putative leukotriene A4 hydrolase	EFX86132	*Daphnia pulex*	
63	LMI_GLEAN_10071089	1.51	hexamerin 4 precursor	NP_001164245	*Tribolium castaneum*	
64	LMI_GLEAN_10187958	1.51	conserved hypothetical protein	XP_002416013	*Ixodes scapularis*	
Down-regulated proteins in gregaria						
65	LMI_GLEAN_10143558	0.106	hexamerin-like protein 1	ACU78068	*Locusta migratoria*	Digestion and absorption
66	LMI_GLEAN_10175495	0.459	ubiquitin carboxyl-terminal hydrolase isozyme L5	XP_002431967	*Pediculus humanus corporis*	
67	LMI_GLEAN_10051974	0.468	similar to putative carboxypeptidase A-like	EFA05749	*Tribolium castaneum*	
68	LMI_GLEAN_10120545	0.557	serine protease-like protein	CAA70820	*Schistocerca gregaria*	
69	LMI_GLEAN_10054296	0.567	beta-1,4-endoglucanase 1	AAF80584	*Panesthia cribrata*	
70	LMI_GLEAN_10109880	0.617	similar to cathepsin b	XP_974220	*Tribolium castaneum*	
71	LMI_GLEAN_10192264	0.653	1,4-alpha-glucan-branching enzyme-like	XP_003707245	*Megachile rotundata*	
72	LMI_GLEAN_10099118	0.513	similar to pre-mRNA-splicing helicase BRR2	XP_970554.1	*Tribolium castaneum*	Regulation of gene expression
73	LMI_GLEAN_10170704	0.588	Splicing factor 3B subunit	EEB16979	*Pediculus humanus corporis*	
74	LMI_GLEAN_10196136	0.599	ubiquitin-conjugating enzyme E2 variant 2-like isoform 1	XP_003398336	*Apis mellifera*	
75	LMI_GLEAN_10002265	0.660	Proteasome subunit alpha type-4	EFN87452	*Harpegnathos saltator*	
76	LMI_GLEAN_10081266	0.666	Arginine/serine-rich-splicing factor RSP31	EEB11604	*Pediculus humanus corporis*	
77	LMI_GLEAN_10133889	0.464	takeout-like	BAH71589	*Acyrthosiphon pisum*	Chemical sensing
78	LMI_gi_311063281-D1	0.490	protein takeout-like	XP_001947537	*Acyrthosiphon pisum*	
79	LMI_GLEAN_10133888	0.562	protein takeout-like	XP_001950706	*Acyrthosiphon pisum*	
80	LMI_GLEAN_10109545	0.479	NADPH--cytochrome P450, putative	EEB11242	*Pediculus humanus corporis*	Immunity and defense
81	LMI_GLEAN_10117209	0.593	glutathione S-transferase sigma 1	AEB91973	*Locusta migratoria*	
82	LMI_GLEAN_10042954	0.638	similar to DNA-damage inducible protein	XP_969775	*Tribolium castaneum]*	
83	LMI_GLEAN_10138023	0.663	importin subunit beta-1-like isoform 1	XP_001599381	*Nasonia vitripennis*	Transporter
84	LMI_GLEAN_10082914	0.663	similar to vesicle docking protein P115	EFA08682	*Tribolium castaneum*	
85	LMI_GLEAN_10168539	0.663	Reticulon-1	EFN73447	*Camponotus floridanus*	Structure formation
86	LMI_gi_159434	0.370	conserved hypothetical protein	EEB10389	*Pediculus humanus corporis*	Others
87	LMI_GLEAN_10002280	0.558	40S ribosomal protein S17	EEB10115	*Pediculus humanus corporis*	
88	LMI_GLEAN_10066930	0.565	similar to eukaryotic translation initiation factor 3	EFA00209	*Tribolium castaneum*	
89	LMI_GLEAN_10131870	0.602	RNA-binding protein Nova-2-like	XP_003494948	*Bombus terrestris*	
90	LMI_GLEAN_10127412	0.665	nipsnap	EAT48786	*Aedes aegypti*	

Notes: Proteins are classified roughly into different categories according to their functions. Hexamerin-like proteins are marked in thick black words because of their high abundance. Detailed information about these proteins is listed in Supplementary Table S3.

**Table 3:** Primers used for qRT-PCR and RNAi.

Gene name	ID number in the locust genome database (version 2.0)	Primer sequence (5′⟶3′)	Product length (bp)
*qRT-PCR*			
*CSN7A*	LMI_GLEAN_10131973	AGAATCGTCGGCTGAAACATAA	184
		CCAGAACTACTGCGAATCCCT	
*JHBP*	LMI_gi_1710156	AAAGTATTCCTGACACGCCAAC	148
		GCTCCACCGTCTCCTTATCC	
*PAPSS*	LMI_GLEAN_10099907	CATCACATAGAGGACACCCTTACA	135
		GCTCAAGTGGGGTTAGACGATA	
*CTL4*	LMI_gi_484000	CTCAACAACAGCATCCACGACG	118
		GGGTCTTGGTTGCGATGTCC	
*Hexa2*	LMI_GLEAN_10154078	CAACGCCCTGACCATCTCC	151
		GGGACCAATGAAGACTCGGAC	
Hexa2^*^	LMI_GLEAN_10154080	AGAGGAGGATCAGGGACGC	294
		AGATGGAAATGACTCGCTTGG	
*actinA3a*	LMI_GLEAN_10056004	TGAGCGATTCAGGTGCCC	283
		CAAGATAGACCCTCCAATCCAAA	
*ETFA*	LMI_GLEAN_10123370	ACCTATAACGCAAAATGCCATAA	166
		AGGGGTGAAGCCCAGAAAA	
*Arylphorin*	LMI_GLEAN_10126245	ACCCCTGTGCGTGCTGAAG	257
		ATGTGTCCGAAGATGGAAATGAG	
*takeout-like*	LMI_GLEAN_10133888	ACTCCGCCAAGACGAAATACA	166
		TGCTCCATCCAGTCCTCCA	
*V-ATPase*	LMI_GLEAN_10124634	TTGCCATCACTCAGTCGTCTCA	115
		GCACATCTTTCCCAATAGCGTAAC	
*ATPsyn-d*	LMI_GLEAN_10120585	AGAAAATCCGCCCAAAATAGA	253
		TTGCTCAAACGGTAAGACTGC	
*RSP31*	LMI_GLEAN_10081266	TGCCAGGCTTCAGTAGTGTAGG	141
		CAAATCCATAGTTCTTCACCACG	
*P450*	LMI_GLEAN_10109545	TGACGAGCCTCAAAGACATCC	159
		TGCCCAGCCCAAATACCG	
*rp49*	LMI_GLEAN_10126536	CGTAAACCGAAGGGAATTGA	209
		GAAGAAACTGCATGGGCAAT	
*RNAi*			
*CSN7A*	LMI_GLEAN_10131973	GCACCCTACTACCGAGCAT	316
		CAGCAGGTTGGATGTCT	
*GFP*		CACAAGTTCAGCGTGTCCG	420
		GTTCACCTTGATGCCGTTC	

Full names: *CSN7A*, *COP9 signalosome complex subunit 7A* (GenBank accession NO. KM396884); *JHBP*, *juvenile hormone binding protein*; *PAPSS*, *3*′*-phosphoadenosine 5*′*-phosphosulfate synthase; CTL4*, *choline transporter-like protein 4*; *Hexa2*, *hexamerin-like protein 2*; *actinA3a*, *cytoplasmic A3a actin*; *ETFA*, *mitochondrial-like electron transfer flavoprotein subunit alpha*; *Arylphorin*, *arylphorin hexamerin-like protein 2*; *V-ATPase*, *V-ATPase subunit B*; *ATPsyn-d*, *ATP synthase, subunit d*; *RSP31*, *Arginine/serine-rich-splicing factor RSP31*; *P450*, *NADPH—cytochrome P450*; *rp49*, ribosomal protein 49; *GFP*, *green fluorescent protein* (cloned from pEGFP-N1 plasmid vector, Clontech , Mountain View, CA, USA; GenBank accession NO. U55762)
