# Supplementary material for: Proteomic analysis reveals that COP9 signalosome complex subunit 7A (CSN7A) is essential for the phase transition of migratory locust
Source: Sci Rep. 2015 Jul 27;5:12542. doi: 10.1038/srep12542 (PMC4515600; doi:10.1038/srep12542)
Supplement: Supplementary Information [file srep12542-s1.pdf]

Proteomic analysis reveals that COP9 signalosome complex subunit 7A (CSN7A) is essential for the phase transition of migratory locust

Xi-Wen Tong[1, 2, #], Bing Chen[2, #], Li-Hua Huang[1*], Qi-Li Feng[1], Le Kang [2*]

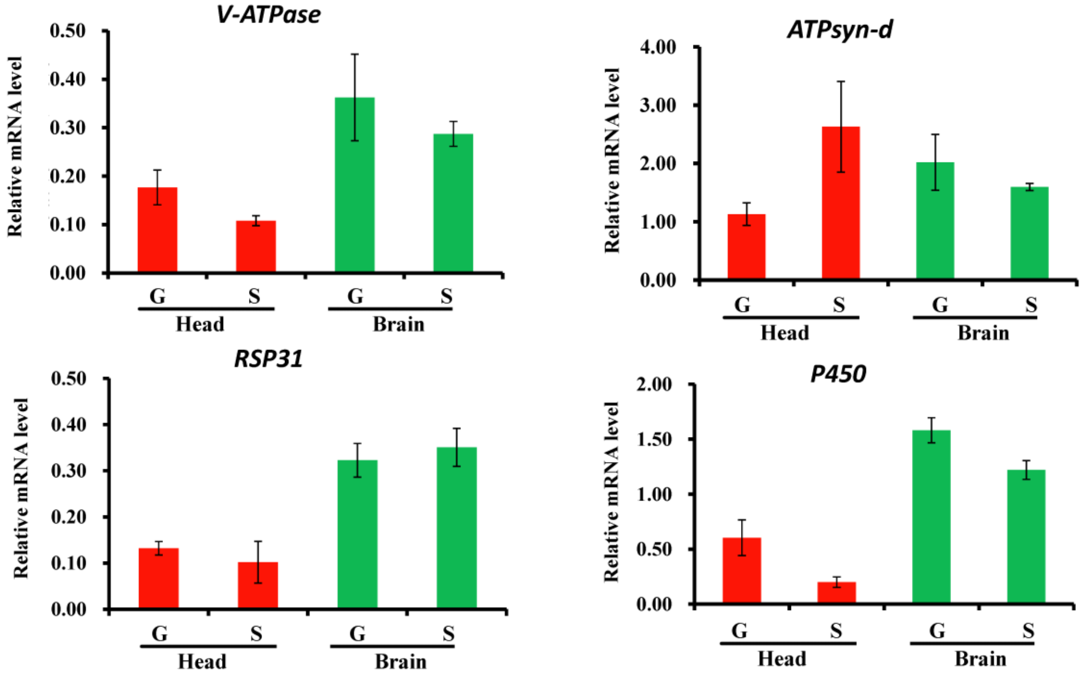

Supplementary Figure S1. The mRNA levels of selected protein genes in both the head and brain tissues. These genes revealed constant mRNA levels between the two phases. Abbreviatins: *V-ATPase subunit B, V-ATPase*; *ATPsyn-d, ATP synthase subunite d*; *arginine/serine-rich-splicing factor RSP31, RSP31*; *NADPH--cytochrome P450, P450*.

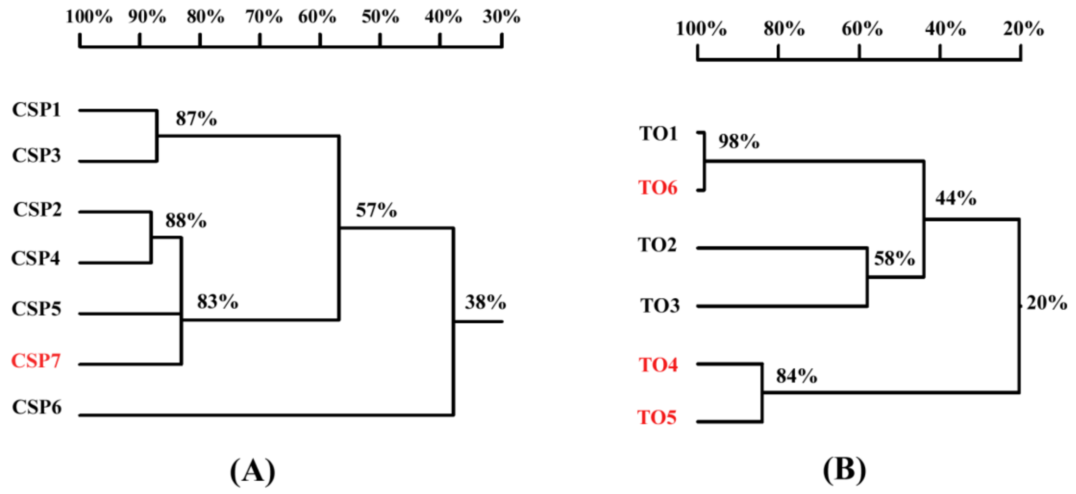

Supplementary Figure S2. Sequence similarity of CSP and takeout in the migratory locust. Amino acid sequence similarity of CSP (A) and takeout (B). Full-length protein sequences were aligned by DNAMAN software, and the similarity was calculated. The newly identified proteins by iTRAQ were marked in red. Abbreviations: CSP, chemosensory protein; TO, takeout-like protein. Numbers of ID in the locust genome database (version 2.0) and Genbank are listed as follows: CSP1(Gu722576); CSP2 (Gu722577);CSP3 (Gu722578);CSP4 (Gu722579);CSP5 (CO835786);CSP6 (CO852124);CSP7 (CAB65179, LMI_GLEAN_10078839); TO1 (GU722575); TO2 (CO856064); TO3 (CO825835); TO4 (KM396886, LMI_GLEAN_10133889); TO5 (KM396885, LMI_GLEAN_10133888); TO6 (KM503135, LMI_gi_311063281-D1).

Supplementary Table S1. Proteins assigned to the "O" cluster in COG analysis

Accession no. in the locust genome database	COG number	BlastP	Sequence
**Heat shock protein chaperone**			
LMI_GLEAN_10065878	COG0459	60 kDa heat shock protein, mitochondrial-like [Nasonia vitripennis]	MALIASQYSNYIPRYCDAKLTLEFAKRACPEQHGVLASVSGRALATRFAYFKRQLRVAISRDGRNVILEQSWGSPKITKDGVTVAKGVELKDKFQNIGAKLVQDVANNTNEEAGDGTTTATVLARAIAKEGFEKISKGANPIEIRRGVMMAVEAIIDHLKTLSKPVTTPEEIAQVATISANGDQKVGELISEAMKKVGKEGVITVKDGKTLQDELEVIEGMKFDRGYISPYFINTSKGAKVEFQDALLLLSEKKISSVQSIIPALELANSQRKPLVIIAEDVDGEALSTLVVNRLKIGLQVAAVKAPGFGDNRKATLQDIAIATGGIVFGDEGNPVKLEDLQPSDLGQVGEIVITKDDTLMLKGKGNKTDIDRRAEQLRDQIDSTTSEYEKEKLQERLARLASGVAVLKVGGSSEVEVNEKKDRVNDALNATRAAVEEGIVPGGGTALLRCGPILAKLQPNNVDQATGIDIVKKALRMPCMQIAKNAGVDASVVVSKVEDATGDMGYDALNNEYVNLIERGIIDPTKVVRTALTDAAGVASLLTTAEAVVTEIPKEETAPAGMGGMGGMGGMGGMGGMM
LMI_gi_37993866	COG0443	heat shock protein 70 [Locusta migratoria]	KAPAVGIDLGTTYSCVGVFQHGKVEIIANDQGNRTTPSYVAFTDTERLIGDAAKNQVAMNPSNTIFDAKRLIGRRFDDQAVQSDMKHWPFKVINDSGKPKIQVQYKGETKTFFPEEVSSMVLTKMKETAEAYLGKNVSNAVITVPAYFNDSQRQATKDAGAIAGLNVLRIINEPTAAAIAYGLDKKVSGHGERNVLIFDLGGGTFDVSILTIEDGIFEVKATAGDTHLGGEDFDNRMVNHFVQEFKRKYKKDLTTNKRALRRLRTACERAKRTLSSSTQASIEIDSLYEGIDFYTSITRARFEELNADLFRSTMEPVEKALRDAKMDKAQIHDIVLVGGSTRIPKVQKLLQDFFNGKELNKSINPDEAVAYGAAVQAAILAGDKSEEVQDLLLLDVTPLSLGIETAGGVMTTLIKRNTTIPTKQTQTFTTYSDNQPGVLIQVYEGERAMTKDNNLL

			GKFELTGIPPAPRGVPQIEVTFDIDANGILNVTAVEKSTGKENKITITNDKG<br>RLSKEEIERMVNEAERYRAEDEKQKATIAAKNGLESYCFNMKSTVEDEKL<br>KDKISDSDKQTILDKCNEVIRWLDANQLAEKEEFEEKQKELEQICNPIITKL<br>YQGAGGAPGGMPGGFPGGFPGAGGAAAGGAGAGGAGPTIEEVD
LMI_gi_93278396	COG0326	heat shock protein 90 [Locusta migratoria]	MQDAAEVETFAFQAEIAQLMSLIINTFYSNKEIFLRELISNSSDALDKIRYES<br>LTDPSKLDSGKDLWIKIVPNKSERTLTIIDTGIGMTKADLVNNLGTIAKSGT<br>KAFMEALQAGADISMIGQFGVGFYSAYLVADKVTVASKHNDDEQYLWES<br>SAGGSFTIRPDPGEPLGRGTKITLYVKEDQTEFLEERKIKEIVKKHSQFIGYP<br>IKLVVEKERDKELSEEEEEEEKKEGGEEGDNESKPKIEDVGEDEEDESGDK<br>KKKKKKTIKEKYLEDEELNKTKPIWTRNPDDISQEEYGEFYKSLTNDWEE<br>HLAVKHFSVEGQLEFRALLFIPRRAPFDLFENKKRKNNIKLYVRRVFIMDN<br>CEDLIPEYLNFIKGVVDSEDLPLNISREMLQQNKILKVIRKNLVKKCLELFE<br>ELTEDADTYKKFYEQFSKNLKLGIHEDSTNRKKLSDLLRYATSASGDETCS<br>LKDYVARMKENQKHIYYITGENKDQVANSSFVERVKKRGFEVVYMTEPI<br>DEYVVQQMKEYDGKQLVSVTKEGLELPEDEEEKKKREEDKAKFENLCKV<br>MKDILDKKVEKVVVSNRLVESPCCIVTSQYGWTANMERIMKAQALRDTS<br>TMGYMAAKKHLEINPDHPVMETLRQKAEADKNDKAVKDLVMLLFETAL<br>LSSGFTLEEPQVHASRIYRMIKLGLGIDEEEPQAAEEEKVDAEMPPLEGDN<br>EDASRMEEVD
LMI_GLEAN_10164084	COG0443	Heat shock 70 kDa protein cognate 5 [Zootermopsis nevadensis]	MLFVCKYVGRKALDSTYYVTETASKNQFSTLLSKVATPALGTNQNVTQF<br>RHKSEGVKGAVVGIDLGTTNSCVAVMEGKQAKVIENSEGARTTPSVVAFT<br>KDGERLVGMPAKRQAVTNSANTFYATKRLIGRRYNDAEVQKDMKTVSY<br>KIVKASNGDAWVEGSDKKMYSPSQIGAFVLMKMKETAESYLNTSVKNAV<br>ITVPAYFNDSQRQATKDAGQIAGLNVLRVINEPTAAALAYGMDKTEDKV<br>VAVYDLGGGTFDISILEIQKGVFEVKSTNGDTFLGGEDFDNVLVNHLVAEF<br>KKDQGIDITKDPMAMQRLKEAAEKAKIELSSSLQTDINLPYLTMDSSGPKH

			MNLKLTRSKFESLVGDLIKRTIQPCQKAVQDAEVNKRDIGEVLLVGGMTRMPKVQSTVQEIFGRQPSRSVNPDEAVAVGAAIQGGVLAGDVTDVLLLDVTPLSLGIETLGGVFTRLISRNTTIPTKKSQASINLTVFSTAADGQTQVEIKVHQGEREMAADNKLLGQFTLVGIPPAPRGVPQIEVTFDIDANGIVHVSARDKGTGKEQQIVIQSSGGLSNDEIENMIRNAEAFAAQDKKKKDRVEAVNQAESITHDIESKMEEFKDQLPKEECDKMKEEIQKVRELVAKKDETDPEEIRKAAGSLQQASLKLFEMAYKKKLDLSLLQIIDVECADNSSNKSSFIILNFKEVLQINKKASGGWEVPNGFGDEAAVKVKGVLLRCMLHHRIVHIALGHSLTVAIHPDREPMAAERDSSSGSSGSSTEKPEEEEEKKAKKE
LMI_gi_241997152	COG0443	ER protein gp78 [Locusta migratoria]	MRVLSLSAFLAVIIGISLAKEEKNKDVGTVIGIDLGTTYSCVGVYKNGRVEIIANDQGNRITPSYVAFTPDGERLIGDAAKNQLTTNPENTVFDAKRLIGREWTDPTVQHDIKFFPFKVKEKNSKPHIQVATSQGEKMFAPEEISAMVLGKMKETAEAYLGKKVTHAVVTVPAYFNDAQRQATKDAGTISGLVVMRIINEPTAAAIAYGLDKREGEKNVLVFDLGGGTFDVSLLTIDNGVFEVVSTNGDTHLGGEDFDQRVMDHFIKLYKKKKGKDIRKDNRAVQKLRREVEKAKRALSSGHQVRIEIESFFEGDDFSETLTRAKFEELNMDLFRSTMKPVQKVLEDADMNKNDVDEIVLVGGSTRIPKVQQLVKEFFGGKEPSRGINPDEAVAYGAAVQAGVLSGEQDTDAIVLLDVNPLTMGIETVGGVMTKLIPRNTVIPTKKSQIFSTASDNQHTVTIQVYEGERPMTKDNHLLGKFDLTGIPPAPRGVPQIEVTFEIDANGILQVSAEDKGTGNREKIVITNDQNRLTPDDIERMIKDAEKFADDDKKLKERVEARNELESYAYSLKNQLSDKEKLGAKVSGSDKTTMEEAIEEKIKWLEANQDASTEEFKKQKKELEDVVQPIIAKLYQSSGGPPPSGSSEDDDLKDEL
LMI_gi_241997150	COG0443	ER protein gp78 [Locusta migratoria]	VKPNMRVIQLFCIAFLVNAVFCSKEEKNVGTVIGIDLGTTYSCVGVYKNGRVEIIANDQGNRITPSYVAFTPDGERLIGDAAKNQLTTNPENTVFDAKRLIGRDWSDPAVQHDIKFFPFRVKEKNSKPYIQVATSQGDKEFAPEEISAMVLGKMKETAEAYLGKKVTHAVVTVPAYFNDAQRQATKDAGTIAGLVVMRIINE

			PTAAAIAYGLDKKEGEKNVLVFDLGGGTFDVSLLTIDNGVFEVVATNGDTHLGGEDFDQRVMDHFIKLYKKKKGKDIRKDNRAVQKLRREVEKAKRALSSSHQVRIEIESFFEGEDFSETLTRAKFEELNMDLFRSTMKPVQKVLEDAGMTKKEVHEIVLVGGSTRIXXXXXXXCEGIAGKEPSRGINPDEAVAYGAAVQAGVLSGEQDTDAIVLLDVNPLTLGIETVGGVMTKLIPRNTVIPTKKSQIFSTAADNQHTVTIQVYEGERPMTKDNHLLGRFDLTGIPSAPRGVPQIEVTFEIDANGILQVSAEDKGTGNREKIVITNDQSRLSPEDIERMIKDAEIFADEDKKLKEHVEARNELESYAYSLKNQINDKEKLGAKLSDSDKTTIEEAVDEAVKWLEDNQNAETEEFKQQKKKLEDVVQPIISKLYQGAGAPPPDADTDRDEL
LMI_GLEAN_10174153	COG0443	Heat shock 70 kDa protein 4L [Zootermopsis nevadensis] Sequence ID: gb|KDR19282.1|	MSVIGIDFGNESCYIAVARAGGIETIANDYSLRATPSCVAFSGKNRILGVAAKNQMVTNMKNTIYGFKRLLGRKFNDPYHLPYRIVQHGSGGIGIKVNYLDEEHVFSPEQITAMLFTKLKDISEVALKTKVNDCVISVPSFFTNAERKALLDAASIAGLNVLRLMNETTATALAYGIYKQDLPAPEEKPRNVVFVDCGHASLQVSACAFHKGKLKMLASAADPQLGGRDIDEILANHFCVDFQSRYRIDPRNNPRAYLRLTTEVEKLKKQMSANSMNLPMNIECFMDDKDVQASMNRAEMEKLCAHLIQRVEHTMRKCLQDSGLRLDEIHSVEIVGGSSRIPAIKHLIETVFGKLPSTTLNQDEAVSRGCALQCAMLSPAIRVREFSVTDIQTYPIKLVWDASMGEDGSVPEDTENVLPVKRRRLRSIWNRFKVTMFDYFSQRVASTQKEMEVYTQNHPVPFSKMLTFFRKEPFSIKAYYVGNIPYPDPYIGQFIVKDVKPTPEGESQKVKVKVRVNLHGILLISSASLTEKKEATEQDSQEDTSMENDVQSSASQTGSQNDAQAEQAANQQNHIGGEDDAEGQGSTEKKDASKKKRIVKTVDLPIESYTHGISQLDLNNYIEQECKMIASDRQEKERIDARNALEEYVYDLRGKLNSEEELATFVNDADRTSLSQQLDDTENWLYEEGEDCNRQVYVDRLTSLKTLGEPIKMRRQEFEQRSWILEEFGAALQLTRKAVDQWRAGEERYNHLAEADMQQVEQKVEQCHRWLEEKRSQLAATPRTQNPPITVAQIRQEKQAFDSAVSPVLNKPKPKVDPPPQQNKEGETKDAHGDSTKTNSQNEQTDEKMDVE

LMI_GLEAN_10097218	COG0443	heat shock protein 70 [Oxya chinensis] Sequence ID: gb|AFN08643.1|	QATKDAGAIAGLNVLRIINEPTAAALAYGLDKGFVGERNVLIFDLGGGTFDVSVLTIDEGSLFEVRSTAGDTHLGGEDFDNRLVAHLAEEFRRKFKRDLNDSPRALRRLRTAAERAKRELSATTESTIQVDALMDGIDFYTKITRARFEELCMDLFRSTLPLVEKALADANLSKAAVHDVVLVGGSTRIPRIQAMLREMFHGKQLCASINPDEAVAYGAAVQAAIISGDKSANLQEVLLVDVTPLSLGIETAGGIMTAVVERNTRIPCTLTKTFSTYSDNQPAVTVQIFEGERALTKDNNLLGTFDLVGIPPAPRGVPRIDVSFDIDANGILNVSARDASTGRTQAITIRNDGGRLTRDQIERMVADAERFRAQDMIARERVQERHRLEDYALAVKRALDEAGSRLGDEGERQRATTACNETLMWLEAVPDASMDDIKRRFNELQAICMPIMTRLHQSG
LMI_GLEAN_10165517	COG0326	Heat shock protein 75 kDa, mitochondrial [Cerapachys biroi] Sequence ID: gb|EZA55266.1|	GSSDRHEFQAETRMLLDIVAKSLYSDKEVFIRELVSNASDALEKLRYRNLLAGVGAEDAGEIHIATDKLARTFTIQDTGVGMTKEELISNLGTIARSGSKAFREQLKEKGSEASNIIGQFGVGFYSCFMVAEKVEVFTRSCEPGSPGYRWISDGSGTYEIQEAEGVQQGTKIVIHLKTECREYGDEETVNNVIKKYSNFVGSPVYVNGKKVNTIQPLWLMDSKEITPQMHDEFYRFVGNAYDRPRFTLHYKTDAPLSVRALIYFPEGKPGLFEMSRDMDVGVALYSRKVLIKSKADNILPKWLRFVKGVVDSEDIPLNLSRELLQNSALIRKLRTVLVNRILRFLHERSTKDTEQYDAFYKDYGLFLKEGIVSSEEQLQKEEIAKLLRFESSSKPVGEKVTLPEYCSRLKPAQRDIFYLAAPSRALAETSPYFEALKQKDVEVLFCYEPYDELVLMQLRQFDRHNLTSVEKEMRQDKEPEDLSNLGSDSLSRTSVEALLPWVKSVLAGKVHNVRVTQRLDSHPCVVTVEEMAAARHFIRTQSHQLPEDARFTLLQPQLELNPRHPIIKKLALLRESNPKLAELVTKQLFANAMVSAGLMEDPRTLVTSMNELLTLALEKH
LMI_GLEAN_10036224	COG0443	Heat shock protein 70 similar to Heat shock protein 68; K03283 heat shock 70kDa	SQVAMNPKNTVFDAKRLIGRRFDDPKIQDDMKHWPFTVVAEADKPKIQVTRTSLQSIQQRISFISNRKDILDEFCYLGGKITHNGRSTEDIKFRMAMGRKAFLKKRNLLTLNTNLSIRKSLLKAFVYGLDKNLQGDKNVLIFDLGGGTFDVS

		protein 1/8 HSP7B_DROME Heat shock 70 kDa protein cognate 2 OS=Drosophila melanogaster GN=Hsc70-2 PE=1 SV=2 Q45XA5_BEMTA 70 kDa heat shock protein (Fragment) OS=Bemisia tabaci GN=hsp70 PE=2 SV=1	VLAISEGSLFEVKSTAGDTHLGGEDFDNRLVQHLADEFQRKHRKDMRAN ARALRRLRTAAERAKRTLSSSTEASLEIDALHDGIDFYAKVTRARFEELCM DLFRQAYLKMFSTSLDETYMDHAIPEDSSASATSDLYSVFVCVRQDFFCG KTLNLSINPDEAVAYGAAVQAAILSGDTSSQIQDVLLVDVAPLSLGIETAG GVMTKIVERNARIPCKQKQTFTTYSDNQPAVTIQVFEGERAMTKDNNLLG TFNLTGIPPAPRGVPKIEVTFDLDANGILNVSATESGSGRSERITIQNDKGRL SKAEIERMLADAEKFRAEDERQRARVEARNKLEAYALSLKQAVEDAGSK LSDADKATVREQSAEALKWLDSNSLAEQEEFDDRYKQLSAACSPIMAKLH QAGGGAGGAKGP
LMI_gi_85816372	COG0484	heat shock protein 40 [Locusta migratoria]	MGKDYYKILGVPKSATDDEIKKAYRKLALKYHPDKNKSPGAEERFKEVA EAYEVLSDKKKRDVYDKFGEEGLKGGAPGASEGGGPGFTYTFHGDPRAT FAQFFGSSSPFQAFFEMSGPGGNRIFDDMELDDPFTSMGMKSGGPAFRSHS FNYHPGGSPTRNKDKIQDAPIEHDLYVTLEDILRGCTKKMKISRKVLQPDG STRKEDKVLTISVKPGWKAGTKITFQREGDQGRNKIPADIVFIIRDKPHPLF KREGSDIRFTSKITLKQALCGTVIQVPTLTGEKIPINLTNEIVKPTTVKRIQG HGLPFPKEPSRKGDLLVSFDIKFPDVLSQSVRDILYDTLPN
LMI_GLEAN_100 88776	COG5269	protein folding; Biological Process GO:0031072; heat shock protein binding; Molecular Function GO:0051082; unfolded protein binding; Molecular Function IPR001623; Heat shock protein DnaJ, N-terminal IPR003095; Heat shock protein DnaJ hypothetical protein; K09522	ERDDEDDSPNAKLFDPDDVALLRTLDPKEWKLQDHYAVLGLSKLRYKAT EEDIKKAYRIKVLLHHPDKRKAAGEDVRPDDDYFTCITKAYEILGNPQKRR SFDSIDPVFKDPAPPSNDHTKKHFFKVFGEAFALEARFSEKLPVPVIGGPDD PRESVEKFYDFWYNFESWREFSYLDEEDKEKGQVREERKWIEKQNKFYR AKRKKEDMTRIRNLVDTAYSLDPRVAKFKQEDKDRKEAAKQAKREAAR ARQAEEERKLQEAQEIEQRKKAEIEAIEKARLSALKAEREAQKKTLKVKR ATLRKLVKSNDYYVENEAERVRHMASLEKLCEVLKPEQFDELLEKMTLG GRSVYISAIEETEKHIEEERQALLAASQRNSGASGAQGSKKGTQVNWTTEE LHLLIKAVNLFPAGTNQRWEVVANFINQHDTTSTNKKTAKDVLAKAKSL QSSDFSRNVLKETVNKQVYHMFEKEHSTGDKSASATERLDVPTEDIPWTA

		DnaJ homolog subfamily C member 2 DNJC2_MACFA DnaJ homolog subfamily C member 2 OS=Macaca fascicularis GN=DNAJC2 PE=2 SV=1 B4PE72_DROYA GE22360 OS=Drosophila yakuba GN=GE22360 PE=4 SV=1	AEQKLLEQALKTYPNSTPNRWDRIAECIPTRSKKQCILRYKEIAEMVKAKKAAQ
LMI_GLEAN_10064404	COG0484	protein folding; Biological Process GO:0031072; heat shock protein binding; Molecular Function GO:0051082; unfolded protein binding; Molecular Function IPR001305; Heat shock protein DnaJ, cysteine-rich domain IPR001623; Heat shock protein DnaJ, N-terminal IPR002939; Chaperone DnaJ, C-terminal IPR003095; Heat shock protein DnaJ IPR008971; HSP40/DnaJ peptide-binding similar to DnaJ homolog subfamily A member 2 (HIRA-interacting protein 4) (Cell cycle	MADTKLYDLLGVSRNASDLEIKKAYRKLAKEFHPDKNPEAGDKFKEISFAYEVLSDPKKRSTYDRVGLKGMQEGAHEGHFGSDDLFSQIFGGGLFGMGMGPSMRARRRHRGEDTIHTLKVTLEDLYNGKTSKLQLSKNVICTTCNGKGGRSGTTHTCRTCAGCGYKVTYRQLGPGMTQQLQSRCPTCSGEGEVIHEKDRCTTCRGKKVVNETKILEVHVDKGMKESQKIFFRGEGDQQPEVEPGDVIIVLQQKPHEKFQRTGDDLIMTHAISLTEALCGFNMTLKHLDGRDLVIKHPPGQVIKPGDIRGIEGEGMPQYRNPFERGNLYIKFDVLFPSNHFANEVTLKELEALLPPRPTFHLPHGDNVEEVDLNEYDPNDRSSGGPGRQEAYASDDEDYGGGSGGIQCAHQ

		progression restoration gene 3 protein) (Dnj3); K09503 DnaJ homolog subfamily A member 2 DNJA2_BOVIN DnaJ homolog subfamily A member 2 OS=Bos taurus GN=DNAJA2 PE=2 SV=1 C7AQY9_BOMMO DnaJ-1 OS=Bombyx mori PE=2 SV=1	
LMI_GLEAN_10065877	COG0459	60 kDa heat shock protein, mitochondrial [Zootermopsis nevadensis	MASNKRKLSDVKIEQLANITELVWNEFMDEFSGGESHHFHVSTQVRIQMQTTVIEEEEQMYRLPRVLRSNALQQIYQVRSFAKDVRFGPEVRALMLQGVDILADAVAVTMGPKKAYTRNYATEFDIQGVPTILSTQNISGTITAIGKRLAPVSMYGWDP
LMI_GLEAN_10145214	COG0484	protein folding; Biological Process GO:0031072; heat shock protein binding; Molecular Function GO:0051082; unfolded protein binding; Molecular Function IPR001305; Heat shock protein DnaJ, cysteine-rich domain IPR001623; Heat shock protein DnaJ, N-terminal IPR002939; Chaperone DnaJ, C-terminal IPR003095; Heat shock protein DnaJ IPR008971; HSP40/DnaJ	MDDLPKGQAHRQTLIKSHTYKLLQRWSLLQEKRKKGEEKGKDMGFKEVEKESFQSQEWMDLPLGGKKEGCFHRPANMVKETTYYDVLGVKPNCTLDELKKAYRKLALKYHPDKNPNEGERFKQISQAYEVLSNPEKRRIYDQGGEQALKEGGGNGFSAPMDIFDMFFGQAFGGRGRGPRARERHGKDVIHQLSVSLEELYKGAVRKLALQKNVICDKCEGRGGKKGAVETCPACHGNGMQVHIQQLGPGMIQQIQSMCSECRGQGERINPKDRCKQCQGKKTIRDRKILEVHVDKGMVDGQKIVFGGEGDQEPGLEPGDIIIVLDEKEHETLKRSGSDLIMRMNIELVESLCGFQRVIRTLDDRDLVITCIPGEVTKHGDVKCIMNEGMPQYKNPFEKGRLIIQFLVNFPSTLPPEVIPQLENCLPPRPESMIPDGAEECLLVEMDPEQEARRREYKNAYDEDEAGTGPSRVQCATH

		peptide-binding similar to DnaJ homolog subfamily A member 1 (Heat shock 40 kDa protein 4) (DnaJ protein homolog 2) (HSJ-2) (HSDJ); K09502 DnaJ homolog subfamily A member 1 DNJA1_HUMAN DnaJ homolog subfamily A member 1 OS=Homo sapiens GN=DNAJA1 PE=1 SV=2 Q2F5I7_BOMMO DnaJ-2 OS=Bombyx mori PE=2 SV=1	
LMI_GLEAN_101 35184	COG0459	T-complex protein subunit zeta [Locusta migratoria]	MAAISLLNPKAEFARAAQALAVNISAAKGIQDVMKTNLGPKGTMKMLVS GAGDIKITKDGNVLLHDMQIQHPTATLIARASTAQDDMTGDGTTSTVLMI GELLKQADLYISEGMHPRVITEGYELARARTLETLEKIKIKIEPTRENLLNV ARTALLTKVHKQLAELLTEVCVDAVLAIRQEGKEIDLHMIELMEMQHKTE TETTLVHGLVLDHGSRHPDMPKRVENAYILTCNVSMEYEKSEVNSGFFYK SAEEREKLVAAEREFIEQRVKKVVELKKKLCDNTDKNFVVINQKGIDPMS LDMLAKEHIIGLRRAKRRNMERLALACGGVAVNSVDDLTEESLGFAGLV YEHVLGENKFTFVEECKGPKSVTILIKGPNKHTLAQIKDAVRDGLRAIKNA IDDGCVVPGAGAFEIAAYRELAQYKEEVKGKMRLGVQAYAEALLVIPKTL AVNSGFDTQEVIVKLQEEARIAGEPVGLDINSGEALKPIDHGIVDNYIVKK QILNSCTVIASNLLLVDEIMRAGMSSLKG
LMI_GLEAN_101 10738	COG0459	T-complex protein subunit epsilon [Locusta migratoria]	MASFPGTLAFDEFGRPFIIIRDQQNQQRLTGVEALKSHMLAGKTIANILRTS LGPKGLDKMMVSADGDVTVTNDGATILKQMDVDHEIAKLMVQLSQSQD

			DEIGDGTTGVVVLAGALLEQAEQLLDKGIHPIRIADGFELAAQHAVKHLDS IADSFPFDINNLEPLIQTAMTTLGSKIVNKCHRQMAEIAVNAVLAVADMEK KDVNFELIKVIGKVGGRLEDTMLVKGVVVDKDFSHPQMPKVLKDVKLAI LTCPFEPPKPKTKHKLDVTSVEDYRALRAYEAEKFTEMVQQVKDTGATLA ICQWGFDDEANHLLLQRELPAVRWVGGPEIELIAIATGGRIVPRFEELTPDK LGHAGLVREISFGTTKDRMLVIEECKNSRAVTIFIRGGNKMIIEEAKRSIHD ALCVVRNLVVDNRIVYGGGAAEISCALSVSTEADKYSSLEQYAFRAFAEA LESVPLALAENSGLSPIHTVTEVKARQAVEGNSALGIDCMLNGTADMRQQ HVIETLRSKKQQIVLATQLVKMILKIDDIRCPNDQGSHGL
LMI_GLEAN_101 85190	COG0459	T-complex protein subunit eta [Locusta migratoria]	MDKLIVDKNGKSTISNDGATIMKLLDVVHPAAKTLVDIAKSQDAEVGDGT TSVVLLAGEFLKQIKPFVEEGVHPRIIIKAFRKAIQLAVDKINSLAVKIDKKN VAEHRSLLEKCAATALSSKLISQQKGFFSKMVVDAVLLLDDLLPLNMIGIK KVTGGALEDSLLVAGVAFKKTFSYAGFEMQPKKYSNPKIALLNIELELKA ERDNAEIRVDNVQEYQKVVDAEWKILYEKLDLIHKSGAKVVLSKLPIGDV ATQYFADRDMFCAGRVPEEDLKRTMKACGGSVMTTAHDLKDSVLGSCE HFEERQIGGERFNIFTGCPNAKTCTIVLRGGAEHDAIMIVRRTIKNDAVVA GGGAIEMELSRALRDYSRTIAGKEQLLIGAIAKALEVIPRQLCDNAGFDAT NILNKLRQKHAQGNCWFGVDINNEDISDNFEACVWEPAVVKINALTAACE AACLVLSVDETIKNPKSGGGDAPAGRGMGRPIGGAELLFDKVKKHTLELP STDKTWTVRKLLVWIKENLLKERQELFLQGETVRPGILVLVNDADWELLV IYNLLNYQGY
LMI_GLEAN_100 45573	COG0459	T-complex protein subunit gamma [Locusta migratoria]	MFGAGAAPILVLSQNTKRDAGRKVQVENIGAGKAIADVIRTCLGPQAMLK MLMDPMGGIVMTNDGNAILREITVQHPAGKSMIEIARTQDEEVGDGTTSV IVLAGEMLGVAEQFLEQQMHPTIIIRAYRQALEDAVSILQERISIPVDTSDR QKMLDVIKACVGTKFIGRWSDLACSIALQAVETITLEENGRREVDIKRYAK VEKIPGGSIEDSLVLRGVMVNKDVTHPKMRRYIKNPRIVLLDCGLEYKKG

			ESQTSVEIMKETDFTRILQLEEEYVQKFCNDIIAVKPDLIFTEKGISDLAQHYLLKAGISAIRRVRKTDNNRIARACGATIVNRTDELKEEDVGTKAGLFEIKKFGDEYFCFITECEDPKACTILLRGPSKDILNELERNLQDALHVARNILIEPKLVPGGGAVEMALSQALTEKAKSVQGVGQWPYQAVAQALEVIPRTLAQNCGANTIRTLTALRAKHATGGSTWGINGDTGELADMHTLGIWEPLSVKLQVYKTAVETAILLLRIDDIVSGSKKKDRDSEVKKTAEPTEESMKE
LMI_GLEAN_10141757	COG0459	T-complex protein subunit beta [Locusta migratoria] Sequence ID: gb|AHB33469.1|	MDLGMDFFKGKEVVHQKVSLNPQRIFKHEAEEEKAETARMSSFIGAITIGDLVKSTLGPKGMDKILVSMGRNEGQVEITNDGATILKAVGVDNPAAKILVDMSRVQDDEVGDGTTSVTVLAAELLREAEKLIDQKIHPQIIIAGWRKALQVAVDALSAAAMDNSGDEAKFHQDLYNIASTTLSSKILSQHKDHFAKLAVTAVLRLKGSGNLSAIQIIKKTGGCLEDSFLDEGFLLDKKVGLHQPKRVENARILIANTPMDTDKIKVFGSRVRVDSMAKVAEMELAEKEKMKDKVAKILKHNCNVFINRQLIYNYPEQLFADAGVMAIEHADFDGIERLALVTGGEIVSTFDNPELVKLGHCDVIEQVMIGEDTLLHFGGVALGEACSIVIRGATQQILDEADRSLXCVLAATVKESRTVYGGGCSEMLMAGEVMAAASKTPGKEAVAMEAFARALQTLPTAIADNAGYDSAQLISELRAAHSQGSHTMGLDMENGRIGCMKTLGITESFVVKRQVVLSAAEAAEMILRVDNIIKAAPRKRVQDRGRC
LMI_GLEAN_10157839	COG0459	T-complex protein subunit theta [Locusta migratoria] Sequence ID: gb|AHB33466.1|	MALHVPKAPGMAQMLKDGARYFSGLEEAVYRNINACKQFSQTVRTAYGPNGMNKMVINHIEKLFVTNDAATIIRELEVEHPAAKLMILASQMQEQEVGDGTNFVIILSGALLEAAEDLLRMGLTPSEIVDGYEVALEKALEILPTLTCYEIKDFRKEEDTARGIKPAIMSKQYGNEDFLTKLITKACVSNVPEKTTFNVDNIRVCKILGSGLQNSDVVQGMVFKRTVDSDVLKKSPAKVAVFTCAVDIMQTETKGTVLIKSADELMQFSRGEENLLENQIKAIADAGADVIVSGGKFGDMALHYINKYNMMAVKLSSKWDIRRVCKTVGATALPRLTAPTKEELGYADNVYVDELGDTSVVVFKLEGRESRIATIVIRGSTDNYMDDIERAVDDGVNTFKGITRDGRFVPGAGATEIELARQIASFAETRPGLEQYAIKKFATALETFVKCLA

			ENSGVKSNEVVSKLYAAHEEGKKNFGFDIEGEGAAVIDVAEAGIIDLYLTKMWGLKYATNAACTILKVDQIIMAKRAGGPKAPPAGAQNDED
LMI_GLEAN_10185305	COG0459	T-complex protein subunit delta [Locusta migratoria]	MTAQTGAGDSRKAQAGAYKDKRKPTEVRESNINAAKAVADAIRTSLGPRGMDKMIQASNGEVTITNDGATILKQMNVTHPAAKLLVELSKAQDIEAGDGTTSVVVVAGSLLEAAQKLLTRGIHPTVVSDAFQRAAAKSVEILSGMSQPVSLADRDSLIRSAATALNSKVVSQQSSLLAPIAVDAVLKVIDPAQDTNVDLKDIRIIKKLGGTVEDSELVDGMIFTQKTVNVNGPKRVEKARIGLIQFCISPPKTDMDHNVIVSDYAAMDRVLKEERAYILNIVKQIKKAGCNVLLVQKSILRDAISDLAVHFLDKIKVMVIKDIEREDIEFVSKTLGLRPIASLDHFVPEHLGSAELVEEVAAGATGKFVRVSGIHNAGRTVTVLVRGSNKLVLDEADRSLHDALCVVRCLVKKRALIAGGGAPEIELALRLAQYSQTITGVDAYCFRAFADALEVIPSTLAENAGLNPIATVTELRNRHAQGEMYAGINVRKGAITNILEENVVQPLLVSISAITLASETVRSILKIDDIINTIH
**ubiquitin-dependent protein catabolic process**			
LMI_GLEAN_10130126	COG5647	ubiquitin-dependent protein catabolic process; Biological Process GO:0031461; cullin-RING ubiquitin ligase complex; Cellular Component GO:0031625; ubiquitin protein ligase binding; Molecular Function IPR001373; Cullin, N-terminal IPR016158; Cullin homology IPR016159; Cullin repeat-like-containing domain IPR019559; Cullin protein,	MCYIAWQRRPNDIYRSGSDCVRGTRDMSLKPKNVDFNSTWSALRETVKGVLTLSSVPRSVWNDRFSDVYSLCVAHPEPLADRLYLETKAFLDSHVRSLLEQVRANGENNLLRAYYQAWSQYSLGCLYLHKLYLYLNQQHIRKQKLSEAEIIYGNLSAALQEQMEVGELGLEIWKKNMIQPLKDSLVSLLLEGIHQDRIGEGNPNSTDIIRGVIQSFVSVEEFKMKGNLCLYEEIFETPFLAASGEYYKLEASRLLQTCDVSLYMERVTLQLKEETLRSQKFLHCSSFPKVRAKCEHHMVEEHLPFLHSECKAMVQQERRRDLSLMYPLLRSVQGGVAVLIKEVLEHIKQQGLEAITGLRGDNVHTQFVENMLAVHKKYKELIADVFAGDQSFMGALDKACSYVINYKSNPKIMCRSPELLAKYCDSLLRKSAKGMSDTEIDDRLAQSITIFKYIDDKDVFQKFYSRMLAKRLIHQQSQSMDAEEAMINRLKQACGYEFTNKLHRMFTDISVSSDFTNKFNVFLKSTYGCDLGFNFSMYVLQAGAWPLTQTTITSFSVPKELEKAVQKFETFYHKGFSGRKLTWLHHLSQGELKLGYLKKPYQV

		neddylation domain similar to CG1512-PB, isoform B; K03870 cullin 2 CUL2_MOUSE Cullin-2 OS=Mus musculus GN=Cul2 PE=1 SV=2 Q174D9_AEDAE Cullin OS=Aedes aegypti GN=AAEL006929 PE=3 SV=1	TMQTFQMAILLQFQEVDALTCREVQECLQLFDDPFNKHILGLIESKLLIAEN QTLGPDTLLRLNKEYTNKRTKFRITTAAHKESPQEIEQTISAVDEDRKLYL QAAIVRIMKSRKVLKHNCLIQEVLRQSKASFAPTINLIKKCIEALIDKQYIER TPHSPDEYSYVA
LMI_GLEAN_10 121674	COG5113	ubiquitin ligase complex; Cellular Component GO:0004842; ubiquitin-protein ligase activity; Molecular Function GO:0006511; ubiquitin-dependent protein catabolic process; Biological Process GO:0016567; protein ubiquitination; Biological Process GO:0034450; ubiquitin-ubiquitin ligase activity; Molecular Function IPR003613; U box domain IPR019474; Ubiquitin conjugation factor E4, core similar to ubiquitination factor E4B, UFD2 homolog; K10597	MSELSQEEIRKRRLARLAVLDTKASSSSSPPQSAQLAPPSPSKAVPSTPPTPG PSQSGSEPGANQGTSCSSPSKSSCAEPMTPPCLTTQKSDSQSTDTSSQMEVD EMPSCEKSASASQMDVDSGIENMEVEDSREGTGLRHRTSSSCNDITEDQV HATVSRVLCVSWKEKSDKAIYLPETAAAIEETDSINVQDLVNQCIMEVLVI VCQRGEDPESELSGVSTTEGDRDGDLSSPAPHAASSSDSPYPPSSPLQQQER SSPALPLTDPQERALRYLTNCYSRVATEERNHPKRCSSPPLSEVLSDLRAQL VQFAALVLQGSLDYWPEERPTSATSGTIGGLGVSESLLLSPILAQTLPRGFL PELVSRTYTSPGPGDAFSKIFGSVLQGLFITMKVASIVGNGHRQPIQALSDL LEIRCGPSGNVRPICRLLTQQVQFLPDLVTPAVGRELARTSYLGPFLSVSVF AEDEPKVAEKFFSGNTAADKSLNHTLQIELENTRVIMHKVFHDILVNNSSR EPTLSYVATLLRTNEKRAQIHADERMLAGDGFMLNLLSVLQMLALKVKM DKVDVFYPFHPSSLIDVKNETRLKFTSQEATSWLDDLNRSSTHKWREPKFP TQCWFLTLHCHHLALLPTCHKYQRRIRALRDLQKLVDEMQATEPQWREM GTAARNKELLRRWKQQVKKLTRSKACADTGLLDEALVRRSLVFYSSVAE LLLSTLNDPAAPPGQLLPSSAPTVFSALPEWYVEDVAEYLLFALQFMPGVV ADCMENNLISWLLVAVCCPHFIRNPYLIAKIIEVLFVINPNVQSRTETLHDR VMAHPISQVHLPSCLMKFYTDVETTGSSSEFYDKFTIRYHISVILKGMWDS

		ubiquitin conjugation factor E4 B [EC:6.3.2.19] UBE4B_MOUSE Ubiquitin conjugation factor E4 B OS=Mus musculus GN=Ube4b PE=1 SV=2 Q6DID4_MOUSE Ubiquitination factor E4B, UFD2 homolog (S. cerevisiae) OS=Mus musculus GN=Ube4b PE=2 SV=1	PLHRQAIINESRSGKQFVKFVNMLMNDTTFLLDESLESLKRIHEVQELMSQ AEAWSQVPHDQQQARQRQLSADERQCRSYLTLAKETVDMFHYLTVAIKE PFLRPELVDRLAAMLNFNLQQLCGPKLPPHPTSSVSQDEVVILTSAKDLAL TGSVAPKVLDSVGSDHLAAGKNLKVRNPEKYGWEPRWLLSQLIDIYLHLN CDSFAAALAGDERSFKKELFEDAAARMERRSIKTPTEIEQFRSLAEKAHDIS IQNLKKEVDYSDAPDEFRDPLMDTLMEDPVLLPSGKVMDRAVIVRHLLNS STDPFSRQPLSEDMLEPGGMFCSKNLFVFPKAAELRERIASWKREKQKSA
LMI_GLEAN_10 106747	COG5647	ubiquitin-dependent protein catabolic process; Biological Process GO:0031461; cullin-RING ubiquitin ligase complex; Cellular Component GO:0031625; ubiquitin protein ligase binding; Molecular Function IPR001373; Cullin, N-terminal IPR013745; HbrB-like IPR016158; Cullin homology IPR016159; Cullin repeat-like-containing domain IPR019559; Cullin protein, neddylation domain NV18061; similar to cullin;	MSLRPTKVNFEETWAGLSTTVQAVLTLRPVARAEWNGRFSDVYSLCVAQ PEPLADRLYRELKELLQKHVSALRRKLAKLPDRELLQQYHQAWDYYNTG VSYLNQLFRYLNQQHIQKRRAVEPDLMYASDADLTQLECPLEVGELAME LWRREMIISTESRLIPQLLDAYAADRDGDSRSSDALAAAVRSLVAVEQFAP ASNRDSLHLYRTIFEKRLLEASAQVYRQRAAKLLQHGDASHYVKAVSAFL REETERARRLLHQTSVPRFRQRCEQHLISDHLQFLQEECQRATRAEDLVAL GSAYPLLRGVPAGAVVMREELEKLVKERGLNEVWPASGQAPAPADFVNN VLNILDRYRDLVMDAFLGDTSFTAALDRAVSRVVNHRLQPNTPCPAPEIL ARHCDTLLRRTGNTVDSELDEQLGRAVSVFKYLDDKDVFQKFYARMLAK RLIFQHSLSMEAEEAMIGRLKQCCGCEFTSKFHRMFLDVSVSSDLNRKFED AIPPAPGSASFSVQVLQAGAWPLNPAPISPFAPPEDLQKRLQSFESFYRHLF NGRKLTWLYHLCQGGASLGYLKKPYVVTMQAFQLALLLMFERSDKIVCR EACDALELNEDQFRRHCISLLESRIILLEGGKDVDSEGATFRLNTEFSNKRI KFRLGAPTQRDPPEETKQTLSSVDEDRKMYLQAAIVRVMKSRKVLRHTAL VQEVLSQSSAAAFAPSISLIKTCIEALIDKQYLERSPRAPDEYSYVA

		K03870 cullin 2<br>CUL2_MOUSE Cullin-2 OS=Mus musculus GN=Cul2 PE=1 SV=2<br>Q174D9_AEDAE Cullin OS=Aedes aegypti GN=AAEL006929 PE=3 SV=1	
LMI_GLEAN_10083806	COG5113	ubiquitin ligase complex; Cellular Component GO:0006511; ubiquitin-dependent protein catabolic process; Biological Process GO:0016567; protein ubiquitination; Biological Process GO:0034450; ubiquitin-ubiquitin ligase activity; Molecular Function IPR019474; Ubiquitin conjugation factor E4, core ubiquitin conjugation factor E4 A, putative; K10596 ubiquitin conjugation factor E4 A [EC:6.3.2.19]<br>UBE4A_HUMAN Ubiquitin conjugation factor E4 A OS=Homo sapiens	KKTGIAKLSEDIFGFTVNKVIDEGKTLIFLEDLASEIAPKSSIDMEVLEQALFQRLLLEEPETYMLNKTVNRLERNDNHVTEKEVISYLFECYKRLCSPLIAKDVKGDEIHEIKNLILRNAATALRQPALFEAQELHLQMINLFRDTSTPPEDFINFFSGISDQFVKDESEPINALLEAFTTTLDFMQNDVAKSNIFTFNRCYLHILQTFSVNPELGAMLLLHSTPKETTPGRLYADTLFGSILSLSCLPRSPNGPYEFFQDPLQSPDIEGANIWPALSFICENMHKMFYNLLKSSTHVKHETLSWLANCLHSNSARGKLWTAHNLGLGAPACVSDGFMLNLGCVLLQLCQPICSSLNDLKVNRIDPTYCAVKVEDDAEAHFRGMHMKGLDSETCLIPIAENEQRPTAEKYSFVTECFFMAHRALDLGFRVSLERLMQLYRDVTQIQRVFMEVQEQGSFQSEVVQTIRQRLEREMTRCFSIRAALLEPHSLSLMVQFHVATAVWLVQVATDVIDEDERVSFMPVKLRDITFPLPEQVPSTLRADTEYCLPFTTEEVATAIKEIKPGRAPGFDGIHAEFLLHCGKYVKHWLAEFLTNILQTGNVPHEMKRTKIIALLKPGKPNDKPENYRPIALLNMLYKLLERMVYNRICYKIFEIILVEQAGFRPNRNCIDQVLQLAAEAESNMEAVSPPIFLRFINLLMNDAVFLLDEALSNMAQLRQMQAARQGTSMVFMILNSGEWRQLPSHEQEQNEALFQNTGMIARKTKKILSFVRMKLLKCHCAHLQTVFLSFPVLILECLYVYMYFLQHVPDYLLNLFGVLVKDQKEYEFDPAAIVMNICKIYVHLYNSDEFCTAVSSDGRSYSPQLFQLAEDVLARIGGGALITDLQLVANKVAMMASRQKTEEEILAEAPEEFLDPIMSTLMMDPVILPSSRKTVDRSTIARHLLSDQTDPFNRSPLTMDQVKSDEDL

		GN=UBE4A PE=1 SV=2 Q17E52_AEDAE Ubiquitination factor E4a OS=Aedes aegypti GN=AAEL003907 PE=4 SV=1	RLKIEAWVREKKE
LMI_GLEAN_10 191607	COG5148	Ubiquitin interacting motif similar to 26S proteasome non-ATPase regulatory subunit 4 (26S proteasome regulatory subunit S5A) (Multiubiquitin chain binding protein) (54 kDa subunit of mu particle) (p54); K03029 26S proteasome regulatory subunit N10 PSMD4_DROME 26S proteasome non-ATPase regulatory subunit 4 OS=Drosophila melanogaster GN=Pros54 PE=1 SV=2 B4KYX3_DROMO GI13465 OS=Drosophila mojavensis GN=GI13465 PE=4 SV=1	MSAGRIIYESGVWNAPLTAGVVDNSDYMRNGDFVPTRLQAQQDAVNLVCHSKIRSNPENNVGLLTLANVEVLATLTSDVGRILSKLHQVQPQGNIKFLTGIRIAHSSFGHMHYLTALWNVELLEEFLSTSGAAVALSAGSVRLALKHRQGKNHKMRIVAFVGSPVQAKGSDLIRLAKRLKKEKVNVDIVSFGEEVNPKVSNNELLTAFINALNGRDGGSSHLVTVPPGPHLSDALLSSPVIQVSNNELLTAFINALNGRDGGSSHLVTVPPGPHLSDALLSSPVIQGEDGMGGAGLGASGFEFGVDPNEDPELALALRVSMEEQRQRQEEEARRAQAASAPDGAPQPADTIKEEQSEEALLERALAMSLEGGAEEQSSQTGIPDFSKMTEEEQIALAMQMSMQDAQPEDVAMKEGSSSVKEEKTETPMEVEGDDDYSEVMNDPEFLQSVLENLPGVDPQSEAIRKAVGSLKDKKESDKKDRKDDDKGKK
LMI_GLEAN_10 140703	COG5201	ubiquitin-dependent protein catabolic process; Biological Process IPR001232; SKP1 component IPR011333;	MPNIKLQSSDGEVFEVDVEIAKCSVTIKTMLEDLGMDEDDEEVVPLPNVNSAILKKVIQWATYHKDDPPPPEDDENKEKRTDDISSWDADFLKVDQGTLFELILAANYLDIKGLLDVTCKTVANMIKGKTPEEIRKTFNIKNDFTASEEEQVRKENEWCEEK

		BTB/POZ fold IPR016072; SKP1 component, dimerisation IPR016073; SKP1 component, POZ S-phase kinase-associated protein 1A; K03094 S-phase kinase-associated protein 1 SKP1_XENLA S-phase kinase-associated protein 1 OS=Xenopus laevis GN=skp1 PE=1 SV=3 Q1HQ42_BOMMO S-phase kinase-associated protein OS=Bombyx mori PE=2 SV=1	
LMI_GLEAN_10027556	COG5078	Ubiquitin-conjugating enzyme E2 N [Zootermopsis nevadensis]	MTALPRRIIKETQRLMQEPVPGISAVPDDSNARYFHVIVTGPEDSPFEGGLFKLELFLPEDYPMSAPKVRFITKIYHPNIDRLGRICLDILKDKWSPALQIRTVLLSIQALLSAPNPDDPLANDVAELWKVNEAEAIRNAKEWTRRYAMDN
LMI_GLEAN_10042466	COG5078	Ubiquitin-conjugating enzyme E2 L3 [Zootermopsis nevadensis]	MAATRRLQKELGDIRSSGMKSFREIQVDDSNILTWQGLIVPDNAPYNKGAFRIEINFPAEYPFKPPKINFKTKIYHPNIDEKGQVCLPIISAENWKPATKTDQVIQALVALVNDPEPEHPLRADLAEEYLKDRKKFVKNAEEFTKKHSEKRPSD
LMI_GLEAN_10169729	COG5077	Ubiquitin carboxyl-terminal hydrolase 47 [Zootermopsis nevadensis]	MERLLRDGPHTPATTTDGRWSEHQETIDNLRAATARRGLQMERLTRDGPLTPATTTDGERTGRKRPLEHQESVNSSGAAQGGRGQRTERPRRDRPERATQALDRAPGKGQQLTSSASHGGWVKLSVILLYLITNHLCTGMVCMLEERNAICTVRDVTTLASQGSQKKITLSLPASTTVQELFQKLADAYQYDPNTFELILQRSSDPVVLNNHKEETIEEVGVNFEPGYNNNLIVTEMRGQQTRKMAVASGD

			GGEDEMMLGASASPTAVSDFSQTPPAPLPPPVVSGDYYGSVMIKHDTVEY VGLVNQAMTCYLNSLLQALYMTPEFRNALYKWEYDDSEKDEAKSIPYQL QKLFLNLQTSKKSAVETTELTRSFGWDSSDSWQQHDIQELCRVMFDALEQ KFKNTDQADLISRLYEGKMIDYVKCLECETEKSREDTFLDIPLPVRPFGTA VAYSSVEEALRAFVQYETLDGNNQYFCENCNKKCDAHKGLKFSKFPYLL TLHLKRFDFDYTTLHRIKLNDKVTFPEVLNLNSFILNEIKEENEGISDDLVV KCDDSSTTDSGSALDDESCQGTEAGLSSHDNEFQEDDEGIDMGSCANHHE NEKNRRHAHEKGPYVYELFSIMIHSGSASGGHYYAYIKDFKKNEWFCFND QIVTRITQDDITKTYGGGPSRGYYSGAYSSSTNAYMLMYRQIDKQRNSEA MTVEHFPPHIQKLLEKMQEREEFDRKRRERELEDYKLKFCAYHPINHKILE DKVYCTSKTTVRQATELAHKALSLQGLVVPLERCRLVNYDRTQETIECSF EDQEDKPICEVLDLRGGRRYDLLLQHRKEDEEFEIYQPGGVTFKVFLINMS AHSTEDVDGPYPVRGNLYQTVGEFKATAAKALNLNPQNMYILQDKSNNH PPYLTSDDATLQSEGLHNTNKIFVAVSSGGETEKPHVPSKLFEIIREILDTFE YVITLNVVVPEVDKEVLRKLAIPPLNLDMNHNDSNSTQEENKGNDDCVSG PTVVSSLPAGGMQSTTPLAGTAETAVRTPLVQAQPPIVPLPAARDNSPQPP LDPEDEGIGSTGHSDQSASEDSSLTDSDRTLVGDVPDECLAHISTPSNGSDQ QNVSSPEEGNTSNYNSYAKDQSGNWDADDSDTPGFMARASNYYFKNIQS ADSDNKRTLKFLVDKRMLLSTLKKNLESYVGVPQEYFKINRVRAGQQEQ ECSRLAESLKSYRDDEKLIIKLEGEFRGKVHLLCPNNTAEISKFLCEWIIGK GMTVGEAKKEILEEVKKKHDLDIPFDRCRLRKKSWKTPKKVYLNHQQFD DDDLKLLANWEMFLQELPGPEVVTSVNQLILFVRRWCPSTYELMPFEEIVL DSNNITDTEIKKKLAELSGIPEEXXXXXXXXXXXXXXXXXEISVLTIQKEL EWNVQSPQDSWHLNMLEDGEILFYRDKTETVKELTNEERKEIVNREGSRV NKPGSVPSYSPRKERALKIYVDSSPKKRDDVDELD
LMI_GLEAN_10	COG5533	Ubiquitin carboxyl-terminal	MSWQDVLESDKEYVKDDSITLEVKVQADAPHGVSWDSKKHTGYVGLKN

127963		hydrolase 7 [Zootermopsis nevadensis]	QGATCYMNSLLQTLYFTNQLRKAVYKMPTESDDSSKSVALALQRVFHELQFSDKPVGTKKLTKSFGWETLDSFMQHDVQEFLRVLLDKLESKMKGTCVEGTVPKLFEGKMVSFIKCKNIDYKSTRVETFYDIQLNIKGKKNIDESFRDYITTEVLDGDNKYDAGEHGLQDAEKGVIFSAFPPVLHLHLMRFQYDPITDCSVKFNDRFEFYEKISLEPYLQQSEPTRADYTLHAVLVHSGDNHGGHYVVFINPKGDGKWCKFDDDVVSRCTKTEAIDNNYGGHDDDMNMTVKHCTNAYMLVYIRDSELKNVLQEVTEDDIPQELVERLLEEKRMEQIRRKERNEAHLYMSVQVLLEDSFDGHQGNDLYDPERAQYRIFRVRKQTTLQELLEHLAESMKYPVDQIRPWPFGFRSNQTCRPTLIDVEADLHKPILEVAENQNPWNIFLEVVPPDSGLQTLPPFDKDTDVLLFFKLYDPKNKRIHYCGHHYMPVAAKVQELIPMLNERAGFPPDTELILYEEIKPNMVEKIENCNEPLEKVLEELMDGDIIVFQKDEREDSELPTCKDYFKDLFHRVEVTFCDKMIPNDPGFTIDLSQRMTYDQMARAVAERVGVDPYLLQFFKCQNYKDSPGNPLRCTYDGTLKELLVYCKPKTPKKIFYQQLSIRINELENKKQFKCIWVGPKLKEEKELILYPNKNGTVSDLLEEARKQVELSENGSGKLRIFEVNCNKIISGPKEDGPLETLNSSASKTFRIEEIPRDELHLADDEMLMPVAHFYKDIFSTFGIPFFLKVKHGEPFSKVKDRLLKKLGIQEKEFEKFKFALVVMGRPTFIEQPDYCINLQDFRCHPNQGNTAPMPWLGLEHVNKAPKRSRYNYLEKAIKIYN
LMI_GLEAN_10136532	COG5207	Ubiquitin carboxyl-terminal hydrolase 5 [Zootermopsis nevadensis] Sequence ID: gb|KDR23210.1|	MAAVDLTRFLPDIRIPANGDKIYKDECVFSFDTPETETGLYVCLNTFLGLGADFVEQHYRKTGNAVFLHLKRIKKEIAAEQQGDGPEKKITRLAIGVEGGFDPDAGKKKFECEESYSIVVLPSYDTIKWPNSDLPEKVQECVKAILAAESATKLAELEALTGTWDGEARIVSKHASNLLQLNNGKKIPPSGWKCEKCDLTQNLWLNLTDGAVLCGRKFFDGSGGNDHAVEHYRETNYPLAVKLGTITKEGKADVFSYDEDDMVEDPNLAQHLAHFGINIAHMEKTDKSMVELELDLNQRVGEWNALQEAGSKLQPLYGPGYTGLVNLGNSCYLNSVMQVVFSIPDFTKRFFDEAPKIFENAPSDPASDFVVQMSKLGVGLLSGKYSQPPSDSDAGDESQPG

			ISPHMFKNLISKGHPEFSTKHQQDAQEFFMHLVNTLERNSRHQKNPAECFKFSVEDRFQCTKSKKVKYTYRAEYSLPLGIPLDAAINKEEVTAYEAHRAEMEAKGQRVDPGQIVRPKIKLFSCLEAFTQPEIITGFYSTAVSEKTTARKTTRLASFPDYLLIHLKKFTLRDDWVPIKLDVAVEMPDTIDLAALRGGGPQPDEEPLPEAPATIEMPPLDENLISQLVDMGFPPEACKRAVYFTDGAGLDQATNWLMSHIGDSDFSDPFVPLGTSSGPNKGKSNFEPNEEFCTMIMSMGFTRSQAVKALQATDNNVERAADWIFTHQAELDAEEEAPATASSSEPEFRDGDSKYQLVAFISHMGTSSMVGHYVCHILRDGQWVIYNDNKVARSENPPKELGYLYLYKRL
LMI_GLEAN_10014972	COG5272	polyubiquitin [Caenorhabditis elegans]	MQIFVKTLTGKTITLEVEASDTIENVKAKIQDKEGIPPDQQRLIFAGKQLEDGRTLSDYNIQKESTLHLVLRLRGGMQIFVKTLTGKTITLDVEASDTIENVKAKIQDKEGIPPDQQRLIFAGKQLEDGRTLSDYNIQKESTLHLVLRLRGGMQIFVKTLTGKTITLEVEASDTIENVKAKIQDKEGIPPDQQRLIFAGKQLEDGRTLSDYNIQKESTLHLVLRLRGGMQIFVKTLTGKTITLEVEASDTIENVKAKIQDKEGIPPDQQRLIFAGKQLEDGRTLSDYNIQKESTLHLVLRLRGGMQIFVKTLTGKTITLEVEASDTIENVKAKIQDKEGIPPDQQRLIFAGKQLEDGRTLSDYNIQKESTLHLVLRLRGGMQIFVKTLTGKTITLEVEASDTIENVKAKIQDKEGIPPDQQRLIFAGKQLEDGRTLSDYNIQKESTLHLVLRLRGGMQIFVKTLTGKTITLEVEASDTIENVKAKIQDKEGIPPDQQRLIFAGKQLEDGRTLSDYNIQKESTLHLVLRLRGGMQIFVKTLTGKTITLEVEASDTIENVKAKIQDKEGIPPDQQRLIFAGKQLEDGRTLSDYNIQKESTLHLVLRLRGGMQIFVKTLTGKTITLEVEASDTIENVKAKIQDKEGIPPDQQRLIFAGKQLEDGRTLSDYNIQKESTLHLVLRLRGGY
LMI_GLEAN_10125866	COG5272	polyubiquitin-B precursor [Homo sapiens] Sequence ID:	MQIFVKTLTGKTITLEVEPSDTIENVKAKIQDKEGIPPDQQRLIFAGKQLEDGRTLSDYNIQKESTLHLVLRLRGGMQIFVKTLTGKTITLEVEPSDTIENVKA

		ref|NP_061828.1|	KIQDKEGIPPDQQRLIFAGKQLEDGRTLSDYNIQKESTLHLVLRLRGGMQIFVKTLTGKTITLEVEPSDTIENVKAKIQDKEGIPPDQQRLIFAGKQLEDGRTLSDYNIQKESTLHLVLRLRGGCSL
LMI_GLEAN_10083497	COG5272	protein binding; Molecular Function IPR000626; Ubiquitin IPR006636; Heat shock chaperonin-binding IPR019955; Ubiquitin supergroup IPR019956; Ubiquitin subgroup NV18924; similar to ENSANGP00000013457; K04523 ubiquilin UBQL1_MOUSE Ubiquilin-1 OS=Mus musculus GN=Ubqln1 PE=1 SV=1 Q9VWD9_DROME CG14224 OS=Drosophila melanogaster GN=Ubqn PE=2 SV=1	MAEGQDPPKKINLTVKTPKDKQTVEVESDASIKDFKVLVAKKFNAEPEQLCLIFAGKIMKDHETLQTHNIKDGLTVHLVIKTTTRNASEQPGTAPTPPRTSGVWLLPAAYGVGLAGLTGLDLLGLGATNFMELQQRIURELRGNPDMMRQILDNPLVQWLMNDPENMRALITSNPUKQKLMERNPKISHMLNNLDLLRQTMELARNPSMLQELMRNHDRAISNLESIPGGYNALQRMYRDIQEPMLTAATUQFVRNPYSTIGSSSNNSDGNNPQHGQENRDPLQNPWSPATSETGSTGTRTGTSGTTPGGNGSRPAAGAGSGAASPGQSLMQQMTDNPQLIQSMLSAPYTLTMLQTFASDPSAAAAIIDTNPLFAENSDLQQQVRQMLPNIVQQLQNPEIHNLMLNPELNALLQIQQGVEQLRNIAPGFGNTSTNTSSATTTASSTPASTATATSSVPGDGQGANQDVSSQFMARMLSSMSTQAGGNQPPEERYRAQLEQLAAMGFANREANLQALIGTYGDINAAVERLLQSRQMSQS
LMI_GLEAN_10181959	COG5021	protein binding; Molecular Function IPR000449; Ubiquitin-associated/translation elongation factor EF1B, N-terminal IPR009060; UBA-like IPR010309; E3 ubiquitin ligase, domain of	MKIDRGRLKKSSSEVPSDCQALIDKLRTSNRKDLFYALKGIQSWNFGKCELYHWIDVLDVLDDVLEECAKRETSRSWSLRIDLPGMEWERQLLLRVLNFTTLLIEHSFSRHLYNSVEHLISLLTSCNMSIVLAVLNLLYMFSKRSNFITRLSADKKKALVARLNRLAESWCAYDEGVTLAQSCRNVYLGGRSTLHYEFYETPKGQQKGNLSKSAIHIENIESLGLSAADLMDNILKRHPYLNEEKQLLLFSRLRLYINFPTYETRLLCVQARLQATSVLAYTNAILDNAHTLLYNGFLEELVEVVQMTDSNLTDIRAASLRTLTSITHLDRHPHFTKKPSARLNMIIEVTTANSFHGF

		unknown function DUF908 IPR010314; E3 ubiquitin ligase, domain of unknown function DUF913 IPR015940; Ubiquitin-associated/translation elongation factor EF1B, N-terminal, eukaryote HUWE1; HECT, UBA and WWE domain containing 1; K10592 E3 ubiquitin-protein ligase HUWE1 [EC:6.3.2.19] HUWE1_HUMAN E3 ubiquitin-protein ligase HUWE1 OS=Homo sapiens GN=HUWE1 PE=1 SV=3 A2AFQ0_MOUSE HECT, UBA and WWE domain containing 1 OS=Mus musculus GN=Huwe1 PE=4 SV=1	LPTFVRNCVSTLLSDPQLNRPKCPFPLPLATALFSFLYHLASYDAGGEALVSSGMMECLLRVIHWHGRELEHITFVTRAVRVIDLITNIDMQSFQAHGGLSSFISRLEVEVNACRRDQPFVIKTSSLRACYQAQHLTSNVNQQSDQGCSTDNEQDVMEVDAEKIQYDIIKTCLPQRAALLKSMLNFLKKAIQDSSFSDSVRNVMAGSLPSSLRHIISNAEYYGASLFLLATDVVTVYVFQEPSFLSTLQDDGLTYVIMNALLVKDVPATREVLGSLPNIFSALCLNARGLEAFIQYQPFLRLFKILVSPTYLGAMKRRKSSDPATDTPCNLGNAMDELIRHQPSLKAPAITAVNKLLDELYDIHQDPGNSLWRGNVRAEVPQPSSARQGGSSSSDEEDEDDASTLSHAQQEDGAVAERMNVPLVDYIINVMKFIDSILTNNSTDDHCREFINQGGIPRLMKILEISYLPLDHPVTSAGQAVATVCKSILNLTHEGEIFRVGLENMETTLCRLSELQLTTVYANGSILLHELASQTNAEMAFNSIHDLRLLHGVNSVHGYVVMFLNLSRSSQNELRSLALAHWGSVRGLYVLSQLKSLYKALVWESTVLLALCSEEVPLDKDFAKEDLQICTLLKIHFQFLGERSSGNDKGMTTAMEALSTNDTEASQESCSEQRNCLKVPPNIIAKYIKPLLHASSRLGRALAELFGMLVKLSVGTPLKRRQRYTVLSRVLTPAAKDISESLAMLLVDCLSWTSLPKVCVPQFKLTYLICSVGFTSPLLFDEKKNPYYLMLYQFLRYGGHTAYFESFRKALTDNDNLPLKTAFEDPLLPEGTGEFLDAWLLLLEKMVNTKCILDSPYSHIYKGPADEGFDPAKFLFEVQKDAYQAVMLMWRKKPLKTYGTRMSETMFAILRHIIRGERIYQEMLNETIASRKKALLAKPPTPSSSEATTKSLSSTTLGVNLDHLKQLMDMGFTEDRCVVALKNTSGLEQATDYLLNHPSPLIQMGKAYATPPESGHLLLGTRYSSTLLQSLDRLLRGSSRKYREALDEFSLDVFGSCMMYIDWIPETTYYAMDLLTAAAKRIGPHFRDVTINTVIAQVETYAEKLFEECFEVYDKPSNVLSAFLESPTGSKLATRMHLLSLLLEELRTPCALLIVRRGIISSLVSLLELAEVVMRMFHATVAPKWLTHLLNLINAVERIKVSSERRDKMLQVTTNTWRWFDISTGKWNAYSAANNKIINDAFFRGDPRVKVSCGGRRRYVISFTDMVQTNEETGNSRPVTLSLKCRDKSDSKTPTKDNVDEDMDVECLDFSERRKPVNL

			QRYDTTRSRQIIRACVGLMKIPLDRDTLHSAMFACIRLTRNYELAKAFIEA<br>GGIQTLLSLTTGPFNAASLNMATILIRHAIEEYAVLRHTMEKVIRAQAQQN<br>DPTPYKEILFFMLQTSSCVCRAPDMYAEIASDILRIDINHLSKRNDDDGQRL<br>LVKCLPSKNTTATVKDDDSVKVVIDLLNALIEPNNSQDPEAKAGSSKVQE<br>KVKSKESANSKSSKSTEGSSSQAWKRPLLTKSAILKILADIAKSYSAFAKVI<br>SDHIYTAGQTDLVKEECSALAFILDNFLMSKEASDKECSTMARVLIVALAS<br>CNHATDVQGTVISEVKSALLRCVSWQECSEKHTRIQQLSALVSSIIYNSPGP<br>VDVPRLHYGSSMNHIVKIMIRKGLVTDMAKISHSLDLCSSSLPNTMNAVL<br>RTLEVVTRIINMPVASGNQKPRSRSIALTDEHAVEVQAAEEQVMREQAER<br>ARVERERAAREQGSNGTEGAVQNAQVEGLEQIARDAANAVAQDASERPN<br>TGNQENREERDRENVSTDATELDSDDSDDSEPSDTDVNHMDDDEEGYDE<br>DNLEFFESDEDLFRIPGLEREGDDLLMIQYADPDTAGSRTGPRTSWNSNTL<br>PLASGSSEDAPNGNDTAGDIPASHPLVMARRNLAASTLSTSRTHRAMRQR<br>HYQYLQMNTRSQNNAVILQRLLGPSAATAIELGIPISPVAITELQDSNIRVIT<br>SERSLGVISNPQSEEDQADTTPGFVFGPSLTATVSTIPTAMFWWYEECKLL<br>DSDSQFSAVIVICGNFKGVLMFQMNSLLSVAENPPQSVTVSEGNAQQSSQT<br>SSSTSSGEQNSSNASNQRSSVTSSDAARNEAQVVEEVMEVDGQHQNREGQ<br>HTRSQPETSVSEPSTVTQSNGSDSSASNTLSLSREEVTVTGLSVTHTTLSSRF<br>RRSPSGNLDSNNFLAPNAQSDNESNGEGGGESQSSRGTLPVRRRAHATTST<br>TACGSATSASSTTSTTPTTATTSAAVSSSSSSSVTSTNSFRLSTANGSDASQA<br>NVSSVPSTSTAAVVHSAPSVSSPIPGTSSSSLQPIASASTSNAGTSDSAGGNP<br>SELLEGVDPSFLAALPDDMREEVIAEQLRLQRLRQRTLQQTRDLIASNVSE<br>VNPEFLAALPPSIQEEVLAQHHLEQQRQAAIASNPNDPVNAAEFFQNLPSS<br>LRQTILTDMEESQMSVLPPDLAQEAQTLRREWEARNRQLMQDRFFSHVSH<br>NTSALSSMLRNPGRVSSSSRYTIQASAAQQRAQWNAWNAASTDSSGSTGS<br>NYARVGARALLDSESVACLLVLLFIDESALNMGRLHRVLRNLCYHPPTRD

			WILKALLSILEKCSFAHKNSLPEQKRMTTRSQSQSSSPPTPNVITVHGAWLNTTFNYALGRVTEVFSLVKYPTKRGPTYITLQARACPVVFRHAVDVLISMAKGLPVHFLLCLKNSKGKPASSSSPAAGRTGKQEAADFWDVLLKLEAQCTKKGKSVARPNLNQNAEEEEFIPSIFEGSAFGKLFNLLKSTYVTQNKPLIDKLLRLLAYISVGLPEIVHTDMKSGELNPKDELLPISKPHMKLAVDVLTSRCCSEDGLEDITNLFLNLANCFYPAKFVILDLLNEGAMELGKMLKQHIGQVLKDLKATNKRLKRQHDDDTSPTDKLPKGTLMDRFTKHSVVINAPVKGKGRYDWSVTNVHLLTSKTSSQAFFLRTLKVIVQIRESIRAVHRRQKDMDERELVLPHLANLLPLDDLWEVLSSCLTELSNTPDSHAVLVLQPAVEAFFLVHTPPPVSEEKKAAPKGQEGNQAEQAGASTSGAAQQSVAPVSPLPPDSEAIANPQSSPSSSRVDWDSLFSVSVPRPPTKPKFLVFAETHRVVLNEILRQSTINIADGPFAVLVDHTRILDFDVKRRYFRSELERMDEGIRREELAVHVRRNHVFEDSFRELHRRSADEWKNRFYIVFEGEEGQDAGGLLREWYVIISREIFNPMYALFTTSPGDRVTYMINSSSHCNPNHLCYFKFVGRVIAKAIYDNKLLECYFTRSFYKHILGIPVKYTDMESEDYTFYQGLVYLMENHISSLGYDLTFSAEVQEFGVTDVRDLIPHGRYIQVCEENKMDYIRLVCQMKMTGAIRKQIYAFLEGFYDIIPKRLISIFNEQELELLISGMPNVDIEDLKANTEYHKYSPTSLQIQWFWRALRDFDQANRAKFLQFVTGTSKVPLQGFAALEGMNGVQKFQIHRDDRSTDRLPSAHTCFNQLDLPVYETYDKLRTYLLKAIHECSEGFGFA
LMI_GLEAN_10109339	COG5227	protein binding; Molecular Function IPR000626; Ubiquitin IPR019955; Ubiquitin supergroup IPR022617; Small ubiquitin-related modifier, SUMO similar to SUMO	MSENQEQKPDAGPGDGNSEYIKLKVVGNDSNEIHFRVKMTTQMGKLKKSYSERVGVPVTSLRFLFDGRRINDDETPKQLEMENDDVIEVYQEQTGGMY

		(ubiquitin-related) homolog family member (smo-1); K12160 small ubiquitin-related modifier SUMO_CAEEL Small ubiquitin-related modifier OS=Caenorhabditis elegans GN=smo-1 PE=1 SV=1 A5JM38_ARTSF SUMO-1-like protein OS=Artemia sanfranciscana PE=4 SV=1	
**Proteasome-related proteins**			
LMI_GLEAN_10195955	COG0638	proteasome subunit beta 7 [Coptotermes formosanus]	MASVVCPDISRPGFQFDNCRRNAFLLKEGFVAPKATKTGTTIVGIIYKDGVILGADTRATEDTIVSDKNCSKIHFLAPNMYCCGAGTAADTEMTTQMISSQLELHRLNTGRVVPVVTANRMLKQMLFRYQGYIGAALVLGGVDNTGPHLFCIYPHGSSDKLPYATMGSGSLAAMSVFESRWKPDMTEDEGKQLVRDAIAAGIFNDLGSGSNVDLCVIRKGSVDYLRPYDEANIKGKRQGTYDFKKGTTAVLSAASFPIEIEDISVNVIDTGESMDTSS
LMI_GLEAN_10028481	COG0638	Proteasome subunit alpha type-1 [Zootermopsis nevadensis]	FRNQYDSDVTVWSPQGRLHQVEYAMEAVKLGSATVGLKNRTHAVLIALKRASSELSAHQKKIIPIDSHVGISIAGLTADARILSRYMRNECLNHKYSHDALLAISRLIANIGNKMQVCTQRYDRRPYGVGLLVAGYDDQGPHIYQTCPSANFFDCKAMAIGSRSQSARTYLEKHLDEFLDCDNDELIKHGLRALRDTLPNEVELNNKNVSIALVGKGTDFTVFNEDETGRYLAMIEGEERRTGQPPPEEQPAPSGD
LMI_GLEAN_10	COG0638	Proteasome subunit alpha	MFLTRSEYDRGVNTFSPEGRLFQVEYAIEAIKLGSTAIGICTSEGVVLAVEK

046816		type-5 [Zootermopsis nevadensis] Sequence ID: gb|KDR23179.1|	RITSPLMEPTTIEKIVEVDKHIGCAVSGLMADSRTMLDRARIECQNHWFIY NEKMSVESVAQAVSNLAIQFGDSDDDGGAMSRPFGVAILFAGCDEKGPQL FHMDPSGTFVQFEAKAIGSGSEGAQQNLQEVYHKSMTLKEALNAALTILK QVMEEKLSSTNVEVMTMTPDKLFHMFTKEEVEEVIKDIA
LMI_GLEAN_10 147795	COG0638	Proteasome subunit beta type-6 [Zootermopsis nevadensis] Sequence ID: gb|KDR18713.1|	MASALTMKYPVESIRAGPANEDWMNAEHMTGIDVTLDYLDANLKVTNR PVSSDRINRFRCALAYQLGLLTSIMAVEYDGGVVIGADSRTTTGTYIANRV TDKLTRITDTIYCCRSGSAADTQNIADIVAYHLDFMQVETGEPPQVKTAAN VFRELCYNYRDSLVAGVIVAGWDKRRGGQVYSVPIGGMCVRMPVTIGGS GSTYVYGYVDANFKTGMSRQECLQFVTNTLTLAMARDGSSGGVVRLAA VGESGVERHTVLAGELPRFYED
LMI_GLEAN_10 002265	COG0638	Proteasome subunit alpha type-4 [Zootermopsis nevadensis] Sequence ID: gb|KDR06556.1|	MEAISHAGTCLGILANDGILLAAERRNTNKLLDEVFFSEKIYKLNDDMVCS VAGITSDANVLTNELRMIAQRYLIQYGESIPCEQLVSWLCDVKQAYTQYG GKRPFGVSILYMGWDKHYGYQLYQSDPSGNYGGWKATCIGNNSAVSAD NP
LMI_GLEAN_10 177216	COG0638	proteasome 25kD subunit, partial [Papilio xuthus] Sequence ID: dbj|BAM19378.1|	MASERYSFSLTTFSPSGKLVQIEYALAAVAGGGPSVGIKASNGVVLATENK HKSILYEEHSVNKVELITKHIGMVYSGMGPDYRLLVKRARKMAQQYQLIY QEPIPTAQLVQRVAMVMQEYTQSGGVRPFGVSLLICGWDENRPYLFQCDP SGAYFAWKATAMGKNFINGKTFLEKRYHPLYLQSKFGCRSRVWSEVPAS DEATPKIAVRFEHVVEPKREVHPRFWGKNKLQGEAHDLELELNMSSLIGL ADFIEDEIIPEAVPMEVFDTVTLSGGVPNVEIMTQFVHALKNGHSCYVESM GKEKKREQEEKAQCQKTAEEIKLIKMKKYSEDLELDDAVHTAILTLKEGF EGQMTADNIEVGICDAEGFRRLDPSTIKDYLANIP
LMI_GLEAN_10 187953	COG1222	26S protease regulatory subunit 7 [Zootermopsis nevadensis] Sequence ID: gb|KDR20131.1|	MPDFLGDDMRKVKKEEKEEEKEIKALDEGDIALLKTYGQGQYTKTIKAVE DDIQTIIKRVNELTGIKESDTGLAAPALWDLAADKQTLQNEQPLQVARCTK IINADSDDPKYIINVKQFAKFVVDLADSVAPTDIEEGMRVGVDRNKYQIHIP LPPKIDPTVTMMQVEEKPDVTYSDVGGCKEQIEKLREVVETPLLHPEKFVN

			LGIEPPKGVLLFGPPGTGKTLCARAVANRTDACFIRVIGSELVQKYVGEGA RMVRELFEMARSKKACLIFFDEIDAIGGARFDDGAGGDNEVQRTMLELIN QLDGFDPRGNIKVLMATNRPDTLDPALMRPGRLDRKVEFGLPDLEGRTHI FKIHARSMSVERDIRFELLARLCPNSTGAEIRSVCTEAGMFAIRARRKVATE KDFLEAVNKVIKSYAKFSATPRYMTYN
LMI_GLEAN_10 135648	COG5110	PREDICTED: 26S proteasome non-ATPase regulatory subunit 2 [Tribolium castaneum]	MPDDVTMTEGTKEEQEKKTEEETAELSEEDKQLQEELNLLVDKLVDDDE TQYLPALESLRSQIQASTTSMTSVPKPLKFMRQHFEAMKTVYDKIKDQKT KEFCADVISVLAMTVTGDKTECLKYRLLGSVCEIGDWGHEYVRHLAGEIA KEWSDVNSEDTGLKQKLIGLAHQIVRYHMGHNAEAEACDLLMEIEQLDM LEQYVDESAYPRVCLYLSSCVPYVADPENTTLLQATLKLFRRFNQYPQAL RVAMQLNDHSLIEKIFTSCPDLSVQKQLAFMLGRQQVYLELNENMPEYDD LVEIMSNSHLNNHFLSLARELDIMEPKTPEDVYKSHLENNRPPFGGGQVDS ARQNLAASFVNGFVNAAFGRDKLLMEDGNKWLYKNKEHGMLSATASLG LVLLWDVDGGLTPIDKYLYSSEDYIKSGALLACGIVNCGVRNECDPALAL LSDYVLHSSNVMRLGAILGLGLAYAGSNREAVLTLLYPVFSDPRSSLEVLC MAALACGMISVGSCNAEVTETLMQLIMERPEVELTESYAKFIPLGLGLCHL GRQDSIEAIIAALEIIPEPFKSMAETMIEVCAYAGTGNVLKIQKLLHICSEHY ETPEKDDLALCKSRVYQIHCQCGETYIGQTMRTVKIAAMNIGDMRLQQPN KSVVTGIRPHTYELFHLELSWEKLEYHATEDKKEDKKEKDKDKDKDKDK DKDKDKDKDKEKEKEKEKKDDKKDEVDLSTRQAVAVLGIALIAMGEEIG AEMAFRTFGHLLRYCEPVIRRAVPLALGLISVSNPKLNILDTLSKFSHDSDA EVAHNSIFAMGLVGAGTNNARLSAMLRQLAQFHGKDPNNLFMVRIAQGL THLGKGTLTLSPYHSDRQLLSPVALAGLLATLVGFLDVKNIILGRSHYVLF TLAAAMQPRMLVTFDEELNPLPVAVRVGLAVDVVGQAGKPKTITGFQTH TTPVLLAYGERAELATEEYIPLTPIMEGFVILRKNPDFRS
LMI_GLEAN_10	COG1222	PREDICTED: 26S protease	MASTAVEDPVSWEGEEGLGEEVLRMSTDELVSRTRLLDNEIKIMKSEVMR

193255		regulatory subunit 6A-like [Megachile rotundata]	ISHELQAQNEKIKENTEKIKVNKTLPYLVSNVIELLDVDPQDQGEEDGAVV DLDSQRKGKCAVIKTSTRQTYFLPVIGLVDAERLKPGDLVGVNKDSYLILE TLPAEYDARVKAMEVDERPTEQYSDIGGLDKQIQELIEAVVLPMTHKERF ENLGIQPPKGVLLYGPPGTGKTLLARACAAQTKSTFLKLAGPQLVQMFIG DGAKLVRDAFALAKEKAPAIIFIDELDAIGTKRFDSEKAGDREVQRTMLEL LNQLDGFSSTADIKVIAATNRVDILDPALLRSGRLDRKIEFPHPNEEARARI MQIHSRKMNVSKDVNFEELSRSTDDFNGAQCKAVCVEAGMIALRRNALT VTHEDFMDAILEVQAKKKANLNYYA
LMI_GLEAN_10 039877	COG0638	Proteasome subunit alpha type-7-like [Zootermopsis nevadensis]	MGSRYDRAITVFSPDGHLLQVEYAQEAVRKGSTAVGVRGKDVVVLGVEK KSVAKLQEERTVRKICLLDDHVVMAFAGLTADARILINRAQIECQSHKLTV EDPVTLEYITRYIAGRSAKTVREFLEKYYTPEVVSTERGTVKLAIRALLEVV QSGRKNLEIAVMHQGKPMQVCVFLLSSSFIYFYVADY
LMI_GLEAN_10 132194	COG0638	Proteasome subunit beta type-1 [Zootermopsis nevadensis]	MKQTEENGSVVAVAGEDYAIIAADTRLSAGFSIYTREQSKLFRLTNTTVLG CTGCWCDTLTLTRILEARMQMYLHEHQKPMATPAVAQMLSTMLYYKRF FPYYVSNILAGLDTDGRGCVFSYDPIGHCERSNYRAGGSAGALLQPLLDN QIGYKNMENVPKEPVPAERALAIIKDGFISAAERDIYTGDSILINVITKDGVT VDKFKLRQD
LMI_GLEAN_10 176880	COG5116	26S proteasome non-ATPase regulatory subunit 1 [Zootermopsis nevadensis]	AGIISLLDEPMPELKVFALKKLDLIVDEFWPEISEAIEKIEILHEDRGFNQHE LAALVASKVYYHLGSFEDSLTYALGAGDLFDVNGHSEYVDTTIAKCIDYY TQQRTANPEDAKKIDHRLVAIVNRMFQRCLDDGQYRQALGLALETRRMD IFERAIKESDDVFGMLTYAFQVAMSLIQNRGFRNTVLRSLVDLYRNLATPD YVNMCQCLIFLDDPLAVAEILDKLSKDTEDSALMACQIAFDLYESATQQFL GRVLQALRATAPIPTATSDTLEKKTDTSETTTASAGEQEIKPERKLENLNED EKLHQLRIEKLSSILSGEVSIDLHLQFLIRSNHSDMLILKNTKDAIRVSICHT ATVIANAYMHSGTTSDQFLRDNLEWLARATNWAKMTATASLGVIHRGHE QEALALMQSYLPREVGPSSGYSEGGGLYALGLIHANHGARIIDYLLGQLK

			DAENEMVRHGGCLGLGLAAMGTNRQDVYEQLKFNLYQDDAITGEAAGI AMGMVMLGSKATQAIEDMVAYAQDTQHEKILRGLAVGIALTMYGRLEE ADPLAASLCQDKDPILRRSGMYTIAMAYCGTGNNQAIRRLLHVAVSDVN DDVRRAAVTGLGFLLFRTPEQCPSVVSLLAESYNPHVRYGAAMALGIACA GTGLKEAIALIDPMTNDPVNFVRQGALIASAMILIQHSEHTCPKVKDFRAL YAKVIVDKHEDVMAKFGAILAQGIIDAGGRNVTVSLQSRTGHTNMLAVV GVLVFTQYWYWFPLAHCLALAFSPTCLIALNAQLKMPKMEFRSNARPSLF AYPPPLEEKKREEREKVTTAVLSIAARARRRHPKDKKDDTKDKKDEKKD DKKDEKKKSGKDEEKKEAEPSFEMLSNPARVMKPQLKVLQLPEGCPYTPL KDLTIGGIIMVRQLRPGHEEELVEPVAAFGPKSDEDKEPEPPPEPFEY
LMI_GLEAN_10 132175	COG5159	26S proteasome non-ATPase regulatory subunit 11 [Zootermopsis nevadensis]	MAGAMLFERARAVSSTNREEGIDLLNKIVRDQEIAENDEDTIRVKEQGILQ LGELYKKEGKAKELADLIKATRPFLSHISKAKAAKLVRSLVDFFLDLEAGI GIEVQLCKECIEWAKEERRTFLRQSLEARLIALYFDTGMFSEALSLGSTLLK ELKKLDDKNLLVEVQLLESKTYHALSNLPKARAALTSARTTANAIYCPPK LQASLDLQSGILHAADEKDFKTAYSYFYEAFEGYDSVECPKALTALKYML LSKIMLNNPEDVHQIISGKLALKYAGKDIEAMKSVAQASHKRSLADFQHA LKTYKKELEDDVIVRAHLGTLYDNMLEQNLCRIIEPYSRVQVEYISQSIKLP MQQVEKKLSQMILDKKFHGILDQGEGVLIVFEDTPVDKTYETALETIHSM GKVVDTLYQKAKKLS
LMI_GLEAN_10 071246	COG5071	26S proteasome non-ATPase regulatory subunit 12 [Zootermopsis nevadensis]	MADEIATDAGRVVKMEVDCTAVCDEKIPECEKLAKEGKLSEALEALLALE KQTRTSSDMISTGRVLVAIVRLCFEAGQWNTLNEHIALLAKRRSQLKQAV TKMVQECCEYVEKTPNKEIKTKLIDTLRSVTEGKIYVEVERARLTHKLAK MKEEEGNIVEAANIIQELQVETYGSMEKREKVELILEQMRLCLAKKDYIRT QIISKKINTKFFDDEGTQDLKLKYYRLMIELDQHEGSYLATCKHYRAVLNT PSVQEDADKRQEVLKNVVLYLVLAPFDNEQSDLTHRVLQEKLLDEIPTYK ELLRLFTNPELIKWSGLCEIYEKELRQGSPQSKPTSVFTPDSEQGQKRWQD

			LKSRVVEHNIRVMAKYYTRISLKRMAELLDLPIEVCTVYLA
LMI_GLEAN_10147761	COG0638	Proteasome subunit alpha type-6 [Acromyrmex echinatior]	MARGSSAGFDRHITIFSPEGRLYQVEYAFKAISQGGLTSVGLKGVDTAVVATQKKVPDKLIDASTVTHIFKLSDSIGCVMTGMIADSKSQVQRARYEAANWRHVNGYEIPADALCRRIADISQVYTQNAEMRPLGCSMIVVAYDQEQGPCVFKTDPAGYYCGYRAVTVGAKQTEANSYLEKKLKKKLAYSADEAIQLAISCLSSVLAVDFKPSEIEVGVVSKDNPKFRILTEQEIDVHLTAIAERD
LMI_GLEAN_10024114	COG1222	26S protease regulatory subunit 4 [Zootermopsis nevadensis]	GQNQSSGSGSGGDKKDDKDKKKKYEPPIPTRVGKKKRRTKGPDAAMKLPQVTPHTRCRLKLLKLERIKDYLLMEEEFIRNQERLKPQEEKIEEERSKVDDLRGTPMSVGTLEEIIDDNHAIVSTSVGSEHYVSILSFVDKDQLEPGCSVLLNHKVHAVVGVLSDDTDPMVTVMKLEKAPQETYADIGGLDTQIQEIKESVELPLTHPEYYEEMGIKPPKGVILYGPPGTGKTLLAKAVANQTSATFLRVVGSELIQKYLGDGPKLVRELFRVAEEHAPSIVFIDEIDAVGTKRYDSNSGGEREIQRTMLELLNQLDGFDSRGDVKVVMATNRIETLDPALIRPGRIDRKIEFPLPDEKTKRRIFNIHTSRMTLAEDVNLTELIMAKDDLSGADIKAICTEAGLMALRERRMKVTNEDFRKSKENVLYRKKEGTPEGLYLGQNQSSGSGSGGDKKDDKDKKKKYEPPIPTRVGKKKRRTKGPDAAMKLPQVTPHTRCRLKLLKLERIKDYLLMEEEFIRNQERLKPQEEKIEEERSKVDDLRGTPMSVGTLEEIIDDNHAIVSTSVGSEHYVSILSFVDKDQLEPGCSVLLNHKVHAVVGVLSDDTDPMVTVMKLEKAPQETYADIGELSASLFLVAINGLATAVESSVSPSMVKTSASILVLKVYLLLQGAIHTLQPWALSHGFQSSATFVGVGHTELYLGDGPKLVRELFRVAEEHAPSIVFIDEIDAVGTKRYDSNSGGEREIQRTMLELLNQLDGFDSRGDVKVVMATNRIETLDPALIRPGRIDRKIEFPLPDEKTKRRIFNIHTSRMTLAEDVNLTELIMAKDDLSGADIKAICTEAGLMALRERRMKVTNEDFRKSKENVLYRKKEGTPEGLYL
LMI_GLEAN_10037484	COG0638	proteasome subunit alpha [Schistocerca gregaria]	YDLSASQFSPDGRVFQVEYAQKAVDNSGTVIGLRGKDGVVFAVEKLVTSKLYEPGANQRIFNIDKHVGMAVAGLISDARQIVETARTEAANYRAQYGDG

			IPLRHLVDRVAGYMHAYTLYSAVRPFGCSVVLSSYEPVDGPAMFMIDPSG VSYGGYYGCAVGKAKQAAKTEIEKLKLSSISIKDLVKDAAKIIYLVHDELK DKQFELELSWVGNVTNGLHERVPAPVFAEAEKAAKAAMEDDSDSDTID
**Peptidase activity-related proteins**			
LMI_GLEAN_10 193267	COG5640	TPA_exp: trypsin 1B [Locusta migratoria] Sequence ID: tpg|DAA64571.1|	MLRTGLVLLLAVALCSASTYPLVRPIPGSRPWRTRLDGRIVGGSAVSISQY PWQLYFTISSYMCGASIISSTWALSAAHCVEGYSVSQMQLRAGTSTRGSG GTVHNIATGYIHSSYSGNDYDIAVVQVSNAFSFGTNVQAVGLASSEPGAG TSVTVTGWGTTSSGGSVSNTLMGVTVQIVSRSTCNQAYGGITARMICAGV NGGGKDSCQGDSGGPLVSGSTQVGIVSFGNGCGLAGYPGVYSNVANLRS WISQATGV
LMI_GLEAN_10 120545	COG5640	TPA_exp: chymotrypsin 1 [Locusta migratoria]	MMQRAALLVFLLASSALAKPTPARQWIRPNGRIIGGTTASIANYPWQLSFQ YSGSHICGASIISSDWVLTAVSGSLLGTNAQTVSLPSSGYDPAGGLAVTVT GWGVTSTNGNLPTNLMKVDTSIVARSTCQSIFSGINTVTARMVCAGAAGK SVCNGDSGGPLVSGTTQVGIVSWGNSRCESSPGVFSNVGNLRSWIQQATGI
LMI_GLEAN_10 193265	COG5640	TPA_exp: trypsin 1B [Locusta migratoria]	MLRTGLVLLLAVALCGASTYPLVRPIPGSRPWRTRLDGRIVGGSAVSISQY PWQLSFTISSYMCGASIISSTWALSAAHCVEGYSVSQMQLRAGTSTRGSGG TVHNIATGYIHSSYSGNDYDIAVVQVSNAFSFGTNVQAVGLAPSEPGAGTS VTVTGWGTTSSGGSVSNTLLGVTVQIIDRSTCNQAYGSITSRMICAGVSGG GKDACQGDSGGPLLSGSTQVGIVSWGNGSGYPGVYSNVANLRSWISQAT GV
LMI_GLEAN_10 157934	COG5640	TPA_exp: trypsin 1C [Locusta migratoria]	IGGYMCGASIISSTWALSAAHCVSGFSTSQMLLRAGTSTRGSGGTTHNIAT GYMHSGYSGKDYDIAVVQVSNAFSFGTNVQAVGLASSEPGAGTSVTVTG WGTTSSGGSASNTLLGVTVQIIDRNTCNQAYGSITARMICAGVSGGGKDA CQGDSGGPLVSGSTQVGVVSWGEGCALAGYPGVYANVANLRSWIQQAS GV
LMI_GLEAN_10	COG5640	TPA_exp: trypsin 2A [Locusta	MLKAALVCLLVVACSAAPSVQRPKPRPRLDGRIVGGQAVDISEYPWQLSM

191920		migratoria]	QEFGSHLCGASIISSEWALTAAHCLEGSYINYVTLRAGSSTRGSGGTVYDVELAYYHGYYDSYTTDYDIGVMQISGSFSFDTNVQAVTLATSEPSAGTSVTITGWGALSSGGSSPTQLQAVTTSVVARSTCNSAYGGEITDRMICAGETGKDSCQGDSGGPLVSGSTQYGIWGYGCAEEGYPGVYSNVAALRDWVTEAAGV
LMI_GLEAN_10078682	COG5640	TPA_exp: chymotrypsin 6, partial [Locusta migratoria]	SLVADYVKPIKLPSAGQTFAGSDATVSGWGLTSNDAWQIAGTLQYANVKIWDNADCQAIYGDLVDSNAICAMGTNGQHSCNGDSGGPLVVKDSAGESMVVGVVSWGPAKDCENPSQPDVYMRVTAFLDWIPECWHLSRAADIQRAIADNASTSSLAQVIAVSVAEYIQKLRLPASSQADPTYEGEVVTVSGWGITADDSTVVSITLQYVEMVVISNSECSESFPGAIDNDVICALGNPDGSPCSGDSGGALVYKASDGNWTQLGIVSFGSDTGCENGYPEVFTKVASYLDWISQNAGI
LMI_GLEAN_10152794	COG5640	TPA_exp: chymotrypsin 9 [Locusta migratoria]	MELTRRSWLIVTLEINHSQRKFQLSPLIEEQSRYIAKRRLSVRDKGTSGSCDSLLDLPRSQALETRCCGAKLPLRRVVPTGPARSLSGAAGRIYGGQDAYSGQFPYQVSLQLSLLIIRAHNCGGTIVSESTVVTAGHCVMGLGTYYAVAGELNLKKDEGTEQESKVSEQIAHPDYPGGLTVSANDIAVFKLSTPFTLGTYVQAIPLAAAGSLPTAGTSAIASGWGSTPTATTPDILQWLDATIIDWQSCRDLLDEAGIEDNPVVESMICTGPVTGGISLCSGDSGGPLVQNGELIGVTSWAITPCGAVGSPSGFTRASAFNDFVEQYI
LMI_GLEAN_10192577	COG5640	TPA_exp: chymotrypsin 3, partial [Locusta migratoria]	MVKPVPLLLFLLFAACGAAPRSGLRRPRSVHRGRIVGGEAVDISQFPWQVSLEHRNDHYCGGSIISKTWILTAAHCCDYGAPNYLLRAGTSTRESGGSVYDISKVIAHGDFDTYSGEYDIGAMSISGTFTFDDNIQAVELATDDPAVGTWVTISGWGLHAVTTQIVDTETCHETWTIITDDMLCTGGNSKGACHGDSGGALVANGTEYGIFSRTFCAMDGASDVYTSVAAFRSWIRRYTGV
LMI_GLEAN_10106452	COG5640	TPA_exp: chymotrypsin 10 [Locusta migratoria]	GEFPHQVSLQYVLLIIRYHSCGGSVISSNAVLTAAHCAISLGHYVAVAGDHDLGSDEGSEQEVRVSDQIVHPDYPGGAVVAANDIAVFLLESSLTLNDYVQAIALPSDGVVPEGGSTAVVSGWGTTETASTPDILQTVEVSVIDYDTCKQNL

			DDLGIGENPLTDTMVCTGPLYDGISTCSGDSGGPLVQNSELIGVVSWGITP CGSDGAPSVFTRVSAHLDFINQYI
LMI_GLEAN_10 103442	COG5640	TPA_exp: chymotrypsin 1 [Locusta migratoria]	RGGGRIVGGTSADIANYPWMVSVQNNGAHYCGGSLISSTWVLTACHCVY DNNVSLFSIRAGTSVRESGGVVTPSPSGLMNSYFNPNSLDYDISVLKVSNPI TIGGYIQLVGLPVQGYDPPGGLAVTITGWGSLGASGVSPTNLMKVDITTID RTSCVSRFGITDRMICAGEAGKSTCYGDSGGPLVSGSTQVGIVSWGRTNCE NDGGVYTNVGFFRTWITEGAEATVNRAKRKTQPWWRSMFRVLLAVLCV ASCALAAPHPQRARARGGGRIIGGSVADIASYPWMVSIQNNGAHFCGGSV ISSNWVLTAGHCLYGANIGTYSVRVGTSVRGSGGFVSGLSLGYLHGGYNP NTVDYDISVLQVSATYILSSTNRPQKYMCLPTLGYDPPGGLSVTITGWGNT VTTGTASTDLMKVDITVLDRNTCIGKFGITDRMICAGEAGKSTCYGDSGGP LVSGTTQVGIVSWGRQYCEDDGGVYSNVGNLRSFITETTGV
LMI_GLEAN_10 007070	COG5640	catalytic activity; Molecular Function GO:0004252; serine-type endopeptidase activity; Molecular Function GO:0006508; proteolysis; Biological Process IPR001254; Peptidase S1/S6, chymotrypsin/Hap IPR009003; Peptidase cysteine/serine, trypsin-like SP22; serine protease 22; K01312 trypsin [EC:3.4.21.4] TRY4_ANOGA Trypsin-4 OS=Anopheles gambiae	MWQFVSLGTSELEPGTSVTVTGWGYTEEGVVSNKLLAATVQIQDLKSCN ASYDRGITERMICAGVDKGGKDVCKGDSGGPLVQGSTLYGVVSWGKPCA LPGYPGVYASVPALRSWIQGATGV

		GN=TRYP4 PE=2 SV=2 B4JVV1_DROGR GH22923 OS=Drosophila grimshawi GN=GH22923 PE=3 SV=1	
LMI_GLEAN_10141490	COG0638	threonine-type endopeptidase activity; Molecular Function GO:0005839; proteasome core complex; Cellular Component GO:0051603; proteolysis involved in cellular protein catabolic process; Biological Process IPR001353; Proteasome, subunit alpha/beta similar to Proteasome subunit beta type 3 (Proteasome theta chain) (Proteasome chain 13) (Proteasome component C10-II); K02735 20S proteasome subunit beta 3 [EC:3.4.25.1] PSB3_ONCMY Proteasome subunit beta type-3 OS=Oncorhynchus mykiss GN=psmb3 PE=2 SV=1 Q1HPR0_BOMMO Proteasome subunit beta type	MSVLAYNGGAIIAMKGKDCVAIASDKRFGIQAQTVSTDFQKIFEMGPHLYMALPGLASDTQTVHQKLKFRLNLYELKENRNIHPKTFAAMVSNLLYEKRFGPFFVEPVIAGLDPKTYEPFVCNMDLIGCQNIPEDFVVGGTCTEQLYGMCEALWRPDLGPDELFEAISQALLNAVDRDAISGWGAVVYIIEKNKVTVKQLKTRMD

		OS=Bombyx mori PE=2 SV=1	
LMI_GLEAN_10020752	COG0638	endopeptidase activity; Molecular Function GO:0004298; threonine-type endopeptidase activity; Molecular Function GO:0005839; proteasome core complex; Cellular Component GO:0051603; proteolysis involved in cellular protein catabolic process; Biological Process IPR000243; Peptidase T1A, proteasome beta-subunit IPR001353; Proteasome, subunit alpha/beta similar to proteasome subunit beta type 5,8; K02737 20S proteasome subunit beta 5 [EC:3.4.25.1] PSB5_RAT Proteasome subunit beta type-5 OS=Rattus norvegicus GN=Psmb5 PE=1 SV=3 B6VAH6_HELAM Proteasome subunit beta type OS=Helicoverpa armigera PE=2 SV=1	MEDIESFTKDEHGKSMKIHFDHGTTTLGFMYKGGVVLAVDSRATGGQYIGSQTMKKIVEINDYLLGTLAGGAADCVYWDRVLAKQCRMYELRNRERISVAAASKLMANMVYNYKGMGLSMGMMICGWDKRGPGLYYVDNEGTRTKGKVFSVGSGSVYAFGVLDAGYNWDLSDEEAYDLGRRAIYHATHRDAYSGGIVRGKLLQMGLLTLCTILLVTPTLTLTNFKYKYL

LMI_GLEAN_10070727	COG4870	proteolysis; Biological Process GO:0008234; cysteine-type peptidase activity; Molecular Function IPR000668; Peptidase C1A, papain C-terminal IPR014882; Cathepsin C exclusion similar to cathepsin C; K01275 cathepsin C [EC:3.4.14.1] CATC_CANFA Dipeptidyl-peptidase 1 (Fragment) OS=Canis familiaris GN=CTSC PE=1 SV=1 C3S7J7_FENCH Cathepsin C OS=Fenneropenaeus chinensis PE=2 SV=1	MKDQLKQFCLAPVRMRSAQRFRRNSLDSDAVQTVTVDLEYPNVATDQYGNVGTWTMIYNQGFEVTVNGRSYFAFSYYTSRNGTVTSICDRTFNGWSHDVTERHWSCYYGVKNSPVAKKTHEDPFAAKPHLMAQVLDIMQEVRLVNEINNAQKLWKAKVYENFLGMTKEELTNMAGGKRMTSDGGGRVDVKEARCSCCSTWLAERPKPMPVTLEQRQRASYLPKQWDWRDVGGVNYVSPVRDQESCGSCFAFAAAAMMEARIRILTNNTQQPVFSPQDVVSCSPLDQGCSGGFSYLVAGRYGKDCSSMSIAEVLFRLGSSGNYLWNTEAELKFVTWREILDYSRRLKSVSLVGRNYANMRLLVLQDYGLVEEACNPYVGGDTECSTDTACTRYYSSDYEFIGGYYGGSNEVLMQEALLQNGPLTVDFMVYDDFRAYAGGVYKHVEATARSDFNPLVVSNCR
LMI_GLEAN_10154023	COG4826	serine-type endopeptidase inhibitor activity; Molecular Function IPR000215; Protease inhibitor I4, serpin similar to mCG117402; K13963 serpin B NEUS_HUMAN Neuroserpin OS=Homo sapiens GN=SERPINI1 PE=1 SV=1	FDAFDWALCRALDARYPDNVVVSPIGVKLVLAMLYEGATGDTARQLETGLLLTKDRSQTREKYSAIVASLQANNSDYLFDLGNKVYADLSLTLRPRFVTILRAFYNSDIENVDFRDPKTVPLINEWVKNATRGHIDSIMSEDGLSDAVLLLVNALYFKGSWKYQFQPQFSFPGNFYAGKGKSIAAQFMRQSAEFYYLHSKEINASILRLPYLGRKFAMFIVLPDDKDGLENLLSTVNPFALREDLGLLRPTAVHVVLPKFTFEFSVLLNDVLKELGIKQIFTDRANLQGIARSRYGRLSVSKVLQKSALEVNEQGTTAAAVTGKLIVQLIIEVTFNATHPFLFFIEDETTGTVIFVGKVVEPSTDKTPKTTISVRQGEFPDPNTIGLRGKFFNKIPNDDRFNYFDIELIQEATKRQDGNVLISPVSIKAALLMVLEGAVGKSAEEIRDVLRLPEEKDSYRI

		A0JCK3_PLUXY PxSerpin 3 OS=Plutella xylostella GN=pxSrp 3 PE=2 SV=1	KTQQFLRRLDVRNNLFISNDLKPYSEYRSAMQEYYSANISEVEFTSPTKAA TTINDWVSRVTHGLIPKLVEAEGLPADTKLMMTNAVYFKGKWKIAFDVD GTTVRCFYKQNLECQRSYFMETLSYFKYGYISALDAEAVEILYNDDQFSM VILLPTKRNNINKLIRDLTHSPLSDTIGKLQLTEVLVSIPRFNITYNSELIPVL EKLGVQEVFGAHANLSGIASDIGTAHISQVLHATKIEVNEEGTIAGAGTGV LVVPLMGTTIPRFRADSPFLFFIRDTVTGSILFGGRV
LMI_GLEAN_10 009014	COG4826	serine-type endopeptidase inhibitor activity; Molecular Function IPR000215; Protease inhibitor I4, serpin similar to CG9453-PB, isoform B; K13963 serpin B ILEU_HUMAN Leukocyte elastase inhibitor OS=Homo sapiens GN=SERPINB1 PE=1 SV=1 Q8IS52_CTEFE Serpin 6 OS=Ctenocephalides felis PE=2 SV=1	LDALTRLVLVNAIYFKGLWNIPFNKDATAPMPFHVSASNKKTVDMMKLV KKFMYTDAEQLEAQVLELPYKGDQLSMVILLPKKNDGLKELEAKLAGVN LPDILNQMRKVEVTVYLPKFKLEHSINLNESLQKLGMKTMFDECNADFTG INDSKPGLVVSKVLHKAFIEVNEEGTEAAAATGAVMCLRMARIPQEPIIFK ADHPFVFLIIDCKTKTSIFAGRI
LMI_GLEAN_10 089109	COG4826	serine-type endopeptidase inhibitor activity; Molecular Function IPR000215; Protease inhibitor I4, serpin similar to serine (or cysteine) proteinase inhibitor, clade B (ovalbumin), member 3A; K13963 serpin B	MIPGSAYWAVRPWEQNATVLPNSSLPDSTANCCCRVTELAKVHSAGRAH AQREALCKTAGSFLYSPFSLEVVLSLVHLGAKERSAQEISDTLHLPQDTKII LDGFGDLLHSIKCTDGLMLHTASKIYIHNKFTLLESFLSDTVRLLAEAELVD FESEPDKARTIANEWVQKKTDNKIKDLIAVGALDPKTRLFLINAVYFKGK WEHPFPDNNTFPMPFYTSSSQSKEIPMMQLERKFMYANLPELEAQVLELP YQGNQMSMMVILPNKTDGIAELENKINTVNLHQISDKLNSIKVNVLLPKFK IEKEIQLNEILKQLGMKEIFEEKLADLSGITGSSQLYVNHIFHKAFIEVNEEG TEAAAASDSKGRRLPNFLSDEEACTGQDSAKNCIKPVCGLKTTTTN

		ILEUC_MOUSE Leukocyte elastase inhibitor C OS=Mus musculus GN=Serpinb1c PE=2 SV=1<br>B4MJR9_DROWI GK20760 OS=Drosophila willistoni GN=GK20760 PE=3 SV=1	
LMI_GLEAN_10159173	COG5640	catalytic activity; Molecular Function GO:0004252; serine-type endopeptidase activity; Molecular Function GO:0006508; proteolysis; Biological Process IPR001254; Peptidase S1/S6, chymotrypsin/Hap IPR001314; Peptidase S1A, chymotrypsin-type IPR009003; Peptidase cysteine/serine, trypsin-like GA18102 gene product from transcript GA18102-RA; K01312 trypsin [EC:3.4.21.4]<br>STUB_DROME Serine proteinase stubble OS=Drosophila melanogaster GN=Sb PE=2 SV=2	MEWRLIINPAKVFVEMKLRRTLLREDGISVEDESFARFLVSVMVQKYENKLDTVLTLTHNDDNRDDNDEVGCRSGGRWRCGRWRRPRRRRRRXXXXXXXXAPSATQAPAAARADCECVPYYLCQNDSIITDGVGLIDIRSGLDTQSVRLRLSRSAEPPGIQDGPCENYLDVCCKKPQTVDRPITPTPHIRSGCGRRNPDGVGFRITGNNDNEAQFGEFPWMVAILREETVNGNPEKLNVYQCGGSLIHREVVLTAAHCVAGKDVNQMKIRAGEWDTQTKNEIYPHQDRRVKEIIVHENYHAGALYNDVALLLLQDPVDFAENVDVVCLPDRNTIVNSSHCFASGWGKDVFGKEGKYQVILKKVELPIVQHHVCQEALRRTRLGPYYKLDKSFICAGGLAGRDTCKGDGGSPLVCPRPSDPATYEQAGIVAWGIGCGEDGTPGVYANVAGLRNWIDEQMVYNGLDVNAYVP

		Q9GRG2_TENMO Prophenoloxidase activating factor OS=Tenebrio molitor GN=PPAF PE=2 SV=1	
LMI_GLEAN_10 126942	COG0638	threonine-type endopeptidase activity; Molecular Function GO:0005839; proteasome core complex; Cellular Component GO:0051603; proteolysis involved in cellular protein catabolic process; Biological Process IPR001353; Proteasome, subunit alpha/beta NV18121; similar to proteasome beta 2 subunit-like protein; K02734 20S proteasome subunit beta 4 [EC:3.4.25.1] PSB2_BOVIN Proteasome subunit beta type-2 OS=Bos taurus GN=PSMB2 PE=1 SV=1 A2IAA2_9NEOP Proteasome subunit beta type OS=Xenopsylla cheopis PE=2 SV=1	METLLGIAFRDFVMVAADMTHAKSIMVMKDDEDKLQKLSEKLVMAIAGE AGDTTQFSEYIAKNIQLYKMRNGYELSPAAAASFTRRNLAEYLRSRTPYN VNMLIAGYDDAGGPELYFLDYLASLVKIPYAAHGFGGYFALSIMDRYHKP GMTVEEGYELLKKCVREVQKRLIINLPNFKVQVIDKNGIKDLPPITVATLA AEV
LMI_GLEAN_10	COG5640	catalytic activity; Molecular	MCRRGSRPCLAWRGCLRRRCCCWVSVAGALISAQHCSTNCYSRLSCPAV

072756		Function GO:0004252; serine-type endopeptidase activity; Molecular Function GO:0006508; proteolysis; Biological Process IPR001254; Peptidase S1/S6, chymotrypsin/Hap IPR001314; Peptidase S1A, chymotrypsin-type IPR006604; Disulphide knot CLIP IPR009003; Peptidase cysteine/serine, trypsin-like PROC; protein C (inactivator of coagulation factors Va and VIIIa) (EC:3.4.21.69); K01344 protein C (activated) [EC:3.4.21.69] PROC_PIG Vitamin K-dependent protein C OS=Sus scrofa GN=PROC PE=2 SV=1 B0WRU4_CULQU Tryptase gamma OS=Culex quinquefasciatus GN=CpipJ_CPIJ009480 PE=3 SV=1	YSMVCKNLAGYRGFSAQQFERNNGFSQTRFLRFSRVTVFALLPVATPQEK NDTKTLVSEPEDKAYQKVFLHCGVAVTQVSEPEDKAYQRCLTASGQQEH CRSLQYCLPADFRRTFTSVLDSMCIINRAFVGICCPDKITSEDGPVLTDDGS PPSLDEIVSRLNNPTCGRTQQGEWRWMVALIRYGEEHFCGGALISETHVLT AAHCIEPYKLADITVRLGEYDLNRPDETRAVEVRVMEATMHPRYDRATY ENDIAVLRLRRRAAFNSYIMPVCLPAIDEGFINRTAYVTAGRRKLHPMIFQ LCTCQSSSGEPTRTDEDSGYQLNIAWNPVISEICSRRRYHKTRQLILAVPPV RAMPPGGVVLLSRSPRLRRMRGNTRTTSVLHKAELRASSSVLVGSPEDDW QVPN
LMI_GLEAN_10	COG5640	catalytic activity; Molecular	MCGASIISSNWALSAAHCVENFVPKSIMLRAGTSIRGSGGTIHNVASSHIHE

166969		Function GO:0004252; serine-type endopeptidase activity; Molecular Function GO:0006508; proteolysis; Biological Process IPR001254; Peptidase S1/S6, chymotrypsin/Hap IPR001314; Peptidase S1A, chymotrypsin-type IPR009003; Peptidase cysteine/serine, trypsin-like SP22; serine protease 22; K01312 trypsin [EC:3.4.21.4] TRY1_ANOGA Trypsin-1 OS=Anopheles gambiae GN=TRYP1 PE=2 SV=3 Q5QBF4_9DIPT Serine protease OS=Culicoides sonorensis PE=2 SV=1	KYENDDYDIVALEVIEAFVFSSTVKAVDLATTEPTPGTPVTVTGWGTTSSG GQVSNTLLAATVNVVDRKQCKRVYRYITDRMICAGVDGGGKDACQGDS GGPLVSDSTLVGIVSWGHRCALPDFPGVYANVANLRDWIQDITGV
LMI_GLEAN_10 109880	COG4870	cysteine-type endopeptidase activity; Molecular Function GO:0006508; proteolysis; Biological Process GO:0008234; cysteine-type peptidase activity; Molecular Function GO:0050790;	MPHTTAHWERRNRHTAATAAEGTSPAGEAVSTPRKRIAHQLKREGRGPT GKGPSANKKLTPREQLEKKILTPSAGRNFPEETPVKHLKRLMGVHPDSYLF QPPVQRHAVEDLDIPEEFDSREKWSFCPTIKEIRDQGSCGSCWAFGAVEAM SDRVCIHSKGEKNFHFSAEDLVSCCRSCGFGCNGGFPGAAWAYWKRKGI VSGGAYNSSQGCQPYEIPPCEHHVNGTRLPCTGEGGNTPRCEKQCEDGYP VSYTEDLHYGETAYSIEGVSKQIQAEIMKNGPVEGAFTVYEDFVHYKSGV YQHVTGTALGGHAIKIIGWGVLDDTPFWLVANSWNSDWGDGGFFRIKRG

		regulation of catalytic activity; Biological Process IPR000668; Peptidase C1A, papain C-terminal IPR012599; Peptidase C1A, propeptide cathepsin B precursor; K01363 cathepsin B [EC:3.4.22.1] CATB_RAT Cathepsin B OS=Rattus norvegicus GN=Ctsb PE=1 SV=2 Q70EX1_DIAVI Cathepsin B-like proteinase OS=Diabrotica virgifera virgifera PE=2 SV=1	NNECGIESQINAGLPRIN
LMI_GLEAN_10034188	COG4826	serine-type endopeptidase inhibitor activity; Molecular Function IPR000215; Protease inhibitor I4, serpin similar to CG9453-PB, isoform B; K13963 serpin B ILEU_BOVIN Leukocyte elastase inhibitor OS=Bos taurus GN=SERPINB1 PE=2 SV=2 Q8IS51_CTEFE Serpin 5 (Fragment) OS=Ctenocephalides felis	EPGNLFFSPLSIQVILALTFLGAKDNTARQMAKGLRIPEDTAVVEEGVGALMNRLQENNEVRLDIANRIFLKPNYSIKEEFNLSATRFKAGVEEVDFLEEEKARKTINDWVENNTNHKIKEIIPSGILNDLTRMVLVNAIYFRGDWVNKFDKDDTMPLPFHSVGGSSKNVDMMSLEETFKYSEISDLNCQVLLLPYEGERLSMMIFLPREVNGLAVLEEKLADFSLQDTLNNMYYTSVQVYLPKFKIEYSKELKDSLTRVSIAQ

		PE=2 SV=1	
LMI_GLEAN_10 193535	COG4870	cathepsin L [Riptortus pedestris]	MKAYVVLLCVIAGAQAVAFFDLFLEEWNTFKLKHGKKYTSPTEEKFRMKI FMENKKRIAQHNAAYERGEVSYQLELNKFGDLLTHEFGATYNGFNKTHN AKLRESLGVRGSTFIPPANVNVPPYMDWRKHGAVTEVKNQGSCGSCWAF SSTGALEGQHFRKAGYLISLSEQNLIDCSHKYGNQGCNGGLMDQAFTYVR DNRGIDTEESYPYDGDDEKCRFKRIDVGADDTGFVDIPEGDEEQLKVAVA TVGPISVAIDAGHDSFQFYSNGVYFEPQCSPKNLDHGVLIVGYGTTEDGVP YWLVKNSWGPEWGEDGYIKMARNRDNNCGIASSASYPLV
LMI_GLEAN_10 179318	COG1404	serine-type endopeptidase activity; Molecular Function GO:0006508; proteolysis; Biological Process IPR000209; Peptidase S8/S53, subtilisin/kexin/sedolisin IPR022229; Peptidase S8A, tripeptidyl peptidase II TppII; tripeptidyl-peptidase 2; K01280 tripeptidyl-peptidase II [EC:3.4.14.10] TPP2_HUMAN Tripeptidyl-peptidase 2 OS=Homo sapiens GN=TPP2 PE=1 SV=4 B7PQH8_IXOSC Tripeptidyl-peptidase II, putative OS=Ixodes scapularis	MSDQVDCEFPVWGLLPKKETGVQSFLNKFPEFDGRGITIAIFDSGVDPGAP GLQVTTEGKPKVIERYDCSGAGDVDTSVVVRAQDGKIQGVSGRQLKIPST WKNPSGDYHIGIKNAFDLYPSKLRERIEKERKENLWDHGHKAVVSKATRE LQEFEAKNTSNLSPADKLQKEDLEARVDVLTSLDKKYNDVGPAYDCIVFH DGEMWRACLDTSECGDLEKCKVLGEYSKTQEFAVLTKSDLLNYSINVHN EGNTLEVVSMCSTHGTHVASIAAAYFPDEPERNGVAPGAQIVSLTIGDNRL GSMETGTALVRAMIKVMERTQNNKIHVINMSYGEHAHFSSSGRIGELMNE VVNKYGVIWVASAGNHGPALCTIGTPPDISTSIIIGVGAYVSPDMMVAEYS LREKQPGMPYTWTSRGPTIDGDFGVTVCAPGGAITSVPNFTLRNSQLMNG TSMASPHVCGAIATILSGLMKKNMSYSPYSVKRALENTANFLENVDKFAQ GHGLLQVEKAFDHLCTYQDQPERDVRFHVSCGVNNSKGIHLRWGMQER MKEFNINVEPYFLDPDNREHNDKIDFRIKFALTCNEPWVQFPAHLDLMYM ARSFSVKIDPTGLPDGVFFTSLKAYDISCTSKGPLFQVPITVVKPFQIPRELL RPEMSFKNVLFKPNTIHRHFVLVPDKATWAVIRLQSVDKDKSGRFVIHCL QLRPKLVCKTLECHKVVNVTAQSEVVQGFPVRGGLVLEVVVAKYWANL GDVPIDYSVSFHGVRPECPAITMQASDGIMSVELQSGLRNEEVAPTISLKNS VQVLRPTESKVTPLTSRDVIPPARQIYELQLTYNFHIAKGTEVIPNCPLLSDL LYESEFESQLWMLFDCNKQLLATGDAYPNKYIVKLEKGDYVIRMHVRHE

		GN=IscW_ISCW006099 PE=4 SV=1	RKDLLDKMTELSLLLVQKLSTPISLDVYASQTQATTCGKKMLCASLPPNHVLPFYIAPLSTEKATKGIPPGQFLTGSITYAKDDIGKKVDSYPFKYVLNEAPKKSNKSTEPKEKTKEEEFEEAVRDVKTAWLAKLDPGDKANSLYEELKSSYPDHLPVYTAMLQALDPAENKKQLPSLESPEQTEEVSKLAHKVIEISDIIISKIDDIQLLAYFGAKNDNRPDAAKVKTTMERQKQALIESLYRRGVAYCRLYRILENEGKNKEDCKEELSKLMNNVDEAWHSLLKFIDPTDSKVIYFSMWHSFVHQHYGRVMKMLLKLTEDKPTRELDERIVDFARLLNWDHVAQLLQSSLP
LMI_GLEAN_10152801	COG5640	catalytic activity; Molecular Function GO:0004252; serine-type endopeptidase activity; Molecular Function GO:0006508; proteolysis; Biological Process IPR001254; Peptidase S1/S6, chymotrypsin/Hap IPR001314; Peptidase S1A, chymotrypsin-type IPR009003; Peptidase cysteine/serine, trypsin-like KLK12; kallikrein-related peptidase 12; K09621 kallikrein 12 [EC:3.4.21.-] KLK1_MOUSE Kallikrein-1 OS=Mus musculus GN=Klk1 PE=1 SV=3 B0X1E9_CULQU	MLRHALLLFALAGCVLGQFPYQVSLQYVTWEFTLIECTGSLITPMRVLTAGHCCGATNTAVAGVVDLDGCEEAKQESKVLDQIYHPDFPVGYYGIYANDIAVFTLETAFTLNEYVQTIPLATAGSIPTANSSAVGSAWGSSPDNLQWVELSIIDYETCRQLVDDLGVIGENFVVDTMVCTVPITDGVGLCSGDSGGPLVQEGALIGIAQWSNVECGKEGVPSGFTRVSAFIDFINEHI

		Chymotrypsin A OS=Culex quinquefasciatus GN=CpipJ_CPIJ013043 PE=3 SV=1	
LMI_GLEAN_10 177179	COG5640	Serine protease snake, partial [Zootermopsis nevadensis]	QAVLGYGNLNDTKWVCAGVLVSESYVLTTARCLGNRNLGLVSRVRLGD VDLTTPSGDDETVQLIEVDKVTPHPLYQPRARFHDLALLHLRTPAKLSSRV RPACLNSLIKLPTSRVIIAGFGVASDQSTEFGTARYVDVSEMPASQCEKALP QLSRIGVKLNESLICAADEAGGRDSCRGDSGGPLQVRLDKPHCQFSVAGLS SFGPVFCGQPGVPSVYTGIAEYLPWIESVVWP
LMI_GLEAN_10 191067	COG5640	Chymotrypsin BI [Zootermopsis nevadensis]	MLLLISFNCVADITDNCTDEDVLGIEENSGLTFGADSQEDDVDDNDAAISP PSHSTGLEREIEVVSDEGRITNGEVATLGQFPYQAGLIITVDGGQAFCGGSII SDSWILTAAHCADPGTLFEVHLGALYIREDESQQVVLSSTDKVVHSRYGA SPPSNDIALVQLPSAVTFNDYIKAVNLPSSAELSNKFVGAETTVSGWGKPS DDSDSISEVMRYAVAPVISHLSCNLHYFLMIDDDMHICTSGDGGVSTCNG DSGGPLVIYESDGSATQIGIVSFGIALGCEIGWPPVFTRVTNYMDWISENTG IAIRK
LMI_GLEAN_10 125597	COG5640	PREDICTED: trypsin II-P29 [Tribolium castaneum]	MTHREKTEILEKRKGDGVLSRGTSVYEIRKRSRSTDQHAEGGAVPRRAAL LXXXXXXXXXXXXXSAPPPTPSPPTPPFVEPQPVTPTAQPPVDVPVGPVK SGCGVRRGMSPANDNATTFAEFPWMLVVQEAVYPGDGKPPVFIFKCGAS LVHPQVALTAAHCLKRLIPQDVKVRAGIWDLTATAEPLPHQERRVIKIIRH PLFEEKTLFNDIALLVLDKTFDEALNVEVTCLPPPSVDLTGLRCIATGWGTS SPDTEIVQTTMKKVELPIVVRDRCENELRKTRLGRYFELHESFICAGGESN VDTCRGSVLHNTGYTEAVPAESAFFRKSPQEKDDDSAKLPTDVHNLHDSA YRVTSLYGSGKYKRHAGDGGGPLVCPITGDDGRYIQVGIVSWGIGCGGSR TPAVYASLSYFRDWVDKQLEDNLFKNIAIPPPSVEEDERLKVPKPTWG
**glutathione S-transferase**			

LMI_GLEAN_10046649	COG0625	GSTO1 [Locusta migratoria]	MSQKHLSRGSRLPPFPKGKLRLYSMRFCPYAQRVHLVLDAKRIPYEVVNVDLTEKPDWLYEKSPFGKVPAIELESGDTLYESLIICDFLDEKYPSRSLYSRDPLKKAKDKIMIDHFNKVIQSMLKVYYHTANSNLNEDQLEEFFQGLDLYERELVERGKSFFGGDRPGMLDYMIWPWCERSDMMKVLGGDQFLLPKDRFKRLMEWRNQMKEDDAVKESYLEPQVHAKYFQSRKAGYPDYDMLVGN
LMI_GLEAN_10185030	COG0625	epsilon glutathione S-transferase [Locusta migratoria]	MPVTLYHFPASPPSRIALAAAKVVGVDVDVKIVDLFAKEQLKEEFVKINPQHTIPTIDDNGFILWDSHAIATYLVSQYAKDDSLYPKDTKKKAVVDQRLYFEIGTLYPRMRAIAFPVLFLGKTSVEDSVKASAYEAIGFLEKFLEASGWVAGDHLTIADVACAVTATSMQAIGLDFSGYPKTKDWIERCKKIPGFQEANEEGAKIFGERVRSRLPPNHL
LMI_gi_329564865	COG0625	glutathione S-transferase delta [Locusta migratoria]	MPSVDLYYVPGSAPCRAVQMVAKAVGVDLNLKLVNLMEGEQMKPEYLKMNPQHTVPTIDDNGLYLWESRAIIGYLVEQYAKDDSLYPKEAKKRALVNQRLYFDIGTLYARFADYYYPVMFGGASYDPEKLKKLEEAYEFLNKFLEGSDWVAGNSITIADYSIMASVSTAEIIGFDIKKFPKVAAWFEKAKKEIPSYEETNQAGALEFKKLFDSMTAKK
LMI_gi_329564883	COG0625	glutathione S-transferase theta 1 [Locusta migratoria]	MSLKVYYDLLSQPSRAVVLFLLANDIPYEAREINVLHGEQFSEEFAKLNPMKKVPVIKDGDFTLTESVAILRYLCRERDVPDHWYPADSKKQARVDEYLEWQHTNTRSNCALYFLNKFMLPAIKGTQPNPETVARRERKMVATLNEVEEIWLRNKTYLAGDKISIADLLGACEIEQTRMAGYNPCDGRPKLAAWLERVRCDTLPHYDTVHALVRKVTEKYGGVPPASFSKL
LMI_GLEAN_10053940	COG0386	glutathione peroxidase activity; Molecular Function GO:0006979; response to oxidative stress; Biological Process GO:0055114; oxidation-reduction process;	GIALKTSPAALAAYILEKFSTWTNPGWRALPDGGLTRKYTLTDLLDNVMIYWITGSITTSVRLYSESFNKAQYALNLDSIPNETPTACAQFVNEIVFTPESLVRDKHVNLVRYSVMPRGGHFAAFEEPQLLADDIUPGAIRYEPAEGIEALQSRSARPLRSGSASTIYDFTAKDIKGEEVSLEKYRGHVCIIVNVASKCGLTATNYKELEDLKKAYGESQGLRILAFPCNQFNGQEPGDSEQIACFALKTMGATFDLFEKVDVNGDDAHPLWKFLKKKQPGTLGNAIKWNYTKFVVDRNGIPVERF

		Biological Process IPR000889; Glutathione peroxidase IPR012336; Thioredoxin-like fold phospholipid hydroperoxide glutathione peroxidase, putative (EC:1.11.1.12); K00432 glutathione peroxidase [EC:1.11.1.9] GPX4_CALJA Phospholipid hydroperoxide glutathione peroxidase, mitochondrial OS=Callithrix jacchus GN=GPX4 PE=2 SV=2 Q5K6H6_AEDAE Glutathione peroxidase OS=Aedes aegypti GN=GPx PE=2 SV=1	APTTEPKKMESTLKKYF
LMI_GLEAN_10 073548	COG0625	glutathione transferase activity; Molecular Function GO:0005515; protein binding; Molecular Function GO:0005737; cytoplasm; Cellular Component GO:0008152; metabolic process; Biological Process IPR004045; Glutathione	GDPQPAAAEGGKLRLYSMAFCPYAHRARLALQVKGAPFDIVNINLLDKPE WLTAVHPQGKVPALDAGAGHIVVESVDIADFLDGKFPEPPLWPADEDTKA RHKKLIDDFGKVIHHSFKVDTAFLLLRGNSRDGEGDPPRNNGYFVAGSCP GMLDYMIWPWAERAK

		S-transferase, N-terminal IPR005442; Glutathione S-transferase, omega-class IPR010987; Glutathione S-transferase, C-terminal-like IPR012336; Thioredoxin-like fold IPR017933; Glutathione S-transferase/chloride channel, C-terminal similar to CG6776-PA; K00799 glutathione S-transferase [EC:2.5.1.18] GSTO2_HUMAN Glutathione S-transferase omega-2 OS=Homo sapiens GN=GSTO2 PE=2 SV=1 B4HJJ6_DROSE GM25061 OS=Drosophila sechellia GN=GM25061 PE=4 SV=1	
**Protein disulfide-isomerase**			
LMI_GLEAN_10 173072	COG3118	Protein disulfide-isomerase [Zootermopsis nevadensis]	MLMLCFIVCLVGSVWSSVDIKTDEGVLVLNKDNFESAISDTEFILVEFYAP WCGHCKSLAPEYAKAAKKLEEQQSAIKLAKVDATEETDLAEKHGVRGYP TLKFFRKGSPVDYTGGRTSDDIVNWLLKKTGPVAKELGSVDEAKSFIDSA DVVVVGFFKDQGSSAAKVFLEAAATIDDHPIGVSSSDDVFSEYSANDGNII LFKKFDEGKAVMDGEVTEQTIKKFVAAQSLPLVVEFNHDTAQKIFGGEIKS HLLIFLSKEAGHYDKYLDAARESAKGFREQLLFVSINADEEDHQRILELFG

			MKKEEVPAMRLIRLEEDMAKYKPENPEITADNIKNFVQGFLDGNLKQHLL SQTLPDDWDKTPVKVLVSTNFNEIAFDKSKDVLVEFYAPWCGHCKQLAPI YDQLGEKFKDSDSVVIAKMDATANELEHTKITSFPTLKLYTKGENKVIEYN GERTLDGLTKFLESGGTYGQAAPDEAEEEDEDDDLPRKDEL
LMI_GLEAN_10 123367	COG0526	protein disulfide-isomerase [Schistocerca gregaria]	MNEDMYLNLLYVIMHVIKKQDTIKREAITPHERLTAVLRLLATGRTYEDF RYYFAKALGEIFQIHASFKPVACGGLQLESAGVVAGDLASPISVDWANTTS GRSGPVLAAEEDVLELTDEDFSTRIQEHDTMLVMFYAPWCGHCKKLKPE YAKAAGIIKDNDPPVTLAKVDCTEAGKETCNKFSVTGYPTLKIFRRGELSM DYSGPREAAGIVKYLKSQVGPSSKDLLTETAFEDFISKDDVAVVGFFEEES DLKLAFLRVADKLREKARFGHTSNRDLLKKGVSDGIILYRPKHLHNKFEP NSITYDGEAKKEAIESWINKEYHGLVGHRQRDNTQDFKNPLVVAYYGVD YVKNPKGTNYWRNRILKVAKSFASVFNFAISAKDDFQHELNEFGFDYVKG DKPVIFARNAKNQKFVLSDEFSMETFEKFLNDLKDEKLEPYLKSEPIPEDN DGPVKIAVAKNFDEIVTNNGQDTLIEFYAPWCGHCKKLAPVYEELGEKMK GEDVAIVKMDASNNDVPEPYEVRGFPTLYWASKDGKSNPVRYDGGRELD DFIKYIAKQSTNELKGWDRKGKTKKQEL
**Peroxiredoxin**			
LMI_GLEAN_10 179379	COG0450	Peroxiredoxin-6 [Zootermopsis nevadensis]	MKLETVVPNFKAPSTQGPLDFYKWKGNSTDIKSYCKDIPGDFPYPIVSDET RELAVKLDMIDERDKDDVEKAMTVRAMYVIGPDNRLRLSMVYPASCGR NVEWCVLFSHPADFTPVCTTELGRIAVHNPEFQKRGVKLLALSCDKLKDH VDWVNDIKSYCKDIPGDFPYPIVSDETRELAVKLDMIDERDKDDVEKAMT VRAMYVIGPDNRLRLSMVYPASCGRNVDELLRVIDSLQLTDRLKVVATPA NWTPGTKVMILPHVPDSDLPRLFPGGVERVSMPSGNNYVRTTTDY
LMI_GLEAN_10 125164	COG0450	Peroxiredoxin-6 [Zootermopsis nevadensis]	MKLESIVPNFKAPSTQGPLEFYKWKGNSWCVLFSHPADFTPVCTTELGRIA VHNPEFQKRGVKLLALSCDKLKDHVDWVNDIKSYCKDIPGDFPYPIVSDE TRELAVKLDMIDERDKDDVEKAMTVRAMYVIGPDNRLRLSMVYPASCGR

			NVDELLRVIDSLQLTDRLKVVATPANWTPGTKVMILPHVPDSDLPRLFPGGVERVSMPSGNNYVRTTTDY
LMI_GLEAN_10025235	COG0678	Peroxiredoxin-5, mitochondrial [Zootermopsis nevadensis]	MGLKVGDKLPSVELYENNPTNKVNLEKLLSNKKAVVFAVPGAFTPGCSKTHLPGYVEKADELKKKGIDEIICVSVNDPFVMDAWGKEHKATGKVRMLADPDASFTKALELETNLPPLGGTRSKRYSMIIENGVVKSLNVEPDGTGLSCSLADKLPL
LMI_GLEAN_10179629	COG0450	Peroxiredoxin-6 [Zootermopsis nevadensis	MFFQFVIVEVLTGQIAGVRRGFPSKGGVCFHDSAGPHVANVTKQLLRKFQWDVWCILFSHPADFTPVCTTELARMAERAYEFTKRGVRMIGHSCDSVAKHLVWLQDIRAFARNLPAEFPYPIIADETRELAVALDMLDESERGLDGEPVSVRAVLIIAPDRRLRLSMYYPNTTGRNVDEILRVIDSLLLTERLRVVTPVDWTAIEERFVSRLYSQLITHYDDSAPYPLDPLTHDIYWIPARDLAFVSVRIACWRLYTIMSVLSDMSKAFCVRPFYKNIDLVNVASRKFYTYRGLHYVHDALIRNQGYEDHQERPNLRTLSIGLLLS
**thioredoxin**			
LMI_GLEAN_10036727	COG0526	PREDICTED: thioredoxin-2-like isoform X1 [Nasonia vitripennis]	MSITAVKVLKTWYTDDLKARLADAGANLVVIDFFADWCGPCKAIAPKFEELSQKYPDVVFLKVNVDDNENIAATYDVKAMPTFVFIKDGKTEGITKVIEYNVRKSGIAAGIILGNE
LMI_GLEAN_10051563	COG1222	PREDICTED: thioredoxin-2-like isoform X1 [Nasonia vitripennis]	MAKGNVGSVVTDPAREKALHDYRKKLLEHKEVEGRLKEMREQLKELTKQYDKSENDLKALQSVGQIVGEVLKQLTEEKFIVKATNGPRYVVGCRRQLDKARLKSGTRVALDMTTLTIMRYLPREVDPLVYNMSHEDPGEVTYSAIGGLSEQIRELREVIELPLLNPELFQRVGITPPKGCLLYGPPGTGKTLLARAVASQLDANFLKVVSSAIVDKYIGESARLIREMFNYARDHQPCIIFMDEIDAIGGRRFSEGTSADREIQRTLMELLNQMDGFDSLGQVKIIMATNRPDTLDPALLRPGRLDRKIEIPLPNEQARLEILKIHAGPIAKHGEIDYEAVVKLSDGFNGADLRNVCTEAGLFAIRAEREYVIQEDFMKAVRKVADNKKLESKLDYKPV
LMI_GLEAN_10	COG3118	PREDICTED: thioredoxin,	MHRVLINISPLLRHTSKECINAFGTKPTLVRHVSASCINFNSFKIQDVNDFN

071989		mitochondrial-like [Megachile rotundata]	ERVKNSKVPVIVDFFATWCNPCRMLTPRIESVIAEKKGQIVLAKVDIDEQT DLALDYDVSSVPVLVAIKDGKEEDRLIGLQDVDKLKKFVDNICGSSEAAA AKAS
LMI_GLEAN_10 071989	COG0526	PREDICTED: thioredoxin, mitochondrial-like [Megachile rotundata]	MHRVLINISPLLRHTSKECINAFGTKPTLVRHVSASCINFNSFKIQDVNDFN ERVKNSKVPVIVDFFATWCNPCRMLTPRIESVIAEKKGQIVLAKVDIDEQT DLALDYDVSSVPVLVAIKDGKEEDRLIGLQDVDKLKKFVDNICGSSEAAA AKAS
LMI_GLEAN_10 111751	COG0450	thioredoxin peroxidase-like protein [Microplitis demolitor] Sequence ID: gb|EZA45433.1|	MPVPELAKPAPAFSGVAVVGTNFEDIKLSDYKGKYLVLFFYPLDFTFVCPT EIIAFSDRIDEFKKLNCEVIAVSCDSQYSHLAWVNTPRKNGGVSKLNIPLLA DKSMKIAKDYGVLNEETGIPYRGLFVIDGKQNLRQITINDLPVGRSVDETL RLITAFQHTDVHGEVCPAGWKPGDKTMKPNPVDSKEYFSTTNHS
**Peptidyl-prolyl cis-trans isomerase**			
LMI_GLEAN_10 064671	COG0652	PREDICTED: peptidyl-prolyl cis-trans isomerase, rhodopsin-specific isozyme-like [Bombus terrestris]	MTARTGFSSEQAKEYKVTDQVYFDITHGDEDLGRIVIGLFGDIAPKTVKNF IVIATKGVDGKTYAGSGFHRVIKKFMIQGGDIVNNDGTGSTSIYGKYFDDE GFELTHSGPGFISMANAGKNTNGCQFFITTVPTPWLDGHHTIFGKVVTGQN IVHTIEHVPTDSSDRPLKPVIIKASGIIDTPQPFTITDELYDLWAWIKASAVPL FFSFSVLSFFHWMIKKLNVDV
LMI_GLEAN_10 121349	COG0652	Peptidyl-prolyl cis-trans isomerase [Zootermopsis nevadensis]	MALAVLNLCRQSGFSLQRIGGSRGLQLSLSSNATRYFATKMSRPKVFFDM AADNQPIGRIVIELRADVVPKTAENFRALCTGEKGFGYKGSTFHRVIPNFM CQGGDFTNHNGTGGKSIYGNKFEDENFTLKHTGPGILSMANAGPNTNGSQ FFITTAKTSWLDNRHVVFGSVVEGMDVVKKIESFGSQSGKTSKKIVVNDC GQI
LMI_GLEAN_10 119039	COG0652	peptidyl-prolyl cis-trans isomerase activity; Molecular Function GO:0006457; protein folding; Biological Process	MARWQVLVPVFVFVLCYVVSAEGEQKKGPKVTDKVWFDIKIGEEKVGRV EIGLFGKTVPKTVKNFVELAQKPAGEGYKGSKFHRVIKDFMIQGGDFTKG DGTGGRSIYGDRFADENFKLKHYGAGWLSMANAGKDTNGSQFFITTKQT PWLDGRHVVFGKVLKGMDIVRKIESTPTDGRDRPNQDVVIADCGAETVSE

		IPR002130; Peptidyl-prolyl cis-trans isomerase, cyclophilin-type IPR015891; Cyclophilin-like similar to CG2852-PA, isoform A; K03768 peptidyl-prolyl cis-trans isomerase B (cyclophilin B) [EC:5.2.1.8] PPIB_BOVIN Peptidyl-prolyl cis-trans isomerase B OS=Bos taurus GN=PPIB PE=1 SV=4 B4J4H9_DROGR Peptidyl-prolyl cis-trans isomerase OS=Drosophila grimshawi GN=GH20332 PE=3 SV=1	PFSVSKDDASE
	**COP9 signalosome complex**		
LMI_GLEAN_10 094977	COG5071	COP9 signalosome complex subunit 4 [Zootermopsis nevadensis]	MTTRCHTPKNFIDKYRAILEAVLTLQGNDLIEALKVFIEAIVNENVSLVISR QILTDVSTQLAVAPDNISKAVSHYTLDKVQPRVISFEEQVAAIRQHLAEIYE REQAWREAANVLVGIPLETGQKQYTVDYKLDTYLKIARLYLEDDDPVQA EAYINRASLLQAESRNEQLQIYYKVCYARVLDYRRKFIEAAQRYNELSYRS IIHEDERMTALRNALICTVLASAGQQRSRMLATLFKDERCQQLPAYSILEK MYLDRIIRRSELQEFGNLLQPHQKALTPDGSTILERAVIEHNLLSASKLYNN ITFEELGALLEIPPIKAEKIASQMITEGRMNGYIDQIDSIVHFETREVLPTWD KQIQSLCYQVNQIIEKITSCEPEWIAKAMEEQMIH
LMI_GLEAN_10	COG5159	COP9 signalosome complex	MSDCEVDFMCEEEEDYGLEYSEDSNSEPDVDLENQYYNSKALKEDDPKA

027555		subunit 2; K12176 COP9 signalosome complex subunit 2 CSN2_RAT COP9 signalosome complex subunit 2 OS=Rattus norvegicus GN=Cops2 PE=2 SV=1 Q178X3_AEDAE Cop9 signalosome complex subunit OS=Aedes aegypti GN=AAEL005730 PE=4 SV=1	ALASFQKVLDLEGGEKGEWGFKALKQMIKINFKLGNYKEMMARYKQLLTYIKSAVTRNHSEKSINSILDYISTSKNMELLQDFYETTLDALKDAKNDRLWFKTNTKLGKLYFDRSDFNKLAKILKQLHQSCQTDEGEDDLKKGTQLLEIYALEIQMYTAQKNNKKLKTLYEQSLHIKSAIPHPLIMGVIRECGGKMHLREGEFEKAHTDFFEAFKNYDESGSPRRTTCLKYLVLANMLMKSGINPFDSQEAKPYKNDPEILAMTNLVSAYQNNDINEFETILKQNRRNIMDDPFIREHIEDLLRNIRTQVLIKLITPYTRIHIPFISKELNIDASEVESLLVSCILDSTIRGRIDQVNQVLELDKQSLCATRYYALDKWTSQLATLHVAVVNKMA
**Others**			
LMI_GLEAN_10127792	COG5187	protein binding; Molecular Function IPR000717; Proteasome component (PCI) domain IPR019585; 26S proteasome, regulatory subunit Rpn7 similar to 26S proteasome non-ATPase regulatory subunit 6 (26S proteasome regulatory subunit S10) (Rpn7 protein); K03037 26S proteasome regulatory subunit N7 PSMD6_DROME 26S proteasome non-ATPase regulatory subunit 6	MPVENMEEEGLDKNPNLMLAQWRFLLSLPEHANDSQVKHQLMEAITRDNMAPFYEECCSSLGWQLDEELMSRMQAANTQRLKELDEAIEDAEKNLGEMEVRDANLKKSEYLCKIGAKDLSLQCFRKTYEKTVSLGNRLDIVFHIIRIGLFYLDHDLITKNIDKAKSLIDEGGDWDRRNRLKVYQGTYCMVIRDFKSAAAYFLDTISTFTSYELMDYNTFVRYTVYISMISLPRNELRDKIVKGSEILEVLHSNADVKDYLFSLYNCLYADFFKNLAHVEGLLKRDYFLNPHYRFYVREMRIMAYSQLLESYRSLTLQYMADAFGVTIDYIDQELSRFISAGRLHCKIDKVGGVVETNRPDSKNWQYQATIKQGDILLNRVQKLSRVINI

		OS=Drosophila melanogaster GN=Rpn7 PE=1 SV=1 Q7PUS4_ANOGA AGAP002061-PA OS=Anopheles gambiae GN=AGAP002061 PE=4 SV=2	
LMI_GLEAN_10 130177	COG1382	protein folding; Biological Process GO:0016272; prefoldin complex; Cellular Component GO:0051082; unfolded protein binding; Molecular Function IPR002777; Prefoldin beta-like IPR009053; Prefoldin similar to Probable prefoldin subunit 4; K09550 prefoldin subunit 4 PFD4_DROME Probable prefoldin subunit 4 OS=Drosophila melanogaster GN=CG10635 PE=1 SV=1 C4WSI6_ACYPI ACYPI007432 protein OS=Acyrthosiphon pisum GN=ACYPI007432 PE=2 SV=1	MASSNSATFQPDSDVHISYDDQQKINEFARHNARLEDYKDELKSKQNQLK NLEEACDELTLMDPTEFVPYFMGEVFINQDIEQTEKMLEEAKNKIISETKD LEDKCSNIKNQMSELKTYLYAKFGNHINLEADDD
LMI_GLEAN_10	COG0545	protein folding; Biological	DEWLDILGTGHLKKKIIKKAENEYRPQSLQICEINLEGRLEDGTVVEKCEN

053312		Process IPR001179; Peptidyl-prolyl cis-trans isomerase, FKBP-type similar to CG5482-PA; K09574 FK506-binding protein 8 [EC:5.2.1.8] FKBP8_HUMAN FK506-binding protein 8 OS=Homo sapiens GN=FKBP8 PE=1 SV=2 Q16MK5_AEDAE Fk506 binding protein OS=Aedes aegypti GN=AAEL012272 PE=4 SV=1	LKIQIGDVEVVQGIDLALPLMNVGEVALLEVGPRFAYGTIGRSPDIPPDAKL IYTIELLSAEDEPDLESLSIARRKELGDKKRERGNWWFSRQEYSFAIQSYRR AIEFLDDAEGGFSIEKKDISEDAEKQLQELLEDRLKVFNNLAAAQMKIKAY DAALTSVDNVLRCQPQNVKALFRKGKILAAKGDTTTALSVLRNASQLEPE SKVIKQEVQRLLDVIKRDTKQEKSLYKKMLGQNKS
LMI_GLEAN_10 141968	COG1382	protein folding; Biological Process GO:0016272; prefoldin complex; Cellular Component GO:0051082; unfolded protein binding; Molecular Function IPR002777; Prefoldin beta-like IPR009053; Prefoldin similar to Prefoldin subunit 6 (Protein Ke2); K04798 prefoldin beta subunit PFD6_MOUSE Prefoldin subunit 6 OS=Mus musculus	MEKIQEELQRKLDQYKAVQKDFHKAVSQRNQLEGQLNENTAVKTELDLL RKDAEVYKMIDPVLVIQDLEEAKQNVAKRMDYIRGELKRADDTLASFEK QQENHRAALTKLQQQFQQAQMKMAKKP

		GN=Pfdn6 PE=2 SV=1 A7T0K8_NEMVE Predicted protein OS=Nematostella vectensis GN=v1g248362 PE=4 SV=1	
LMI_GLEAN_10 142869	COG0625	protein binding; Molecular Function IPR004045; Glutathione S-transferase, N-terminal IPR010987; Glutathione S-transferase, C-terminal-like IPR012336; Thioredoxin-like fold glutathione-s-transferase theta, gst ; K00799 glutathione S-transferase [EC:2.5.1.18] GSTT6_DROME Glutathione S-transferase D6 OS=Drosophila melanogaster GN=GstD6 PE=1 SV=1 Q16P53_AEDAE Glutathione-s-transferase theta, gst OS=Aedes aegypti GN=AAEL011752 PE=4 SV=1	LKLYSVSDSPPSTAVKMALEALALEYTNVEVDFAAGEHLSDDFSKKNPQR EIPCLDDNGFFLSESVAILQYLADKYGPGHSLYPRDPQQRALVNHRLAFNI STYYARIAEYAVAPIFFDYKRTPEGLNKLKIALNVLNTILERQGTKFAAGE HMTLADLSLVAATMCLEAVKFDLTPWARVQRWYADFKQAAPRLWAVA EPAMLELRAFSNSPPDLSALRHPYHPVR
LMI_GLEAN_10 075935	COG0545	protein folding; Biological Process IPR001179; Peptidyl-prolyl cis-trans	AIDISPKKDNGILKEIIKEGTGSETPAPGSKVFVHYVGTLTDGTKFDSSRDR GEAFDFQLGKGTVIQAWDIGVATMKKGEVAVLTCKSEYAYGKSGSPPKIP PDATLVFEIEVIDWQGEDISPKKDGSITRYQIIQGEGYSSPNDGATVEVNLV

		isomerase, FKBP-type FKBP59; FK506-binding protein FKBP59; K09571 FK506-binding protein 4/5 [EC:5.2.1.8] FKBP4_MOUSE FK506-binding protein 4 OS=Mus musculus GN=Fkbp4 PE=1 SV=5 C1BKB3_OSMMO FK506-binding protein 4 OS=Osmerus mordax GN=FKBP4 PE=2 SV=1	GRHNGCVFEERSVTFPLGEGSEYGICEGVEKALEKFKKGEKSRLIIKPKYAFKKEGKPDLNIPGNAEIEYEVELKNFEKAKEAWSLDANEKIEQAKFFKEKGTNYFKAAKYTLAVKMYKKIVNFLEHETGFEEETEPERKSLLLAGHLNLAMCYLKLEEHFEAREQCNKALELRPSSEKGLFRRGQAYLGMGEPELAKADFEAVLKLEPENKAAANNIVICNVKIKEQRSREKKIYANMFEKFAQRDKEKEEEWRRNQPDVMKTLGEWGQDEREREMTDFERENPNILMLNKTGEFKNM
LMI_GLEAN_10108346	COG3914	protein binding; Molecular Function IPR001440; Tetratricopeptide TPR-1 IPR013026; Tetratricopeptide repeat-containing IPR019734; Tetratricopeptide repeat Ogt, NV10004; O-glycosyltransferase; K09667 polypeptide N-acetylglucosaminyltransferase [EC:2.4.1.-] OGT1_MOUSE UDP-N-acetylglucosamine--pe	MQAQQVQQQAQHQQITGANVILKMNDIQQLSTVSLLELAHREYQAGDYENAERHCMQLWRQETNNTGVLLLLSSIHFQCRRLDKSAHFSTLAIKQNPLLAEAYSNLGNVYKERGQLQEALENYRHAVRLKPDFIDGYINLAAALVAAGDMEQAVQAYVTALQYNPDLYCVRSDLGNLLKALGRLDEAKACYLKAIETRPDFAVAWSNLGCVFNAQGEIWLAIHHFEKAVALDPNFLDAYINLGNVLKEARIFDRAVAAYLRALNLSPNNAVVHGNLACVYYEQGLIDLAIDTYRRAIELQPNFPDAYCNLANALKEKGQVVEAEDCYNTALRLCPTHADSLNNLANIKREQGYIEEATRLYLKALEVFPEFAAAHSNLASVLQQQGKLNEALMHYKEAIRIQPTFADAYSNMGNTLKEMQDIQGALQCYTRAIQINPAFADAHSNLASIHKDSGNIPEAIQSYRTALKLKPDFPDAYCNLAHCLQIVCDWTDYEARMKKLVSIVAEQLDKNRLPSVHPHHSMLYPLSHEFRKAIAARHANLCLEKIHVLHKPPYKYPKELSARLRIGYVSSDFGNHPTSHLMQSIPGLHNKSKVEVYCYALSPDDGTTFRAKITREAEHFVDLSQIPCNGKAADRINSDGIHILVNMNGYT

		ptide N-acetylglucosaminyltransferase 110 kDa subunit OS=Mus musculus GN=Ogt PE=1 SV=2 Q7Q5R0_ANOGA AGAP006254-PA OS=Anopheles gambiae GN=AGAP006254 PE=4 SV=4	KGARNEIFALRPAPVQVMWLGYPGTSGASFMDYLITDVVTSPLELASQYS EKLAYMPHTYFIGDHKQMFPHLKERVILTDRSTGKKEVPDNVAVINATDL SPIMENTSVKEIREVVSSDGKHTSRPVEISLKVAELPTTTPIETMIASGQIQTS VNGVVVQNGLATNQTNNKAATGEEVPQSIVITTRQQYGLPDEAVVYCNF NQLYKIDPLTLQMWVYILKHVPNSVLWLLRFPAVGEPNLQAAAQQLGLPP GRILFSNVAAKEEHVRRGQLADVCLDTPLCNGHTTSMDVLWTGTPVVTLP GETLASRVAASQLATLGCPELVASTRQEYQDIAVRLGTDREYLKATRAKV WRARTESPLFDCLQYAQGMEKLFQKMWERYQRGDKPDHITDVK
LMI_GLEAN_10152658	COG5656	binding; Molecular Function GO:0006886; intracellular protein transport; Biological Process GO:0008565; protein transporter activity; Molecular Function IPR001494; Importin-beta, N-terminal IPR016024; Armadillo-type fold NA XPO2_DROME Exportin-2 OS=Drosophila melanogaster GN=Cas PE=1 SV=2 Q7QE09_ANOGA AGAP010711-PA OS=Anopheles gambiae GN=AGAP010711 PE=4 SV=1	MELSDENLKTLSQYLQHTLNPDSEVRRPAEKFLESVEVNQNYPLLLLHLV DRKDADMTIRIAGAVAFKNYIKRNWSVSEDAADRIHAQDREAVKQLIVNL MLHSPEAIQKQLSDAISIIGRHDFPNKWPNLISQMVEKFDTAWACTIICFWN LNYSGWVIFLTPIVHWKLDILLFSSKYTKQNLMSVVSCLLATMNLRTVHA NNPEALKVIYSSLVIICKVFYSLNFQDLPEFFEDNMGNWMSNFHELLTVDV PCLKNEDDEEAGLLEQLKSQICDNVALYASKYDEEFQSYLPQFVTDIWNL LISTGTQPKYDLLVSNALQFLATVANRSQYRHLFEDPIIMSSICEKIVIPNME FRASDEELFEDNPEEYIRRDIEGSXXXXRSVCDLVKVLSQYFEAKMIEVFG QYVQAMIQRYGESPEQNWRSKDAALYLVTSLAAKGQTQKHGITQSSQLV DLNSFAQEHILTELQKPVNTIPVLKADSIKYIMMFRSVLPREMVVGSLSQIV KHLTAVSHVVHTYAACAIEKILVMKTPDGNSVVKTEEVTPIARELLTNLFS ALDLPGSQENEYVMKAIMRSFSALQAQVIPHLGDLLPKLTQKLALVARNP SKPHFNHYLFETLCLSIKIVCTSNKAAVSTFEASLFPLFQWILQQDVQEFVP YVFQMLSLMLEQHTSGVPEPYMLLFPCLLAPVLWERPGNIHPLVGLLRAFI AQGGSQIIATQKVSGLLGVFQKLIASKANDHEGFFLMQSLIEHLPTEALSPY MKQVFVLLFQRLSSSKTTKFIKGLLVFFCLYCLQYGPANLIGMIDGIQQQM FGMVIERLFIPDLQKISGNVERKIAAVGVTKLLCETPELVDGPYSQFWVPLL

			QALIGLFELPEDQSLLPDDHFIEVDDTPGYQPVFSQLLFANKRDRDPLQGV ADVRLHLAQSLSKLSVAYPGRLQPMLGGISEPHRIHLKTYLDAAKVEIS
LMI_GLEAN_10 088430	COG0464	binding; Molecular Function GO:0005524; ATP binding; Molecular Function IPR003338; ATPase, AAA-type, VAT, N-terminal IPR004201; Cell division protein 48, CDC48, domain 2 IPR009010; Aspartate decarboxylase-like fold similar to Vesicular-fusion protein Nsf1 (N-ethylmaleimide-sensitive fusion protein 1) (NEM-sensitive fusion protein 1) (dNsf-1) (Protein comatose); K06027 vesicle-fusing ATPase [EC:3.6.4.6] NSF1_DROME Vesicle-fusing ATPase 1 OS=Drosophila melanogaster GN=comt PE=1 SV=1 Q9U4Y6_MANSE N-ethylmaleimide sensitive fusion protein OS=Manduca sexta GN=NSF PE=2 SV=1	ISFQRMKVFRCPTDELTLTNCAILNDGDFPSDVKHIEVTPKSVKEYIFSVRF HPDVPRGHVGFSLPQRKWAMLSLNEEIVVKPYYFDATSSSECLCTIVLEAD FLQKKSVSLEPYDTDLMAREFLLQFSGQAFTVGQQLAFSFQDKKLLGLVV KSLEAADLNAIKAGQDAKPKKTRLGRCLGDTIVQFEKAENSSLNLGKAKG KQPRQSIINPDWDFQKMGIGGLDKEFNAIFRRAFASRVFPPEVVEQLGCKH VKGILLYGPPGTGKTLMARQIGTMLNAREPKIVNGPQILDKYVGESEANIR RLFADAEEEEKRLGPNSGLHIIIFDEIDAICKSRGSVAGNTGVHDTVVNQLL AKIDGVEQLNNILVIGMTNRRDMIDEALLRPGRLEVQMEIGLPNENGRFQI LSIHTSRMKDHKKLATDVDIKELAALTKNFSGAELEGLVRAAQSTAMNRL IKASSKVEVDPEAMEKLLVDRSDFLHALEHDIKPAFGTSAEVLEHFLSRGII NWGTPVSGVFEDGMLLIQQARMTDTSGLVSVLLEGPPNSGKTAIAAQLAK DSDFPFVKVCSPEEMVGYTESAKCLQIRKVFDDAYRSQLSCILVDNIERLL DYGPIGPRYSNLTLQALLVLLKKEPPKGRKLLILCTSSRRQVLEDMEMMSA FTAVLHVPNLSKPEHLIAVLEETDVFSKKDVATISRKIQGQRLFIGIKKLLA LIDMARQTEEQNRIIKFLSKLEEEGALE

LMI_GLEAN_10112462	COG5021	binding; Molecular Function GO:0005515; protein binding; Molecular Function IPR002110; Ankyrin repeat IPR016024; Armadillo-type fold IPR020683; Ankyrin repeat-containing domain hect E3 ubiquitin ligase, putative; K12231 E3 ubiquitin-protein ligase HECTD1 [EC:6.3.2.19] HECD1_HUMAN E3 ubiquitin-protein ligase HECTD1 OS=Homo sapiens GN=HECTD1 PE=1 SV=2 Q171B1_AEDAE Hect E3 ubiquitin ligase OS=Aedes aegypti GN=AAEL007705 PE=4 SV=1	MADVDPETLLEWLNMGQGDERDMQLIALEQLCMLLLMSDNVDRCFESCPPRTFLPALCRIFLDECAPDNVLEVTARAITYYLDVSAECTRRIVAMEGAVRAICSHLVVAEPASRTSKDLAEQCVKVLELICTREAGAVFEAGGLSCVLSFIRDNGWRVHKDTLHSAMAVVSRLCTKMEPQDASLPSCVEALSTLLKHEDSHVADGALRCFASLADRFTRRGVDPAPLAEHGLCNELLTRLSNAVGPVVAPNAASGTSGTTAPTVAPETKSSASVSTIISLLSTLCRGSPTITHDLLRSELPDAIEKALKGDERCSLDSMRLVDLLLVLLFEGRKALARSGAGGSQLLPRLRRMDSAGEKTHRQLIDCIRSKDTDALIEAIDSGGIEVNFMDDVGQTLLNWASAFGTQEMVEFLCERGADVNKGQRSSSLHYAACFGRPAIAKVLLRHGANPDLRDEDGKTPLDKARERSDEGHREASAAILQSPGEWMVPQEARDTTEDPEENSEPKGDPEMAPVYLRRLLPVFCITFQSTMLPSVRKASLGLIKKMVHYIQPNLLVEVCSPESPTYNFGTMLVEVIATVLDNEEDEDGHLVVLQIIQDLMTKAQDIFLDHFAKLGVFSKVLQLAGPQDAVEKKEEPPEETEQQPPEPTVEDAKEMVAGKAYHWRDWCLCRGRDCLYIWSDAAALELSNGSNGWFRFILDGKLATMYSSGSPEGGTDSSENRGEFLEKLQRARSQVKPNTPSQPVLTHAGPARLVVGNWALSSRKEGELHIHNSDGQQQTTILREDLPGFIFESNRGTKHSFTAETSLGPEFAAGWTGKRGKRLRSKMEAMKQKVKVQAQEIYDKYFKAAQAQPRGVVAKLGNIVAQIDLACQKQHMRGGDAWRDNLRSALGELTALLQEEGLVSAYELHSSGLVQALLQLLATSYWDQGQHSRTTARLQKQRIRVFKRCFQDKDSGVNSVSVLVHKLVAVLESIEKLPIYLYDNPGSGYGLQILTRRIRFRLEKAPGESSLIDRTGRCLKMEPLATVQQLERYLLKMVAKQWYDYERTSFSFVRRIKEQGRITFLQQHDFDENGLFYWIGTNAKTSPEWVNPAQFGLVTVTSSDGRNLPYGRLEDILSRDTSALNCHTNDDKRAWFAVDLGLWLIPTSYTLRHARGYGRSALRNWLFQVSKDGVNWTTLYTHVDDGSLNEPGSTASWPLEPPADETQGWRHVRLQQAGKNASGQTHYLSLSGLELYGTVTGVCDDLGKAAKEAEASLRRQRRLLRTQVLKQLVVGARVARGLDWKWRDQDGSPPGEGTVTG

			ELHNGWIDVTWDHGGSNSYRMGAEGKYDLRLAGTPSGAVVSDADAPKT<br>PTPPAAQQTSSSKASGKNGSSSVLASRKSSSTPSLPDATEVGAARASVASTE<br>QAASADNLAAKQAAEAIAESVLTVARAEAIVAVAGEAQLQPQGELSVVV<br>HALREPHSDLSAIASGDLATIVETLALGESGSGSGQLRRQASEDNKGWNE<br>QGQGRSKSSRSKAALAAEAADVLDKMREGADLLRNNTNSFLSGELLPSSL<br>LGLMASALAPSVRISVSGSGGESTDSTSSEEKGLGIRIKSVAGGVKIPSSQQ<br>QQQQQQSTSVTSDAFKSRTFPPDLVQKQIARGTYSSPQIQNLPQKNPRSVP<br>LVTRYFLGLERTLNVALQQGYDFLKSCPEMRSILHDILPTPPTGGGPPTSGN<br>AATLFPRGPGSVSGLVRLALSSNFPGGLLSTAQSYPSLTTSGGGSGGGTGS<br>GGVSTTAGGAPCLSQALTMSLTSTSSDSEQVSLEDFLESCRAPTLLAELED<br>DDEIPDADCDDECDENEDDDDEEYEEVMEEEGYEVRGGKRRSWDDDFVL<br>KRQFSALIPAFDPRPGRTNVNQTSDLEVPPPGSDESSSGESSSVSGGSSSDA<br>ETVPQPRLKLSLRGPNQHGVPDVEVPLTDRNWTIFRAVQQLVQSAELGTR<br>QEKLRRIWEPVYTIVYREDDSTPGQDDQQNVDSTHQSSIPSSTTSPSTPATP<br>TMPPACSVDDVLQLLRHLFVISTFRDEENENETEDSEGHLAIEEFTSKKITN<br>KLLQQIQDPLVLSSGALPSWCEELNRSCPFLFPFEMRQLYFNCTAFGASRSI<br>VWLQTQRDVTLERQRAPGLSPRREDPHEFRVGRLKHERVKVPRGEKLLD<br>WAVQVMRLHADRKSILEVEFQGEEGTGLGPTLEFYALVGAELQRCDLGL<br>WLCDEAIDTEVSDSAAYDTAVADLGGVARPPGFYVRRPSGLFPAPLPQDS<br>AACDRATKYFWFLGVFLAKVLQDNRLVDLPLSQPFLKLMCGGDLVSGIGP<br>SSNIRPSDDDPMTSSLLSEESEKELELDPPKPMFITETRPWFHNILGPEDLAI<br>VDPERGKFLSQLSELSIRKARIMQDNTLSPEARAHQVANLALCGGVRLEDL<br>ALTFQYLPGSRDFGFTAIDLIRDGADVEVTLDNIEEYAELTTNFCLERGIAR<br>QMEAFRAGFNRVFPMDKLRAFSPDEVRVMLCGDQNPHWSREDLLTYTEP<br>KLGYTRDSPGFLRFVNVLVAMSAEERKAFXXXXXXFLQFTTGCSSLPPGG<br>LANLYPRLTVVRKVDAGEGSYPSVNTCVHYLKLPDYPTEELLRQRLLAAT

			RERGFHLN
LMI_GLEAN_10085215	COG0526	glycerol ether metabolic process; Biological Process GO:0009055; electron carrier activity; Molecular Function GO:0015035; protein disulfide oxidoreductase activity; Molecular Function GO:0045454; cell redox homeostasis; Biological Process IPR005746; Thioredoxin IPR012336; Thioredoxin-like fold IPR013766; Thioredoxin domain IPR017936; Thioredoxin-like similar to protein disulfide-isomerase A6; K09584 protein disulfide-isomerase A6 [EC:5.3.4.1] PDIA6_RAT Protein disulfide-isomerase A6 OS=Rattus norvegicus GN=Pdia6 PE=1 SV=2 Q1HQU9_AEDAE Protein disulfide isomerase OS=Aedes	MGITRCSLGIEWDGRLPNKLTFAVQQSDPKDVIELTDSNFKSKVLDSEDIWLVEFYAPWCGHCKNLAPHWAKAATELKGKVKLGALDATVHTVTANEYGVQGYPTIKYFGPGRKDSSSAQDYTGGRTSSDIVTWAMEKLAENVPAPEVKQIINEETLKEACEEHPLCVVSVLPHILDCQSECRNSYLDTLKKMGEKYKQKMWGWIWAEAGSQPELESMLEIGGFGYPAMAVMNAKKMKYSILRGSFSVDGINEFLRDLSFGRGSTAPVKGAALPKINPTEEWDGKDGELPPEEDIDLSDVDLDDIGTKDEL

		aegypti GN=AAEL010065 PE=2 SV=1	
LMI_GLEAN_10187125	COG0526	glycerol ether metabolic process; Biological Process GO:0009055; electron carrier activity; Molecular Function GO:0015035; protein disulfide oxidoreductase activity; Molecular Function GO:0045454; cell redox homeostasis; Biological Process IPR005746; Thioredoxin IPR012336; Thioredoxin-like fold IPR013766; Thioredoxin domain IPR017936; Thioredoxin-like AGAP000044-PA; K13984 thioredoxin domain-containing protein 5 TXND5_MOUSE Thioredoxin domain-containing protein 5 OS=Mus musculus GN=Txndc5 PE=1 SV=2 Q1HR90_AEDAE Thioredoxin/protein disulfide isomerase OS=Aedes aegypti	MFYAPWCGHCKNLAPTWDQLAEKYHKDDSVGLTVGKVDCVKETALCSEQDVTGYPTLKFFKAGENEGVKYRGTRSLKAFSTFIEEQLGLTVQGSDTQPKVPEPVSGLIELTEDTFEDHVAKGRHFVKFYAPWCGHCQVAAEHSLEPGDWKVEAGMAHDQNIAFLWSCRGAPVKSQPVVQLYKDDISNQSMVSHLTMAEEITAEILWIKLAPTWDELAKSLEHDTSVVIAKIDCTAHRSVCNDFEIKGYPTLLWIEDGKKVEKYQGPRTIEELKAFVSKMAGSDASHAAKSSDTQDSSPVVSFSGENFEHGIARGITFVKFFAPWCGHCKRLAPTWEELGKKFASNSRVKIVKVDCTLNSNKDLCNDQEVDGFPSLFLYKDGKRIAEYNGSRTLDDLYEFVSKHMPHDEL

		PE=2 SV=1	
LMI_GLEAN_10 098921	COG0526	cell redox homeostasis; Biological Process IPR012336; Thioredoxin-like fold IPR013766; Thioredoxin domain IPR017936; Thioredoxin-like thioredoxin 2; K03671 thioredoxin 1 THIOM_RAT Thioredoxin, mitochondrial OS=Rattus norvegicus GN=Txn2 PE=2 SV=1 B0XDN0_CULQU Putative uncharacterized protein OS=Culex quinquefasciatus GN=CpipJ_CPIJ017625 PE=4 SV=1	MLALLRPTWRPLARAFSRSPATAKALTVKNQEEFLNKVMHSDLPVIVNFHAEWCEPCKILSPRLKELVEPLEDIVLAVVDVENHVDLVHTFEVKAVPAVIAIRNGIVVDKFIGLVDGEMIDKLIAELSRGSKPESGGSNSKQ
LMI_GLEAN_10 172979	COG5541	transport; Biological Process IPR011012; Longin-like IPR022775; AP complex, mu/sigma subunit NA COPZ1_BOVIN Coatomer subunit zeta-1 OS=Bos taurus GN=COPZ1 PE=1 SV=2 C4WUX3_ACYPI	EPSLYTVKGIAILDNDGNRILAKYYDKSIFPSAKEQKTFEKNLFTKTHRANAEIIMLDGLTCVYRSNVDLFFYVMGSSQENELILVSVLNCLYDAVSQILRKNVEKRVVLENLDIVMLALDEICDGGIIMEADPSALVSRVALRVDDIPLGDQTVSQV

		ACYPI002203 protein OS=Acyrthosiphon pisum GN=ACYPI008765 PE=2 SV=1	
LMI_gi_1887439<br>1	COG0625	translation elongation factor-1 gamma [Locusta migratoria]	TLYTYPENFRAYKVLIAAQYAGSNVKVAPGFVFGETNKTEAFLKKFPLGK<br>VPAFETSDGHYLTESNAIAYYVANAQLRGQSDLERAQVLQWLGFADSEIL<br>PASCAWVFPCIGILQFNKQTTERAKEDVKAALGTLNKHLLTRTFLVGERIS<br>LADICVACTLLHLYQYVLDPAFRKPYQNVNRWFNTIINQSQVKAVIGEFKL<br>CEKMAEFDPKKFAEFQAATKQQQPGTAKKEKKQKGQGEGQQQPKEKEQ<br>EERGTRPRRGDGRGGPGRREPKAKDPLEALPKGTFNMDDFKRFYSNEDES<br>KSIPYFWEKFDPEHYSIWLGEYKYNDELQKVFMSCNLISGMYQRLDKMR<br>KNAFASMCLFGSDNDSSISGIWVWRGQDLAFDLSPDWQVDYESYQWTKL<br>DPTKEETKELVKQYLSWTGTDKQGRKFNQGKIFK
LMI_GLEAN_10<br>156223	COG0464	Transitional endoplasmic reticulum ATPase TER94 [Zootermopsis nevadensis]	MADGRNPDDLATAILRKKDRPNRLIVEEAVSDDNSVVALSQAKMDELQL<br>FRGDSVLLKGKRRKETVCIVLSDDSCPDEKIRMNRVVRNNLRVRLSDIVSV<br>QSCPDVKYGKRIHVLPIDDTVEGLTGNLFEVYLKPYFLEAYRPIHKDDTFIV<br>RGGMRAVEFKVVETDPSPYCIVAPDTVIHCEGDPIKREEEEEALNAVGYD<br>DIGGCRKQLAQIKEMVELPLRHPSLFKAIGVKPPRGILLYGPPGTGKTLIAR<br>AVANETGAFFFLINGPEIMSKLAGESESNLRKAFEEADKNAPAIIFIDELDAI<br>APKREKTHGEVERRIVSQLLTLMDGLKQSSHVIVMAATNRPNSIDGALRRF<br>GRFDREIDIGIPDATGRLEILRIHTKNMKLADDVDLEQIAAETHGHVGADL<br>ASLCSEAALQQIREKMDLIDLEDDQIDAEVLSSLAVTMDDFRYAMSKSSPS<br>ALRETVVEVPNVTWEDIGGLENVKRELQELVQYPVEHPDKFLKFGMQPSR<br>GVLFYGPPGCGKTLLAKAIANECQANFISVKGPELLTMWFGESEANVRDV<br>FDKARAAAPCVLFFDELDSIAKSRGGNVGDAGGAADRVINQILTEMDGM<br>GAKKNVFIIGATNRPDIIDPAILRPGRLDQLIYIPLPDEKSREAIFRANLRKSP

			VAKDVDLTYIAKVTHGYSGADLTEICQRACKLAIRQSIEAEVRRERERANNPNLAMDMDEEDPVPEITRAHFEEAMRFARRSVSDNDIRKYEMFAQTLQQSRGFGTNFRFPSSNPGNPSQGGNSGSQPGTFQEDGDDDLYS
LMI_GLEAN_10117853	COG0625	Thioredoxin-like fold	MPSIILYGNELSPPSRAAKMIAEKLGVDVDFKRTYPIKGECKKPEYLKINPMHTIPTIIDGPFTLSDSHAIVAYLVDRFGKNDSLYPKDIQKRSKVNERLCFDIALFTKVLKFVVGPLLRTHEPTEELRNDCIDGLETVERFLSASKFIAGDDLTVADYCYYCTITFVDV
LMI_GLEAN_10151702	COG5044	Rab geranylgeranyltransferase activity; Molecular Function GO:0005968; Rab-protein geranylgeranyltransferase complex; Cellular Component GO:0006886; intracellular protein transport; Biological Process GO:0043087; regulation of GTPase activity; Biological Process IPR001738; Rab escort (choroideraemia) protein IPR002005; Rab GTPase activator IPR018203; GDP dissociation inhibitor NA RAE2_MOUSE Rab proteins geranylgeranyltransferase component A 2 OS=Mus musculus GN=Chml PE=2	MEDDLPTEYDVIVVGTGMTESIVAAAASRVGKKVLHLDSNEYYGGLWASFNFEGFQKWMEECRQPAKASDDVELPDGILRNGEAPVKAGNQFSVITNIEEKWYIVEEAADAVEGQSSSQHTQTDGAEDAKGQADEKESEQGDSKKTEEKQAPPPSKQWSQNRVRKEYRKFNLDLAPKLLFARGSLVELLISSNIARYAEFRSVTRVLTWLDGRLEPVPCSRADVFSTKNVSVVEKRLLMKLLTACVDQEDASKEFEGFQDKPFVDYLNSKKLTPNLMHYVIHAIAMCTEDTSCDDGVARTQRFLSSLGRYGNTPFLWPMYGSGELPQCFCRLCAVFGGLYHLKRAVQHLLIEDGKCKGVASGGRRLDSVNLVMGVGHAPAQYLAAAKPGGLSRGIFITDRSILPADKEALTLLRFPPVEGSKEPVTVIEIGPATNACPQGLYVVHMTCRQQKTAREDMQEVVKSLLHTEFDDGTVSRIDTDTQSTQTSHPGGEGDGETQEESTTRPAALKPQVLWSLYFNCPETSSCDLTTGVPSNVFLCSGPDLDLDFEHAVAEAKSIFMKMYPDCEFLPRAPDPEEIVLEGDDAPGPAFESGEAVDGEAEGEVGGGGAPPAVEGS

		SV=2<br>C3YZ95_BRAFL Putative uncharacterized protein OS=Branchiostoma floridae GN=BRAFLDRAFT_123298 PE=4 SV=1	
LMI_GLEAN_10053226	COG5044	Rab GDP dissociation inhibitor alpha [Zootermopsis nevadensis] Sequence ID: gb|KDR21130.1|	MDEEYDAIVLGTGLKECIISGMLSVSGKKVLHVDRNKYYGGESASITPLEELFARFGAPAPGETYGRGRDWNVDLIPKFLMANGSLVKLLIHTGVTRYLEFKSVEGSYVYKGGKISKVPVDQKEALASDLMGMFEKRRFRNFLVYVQDLKEDDPKTWKETDPNTCTMQQLYDKFGLDKNTQDFTGHALALYRDDDYLNRSAIETIRRIKLYSDSLSRYGKSPYLYPMYGLGELPQGFARLSAIYGGTYMLDKPIDEIVLENGRVVGVRSGEEVARCKQVYCDPSYVPDRVKKTGQVIRCICLMDHPVPNTRDALSTQIIIPQKQVGRKSDIYVSLVSFTHQVAAKGWFIAMVSTTVETDNPEAEIRPGLDLLGPIRQKFVSVSDYYEPTDLGSESQIFISTSYDATTHFETTCLDVLDIFRRGTGEDFDFSKVKLDLGDEEQ
LMI_GLEAN_10107249	COG2518	Protein-L-isoaspartate(D-aspartate) O-methyltransferase [Zootermopsis nevadensis]	MSLNKLRYKHYIRSAYRTTAHLVALPPTEAASQQHTLRVYFQVQQWLGNEKDPEQWGWKKTKDGLQPVPTLQPPAPETYFLLPAPMTHFFALLAVGCVFYLSFNRQFAMAWRSHGKNNSDMVRNLRANGIIRSKIVEDVMEQVDRGKYAKYNPYMDAPQGIGYGVTISAPHMHAHALELLKDHLKEGERALDVGSGSGYLTVCMALMVGETGRAVGIDHIPELVEFSRNNVRADKPELLESGRVKLVVGDGRLGWKEDAPYNAIHVGAAAATLPQALIDQLKPGGRLVVPVGPEGGNQQLEQVDKLPDGTVRRTPLMGVVYVPLTDKDHQWPGR
LMI_GLEAN_10085130	COG1730	prefoldin subunit 5 [Culex quinquefasciatus]	MASKKQPQMQEIDLTKLNLQQLTQLKQQLDQELSLFQDSLQTLKLAQNKFQDSLESLDPVNSSLKDKPILVPLTSSIYVPGTVIDHEKVLIDIGTGYYIEKDIDGAKDYFKRKVDFVTQQLEKLQGVMIEKAKVREEFVAVLRKMPAGKLYFPRESLMRCENEECVYKGTLHSEADCENVPKLDGVSHPEVTNQPARSGLSQR

			VGLLVVRSSKVKRVMEPLREMAAKEGKKANVHSLCIPGGVIPDVERVLPDAMNSTGCSQLQAVAHIGTNDVCCFGSEEILSGFERLSELLQIQLMPVESVASNQGLTRTTEEKII
LMI_GLEAN_10101934	COG5640	PREDICTED: venom protease-like [Apis dorsata]	GCGYSNVTHVRIIGGKKAELGAWPWLTALVYRGNTGPRVLCGGALITERHVLTAAHCVYNRSDLYKVRIGDLNLLADDDGATPIESEIIERRMHEGYRKGRFENDIAILKLADKIPFSFYLHPICLPVAEDLRSNAFVRYNPFIAGWGAIEFRGPSSADLLETQLPVVEPETCEKAYERFSSAVIDERVLCAGFTKGGKDACQGDSGGPMMLPQGRIFYIIGVVSYGHNCAEPGVPGVYTRVTRFLDWIQNNLD
LMI_GLEAN_10164451	COG5272	PREDICTED: UV excision repair protein RAD23 homolog B-like isoform 3 [Bombus terrestris]	MLITFKTLQMKTFVIDFDINLTVKELKEKIRSEKGADYPVENQKLIYAGKLLNDEDIVSKYDVDEKKFIVVMVSKPKPPVVENGEKSTTATVTPPTENSTTVSSAQTGTATPSTTTTTAGTSTPTTTASSSITTSADAATTTTATRTTAASTVTNTSASASEPLSTTAQSASSLTSSTNESSRSSSGESPRVPVPTTGSGNTHPGGGDGPHVLPSSGQAVARPAGREEREPTSGGGNEASGQSARTDTERERSASTEGRRGSGSSANPPETTGGLSDDDDNDLEATLISEEQFRDYVRNIMDLGYSREQVETALRASYNNPDRAVEYLLSGIPDQVAEGVGNLPPRSDRQRRVPEEPIICGAIRNSLFPLLCTVAGQSSCEESVYKTLMLLSDNSKPAAVAEWSAVETALRASYNNPDRAVEYLLSGIPDQVAEGVGNLPPRSDRQRRVPDDPLAFLRNQPQFQQMRQVIQQNPQLLNAVFQQIGQTNPALLQLISQNQEAFVRMLNEPPVDSLELAKGPCTEYLEIGILPGASLIYYALRPEARNELLKALGFPEHLVIQAYFACEKNENLAANFLLSQNLDD
LMI_GLEAN_10134478	COG0545	PREDICTED: FK506-binding protein 2-like [Apis mellifera]	ESDFKVEKLFVPDECTEKSKTGDMLTMHYRGTLDDGKQFDSSHDRDQPFSFQLGVGQVIKGWDQGLVDMCVGERRRLVIPPELAYGDRGAGAVIPGGATLTFEVELLSIGDSPPPVNVFKEIDADKDQNLSRDEVSEYLKKQMVAAEEGGDSEEVKRMLEDHDKLVDEIFQHEDKDKNGLISHDEFSGPKHDEL
LMI_GLEAN_10	COG0545	PREDICTED: 12 kDa	MDVSPVSHEGADATEEEVFQTLAPVCPANCTRYEQWHRHAMTYTAAAK

112433		FK506-binding protein-like [Apis florea]	WQPTDQPTWPQPTPPATMMHRQRTDVWRTKTTHHCVSTVGTLDMSYATAERECTLENGVKFDSSRDRGVPFKFRLGKGEVIKGWDQGVAQMCVGQRARLTCSPDYAYGSRGHPGIYPFSAIFRMFEIRLQFSSKITTLFFKKNCFEMVEGIAYPSHKDGKNMQL
LMI_GLEAN_10065008	COG0225	oxidoreductase activity, acting on a sulfur group of donors, disulfide as acceptor; Molecular Function GO:0019538; protein metabolic process; Biological Process GO:0055114; oxidation-reduction process; Biological Process IPR002569; Peptide methionine sulphoxide reductase MsrA EIP28, putative (EC:1.8.4.11); K07304 peptide-methionine (S)-S-oxide reductase [EC:1.8.4.11] MSRA_DROME Peptide methionine sulfoxide reductase OS=Drosophila melanogaster GN=Eip71CD PE=2 SV=2 C4WT37_ACYPI ACYPI005402 protein OS=Acyrthosiphon pisum	MQEQPRAKVQGGAVCAQADASRQASHILVAEEEEKTVPEVSHLAPPCQVDVAVVEEERRRWLPWQRHRIVRMKRTVERETAEESHDVPATRVNVEALRSVNPKFAERNRLAPPNGPKFFYPQTQVYSSTKDMAMATANILHDIDVPTKKATFGMGCFWGPDTLYGTLKGVIRTRVGYSGGTKLNPTYRDLGDHTECIDIDYDPNEISFDKLLEEFWKNHDPTYRAKKQYTSLIFYHDDDQKQIAEKSFKEEEEKKGQKGKFVTKILPAVEFYNAEDYHQKYRLQQHTQLVQWLGLTSGKKLISSHAAARINGYVVGMGGVKMFEEDVNRLGLTPEIADYVRKLVTKYEGRGMIC

		GN=ACYPI005402 PE=2 SV=1	
LMI_GLEAN_10034589	COG1222	nucleotide binding; Molecular Function GO:0005524; ATP binding; Molecular Function GO:0017111; nucleoside-triphosphatase activity; Molecular Function IPR001270; Chaperonin ClpA/B IPR003593; ATPase, AAA+ type, core IPR003959; ATPase, AAA-type, core<br>26S protease regulatory subunit 6B, putative (EC:3.6.4.3); K03063 26S proteasome regulatory subunit T3<br>PRS6B_MANSE 26S protease regulatory subunit 6B OS=Manduca sexta PE=2 SV=1<br>Q172T1_AEDAE 26S protease regulatory subunit 6b OS=Aedes aegypti GN=AAEL007297 PE=3 SV=1	FVPLKLEDTTQDAKQTTTGALGIGASDDTDFEDLYTKYKKLQRQLEFLAVQEEYIKDEQRNLKKEYLHAQEEVKRIQSVPLVIGQFLEAVDQNTGIVGSTTGSNYYVRILSTIDRELLKPSASVALHKHSNALVDVLPPEADSSISMLQADEKPDVTYSDIGGMDMQKQEIREAVELPLTHFELYKQIGIDPPRGVLMYGPPGCGKTMLAKAVAHHTTAAFIRVVGSEFVQKYLGEGPRMVRDVFRLAKENSPAIIFIDEIDAIATKRFDAQTGADREVQRILLELLNQMDGFDQTTNVKVIMATNRADTLDPALLRPGRLDRKIEFPLPDRRQKRLIFSTITSKMNLSEEVDLEDYVARPDRISGADINAICQEAGMHAVRENRYIVLTKDFEKGYKNNIKKDESEHEFYK
LMI_GLEAN_10146574	COG5193	La protein-like protein [Zootermopsis nevadensis]	MVPILVGGVSGAACCGSRDIGGSSESDLETDMKDLSVGSGSVSGVVASIMAEENDVSVSTEEKADKSENETTGGDKSENEKAGEDKNEVENDAVSSETV

		Sequence ID: gb|KDR22239.1|	KETEEAPKNGDLQLLEKQIIRQVEYYFGDINLPRDRFLQEQIKLDDGWVPLTVMLNFQRLSNLTKDTTMIANALEKSSLIEISDDRAKIRRSPDQPLPVWNEERRKELMTRTLYLKGFPVQDTTLDKLLDFFAQHSTVENVQMRSYKDKATNKFVFKGSVFATFPTKEKAEEFLGKEIKYEGNDLIKKWQSVYVEEKKKEREEAKAKRKGGAQNEADKKALPKGAILHLKGLTSDISREQISEKLSEYEADIAYISYDKGDEEGWIRLQGENSTKAVLEKIGETTLEIGNVKIEVRALEGEEEEQFLKKTMEDIQKRRFHYNQNKRGRRGGGRGGFRGDRKRKQASAESTDEPPSKVSAKTE
LMI_GLEAN_10075572	COG0694	iron ion binding; Molecular Function IPR014824; NIF system FeS cluster assembly, NifU-like scaffold, N-terminal NV13733; similar to protein phosphatase 2a; K04382 protein phosphatase 2 (formerly 2A), catalytic subunit [EC:3.1.3.16] NFU1_DROWI NFU1 iron-sulfur cluster scaffold homolog, mitochondrial OS=Drosophila willistoni GN=GK25604 PE=3 SV=1 B4L310_DROMO GI14603 OS=Drosophila mojavensis GN=GI14603 PE=4 SV=1	RTMFIQTQETPNPNCLKFLPGVEVLGPGQTRDFPNGQSAHCSPLGRLLFRIEGVKSVFYGPDFITVTKEKKHIHSHNHLDNYTYATNMSSHTSATSIWTVGEVRSGTGRMLTSLFTVSCYVSSIIDILIRFSIVIYFILLQGFEDGIVKLKMQGSCTGCPSSSVTLRNGVQNMLQFYIPEVLGVEQVEDEIEIMTKKEFEEFEK
LMI_GLEAN_10	COG1025	Insulin-degrading enzyme	IKRRVEDIIKSTEDKRLYRGLVLQNDMKVLLISDPTTDKSAAALDVNVGH

161004		[Zootermopsis nevadensis]	MSDPDHLPGLAHFCEHMLFLGTEKYPSENEYSKYLSEHGGGSNACTFSDH TTYYFDVVPEFLPGALDRFSQFFLSPLFTESATEREVNAVNSEHEKNISNDS WRLDQLDKSTAKQEHPYAKFGTGNKYTLDTLPKEKGINVREELLKFHET WYSSNIMSLSVLGKETLDDLEKMACQLFSNVKNKNVVAPEWPEHPFGRE QVRVRGYIVPVKDIRNLNITFPVPDLHQYYKSGPGHYLSHLIGHEGPGSLLS HLKAKGWCNSLIGGYRTGSRGFGFFVINVDLTEEGIEYVDNIVELVFQYIN MLKKEGPKQWIFEEYRDIMAMHFRFKDKEMPQNHASSTAQNLNEYPLEE VLSGHYLITEWKPELINMVLDYLTPENIRIAVVGRKFEPLVDKTEKWYGTK YRLEEIPQETLKRWQETDLCEDLKLPPKNEFIPTDFDLLPREDNALEHPTVI QNTPLTRVWFKQDDEFLLPKANLSFEFVSPLAYLDPVSCNMTYMFVLLFK DALNEYAYAAELAGLRWELSNTKYGMILGIGGYNNKQHVLLDKIVEKLT NFKIDPKRFDILKEHYIRGLKNFEAEQPYQHAVYYSAVLLAEQAWTKEEL LAATHELTLQNVEAFIPQILSKMHIECLIHGNADRQRALELVHLVEDRLLSS VSLUPLLPRQLLRHRELQLVDGCNFVFEVNNALHRSSCIEVYYQCNLQSTE TNMLLELLVKIINEPCFNILRTKEQLGYIVSSGIRRSNGVQGLRVIVQSDRHP EYVDQRVETFIASMETYIEQMPDEEFERHKEALAAQRLEKPKRLSARSSRF WAEITSQQYNFDRVNIEVAYLRTLTKTDILNFYKELIHHSSPKRHKLAVYV VSTAEGGAGNMPRPSDPDAVIDGMLPPPPFYKKPTRIQDITEFKSSQGLFPL VKPYINIPSK
LMI_GLEAN_10 084257	COG5656	Importin-7 [Zootermopsis nevadensis]	MLSDVDMPVRQAGVIYLKNLVTQNWADREVEPGQPLPFSIHEQDRAMIR DAIVDAVVHAPDIIRVQLAVCVSNIVKHDFPGRWTQIVDKISIYLQNPDAS GWSGALLCLYQLVKIFEYKKREERGPLNEAMNLLFPMIYQLCVRLLPDQS EQSVLLQKQILKIYFALTQYSLPLELISREVFSQWMEICRAVVDRPVPEQTN AVDEDDRVDLPWWKAKKWALHILHRMFERYGSPGNVTKEYKEFAEWYL KTFSAGILEVLLKVLDHYRRKIYVSPRVVQLTLNYINLGIGHAFSWKFLKP HMFAIVQDVIFPALSYSQADEELWNTDPYEYIRVKFDIFEDFVSPVTAAQT

			LLHTACKKRKDMLQKTMHFIMQVLTSPNADPRQKDGALHMVGTLADILI KKNMYKTEMDSMLCQYVFPEFASPRGHMRARACWVLHYFSEIKFKQEA VLAEAIRLTTNALLNDQDLPVKVEAGIALQMLLNAQEKSEKYVEPQIKQIT LELLRIIRETENDDLTSVMQKVVCTFTEQLMPIAVEICQHLATTFSQVLETD EGSDEKAITAMSLLNTIETLLTVMEEQPEIMAQLQPTVLQVIGHIFSQSVME FYEEALSLVYDLTSKNISPDMWKVLELMYQVFQKDGFDYFTDMMPALHN YVTVDTATFLSNENYILAMYNMCKAVLTGDSGEDPECHAAKLLEVIILQC KGRIDQCIPSFVELVLQRLTREVKTSELRTMCLQVVIAALYYNPQLLFETLE KLQMVVSPTESITSHFVKQWIHDTDCFLGLHDRKLCVLGLCTLLSTTGAA RSVALPNECASQIIPSLILLFDGLRRAYVAKAQEGEEEAEDEEDETDIDQEIL SSDEDDIDEAHQEYLENIEDKVHKANKAFTITTSIQDDDDSDSDDEFEANE ETALESYTTPLDEENCEVDEYIVFKEVMQNIQASDPTWYQILTTDLTTEQQ KALQEIMVLADQRKAAAESKRIEQSGGYVFNQQTVPTSFNFGGSPL
LMI_GLEAN_10 119170	COG0443	Hypoxia up-regulated protein 1 [Zootermopsis nevadensis]	MEVLRACADDRMRNAADKLTQDRNWFENVYLSPAHTGCSEKGSLISKLN IPKTKIDRVPVMRLLVAVWVVLSLLHVVRHADGIAVMSVDLGSEWMKIAI VSPGVPMEIVLNKESKRKTPATIAFRDGERTIGEDALVVGVRFPQNSYSYL LDLLGKSVDNPLVQLFQKRFPYYKIEAEPERGTVVFRHDSETAYSVEELLG QLLQSARQTAQVSAGQPVKEAVLTVPGFFNQAERRALLQAAELAGLKVL QLINDYTAVALNYGIFRRKDFNETAQYIMFYDMGASSTTATVVSYQIVKT KERGFVETNPQLAVLGVGYDRTLGGLEMQLRLRDYLAKKFNEQGKTKSD VFGSPRALAKLFKEAGRLKNVLSANAEHYAQDPVEERDSMVMEQVEGLL DDVDFKLLVTRDDFEEMCADLFDRIQKPVEQALKTSGLDMNVISQVILVG AGTRVPKVQERLAAAVGLELGKSINTDEAAALGAVYKAADLSTGFKVKK FLTKDAVIFPVQVMFERELENGAGTKQIRRILFGPMNPYPQKKVLTFNKH MNDFSFYVGYGDLDHLPEHEIHKEKRMWSVYETFIRSPKKLVLGVSREIE MSPMTVWKVLQLKLSLKPSKLEVMFERELENGAGTKQIRRILFGPMNPYP

			QKKVLTFNKHMNDFSFYVGYGDLDHLPEHEIQALSTLNLSRVDLTGVSEA LSKHKDAHGVESKGIKAHFSMDESGVLLLSGVELALEKTVSPEEQEAEAE GTLSKIGSTISKLFTSSDEKKDEQKEEKKDEKPEDKASEDTSGKEGQAGGD GAGQEGDGKDSDGKEQKSGDDKPAGEKAEAAEKTAKEGADKADAKNVT KPAEKKPKIVMLKEPINAAEERSGVPPLNDEQFQVSLKKLESLNEFDEQRK RRESALNALESFVFDAQNKLYADEYQAAGTEEELEKIKQSCSEVSDWLYE DGSEADAQAYEDKLSELKQLTRPLFRRVWEHQERPTALKSLAEILEMSSK FLDGAKNGTEIFTTVEIETLEKVIKETKEWKQKMEEDQAQLKLSETPKLTV KSITDKISLLDREVKYLINKAKIWKPKKKDDTKEKDDASSGTKDKNETTTD KKTESEQPVPDSDDDGGERPDPERETPLPAEDVSGGPEETALPEPPEPELPT STDVPLAADRGGVSSGXXXXXXXAALRTSATDDHKISRRFDDRFLSMMTI
LMI_GLEAN_10 061914	COG0545	hypothetical protein YQE_06527, partial [Dendroctonus ponderosae]	MRRSSDEDEFLLLLTVASKKRRKWVHDINTKREEYALVLEPGKKYAQTVS KPFHISMASLDVVHSENELVTVNVNFQNAEFILCNLQKNKILQTSLDLNFE AGDRIAFYTSGKGRVHLTGYLIDDDDVDDDLDIDAEAEESEEDVTPQKNAI KANKQEKRKSVGQTPGKPVLKKSKMDSEDEDGDDNGDDFDDDGDSDLE SLGDSDEEMEVESEEDDGEEDEDSELEETPPQKHQQGKKKEKQSAQKQLQ DKQTNTPNEMKKKKNKGHDAATPNTPTVQANGTPETQSGKKNKKKGGE TPGDKGANTPKPPQAGSPQTPQKKLLEGGVAVEDTVVGSGPVAKPGRFVT VYYTGRLKQNNKKFDETVQGPGFKFRLGKGEVIKGWDIGVTGMKVGGK RKLIIPPHMADGKIFNRDGLAGQYFRAVLLIVSQRKKKVKKVAQISEQDSR YGAKGSPPVIPPNSALVFEVELKNDPKPACRRASGSSTITDATSGKPSQPLT PKGQREGQKKPKEEEDANEVSSVPRKKRSVTSSASEDEVEILASPEDLAIL GSPEPMMLDPEESAKLAAGAHAE
LMI_GLEAN_10 147494	COG1222	hypothetical protein L798_04485, partial [Zootermopsis nevadensis]	MRRFRPLGYSTEAPLGSQGQVPAIVPNVPNPGVATRLAAPVAVFVPVSKV VAMEVDDRPTKGEGFRQYYITKIEELQLVVAEKSQNLRRLQAQRNELNAK VRMLREELQLLQEQGSYVGEVVKPMDKKKVLVKVHPEGKFVVDLDKNI

			DINDVTANSRVALRNESYTLHKILPNKVDPLVSLMMVEKVPDSTYEMVG GLDKQIKEIKEVIELPVKHPELFDALGIAQPKGVLLYGPPGTGKTLLARAV AHHTECTFIRVSGSELVQKFIGEGSRMVRELFVMAREHAPSIIFMDEIDSIGS SRIESGSGGDSEVQRTMLELLNQLDGFEATKNIKVIMATNRIDILDPALLRP GRIDRKIEFPPPNEEARLDILKIHSRKMNLTRGCXAGMYALRERRVHVTQE DFEMAVAKVMQKDSEKNMSIKKLWK
LMI_GLEAN_10 191702	COG5193	hypothetical protein L798_02593 [Zootermopsis nevadensis]	DQKKKDPKQKWVPLDIDMKAPGKRDHSPKFRGSDNYSHHDNENHWHHF DASERGYQPHYRGGRGSAPRYRGRGMRGNVRGIGRGAGGFRRSQSQEID YPDFPTEYSQVQTGFMTPYMGTFYYGNNSYMHLDDLTLKEYIRKQIEYYF SEENLLRDFFLRRKMDSDGYLPINLIASFHRVQALSTDLKVVMDAIRDSEV LEITDFKVRTKDDPTRWPIQDPTGAVMTSPAVNMTSTPPVPSSAHLVDTLN PDVPEFVPENVRLQVESRGQADGQEADSDLEGEKSETEEVVKSKDENTCT AKPTEDIPRERMCAEEKKSDEKRERDQFKDREDLNFQFDEELDNPVPSGR HNTFTDWSEDESDYELSDREINKILIVTQTSQSSRYPKHEGYDRTGDWTTR VKITQDLAQAINDGLHYYEEDLWREHEWVQQTGSYKTVNVITQEDFEKM TPRLPKKMNPEVPPPPPPSLPAPTGRPSKDTAHRRRAARFFPVVKDEHPVD PLTPRKRKTRHGSNPPVEHHVGWIMDVREHRPRTTSIGSSTGTSPSEGFLT GGTPTSLPAFQHPSHSLLKENNFTQEVYHKYHSRCLKERNRLGIGQSQEM NTLFRFWSFFLRENFNRNMYKEFRSLAVEDAHEGYRYGLECLFRYYSYGL EKKFRPDVYQDFQEETIADYESGQLYGLEKFWAFLKYYKHSANLIVNPKL KEYLSKFKSVEDFRVVEVSYPIYYNYILQTSIDVY
LMI_GLEAN_10 106309	COG0450	hypothetical protein DAPPUDRAFT_305931 [Daphnia pulex] Sequence ID: gb|EFX63892.1|	MAGLIKCIATRVLFSNAVVNIHFSLLRAVEAECLKESAASFAPRVQAPAPD FHGTAVVDNSFKEIKLADFKGKYLVLFFYPLDFTFVCPTEIIAFHERINEFKE LNTEVVGVSTDSQFSHLAWINTPRKIGGLGALSFPLLADFKKNISRDYGVL VESEGIALRGLFIIDPEGTVRQITINDLPVGRSVDETLRLIKAFQFVEKHGEV CPANWKPDSPTIKPDPVGSRKYFDKVN

LMI_GLEAN_10166914	COG4870	homologue of Sarcophaga 26,29kDa proteinase [Periplaneta americana]	FAVYCASPPNFSDSYSVRGTLYIPYAEIEEPFYAWFDLKLGASRIDYYGDMVKTFQLSQSSKYGTSIKVAPVSTDTQLNKQTCLQVNGTDKFKIEPQAVLPDLSDFQLIGKEVVNGMSCEKWRFVDKIGAKVNKYTMWIYYKGSEQPPPKNMVAIPVRYEMKGYNSLLGSHYDHYYLEYDEYSFSPPNPNVFQIPGNMTCTSFPGPGSKHIYTFNPMKEFIHNYDKHVDKAFEEFKHIHKKSYVSDVEHSQRKELFRQNMRFIHSKNRANVGYTLAVNHLADRTDLELQALRGRKYTPGYNGGQPFPYNVSVETKDGLPSTFDWRLYGAVTPVKDQSVCGSCWSFGTTGAIEGAFFVAHGYQVRLSQQALIDCSWGYGNNGCDGGEDFRSYQWMMKHGGLPTEDEYGPYLGQDGYCHVDNVTLTAKITGYVNVTPDDANALKVALLKHGPISVAIDASHKSFTFYSNGVYFEPKCGKTPDELDHAVLLVGYGALNDKKYWLVKNSWSTYWGNDGYVLMSPKENNCGVMTSPTYVTM
LMI_gi_241997148	COG0326	endoplasmin [Locusta migratoria]	MKYFLLCLLGVLIFSGTCYAEKEDEGTTVEADLGSSREASRTDDEVVQREEEAIKLDGLNVAQLKELREKAEKFTFQAEVNRMMKLIINSLYRNKEIFLRELISNASDALDKIRLLSLTDNSALSATSDLDIRIKADKENHILHITDTGIGMTKNDLVNNLGTIAKSGTADFLSKMQDATTSAQDLNDMIGQFGVGFYSSFLVADRVVVTTKHNDDKQYIWESDAGSFSIVEDPRGDTLKRGTQVSLHLKEEAFDFVEQDTIKNLVKKYSQFINFPIYLWTSKKDFEKHCTDTSGIRQKVNTHLYLQIKLMTVMALDLWKPADVPDDEYNEFYKSLTKDTKNPLTKIHFIAEGEVTFKSLLFVPQTQPGESFNRYGTKTDNIKLYVRRVFITDEFNDMMPNYLNFVQGVVDSDDLPLNVSRETLQQHKLIKVIKKKLVRKALDMFKKIEKKDYEKFWKEYSTNIKLGVIEDPSNRTRLAKLLMFHSSNGPEMTSLSDYVSRMKEKQEQIFYIAGANRKEVENSPFVERLLKKGYEVLYLTEAVDEYCISALPEFDGKKFQNVAKEGFSLSGDSKSKEKLENIKKHYEPLLNWLNDKVLKDQISKATISERLSGSPCALVASMFGWTGNMERLAISNAHQKADDPQRSYYLNQKKTLEINPRHPLIKELLKRVEEDPSDPTAKDMALMMFRTATLRSGYMLRDTTDFAESIEVMMRKTLGIPESEELEEEDEDTEEGDDEQKKSEDSENEVDDKDGENED

			HDEL
LMI_GLEAN_10 182911	COG0526	cell redox homeostasis; Biological Process IPR012336; Thioredoxin-like fold IPR013766; Thioredoxin domain IPR017936; Thioredoxin-like	VIEEVTAKQLERLLNEKDFVAVFWYARSCITCDKVLEELEKIDDDTDIFGV DFVKINDKRLAKQYGIKNFPALTYFREREPIIYEGDLMDEENVLDFLTSLEA MDLPDRIEEVNARILEKIVQDTEYVAVLFCPDTKTCPSTGSNRPECRKCAK ALLELENIDDEADQLGIGFVKIADESLAEEYNLGSLPALVYYRNQIPIIYEG DLTKEEDVLEWLVQNKSTGDEEDVIEDVTSKTLETLISSIDNLVVLFYDND DEESMQVLTDLETIDDDCDKHGIQFVKIDDDTTAKEFGIDDVPAIVYFEKGI PNVYDGTLENEDEILEWLVDQLEKDEIEDVTDEMLDRLIKDGKTVAVLFY DNNDRKSQRALNELENIDDECDQLGIVFVKIDDPEEAKEYGVEKIPALIYFE GGIPTYYEGNLEEEEKVLSWLEHQIKTDEIEDVTDEMLDMIISKMTHVAVL FYDKDQKKSQKVLQELENIDDECDQNNIAFVKIDNDEEAKEYGIDTLPTIV FFEKGIPHVYEGDLTREEELLGWLLHQKRHHEIPEVTDEMLEILIEKRPYLA VLFYDKDDKQDIRILNELENIDDDLDREGIVIVRIDNDAEAKEYGIDHLPTL VYFEDKIPALYEGDLLNEEEVLAWLIEQKNTATIEEVTDEILTDLIEEHEYV VVYFSGKCEEGEECDNILDELENIDDELDEAGIIFVTTEDLSIAKKNGIKTFP SLVFFRNKDPMIYKGDLDDEDEVLAWLTDEDTLEIPGRIEEVNTRMLEKIL QDNQNVVVFFYQEGDKKSQKIISELENIDDECEEKDISFVKTSDEGIQKEYD LPTLPSLAFYRNRFRQIYTGDLMHEEAILDWVLELHESTPDVIESVDRKTL QMLINDVEHLAVFFYDDNCEKCEEILEELETIDDDTDKHGIQFVKSKDSKL ASEIGIFSFPALVYYETGVPIMYNGNLKNEDKVLHWLVEQKSNDGGDGDE DDDDDDDDEDDDDDDDDDSDINDRRIVVKGDGCFYIGLGGKSVVPNPSY EPYQCCPTKVKQGSRIAKATPTPVNKKFTPNKLTNPTSKPTNQPTSKDRSG ANVKDKPFAASTKPPSKSIPLSDKSEKVKKGKKIIDESNLLDETDVLDWML DQKNDESIEEVVRDKLFKYIETKEFLAVIFYTEDNPISPRVLRHMELIDDEA AEYGIKIVKCKDRLMAKKYGYRNPPGITYFRKGKYINYDGDIDDDEEVLD WLTNPENMELTDHIERVNRKMFQKIRQTSDYVAVFFYSNDCKQCDRVLA

			EIEHIDDEADGAGINFVKIDDKQMAKEYGVFALPGVVFFKMTSKEPVIYAGDLYNEEQLLNWLLTQKDPSGEVIEAVEGEELQKLIKESDSLAVYFWNQTLCELCNTKLYRKLRHKKETNQQAEHQGDSVDECDQCKIILEELENIDDDCDRHGITFIKTQ
LMI_GLEAN_10111079	COG0450	antioxidant activity; Molecular Function GO:0016491; oxidoreductase activity; Molecular Function GO:0045454; cell redox homeostasis; Biological Process GO:0051920; peroxiredoxin activity; Molecular Function IPR000866; Alkyl hydroperoxide reductase subunit C/ Thiol specific antioxidant IPR012336; Thioredoxin-like fold IPR017936; Thioredoxin-like IPR019479; Peroxiredoxin, C-terminal peroxiredoxins, prx-1, prx-2, prx-3 ; K03386 peroxiredoxin (alkyl hydroperoxide reductase subunit C) [EC:1.11.1.15] PRDX4_MOUSE	MVIRVYKCLLLCMIVNAVSALTEDSCHIYGGGDVYPQEIGNTVDHKLQWTKAVISKPAPEWEGTAVINGEFKALKLSDFKGKYLVFFFYPLDFTFVCPTEILAFNDNIEEFRKLNAEVVACSVDSPYTHLAWMNLPRKEGGLGKLKIPLLSDLTHKIARDYGVYLEDLGHALRGLFIIDPKGILRQITMNDLPVGRSVEEALRLVQAFQYTDKHGEVCPAGWKPGGDTIVPDPVQKTKFFKKQYKTES

		Peroxiredoxin-4 OS=Mus musculus GN=Prdx4 PE=1 SV=1 Q16G24_AEDAE Peroxiredoxins, prx-1, prx-2, prx-3 OS=Aedes aegypti GN=AAEL014548 PE=4 SV=1	
LMI_GLEAN_10 147897	COG0450	antioxidant activity; Molecular Function GO:0016491; oxidoreductase activity; Molecular Function GO:0045454; cell redox homeostasis; Biological Process GO:0051920; peroxiredoxin activity; Molecular Function IPR000866; Alkyl hydroperoxide reductase subunit C/ Thiol specific antioxidant IPR012336; Thioredoxin-like fold IPR017936; Thioredoxin-like IPR019479; Peroxiredoxin, C-terminal hypothetical protein ; K11188 peroxiredoxin 6, 1-Cys peroxiredoxin [EC:1.11.1.7	MVNLGDEFPNFKVNTTIGTIQFHDWLGDSWGVLFSHPADFTPVCTTELAR VASLMPEFEKRNVKVIALSCDSVESHAKWIEDIKSYGGQVGDRFPYPIIAD EGRDLAVQLGMLDPDERDKDGLPLTCRAVFVINPAKRLKLSILYPATTGR NFSEILRAIDSLQLTESKKVATPADWQSGGDCMVLPTLKEEEAANLFPKGV SVKPLPSGKSYLRVTPQP

		1.11.1.15 3.1.1.-] PRDX6_CHICK Peroxiredoxin-6 OS=Gallus gallus GN=PRDX6 PE=2 SV=3 A7RMT9_NEMVE Predicted protein OS=Nematostella vectensis GN=v1g234225 PE=4 SV=1	
LMI_GLEAN_10 169203	COG1397	ADP-ribosylation/Crystallin J1 NV14166; similar to ENSANGP00000022032; K11687 poly(ADP-ribose) glycohydrolase ARH3 [EC:3.2.1.143] ARHL2_MOUSE Poly(ADP-ribose) glycohydrolase ARH3 OS=Mus musculus GN=Adprhl2 PE=1 SV=1 Q7QCA6_ANOGA AGAP002530-PA OS=Anopheles gambiae GN=AGAP002530 PE=4 SV=3	MDLNTLTQKFRGCMLGSLLGDCLGAPYEGDSTVSKAVLQQYFDKIEGPPF KAPIKQYTDDTAMTKCVAESLIDCCGFDPQDMAKKFVKEFFASPRRGYGQ NVVRVFMKLKDQKCEDPFGPAKEQFDGAGSLGNGGAMRVAPVALFYHK DYPTLIEKARKSALLTHTHKLGRDGAILQAIAVYQGLQSNPSEGINAKVFIK DLMDKMHEIEDDDYGVEMEECRPYQKQLSVALDLLDADGSPDDIDTKVC DLLGTNIAALYSVPTAVFCFLRALQPIPDIETENPFRRTIQYAISLGGDTDTI ASMAGAIAGAFYGQSAISESLQRHCEAHVEVTDLADRLMAAYASSSDENE DHNKNPNNEIILNTAEELCLRAPHEETRENIVSRKRNKELGVMEIE
LMI_GLEAN_10 104676	COG0576	adenyl-nucleotide exchange factor activity; Molecular	MAAAGLRLGMKVACGFVESISGQASIIASRYPHSKYVPRTRQQWSTCSTA AYEGKKTDPDPTPPGTDTTANLKSEIEKLSKEVSRLSEENKTLDDKYKRAL

		Function GO:0006457; protein folding; Biological Process GO:0042803; protein homodimerization activity; Molecular Function GO:0051087; chaperone binding; Molecular Function IPR000740; GrpE nucleotide exchange factor Roe1; GrpE protein homolog, mitochondrial; K03687 molecular chaperone GrpE GRPE_DROME GrpE protein homolog, mitochondrial OS=Drosophila melanogaster GN=Roe1 PE=2 SV=2 Q17CT4_AEDAE GrpE protein homolog OS=Aedes aegypti GN=AAEL004438 PE=3 SV=1	ADGENLRRRMMKQIEDAKLFGIQSFCKDLLEVADVLGKATESVPKEEIKD SNPHLKSLYEGLKMTEVQLHKVFKRHGLEPISPLNEKFDPNLHEALFEQGD TRETDGFEQRSTEGTVVGGGHGGDGPDPH
LMI_GLEAN_10 145912	COG5078	acid-amino acid ligase activity; Molecular Function GO:0043687; post-translational protein modification; Biological Process IPR000608;	MSTPARRRLMRDFKRLQEDPPTGISGAPTENNIMLWHVVIFGPHDTPFEDG TFKLTLEFTEEYPNKPPTVRFLSKLFHPNVYADGGICLDILQNKWSPTYDV SAILTSIQSLLSDPNPNSPANSMASQLFKENRREYEKRVKSCVEQSFLDT

		Ubiquitin-conjugating enzyme, E2 IPR016135; Ubiquitin-conjugating enzyme/RWD-like ubiquitin-conjugating enzyme rad6 ; K10573 ubiquitin-conjugating enzyme E2 A [EC:6.3.2.19] UBCD6_DROME Ubiquitin-conjugating enzyme E2-17 kDa OS=Drosophila melanogaster GN=UbcD6 PE=2 SV=2 Q17J88_AEDAE Ubiquitin carrier protein OS=Aedes aegypti GN=AAEL002118 PE=3 SV=1	
LMI_GLEAN_10110788	COG5078	acid-amino acid ligase activity; Molecular Function GO:0043687; post-translational protein modification; Biological Process IPR000608; Ubiquitin-conjugating enzyme, E2 IPR016135; Ubiquitin-conjugating	MFQELQDLGRDPPAQCSAGPVGDDLWSGTACDFILACGRNLFGEGAELLPRKIKNTKQKGVTKYMKFGVKERPDSPYQGGVFFLTIHFPTDYPFKPPKVAFTTRIYHPNINSNGSICLDILRSQWSPALTISKVLLSICSLLCDPNPDDPLVPEIARIYKTDREKYNELAREWTRKYAM

		enzyme/RWD-like Eff; effete; K06689 ubiquitin-conjugating enzyme E2 D/E [EC:6.3.2.19] UBCD1_DROME Ubiquitin-conjugating enzyme E2-17 kDa OS=Drosophila melanogaster GN=eff PE=1 SV=1 Q7PRY1_ANOGA Ubiquitin carrier protein OS=Anopheles gambiae GN=AGAP000145 PE=3 SV=2	
LMI_GLEAN_10135161	COG0760	isomerase activity; Molecular Function IPR000297; Peptidyl-prolyl cis-trans isomerase, PpiC-type similar to peptidyl-prolyl cis-trans isomerase; K09579 peptidyl-prolyl cis-trans isomerase NIMA-interacting 4 [EC:5.2.1.8] PIN4_DANRE Peptidyl-prolyl cis-trans isomerase NIMA-interacting 4 OS=Danio rerio GN=pin4 PE=2 SV=1	MPPKKGAGGAKGGSKSTEDTKNAAGKEKKGGSAVKLAWPVIGTGVGCAGWVNWTGLAQGYDPCGKRMGFAMVRHILCEKQSKALEALEKLKGGMKFPEVAAQYSEDKARQGGDLGWMTRGSMVGPFQDAAFALPVSNVTNPVYTDPPVKTKFGYHIIMVEGKK

		Q2F670_BOMMO<br>Peptidyl-prolyl cis-trans<br>isomerase OS=Bombyx mori<br>PE=2 SV=1	
LMI_GLEAN_10<br>136306	COG5078	intracellular; Cellular<br>Component IPR001370;<br>Baculoviral inhibition of<br>apoptosis protein repeat<br>similar to Bruce CG6303-PA;<br>K10586 baculoviral IAP<br>repeat-containing protein 6<br>(apollon) [EC:6.3.2.19]<br>BIRC6_HUMAN Baculoviral<br>IAP repeat-containing protein 6<br>OS=Homo sapiens GN=BIRC6<br>PE=1 SV=1<br>B0W6D8_CULQU Survivin<br>OS=Culex quinquefasciatus<br>GN=CpipJ_CPIJ002689 PE=3<br>SV=1	MADEDPWKLKEDGYLNVETDSKSIIYHPHLNIIFVLTRDSEVKVLDVNSGV<br>ILQRSSLSAKPGGNLCGTYLHGHDKILFTDGRGLGVRRDYNGVMFLDSILQ<br>TPLMKPDDVVKIELLLSEAILLQQSLRCIELPGVDQTTEWNSVCLELPHCAL<br>KLVCAGMVQELKRQNKHIPALSIASAVSERLNCLLVPPALAAESPPVTDRA<br>LMFSEAARRDTFVKWPHMNYKWALPDQMAQAGFYHQPNSTGDDRAMC<br>FTCNVCLVCWEPTDEPWSEHERHSPSCPFVKGEYTQNVPLSVTYATAPAV<br>PVADSRDEEVVCVSTSSVPDLVATATCHGLVTIWNITRQLKVKPEYKLTIE<br>ENISHRSKELFNFHNNIFSLPMVCEDLDLEITALSIVGGPQHQQSCNHAGPK<br>QYSKATNSSSSRLRPSFIAGISVGSRSPPNTSDVWGSGGCCSSLVRAMNDV<br>NEASNLASASLDIMKVRDSTESTAGHTVSSCTHGRCPFLVVYNFHHYGGS<br>PGCEVDSTADDVSGSSGGGGQNQGIAGGGSSKKAVSGTGTSTSSGPRQDG<br>VYQEIFLSKFMFDVPGVDDLVADVVMDTPPDCLESVEFLSGSAPPGAALY<br>GPPVIPSGSSQGASGSGGGTACDSSMLNVTFSNVNGGLDMSKYPSTSEETH<br>REPIAIQCLPVPQRLRNAGNLAITSIHPTLDGGHIFVVVSTTAAVDVRVPVA<br>DETDSKMDVDVEVCYFKNDPSYIARGGTTRSSSRGKDSASIAGALLVYSL<br>DLSGSAGEAIRLDEEPVAVRLLSDIGECPTEVTLLPLAERDESTRPEVPSTPV<br>KGVPQGLAALICRDGSVRIVSLVNLRTVAVAVPEKSGTRYISAAYCNSLER<br>LCVCTEKGSIHFFVMSDEGFTEECEDDQSESYMIPSSSTSQLSSGNSHLLAN<br>KTELSMSDLRQLYELTLFENLTPCYAAAVPPCWSEIMQAQKQRRHPQHLQ<br>QGDDLQLTRSWRLQNDATTWDEHVFEITLPRSCCIGHVDLKFSLHSPCSNP<br>PNIQVTLLKQNATGIGRKEKTMMPQVDEGIDFNIGMQCDGVKGKTENPVT<br>SEEYMRDHNCEVLCGPVILSQCLDLSEQGGTITLTSPKLFRVRGRTFLVHIK

			AVATEKELVKDTVISITIRKTKQTNIPNERSQRCAMLESIQFVERLLHVVFL<br>EGPDRTTTAQGGMKNASDGGNARFDASVLQALIDWLPIISCCQTAGALRW<br>FLLLLTRVLPFDSTGAAAQQCVTLLQQVAQEMNTRTNPFHLLLRTRFGLY<br>STPFEPELFDTEPPPPAKFSSVPITYATVVSGEMGGATAASAAAFSADVLDL<br>RELLTTSDSKFTQLRLKGLSSNHYLKGLLEVEPLHFTCHAASDGTKMEKID<br>QGKYCLRVUGSGRHIYDNISLLEEVEMKINEVPGEVGEKVWAKMQTVLQ<br>NNTGLKELCAAADILSGKSSTPDCFIPVQPVLKMKYDUYSRKVWIAFIITCT<br>ICKIYPVENTGSHLILWVMDSRLKLKKRQKASLPWQQLLTAPPQQTLLIER<br>MHSGARRFVVLDFGCPVLLTDLMVPACSDLVSLSVDIWCRGEEFDGQRLV<br>VASDIGSKALVMSDLQPPPICRFLKITTIGRYGMSTTRCKIPIGSFYGHIIILP<br>GENYANNNDVREVSPDSIQAHLRMLSALFEDIHCRYSLACAKLQEFLTPLL<br>TSEIPNVVHMYHYLQRRREFEKLFPSQEGQKIMSSFHECVTFQHQLNLVCS<br>VIRRLETAQGKLAVLPQVRDTQKLLSTCCTDKLRVLGEGLLDVLLYLVYEI<br>GPVPRVPVSLYNIFDQSVCERLFHWLCVREDTHIQLSACTLLARMCGMQP<br>WWGDFLASTLSRLYSSQNTAVFPQDRVFILLTYLGRKSLAGGASRSSVLDS<br>VLSMLTRLLSPLATPQHSAPGFLRAEMDLSQIGWVLLFLSVCLDASCTPTP<br>SRWDFIQGEMAMQRKMASSARSNASRSYRRKLQKRLMHHKQHLEDIELS<br>NKTYHASKQALSALSSQAATLSSKLEAALKQQEQFFKKTLKQHSAKHIKD<br>ILQIRRTDGSLLSKQFNSRGSQTRSGGSGGGSSRLGSEREDSETTTLTSDPR<br>DPVLLLPQERCLPVARGLVTLVLSMDFTCNIDMFLLACKVIARIVAVARPA<br>ISLSELVTQEQLLHLIHLAVWNDQHKASWGGPWASHAISCLLQDILEGTRL<br>FRETDPELIISDHEATPPPSQPEAASSSSTSQQEDAFVADDSSESGNAGPSTS<br>KGTGNCFQLPSLLESDDSELDEFLDDILERGRSLAKRTSAAVANRVTSATG<br>ITGTVATSSLSVAMDARLELGVGTSAEVALRRLAAQGAHNLALSVTAPVI<br>APPESARQPYSSSTAGNLFEHQVLCWQKSWMNIELVLQLWLMLNSDSAFE<br>PVGGAPGLFDPGAVPAVRVTTQAVSGLISALAQQPGVSLRVWCLAFQCLI

			LMANQPQIQTRCGEAQSMGLDDVEVSLHATSGVIIEDPQFLPMLLRFLSGS<br>SLSYVSSDKGTQAGPSVCGAMRELLVRLQSRCDVISPGSRLGNSLKELLLQ<br>LVYKLVQTTGALTNRQGPLDAQCELVDLVLNLNFTAVNLSTAISIVECVA<br>NLAYNYILSSEKVRCRSISEHSTGASDASSCFGGLFASVLGGEVKQSRPAT<br>WDALLCNLLKLCSHIVQTPLSNEVSGKKSVNLIIFYQVPRHSEESMDTGES<br>QLPESETEPSGSEKQSPFGQHLSWQLEQELKVPCAADTVLEHQTTVLRLLS<br>SLAGCSGSTLAMLLGSAAASPSLNTSSGDRLSASAFGDLGEPLSVGDAVFQ<br>LLLTLARKATNVGTIGVMQLSEPLLWFILHVLDSAKALNEFNAMGGVKVI<br>CENLVKSNQALINSHPSMVSVVMQHFSHIPSASTSTSKKLSNPIETYEGLLN<br>FAPLGNISASNLTAQPADVLIQSAPPHRRARTPAWSYHFYPEESWVELTLT<br>LPCAVLLKEVQLQPHLTSLATCPSAVAVEVCRDCSVGLVPVCPPVRTSGLT<br>FIRLHLPQPEIVTSVLLRLYKPHDSSNIGLSQIRLLGSTTFGETAFRTVNMDV<br>PDEEQLTKSSLGWLRLLHHCLCVPSIGSALSHDVAASAAEVEGLPEACCGL<br>LLVPAPAPSLFTPSLERVLLRLGLHSRDLGLKQINTLLRNGAAAFLQGSLPT<br>SHSSTSNSTVVDSVVELLYQLCTIQDSATKDRVKALLLWLQETASAAIQAQ<br>EQSFSSTEIWSPSSAYVHSAAAILWAGHERKVKYDLYSMVTPELFQIVYK<br>WSLSLPSHSALKKAIDTLLCAMCYIRHSLFPTLLQRTGVLLSNLGISQLATE<br>LNNSASDSNLQSQDEKPNGEWYSHVVAHEMDKMQRSESQLMTLAMACQ<br>SPPSIQQLLDSGLPAVLTRIILDFCHQEEQQLSDNSDKEAQSSSKKSAYQDS<br>SSLASDMVAGTSNHNSATNFSQDGLHMLQVDTITMILKFFAEVCSEGKMR<br>DWLGSSEGSVFWLPLLHLLCTKSCSVDSASSRIPKRCELRSESYAALESATI<br>KFLSQCCWCHPSNQRLLSKVLCEVINEQKTTQDLSFLHGISGFTRRLILQLL<br>LENEKIFVFIKSDYPLQRVSMATSAPYHPRYGVGHQKQLFYFSTQTTCGDI<br>LKSISDYSKFIVIDTFMSIFSGGLVGSKKELWELGIYMVDQLSVQAGVSAK<br>DKRTKDARNTVAAATRSISGKKSRSSSLADSSQGPSQVPSQTASMSSVQPH<br>QFLEHDTCPGIPLPPQLQVSQLLTLVLENGDSLSTPCLTLNLHNLPRTVEKN

			SKDNTLEAETERLLQLPGLATPLQVFTEMGGLALLAQHLPLVYPDTLRYAAPDKVPASTPDQIDAEWVKVDDIYEDLDESMHGGSSSPGGAGGRTTALPPPSVPPHSLAAFGLFLRLPGYAEVLLQDKKKAQCLLRLALGVTDDGEGGDIFSSPVANHLHTLPFQVLRQLFDSTPLTTTSGALLRRTTIEIGAIHLLLGCLAIFTHQSQDITLPGVQHELVLAATKASATGGETNVRGGKSDDKSHLYWAKGTGFGTGSTAQSWNVEQALLRQRSEEEHVTVLLQVLDETVTDCGKEQPQLPRVFYELLKQSCLLPAVSAYLRNDSVLDMARHIPLYRAVLQLLRAMALSSQLVTLLLPHDGGQHDDLSVVCLLTKMRTCVDTYASRLKLNKSKGNGKSRTNAKLQEETEQDEGLALLIPDIQETANIVQAATDRLTMEDGQENSVVREGPHLSSIDLPLRSHEERYLEVMRRLQFATGPNEMITESSEGGYQFVVSYHFESNVRSAGERSHPARMKRLAQETVTLSTSLPLSYSSSVFVRCDTDRLDIMKVLITGPAETPYANGCFEFDVYFPPDYPNSPMLINLETTGHHTIRFNPNLYNDGKVCLSVLNTWHGRPEEKWNAQTSSFLQVLVSIQSLILVPEPYFNEPGYERSRGTPSGNHSSREYNSNICQATVKWAMLEQIRNPCPCFKEVIHTHFWMKRHEIVKQIENWIADMDSQCGDRRTGRAISLNSMALKRHFRQLKEELNKLKTPEGLEDLADSCLELSPPSTSSAQSVSESKDASPVVSTPSQGFLPFGATSSTNDTNKKPAEENVPTEVYKDMEMEKMVSQ
LMI_GLEAN_10032509	COG0278	electron carrier activity; Molecular Function GO:0015035; protein disulfide oxidoreductase activity; Molecular Function GO:0045454; cell redox homeostasis; Biological Process IPR002109; Glutaredoxin IPR012336;	MGVINIQTKTEFEDVAKSNNLVVVHFYADWASQCETMNEVLEELAKQPELNGVKFAKCPAEDVPEVSMKYDVSSVPTCLLLNGDILLDRVDGAKAAELTKKVNQLVSSPPVLPSRVEETAKEDLNTRLRKLITAAPVMLFMKGSPQEPRCGFSKTIVGILDKHSAKYKSFNILADEEVRQGLKTFSNWPTYPQVK

		Thioredoxin-like fold IPR013766; Thioredoxin domain IPR017936; Thioredoxin-like Hypothetical UPF0055 protein YPL059w; K07390 monothiol glutaredoxin GLRX3_HUMAN Glutaredoxin-3 OS=Homo sapiens GN=GLRX3 PE=1 SV=2 Q5XGR5_XENLA LOC495269 protein (Fragment) OS=Xenopus laevis GN=LOC495269 PE=2 SV=1	

Supplementary Table S2 The top 70 most abundant proteins in the locust head

No.	Accession	Blast P	Seq	Peptide number
1	LMI_GLEAN_10128031	Twitchin [Cerapachys biroi].EZA52953	PVDKSKPGMKVPEIKAPEGPKIEVIREKTPTRTDSRKGSLAPGSGPPSRRGSLIPPEDQQRRPSLIISDEVGKLRPGEVLDSKRRRPSGDMRRPSVAELGEMIDKPSTPLRAVGNPGPPVIVDVQESYSAVEDQTGYITIGVEGNPAPTFKFYKGMTELLYIEGGRFKFVTDGETNSITLCIRKVKPNDEGKYKVVVSNEHGEDSAEMQLYVSDASGMDFRAMLKKRKYAKWGKDKEEPDWGDLKEVEKEVPKLKKVEKKQESFLKPLVDQVAKEGKDKKVVFEAVFSKPNAKPKWMFRKDELFPGSKYKFVNENDVYKLIINTPKVEDTGKYTIEIGGISCTAFLNVEEQEFTVFPMWRIVMTLMAFFCSIKISWRQLELPHKIVPYDVILLKNEKYVICTYFSDTLSSSGKYPSDIHLFIQKVKGYTMQSITLYSILQECYIYLHCLMISTALLFLVLTRGDFGESKHIYICHISCCPTVDRIIHIVKDGRKRKLIIKDSKVTDCGMYSCISNADKTEAELVIQYQNRFNKKLKDTTVVEREKLVLDIELQDQTAPAEWFFNGEPIKESDRIQIKNLGGGKHQLIFNEVQLDDSGEITCESGKLSSSAKLTVKKGESKPLIDFPDTVDGPCNKPIIFEVPYKVEGTRQSQIEAKLLKDGKPLPLKDVDVVVAEDKITYKIKKPARNLSGPYQIKISNSQGEAVQNVKINMQDVPTPPQDVTVTEIFQNSCVVKWKPSVDDGGSPLVHYVVERLDMSGWDSVAEITPDKPTVFKCEDLVHKREYKFRIRAVNKFGSSEPALFAKPVLAKDPWDEPSKPTNVEVLDWDKDHADLKWTKPENDGGAPITGYVIEFKEKFGKDWQTGKVLEGDITAATIDGLKEGTQYEFRIRAVNKAGPGEPSDATKPIIAKARFVKPFIIGDGLTNIVVRKGQVIKYDIKYGGEPEPVARWEVDAKEIIEDKEERITIDKYERNTVLTVRRTTRVDSGKYKLILKNDSGICESVADVVVLDKPSMPMGPMKVEEVRANHVKVKWDKPEDNGGTEVTGYVLEKMDLDTGRWIPAGEVGPDKNTFTFDGLTPKKKYKFRVKAVNKEGESEPLETEGAILAKNPYDEPGRPGKPEIFD	166

			YDNKSVSLRWAKPENDGGRPITHYTIEMKDKFSVDWVEVCKTQDSTCEAKVE<br>DLKERMVYQFRVRAHNKAGASEPSEPTDTHVCKHKHLKPRIDRATFKSVTIKA<br>GRTHKWSVDISGEPPPAVTWLWKDSVKLVPTEKIKIDNVDYHTDFTIINAVRR<br>DTGKYTLIAENASGKDQETVELTVLGKPSAPQGPLEVKDVTKTSAKVKWQKP<br>EDDGGCPIKEYEVEKMDLATGKWVRVGRVPGDRENPEMEITGLEPGAEYKFR<br>VTAVNDEGDSEPLVTEKGTIAKNPFDEPTAPGTPEITDYDNESVDLKWTKPESD<br>GGAPIEKYIIEKKDRFKPDWEKAIEVPGDQLAAKVPDLKERAEYQFRVVAVNK<br>AGPSPASEPTKTHLVKHRALKPRIDRTNLKPIVIRAGKGVKYDVNIRGEPPPDV<br>KWFLQDTEVKSEGNYEIINVDYNTKFSLNDAVRKNTGVYKIVAENQHGKDEA<br>EVEITVLSAPSKPKGPLKVSDVTKNSCKLKWEKPEDDGGKPITGYVVEKLDKE<br>TGRWVPVGRTLEPEMDVKGLQEGHEYQFRARAVNEEGESEPLETERATLAKN<br>PFDIPSKPGTPEIVDWDVDRVDLKWKAPKKDGGAPITGYVIEKKEKFSSSWDEI<br>LTTTSPATEARVPGLKEGNQYQFRIRAVNKAGPSEPSDSTKPHIAKARFLKPLIN<br>REKLQKVTVRAGQFVKFDVDVKGEPPPVITWSFANKVLENGPTVKIENEDYNT<br>KLTLSETTRKNTGTYTIKAENDSGKDEATVDVIVLDKPGKPEGPLEVSDVHKE<br>GCKLKWSKPKDDGGLPLTGYVLEKMDTTTGRWVPAGFVDPEKTEHEVTGLE<br>PNKKYQFRVKAVNEEGESEPLETDTAILAKNPYDPPSAPGLPEIVDWNENMVK<br>LKWERPIRDGGAPITGYVIEMMDKYGGAFVKCAEIPGNVCEGTVPKLEEGNQ<br>YQFRVRAVNKAGPGDPSEETNPHTAKARFLKPRIDRTNLQNITLKVGLTVSLDI<br>NIIGEPPPEVTWYFKDKELKTEDQIRIDNIDYNTKFFIMKAMRAHTGKYKIVAK<br>NSVGEDSAEIDITVLGKPTKPKGPLDVSDVTKNGCKLKWKKPEDDGGSPIEYY<br>EIEKLDPLTGQWIPCARSTEPEANITGLQEGKPYKFRVKAVNKEGESEELETDK<br>PIIAKNPFDEPSKPGRPEPRNWDKDFVDLEWTKPKSDGGAPIEKYIVQMRDKE<br>GRNWVDALTVPGDRTAGRVTDVVEGHEYEFRVVAVNKAGPSEPSDVSKSVV<br>AKPRFLAPYIDRKNLQKKVLRSGQLLRIEADVKGEPPPKITWTLKETVLKSHDR<br>LKIENEDYKTTFILQKVKRADTGVYTVTAKNDSGVDSVDVEISVLSKPSKPKGP	

			LKVSDVTAEGCKLKWDKPEDDGGEPVDHYVIERMDTETGRWVPVGTSKTPE<br>AEVAGLNEGKEYQFRVKAVNAEGESEPLETDVPTLAKNPYSEPDKPGKPEVR<br>DWNKHQADLKWAPPKSDGGAPITSYIIEKKDQYSSKWQKAVEVIGNKCEAKV<br>PDLVEGMKYVFRVRAVNKGGQSKPSEPSDTVLAKDRFAPPRIDRSTLKDTTIK<br>AGQMIRFDVKISGEPPPSKSWFLNKARLESNDEISIESEEYRTKLLIQTATRKHS<br>GTYLIKAENNSGKDEASVEITVLDKPSKPVGPLKISDVHKEGCNLKWNAPEDD<br>GGSPIEHYVVEKMDTETGRWVPVGRSKEPNMEVANLVPGQEYKFRVMAVNA<br>EGESEPLEADQAIVAKNPYDEPDAPGTPEPTDWDKDHVDLRWTPPTSDGGSPI<br>TGYIIEKREKGSPRWTKAGEVKGPECKGTADNLEEGVEYEFRVKAVNAAGPS<br>APSQASKPVVAKPRKLAPKIDRRNLRNITVRQGEPILFDVKIIGEPPPDVTWTLN<br>DKSIQQTSTRRIENVPYNSKFFNDEPDRKDSGIYKITAVNKYGSDTAEVEVNVV<br>SKPGKPEGPLEVSDVHKDGCTLKWKKPKDDGGEPIETYVVEKFDTDTGVWLP<br>VGRSKEPQMEVTGLIPGHDYHFRVKAVNKEGESEPLETLTPITAKDPFTVPGTP<br>GAPEPVDWSQNHVDLVWKEPAHDGGSPITGYIIEKKDKYSMMWEKALETETP<br>TPQAVIHGLIEGNEYQFRVIAVNKAGPGEPGEASKNFVAKPRFLAPRIDRRNLR<br>DVTLSAGSTLKFDVNIIGEPPPNVEWRYGSIPLKSSKTVQVDNVDYNSKLVIRP<br>VHRGDSGEYTITASNSSGKDSVTVNVTVTDKPTAPEGPLQVSDVHKEGCKLK<br>WKRPKDDGGTPIEYYQVEKMDPETGCWVPCGRSNEPAMEVTGLTPGKEYKF<br>RVAAVNSEGESEPLVATETIVAKNPFDEPGKPGDLKATDWDKDHVDLKWTPP<br>KDDGGSPITGYIVEKKDKYGQWEKAVEVPAGQTTATVPDLIEGQPYEFRVRA<br>VNAAGPGEPSDATPTIIAKPRNLAPKIDRTNLIEVRIKAGQNFGFDVKVSGEPPP<br>TTKWMLHGKEVRPSDRIKVQHAEYNTKINVRMATRAESGKYKIVAENINGRD<br>EAEVNVIVLDKPSPPGGPLKVSDIHAEGCKLSWNPPADDGGQPVEKYVVEKM<br>DEASGRWVPAGETDGPQTSLEVDGLTPGHKYKFRVRAVNKQGKSEPLTTAQA<br>IEAKNPFDEPSQPGTPEITDYDKDFVQLKWAKPEKDGGSPITGYIIEKKDKYSPN<br>WEKCAEVEGDVTTGKVPDLIEGNQYEFRIRAVNKAGPSEPSAPTKPHTARPKN	

			LAPHIDRNALLDIKVRAGQNFEFDVPVIGEPPPNKQWSLKGDAVINTDRIKVIN<br>EDYNTKLRVTDAKRADSGTYTITAKNINGVDTATVNVTVLDVPLPPEGPLKPD<br>NITKSSCSLFWRPPKDDGGSEITHYVVEKLDTENMRWVPVGECNGTSMRVDH<br>LIEGHDYNFRVRAVNKQGESQPLTGTQTITAKDPFSKPDKPGTPELVDWDKDH<br>VDLEWTAPKKDGGSPITSYIIEKRTRFGPWEKAAEVPGNQTKGTAPNLTEGEE<br>YEFRIIAVNKGGPGEPSEPSASVVAKPRFQSPVFDKTLLQDLVVRAGQRINYTIP<br>IEASPKPTATWTVNGKKVEPGVRADIHTTTKSTVFEILFSVRADTGRYTLTLEN<br>KYGQASASANVTVLDRPSPPEGPLIVSDVTKESARLSWKVPLDDGGSPILHYVI<br>EKMDVSRGTWSDAGMSTSLTHEVTRLVHRKEYLFRVKAVNSIGESDALETEK<br>GVIAKNQFDEPDAPGKPLIMDWDKDHVDLEWTAPKSDGGAPITGYIIQKKEKG<br>SPYWVNAVHVPPNKTSATVPDLTEGQEYEFRVVATNSAGQSEPSEPSDTVTAK<br>ARYLAPKIKTPLNDIRIKAGQILHVDIDFIGEPQPEVTWTVDGKPLKTDERTTITS<br>IGYHTIVHTVSTKRSDSGLYHLQLRNNSGLDEGSFQVIVLDRPSPPEAPLEYEEI<br>TSSSVTLSWKPPKDNGGSEITYVFLFPRLLEGTKYEFRVSAENLQGRSEPLNTT<br>KPVVAKNQFDVPGRPGKPEGVDSDKDHIKIKWTPPISNGGSPIIGYDVERRDRK<br>TGRWIKVNREPVRQTEYYDDTVQEGHQYEYRVSAVNAAGPGKPSDTSQVLN<br>AKSMREKPKLYLDGLIGRKLKVRAGEPINIQIPLSGAPLPTVEWTKNSIKLPESN<br>RVTAETASDNTHLRIDSSTRNDSGKYTVTAKNDFGKDSADIEVIVVDKPGCPK<br>GPLQYTSVSQDSVSLQWNPPADDGGGDITGYVVEVAEFGTDSWRQVPGFCPK<br>PAFTVKSLTEGKRYVFRVRAENIYGLSEPLEGKPVVAKSPFDPPDAPGQPEITSY<br>SPNSCNLSWTPPQNTGGKPVTGYYVEKRERGGEWIKVNNYPTPNTSYTVQDL<br>REGNRYEFRVVALNEAGPGKPSKPTEPLTAQAQRLKPDAPEPPKPDRVTKDSV<br>TLSWRPPRSDGGAKIKGYIVQKKAKGDKDWSDASGAPIPVTVFTVPNLKEGDE<br>YQFRVIAVNDVGPSDPSRPSQNVLIEEQPNKPCMDLGALRDITVRAGEDFSIHV<br>PYVAFPKPTASWFANDNLLDEADTRVFQQLADDFASIVVKNSKRSDTGQYRL<br>QLRNPSGYDTATVNVRVLDRPGPPENLRADEFAGDALTLYWNPPKDNGGDI	

			TNYIVEKKEPRSPTWSKVSSYVTTPFCRLRNLVIGREYEFRVMAENQYGVSEP<br>AVTKDPVRARHPFDPPGPPGAPRGVETSEDSITIAWTKPRHDGGSPITGYVVEK<br>KLINEDKWTKANHVLIQDLTYKVCSLIENHEYEFRVAAVNAAGQGPWSSASD<br>AICCRPPPSSPKITSDLSIRDMTVIAGEEFTITVPFTGNPVPKPTWTINGEEVIPGD<br>RIKFETTSNQTVFHNKCAKRSDMGNYTIQLVNIEGSDTASCKVLVVDKPLPPQ<br>GPLDVSDITPETCSLSWKPPLDDGGSPITNYVVEKLDPTSGLWTKVSSFVRNCH<br>YDVIGLEPNRKYSFRVRAENQYGVSEPLETDQPITAKFAFTVPDPPGQPRVTD<br>WESGNITLIWDRPLSDGGSRIQGYKIEYRDVAEDHQWRVANDYLVKDTTYTV<br>HSLLQGREYEFRVRAKNAAGFSKPSPPSSRFQLKGKFNVPSPPGTPKVVKVGK<br>SYVDLTWEPPISDGGSRITGYIIERREVGGPPWARCNEYNVTDCSYTVLNLVER<br>ADYEFRIFAVNAAGRSEPSSCTTPVKICEVEGGEKPEFVRHLLPTTVPLNSSVVL<br>ECEATGKPPPSARWLRNGREIMPGGRFRMESKGGVFRLIISEVWEVDDGDYTC<br>EANNTIGFVTTTARLKIGAPPRIDRMPGDLYLPEGDNTKIKIYYSGDQPMEVTL<br>TKDGRKIDETTHIKYTVFDEYLIIFIKEIVKSDAGTYTLNVKNDSGSVSASFTVYI<br>TGLPGPPIGPLDVSDITKHTCTLNWKPPTYDGGLRITHYVVERRDISLSHWIIVS<br>SFCKDTVFTVQGLTEGQEYLFRVMAANDNGMGPPLEGTNPVKAKAPFDPPSA<br>PGVPVVKEVGGDFVNLSWDKPESDGGSRIQGYWIDKREVGSEAWQRVSATLC<br>VATTINISNLIEGRQYEFRVFAQNVAGISPPSTASTSVKIKDPHAAQPPEIVKPLR<br>NVSAIQNRNAEFKCTITGSPRPVITWYKGARELSQSMKYHMLTEGDSYSLIIND<br>IFGEDADEYVCRAVNKGGVKSTRAELFIMTPPKLNVPPRFRDSAFFDKGENVVI<br>KIPFTGHPKPRITWTKDGETIESGGHFALEVKERHAVLTIRDGSKLDSGPYRIVA<br>ENDLGMDSAIIKIQISDRPDPPRFVTAENIGHDSLALSWQPPTWDGGSGITNYLV<br>EKREHPMSSWIRVGNTRFTTMAVTGLSPGHEYEFRVYAENIYGRSDPSSTSTLI<br>KMKDTGKKIVRKKKYEVDATGKKIRGQADGTVSDYDQYVFDIYSKYVPQPVE<br>IKTSSVYDYYDILEEIGTGAFGVVHRCRERKTGNIFAAKFIPVSHAMEKELIKKE<br>IDIMNQLHHPKLINLHDAFEDDDEMVLIFEFLSGGELFERITAEGYSMSEAEVIN	

			YMRQICEGVKHMHEKNIIHLDIKPENIMCQTRKSTNVKLIDFGLATKLDPNEVVKISTGTAEFAAPEIVEREPVGFYTDMWAVGVLAYVLLSGLSPFAGDNDIETLKNVKACDWDFDEEAFANVSDEGKDFIRRLLVKNKEKRMTAHECLLHPWLTGDHSNRTSVISSSRYTNIRDKIRAKYTDWDAFILPIGRLAEYSSLRKLLVDKYRIHDTSFDRRQAAPRFVIKPQSAFAYEGQSAKFYCRVIAIATPTLTWYHNNAELKQSVKFMKRYGGDDYTFIINRVKLEDRGEYIIRAENHYGYREEVVFLNVQPIPKEIPQYRPEVQPVRRREPLSYALWQDERESAPSFTFHLRPRVMQIRQTCKLLCCLSGKPVPTVKWYKDKRELSKYDYTMTHADGVVTMEIVDCKPEDSGKYRCVATNIHGTDETSCVVIVEGYGSTAEQTELANKLMYSGDRRYIEQPSRPTSSPSVSVRKSSYKASDTYSSSDYRSSASSSAKQSSTSHSNYKVUSKTTQSSDKRVMKKYGSKLDATGSPSRSRSATKELILPPDDSLMCAPEFTKKLDDLTINDGDHLQLSCTVTGDPDPQITWSKNGETLTSSDIVDLKYKGGVASLTINEVFPEDEGEYCCEATNSIGTAKTKCKLTVKPMESASGAKSKGGDSAPKIVSHLESAYVKDGEPVTLSCRIIGAKKFDVIWLHNNKEIKPSKDFEYTNEANIYKLNIAEIFPEDSGTYTCEAFNDAGESFSSCTLVVLVPNEEPKSPVFKTFPQSATVQEGESVRFECETEKVPVK	
2	LMI_GLEAN_10163232	Apolipophorin II,Locusta migratoria, Q9U943	MSNVEVNIIGVVQQLKSLNKNKSSGPHCILIRFLSDYAEAITPYLTAMYNRSLKEGSVPKDLSIPEFPSTTAAQSSVFQYQKGQTYTYSFEGTTLTSLPGTQGEPVRLKLKATADLSVADDCNKVLRLRGVTVSGPDSKNYANLKDLEAHPVLANFKGSSINKQLCSEDGDNQSSLNIKRAILSLLQTPNTKSSTASEVDVFGICPTNVRHSQRGDVTVISKTRNLNRCASRENLIQETLSTRFTGQSDLHATPFLDADLHVEQQIKGGLIVSATSRESYLFRPFSNQGNGAKTIVETKLTLTSQNAQPAPPLASFTVPKSIVFEAPHALASVPGGSSAITAALHAAESSTKDGVTVDAAEKFRTLVSVLRQSSTTDILKVYNDVKAGAGFSNKHSARNLLLDALFRTSTGDAVEVIARLLKTKEITANHWYLSLAFIQHASLKSVVSISSLLDQKNLPTEAFLGIGSFIGRYCREHNCENVAEFDEVLNKFSKHLSGSTTSKAGENRAIAALKALGNIRHLNNALGEKVKQLALDKSLPPRVRVAALEVIQSDPCRKNIKQAALQILRDQVEDSELRIKAYLAVVECPCDNV	103

			VKTISNLLENEPIIQVGSFVVSHLKNLQASTDPSKAEAKEKLGQLKPKKIFSSDI<br>RKYSQNYELSYAIDAINAGASVESNVIFSQSSYLPRSVSLNLTADVFGHSYNVF<br>EIAARTENLDHIIESFLGPKGYIETEDDDKFADEVQEKTKSLYNRITERFEKTFR<br>QKRSVSKDAVDNIRQQAYKSLLPSQRDRSLDVDLSLKTFGSELAWFNYDGKH<br>EQKSSERVVDEIFDAIDEGLKKSKKFNYDFEPHFTFLDSELSYPTNLGFPLKLAI<br>DGSIAARLKLNGEVDVRSILRQPENAAFRLEFVPSAAVELTGKLLVDAYVVEG<br>GLKLDYNVHSSTGINVAVHNLNDLGIDIKVGLPVKKQDIIDVKTDVLTTVKER<br>GHPETSTPLHFNLKGNDYKQYRGCFDQLSPVSGLTFCGNVSVPWVSPTQAAAF<br>YPLNGPSHLSVSIEADDVSEYHFRAEIKKDESAFKSAAVLFDTPGSSADRKVLL<br>LVEKKEKPHQGITAHLKSGWKEIVAEGLLIDDNNEKSVSAKLVIESDEYSIKGG<br>VKISGNPSRQVYKPILEYKAPAKDSGAKVKKSHKTSEGITVDGAVVVERTSDK<br>GKYTFQDLSLKTPKGTFVINGQLDIVPRNYAFDLKLSVDKNELLLNGHLNYAE<br>PKSIDVALEVTSPQFPDYGSGFQLINKRGDDYSDTKIILACGRDLKSDGSRLILE<br>HFIKGKYETPDTFNLETKGEVLGTGHKIFGKFDIDSKPKHLEYDLKLGFDENEV<br>TSDLVAKRDIKSPDDYELKFSAKILDNSIRIESSREVKPKDDSAFLNTLVVLSGK<br>KYEFQVDVKLAAEDEYHTSLKAESNLKIEGKTSVRLITDFTTDAQTVNGHVKV<br>SNEGEDFFELIYKLNRGSGNPSGNAKLFVKNYLDGAATFKYNNGVGSGTLQID<br>VLKLHRKIKATGDLTLSGSQRSAAIDLYWDADRDQSKQLLFKTENDVKEKSID<br>SKNTLKILDKLTTLNFKGSLSGAIDDGEVEGQGELILPAGTYLGVKFGRALHLT<br>QADTKVGLHLQAEGRESASSTQPVWKSDFLLESAVTRDSFVGEAKLLFETKGK<br>DDLKLFLSGKSLPQGEKKLISGQFSGQGSLIGGRTSVIKLNSEIDETFIAYNLNSE<br>CNQGYRANIVGKINRGYSPVAVKQIENTLELLLPFDKLKQLKHTIIGTFSSQPES<br>TPEFTVSNVIIWNNENTLKLTGEAAGDEKEGRTKWDLILPKEEPRTLETTWSNA<br>GDNKKAGSLSFKWGGNKEAKVSTDIEFTSDNQPQILHLKATSPTEKFGIFDLAL<br>SLKKNADPADKIDFELTVTADQKKTDVKGSLGLAPGVPIIDVVAVQPSGTSKV<br>FVDFLRKSDSELHGAIELQWVAFGGGHLTANGDIKLDIDDFYLKLDVDSPKFN	

			FNKWHLEAGQRAAKGSKRIVFTAKSAEKVLFSGSTNFHSKAENNKISYSGNGQ<br>VRIGDKAHAFNFRSSRQNLIQDANKEIGVEYNLDFEIAGHGSLHNILKVTNKEL<br>HALGKQCSEGKPNCAVVEIKSKVSAADAKETTHDLVFLVDLKSVGVDTGVAF<br>TAETVRRGFWLIDEQASLTLSHNGETTYKYKGYLKESGSGFTLTLPSRVIAAEV<br>KLSSDVKPNHSKQQISASVWLDKTRLPNSFSSVSILLEEIEDKNTDKYVSQLRFT<br>HPNLEKDLTVKGYVQIGLENKLFDSNLEIDIFKQKNQKISISSTVVEQKQNDVV<br>KYLSTLDVKSKGHELDVTGRGEATVKPSLIALQSVLKYKKDKRIKESEFQFEVS<br>TEKLLVHVKVPNHHLLHIDANTKINDKHASGDASVHIIGLPTSVIHIEGENKGFP<br>VVKGTISSEGTPNKLELIADLSDGLLVEADFISESGKKELFYTFLSGKKDSRKPE<br>FRWSVENIQSALEPHKNDIQEVLNKLKEISDEAGNEITKESSRLADSLKAGLPNF<br>RRFVNTYETQLKALKEEIANDKVLKEISENWKEVIGDAAEVVSTLVNGILVTID<br>ALLKTLNELAESVLDALKKSLPALKDSYKQAVDAIVGIAKSLTQSLVNILSSAA<br>EILKKHEADIKEYLSVLADLANDVGKFVTKITGVIYEGVVEFSKPIKEKLDGLK<br>FGVAIEFGKVVEQLQNLIVPQELLAFAQEIVSELKETTLTPEIQDLLQAIEKYLE<br>KVSKKKDADVEKELKLIFEKAIDAVESVINFVVSEITGGDHTKDLYDINIPTVLP<br>SFIQLPRVFSVRFSPLIYLVSNGVPCLSDLLASYRPSLRFDNIIPPYDATAILLNSH<br>HFFTFDRRHLTFKGICSYILAQDVQDGNFTIIANIEGGSLKSIIVSDQATTFELAS<br>DKSLLVNGRPTEYPADEGEFHAWREYNRVGIQTKAGVKVTCETSIELCTFEIN<br>GFYFGKTRGLLGTINNEPWDDFTKPDGQVASKANEFGNAWKVDAQCANVDG<br>VDHHEHSIKVEECEEVFSKASLLSPCSLFLDPAPYLEACSHIAHEATTKEEKQLA<br>ACRTAAAYVQACSVENVFVSVPPHCVHCSVNGDAAIDIGQSFSVKVPQKSADI<br>LIVLEQVTGNAETVKDFVSPIVSQLTQELSSRGISDVWISLLGYGAPGQEYPHL<br>YTSSGGKLSYDGKQKNIQFGERKVLGPFPFDNLTESIDWLDEFTNQAFHLITTA<br>DTILDYPFRPGAAKSIIYVLDTSCETTLFLKHLPVKALKLKDAIGSPGIVLHLVT<br>NVDSVQSKHIVGFDTNHAYYNQEGKKRVVSEVTGNEKAALKISETACSQIALA<br>TSGTVFNKNNLKQTKKFVAQHIADSLTNVELTQDCKCLPVEGIHTRAVCAVTG	

			AREKEHLSVKGVKGTKGVKG	
3	LMI_GLEAN_101369<br>68	myosin heavy chain, muscle-like [Apis florea] XP_003695415	PKKTEGEDPDPTPYLFVSLEQKRIDQTKPYDAKKACWVPDEKEGYVLGEIKAT<br>KGDLVTVTLPGGEEKVLKKDQLSQVNPPKFEKVEDMADLTYLNEAAVLHNLR<br>QRYYCKLIYEKNFKKDQVSQVNPPKYEKCEDMSNLTYLNDASVLHNLKQRY<br>YHKLIYTYSGLFCVAINPYKRFPVYTNRCAKLYRGKRRNEVPPHIFAISDGAYV<br>NMLTNRENQSMLITGESGAGKTENTKKVISYFATVGASSKKDEQQSSKKGTLE<br>DQVVQTNPVLEAFGNAKTVRNDNSSRFGKFIRIHFGPSGKLAGADIETYLLEKA<br>RVISQQPLERSYHIFYQMMSGAVAGLKEMCLLTDNIQDYYFVSQGKTTIPGVD<br>DGEELQLTDGKITIPNVDDGEEMQLTDEAFDVLGFTQEEKNDVYKITASVMH<br>MGGMKFKQRGREEQAEADGTAEGERVAKLLMTSAEDLYKNLLKPRIKVGNE<br>FVTQGRNKDQVAYSVGAMSKAMFDRLFKWLVKKCNETLDTKQKRQHFIGVL<br>DIAGFEIFDYNGFEQLCINFTNEKLQQFFNHHMFVLEQEEYKAEGINWAFIDFG<br>MDLLACIELIEKFNGFEQLCINFTNEKLQQFFNHHMFVLEQEEYKTEGINWTFI<br>DFGMDLQACIELIEKFNSFEQLCINFTNEKLQQFFNHHMFVLEQEEYKTEGIEW<br>AFIDFGMDLQACIELIEKFNSFEQLCINFTNEKLQQFFNHHMFVLEQEEYQREGI<br>EWTFIDFGMDLQACIDLIEKPMGILSILEEESMFPKATDKTFEEKLMNNHLGKS<br>PNFMKPKPPKPGQPAAHFAIAHYAGTVSYNITGWLEKNKDPLNDTVVDQFKK<br>SGNKLLVEIFADHPGQSGGAEAAGGGKGGRGKKGGGFATVSSSYKEQLNNLM<br>TTLRSTQPHFVRCIIPNELKQPGMIDSHLVMHQLTCNGVLEGIRICRKGFPNRM<br>VYPDFKLRYMILAPAIMQAEKEPKNAAAKCLESVGLDPDSYRIGHTKARILLN<br>HQYKILAPQAVDALKGDDPKKMAQTILDAVNLDTESYRLSHQGIYKILCAHLV<br>KEPIAAEKATQIILDHVNMDAEQYRMGKTKVFFRAGILGQMEELRDERLGKIIS<br>WMQAWIRGYLARKEYKKLQEQRVALQVVQRNLRKYLQLRTWPWWKLWQK<br>VKPLLNVTRIEDEIKKLEEKAQKAQEAFEREEKARKEVEALNSKLLEEKTQLL<br>AQLEGEKGSLGEVQERANKLQAQKADLESQLRDTQDRLQQEEDARNQLFQSK<br>KKLEQEISGLKKDVEDLELAVQKAEQDKATKDHQIRNLNDEIAHQDELINKLN	77

			KEKKNQGDINQKTAEELQAAEDKVNHLNKVKAKLEQTLDELEDSLEREKKLR GDVEKAKRKVEGDLKLTQEAVADLERNKKELEQTIQRKDKEISSLAAKLEDE QSLVSKLQKQIKELQARIEELEEEVEAERQARAKAEKQRADLARELEELGERL EEAGGATSAQIELNKKREAELSKLRRDLEEANIQHESTLANLRKKHNDAVAEM GEQIDQLNKLKAKAEKDKAQFASELNDLRAGVDHLSNEKAAAEKVAKQLQH QLNEVQGKLDEANRTLNDFDAAKKKLSIENSDLLRQLEEAESQVSQLSKLKISL TTQLEDTKRLADEESRERATLLGKFRNLEHDLDNVREQLEEEAEAKADIQRQL SKANAEAQLWRSKYESEGVARAEELEEAKRKLQARLAEAEETIESLNQKVVA LEKTKQRLSTEVEDLQLEVDRATALANAAEKKQKAFDKIIGEWKLKVDDLAA ELDASXXXXXXXXSQGAYEEGQEQLEAVRRENKNLADEVKDLLDQIGEGGR NIHEIEKARKRLEAEKDELQAALEEAEAALEQEENKVLRSQLELSQVRQEIDRR IQEKEEEFENTRKNHQRALDSMQASLEAEAKGKAEALRMKKKLEADINELEIA LDHANKANAEAQKNIKRYQQQLKDIQTALEEEQRARDDARELLGISERRANA LQNELEESRTLLEQADRGRRQAEQELSDAHEQLNELSAQNTSISAAKRKLESEL QTLHSDLDELLNEAKNSEEKAKKAMVDAARLADELRAEQDHAQTQEKLRKA LEQQIKELQVRLDEAEANALKGGKKAIQKLEQRVRELENELDGEQRRHADAQ KNLRKSERRIKELSFQAEEDRKNHERMQDLVDKLQQKIKTYKRQIEEAEEIAA LNLAKFRKAQQELEEAEERADLAEQAIAKYRAKGRGGSAARALSPPPHRPAIR PQLDGNAFPPRFDLHPE	
4	LMI_GLEAN_101432 05	spectrin alpha chain-like [Apis mellifera] XP_006558458	MDQITPKEVKILETAEDIQERREQVLHRYSDFKQEARSKREKLEDSRRFQYFKR DADELESWINEKLQAASDESYKDPTNLQAKIQKHQAFEAEVAAHSNAIVSLDN TGMEMINQGHFASDIIRRRLDELHRLWELLLSKLAEKGMKLQQALVLVQFLR QCDEVMFWINDKETFVTTDEFGHDLEHVEVLQRKFDEFQKDMASQEYRVTEV NELADKLIMEGHPERDTISQRKEGLNEAWMHLKQLALMRQEKLFGAHEIQRF NRDADETVAWISEKDVVLSSDDYGRDLASVQTLQRKHEGVERDLAALEDKVS TLGQEADRLCGIHADHADQIQAKRAEIVAYWESLTRKAEERRQKLDESYYLH	50

			RFLADFRDLISWINDMKAIISADELAKDVAGAEALLERHQEHKGEIDAREDSFR<br>ATAEAGQLLLKHGHYASEEVKDKLGVLSSEKASLLSMWEERRILYEQCMDLQ<br>LFYRDTEQADTWMAKQEAFLANEDLGDSLDSVEALIKKHEDFEKSLAAQEEKI<br>KALDEFATKLIEGQHYAADDVAQRRAMLLERRSALLEKSAQRRAILEDSNRFQ<br>QFERDCDETKGWINEKLKFATDDSYLDPTNLNGKVQKHQNFEQELNANKSRM<br>DEIASTGQELIEANHYASDRIQARMDEILQLWETLVSETEKKGSKLQEASQQQ<br>QFNRTVEDVELWLSEIEGQLMSEDYGKDLTSVQNLQKKHALLEADVASHQDR<br>IEGIRVAAAQFVERGHFDADNIKAKELALTERYSALQRPMNIRKQRLLDSLQV<br>QQLFRDIEDEEAWIREKEPVAASTNRGRDLIGVQNLIKKHQAVLAEINNHENRI<br>TAVYQSGQQMLDEGHFASDEIKQRVATLNEHWTQLKDKALQRKQDLEDSLQ<br>AHQYFADANEAESWMKEKEPIVCNTDYGKDEDSSEALLKKHEALVSDLEAFG<br>NTILSLREQAQSCRQQETPVIDVSGKECVMALYDYTEKSPREVSMKKGDVLTL<br>LNSNNKDWWKVEVNDRQGFVPAAYVKKIEAGLSASQQNLADKNSIAARQNQ<br>IEAQYDQLLSLARERQNKLNETVKAYVLVREAAELATWIKDKESYAEVADVG<br>EDLEQVEVMQKKFDDFQADLKANEVRLAEMNEIAMQLMSLGQTEAALKIQT<br>QIEDLNQKWTSLQQLTSERATQLGSAHEVQRFHRDVDETKDWIQEKDEALNN<br>EDXXXSVQALQRKHEGLERDLAALGDKIRQLDETANRLMQTHPETADQTYA<br>KQKEINEEWTQLTAKANSRKEKLLDSYDLQRFLSDYRDLMSWINSMMGLVSS<br>DELANDVTGAEALLERHQEHRTEIDARAGTFQAFDLFGQQLLHSGHYASVEIQ<br>EKLESMAEARQELEKAWIARRMQLDQCLELQLFYRDCEQAENWMSAREAFL<br>AAEEVDSKGDNVEALIKKHEDFDKAINAHEEKIAALQTLADQLIGADHYASKQ<br>IDEKRCQVLDRWRHLKEALIEKRSRLGESQTLQQFSRDADEMENWIAEKLQLA<br>TEESYKDPANIQSKHQKHQAFEAELAANADRIQSVIAMGQNLIDKHQCAGSEE<br>AVQARLASIADQWEYLTQKTTEKTVKLKEANKQRTYIAAVKDLDFWLGEVES<br>LLTSEDSGKDLASVQNLIKKHQLVEADIQAHEDRIKDMNAQADSLIESGQFDT<br>ASIQEKRQSINERYERIKNLAAHRQARLNEANTLHQFFRDIADEESWIKEKKLL	

			VGSDDYGRDLTGVQNLKKKHKRLETELASHEPAIQAVQEAGEKLMDVSNLG VPEIEQRLKLLNQAWAELKQMAATRGQKLDESLTYQQFLAKVEEEEAWISEK QQLLGVEDYGDTMAAVQGLLKKHDAFETDFAAHGDRCADICAAGQKLIADN NHHMESIAQRCQQLQVKLDHLASLAARRKLKLMENSAYLQFMWKADVVES WIADKETHVRSEEFGRDLSTVQTLLTKQETFDAGLHAFEHEGIQNITSLKEQLV SASHDQTPAILKRHADVIARWQKLLADSDARKQKLLRMQEQFRQIEELYLTFA KKASAFNSWFENAEEDLTDPVRCNSIEEIRALREAHQQFQASLSSAQADFDAL AKLDQQIKNFNVGPNPYTWFTMEALEDTWRNLQKIIKERDIELSKEAQRQEEN DKLRKEFAKHANAFHQWLTETRASMMEGSGSLEQQLEATKRKAGEVRARRS DLKRIEDLGAILEEHLILDNRYTEHSTVGLAQQWDQLDQLGMRMQHNLEQQI QARNQSGVSEDALKEFSMMFKHFDKDKSGRLNHQEFKSCLRALGYDLPMVEE GQPDHDFDAILDIVDPNRDGYVSLQEYMAFMISKETENVQSSEEIENAFRAITA GDRPYVTKEELYANLTKEMADYCVARMKPYVDPKTERPIQGALDYIEFTRTLF QN	
5	LMI_GLEAN_101828 24	Muscle M-line assembly protein unc-89 [Cerapachys biroi] EZA59129	LQELSQQLGDDKSLSEHLKLPIQRINDYQLLLKELVKYSSRLGEDTSDLQKALD LMLAVPHRADDIKFISNIEGYHGNIHKLGRLLKHDWFTVTDKEGKHKERYLFL FKARILVCKVRRISEDRSVFVLKDIVKLPDVEVEDHKDNLRVFELHHKSTGFGN FPLTIAAHSDTIKDIWLKEIRQYSTDKFALAEHAADDLQLKSHIEDTVDTISQEK TLKKTSVTEVISEKSKVVNGIVKEHTQDLPQENQREPDTKGKETIKKPLDSEDQ DNRGPPKKQKTTNENNMDQYMSFESRRISSSSRSVHEYSSVSSSHLSEEIYMES SNVSSSESMRVASSATQSIAGGDTVVRLALTSSDGRPVFTETIEGCNVEPGQNA IFQCKVASDTPTNMTWLKDNRPMDDKLADRIKMESKDSSSFKLEIQHCRESDT GIYTAKASNVIGSSTCTAQLVVQDISEEERAQRAAANCPSFLIRLKDTELLENTY LRFMVKVQGTPNPEVTFYKDGKLIEKGSDHIQVIRDKAESGYYELVITDVHQN DAGKYSCTAKNKYGEVSSEATVTVTTEKKIFDGLESAGLLQPGEEPKFTWLRD GKPFDPEERFKVLFKDEEDSLALVFQHVKPEDAGLYTCVASTTSGKISCSAELT	49

			VQGSVNQLLKEPEVPKIVTETKATEVSVGGSAMLELKINGFPKPDVKWYKDN<br>KEVTAGGRVRFLYEDDESISLIIKGVTPDDAGKYKIVAKNDLGEDSAEVDLMV<br>KAPPKIKTKMQDASCMTDQPFKMTVEVEGVPEPEIKWYKDGQQIVQTDRIKII<br>KETSETYTLSITSTKLEDSGSYSIVATNEMSQTSEFWKFVVHSGPQFLKTMTKS<br>VEAREDDTITFEVKVQGDPKPDVKWLKNGEELKADGKHIIIEVDGLTHTLTIKG<br>TTRNDSGTYSCEVINDHGWKKDEGVLNVRCAPEFKKDLSDLQANEGDTNIEF<br>NVKVDAYPKPKVKWFLGDIDITEKKNEFTCTEEEGTFKLVIKEVTTELSGKYTC<br>KVVNDLGTSESSSNLTVNFKPKFKKPLKDVEVDEGDSLTLTIECDAYPEPNVK<br>WYKDGNEVSADARIKITRDSTRKESYSMSFTLVKSSDAGEYEARAENKMGTSS<br>TKSTVKVSKKKEEEYLKDKFQEETQETEVGTLEEKYKLKIEEEIIEEHFSPESVK<br>TKEIFEEKGTPLNAVSSKVTLERQSITYSRGDGDTVTISVASTTHHEVATVSDN<br>GLEGPYAVHTISETKSTRIVCEELDGPEEERGTLLKKHSVTHEEIEDSQGGLATV<br>EEPESEEQHVKRLKIDVNRGVSIVSLSEDEPTSRASLSPGPISEENLEIHRIEIGNV<br>FPGDTKEIANETNKGKLWQTEEAVAVPNCQQKTLISDVSTEKDEGNLYEKQE<br>MDVIENKPQNITGSDDIINEMPKPLKGKEIISGTKEDIVNGVPENQLHRTMSQEE<br>AQKASSEEDPEVEALLKRIQKQRSVLEEILDKEGERKIEGTVLRPRLPSHSNGH<br>GKREDRRKFDADEKTFDAAPPEINSSNLEDREIFESLGTVFEVKANGIPKPEVK<br>WYKDGEELKPSGRITISEDGDTYKLELKDAQTSDAGVYKVKITNRLGEKSQEA<br>KLSLKPVNEFRKPKIKTPLKDVAVCKNEDSELTCVFIGDPVPTVTWFHNAEKIT<br>EDDRHIFTSATKDIGDGLKETTFTLKIPSGQHGDTGQYKVKAANKFGEDETSA<br>RLDILLIPEIDALTDITKIPYEDTVFEVKILANPKPTVTWTRNGEKLSNSDHTAIS<br>EDVEKEIYKLEIKNIGLGDDGVYMVTASNSQGERNQQAKLTVHTEPPSLVKNL<br>EDRSVKDYEEVEYRVRVNGVPKPKITWYKDGKELQSGEHVVIETDSEALVVSS<br>LSIQHFQESDVGVYKVKAANLVGEAESSSKLSMTQLPPSFARPLDRAAEVDEG<br>DILDLKCKLDGSPIPTVQWFKDGAPLHADEHVKLTALPDGTVRLLIEGVKPTD<br>CGAYKLVAKNKNGEHAAICAVAVKPNNRKPSFSKPLEGAKCVVGEPLKLEAQ	

			VAAFPAPEVKWFKDGQPVRPSQAVNFVNQPGGIIGLHIDSVKPEDAGVYTVTV<br>SNRLGDITGTAKVEVEPREKKPTFQVPLLPATVVEGFPARFEVKVVGHPPATLK<br>UCLFFCVLQDSQWERVPDGQHAKITQLPDGTCALIIDKVKPEDAGEYEVIATN<br>EKGSVPSKAQLDVTARARDAPEEKPAFLHGLRDVTVEEGDSLTLSAPFIGNPIP<br>DVAWSKDGTTLSPSDRILLTCDGRKVGLEINPASLDDGGKYTCKLINPLGEDET<br>TGVATVRKIYKKPNFTQRFTDLQQRPTFDARFPARVTGIPKPEITWYFNDKPIH<br>DSDKYKIKRDGETCCLYVKDCQPSDSGRYKCKATNRDGEDSCEANLDVVEKI<br>ADTQRTEPPSFMKKIGDTEVYKGMTAKFTACAAGIPEPDFEWYRNNERLFPSD<br>RIRMEREGSGLLRLIIDSVDPVDIGRYRLKIFNPHGEASCEAELAFDSLDMHPKR<br>PLGYLYTDFDKYQSSAAPLPLPDKPIICRMSDRRLTLSWRPSIPIGPTVPVTYRV<br>EMSEQPDGEWFTARTGIIGCACDIHNLQPFQDYKFRIRVENRYGISDPSPYAVT<br>YRARLEPEPPKFFPYLEPGIDFRPETSPYFPKDFDIEKPPHDGYAQAPRFLRQEH<br>DTQYGIKNHNCNLFWFVYGYPKPKMTYYFNDEVIEMGGRYDSSYTRNGQAT<br>LFINRMLDRDVGMYEAVATNEHGEARQRVRLDIAEYPTFIKRPEETIVMLRKS<br>GRLEARVTGVPYPDIKWYKDWQPLTASSRIKIQFREPDTCILIINDAINKDEGLY<br>SISARNVAGSVSSSAMIRIEESEQEYGYLTYSKGRNIKPKAKPFEDLYDIGNELG<br>RGTQGVTYHAVERLTGRNYAAKIMHGKSDLRPFMNNELEILNVLNHPKIIRLH<br>DAYETRNSLTLVTELAGGGELLSNLTKERYTTESDIAGYIRQLLWGLEHMHDQ<br>NIAHLGLTIGDLLIAHQGGDDLKICDFGLSRRINFGKLASLDYGLSFYFRNXVA<br>PEVVNGDGVSTSADMWSVGIITYILLSGISPFRGINDRETLTRIKEGKWEFIEEW<br>FSKLSMEARDFISKLLVYQADGRMDVKAALKHPWLLRADKMPADEYQITTDR<br>LKIYYDTYRDWYNNAACRTWYRRKPLAGAFSHPSRMVYPPGHIYTPEPTPEPQ<br>RRESXXXXXXXXXEPIDYEIGMPKSESHYQYGPDTYLLQLRDVDFPVRLREY<br>MKVAANRAPGYSAHLTETAPYDWRTPVIRERRRFTDVMDEEIDDERKERINK<br>YGSGEVYSLRRLRHELGTRLDGHAEAEAIIEYKREGQPPFFREKPQSPLPILEDQ<br>PAELSCLVVGDPKPVVQWFKNDQVMLESNRVKVREDEEGRSFLRLEPALHID	

			VGIYKVVARNRVGQAISRTRIVLASVPSSPDSPEASEVSDTEVLLRWKQPKDDG NSAILCYSLQYKEADNVDWLDIANNIDHEFFLVHDLKPATNYQFRLAAQNRIG WSEKGIPTELIKTKESGSTKIQVTRAMKHLQQITESGQAITVEDSKPKLDYSVE KNPIGWTTEGSLTEKYSFVSELARGRFSIVVKGIEKSSDRVVVAKLLEIKPETEE QVNREFEALRSLRHERIASLEAAYKISGSGVAALVQEKLQGADVLTYLSSKHE YTEQQVATIITQVLDGLQYLHWRGYCHLDLQPDNIVMASVRSVQVKLVDLGS AQRVSKLGTVIQKVGETEYTAPEILNDEPAYPQSDIWSVGVITYVLLSGVSPFV GKDTEETRQNVTFVRYRFEHLYKEITQEATRFLMLIFKRTPSKRPTAEECHEHR WLIPSEITIKKRERAVFLGNRLKEFSEAYHSKKTQDAIKSDSLTGAFGGSGKAA LTRSSSIQEELYTTF	
6	LMI_GLEAN_100884 60	filamin-A isoform X3 [Tribolium castaneum],XP_008199793	ERELAEDAQWKRIQQNTFTRWANEHLKTVNKHIGNLETDLCDGLRLITLIEVL SGKRLPKHNKRPNFRSQKLENVSVALKFLEHDEAIRIVNIDSTDIVDCKLKLILG LIWTLILHYSISLPMWEGEDDSQHDGKGATPKQRLMHWIQSKVPDLPITNFTSD WNDGRAVGALVDAVAPGLCPDWQDWNPNDSVQNAAEAMGLADDWLNVRQ LIKPEEMVNPNIDEMSMMTYLSQYPNAKLKPGAPLRPRTNPNRVRAYGPGIEP MGPVVGAPANFTVETFSAGKGKVEVTVEDPKGNKEPVDIRFNNDRNLTYSVS YVPKVEGNHKVTVTFGGREIPKSPYNVLVEGHAGDPTKVTASGPGLQPEGVMI NRPTYFDISTKDAGRGVPEVIILDPAGAKTSVPVKVRQISPDLWRCEYVSSAIGL HSVNVFFAGKPIPNSPFGVRVSPVSDAKKVRASGRGLQPSGVRVGDDAAFRIY TEGAGEGTPAVRIIGPGGVNEQVNLKKVDGTVYEAVYHPRKEGRYVVMVTFA GQEIPRSPFEVNVGPYKETQIRAYGPGLAGGVVGYPALFTVETNGETGALGFSI EGPSQAKIECNDNGDGSADVKYYPTAPGEYAVHILCDNEDIPKSPYISQILPNT DYFPEKVECYGPGLEKSVSKGKPVDFTIDTRRAGSAPLDIQVLDANCKNIDVK LKDNHDGTYKAEYVPKGGKNVVQVNYGGVSTRNSPYRVFVTEPLNPAKVQV FGPGVEKGVKSNTPTHFNIDCREAGQGDIQLSLKSETGIDVPLDISDNEDGTFTV DYCVAQPGLYTATIMYGGQMIPQSPIKIQVQPHVDISKIKVDGLEPSVFVDSPT	48

			DFVVDSRAIAKRDDGKVTCTITNPSGTKTENLITPMADGTYRISYTPFEEGRHTI<br>DILYDGLPIPGSPFVVNVRRGSDPKKCRAFGPGLEMGLVDKPNVFTVETKGAG<br>TGGLGLAIEGPSEAKMTCKDNRDGSCSVEYIPSEPGDYDVSIKFADQHIPGSPF<br>KVPVEKSVDASLVKAYGPGLDPNKCRAEIPVSFKIDTSKSGKAPLDVKLRSER<br>GSLQHQPMIKDLGGGIYEVTYLPPPEGSTLKGRITWDGKDIPNSPFQIKVRPPAE<br>PERVKVDGPGVSSKGIPASIPAEFTIDTSQAGYGDLEVQVKGPDGYPRKVKVTE<br>NDDGTFKAVYVPDDCGRYKVTVKYDGKEVRNSPFQVQAYATGSVKKCKITE<br>GIQRVLTSGEEYCITVNAKNAGYGAVTCRIRSTSGSDLDIDIEDNGDGTFSIYYT<br>VKDAGDYTLSVKFGGQPVPDGFYTFTAGPEDYVKQTVVQETQRVQKTTTSQQ<br>YRAVQLDNIPLPTTGGHVTAEIKMPSGNIDKPIIEDNRDGTVSIKYDPREEGLHE<br>LSVKYNGEHVQGSPFKFHVDSITSGYVTAYGPGLTHGVSGEPSNFTISTKGAGA<br>GGLSLAVEGPSKAEISCHDNKDGTVSVSYLPTAPGEYKISVKFGEKHIKGSPYN<br>AKITGEGRKRNQISVGSCSEVSFPGKVTDADIRSLNASIQAPSGLEEPCFLKRLP<br>NDNIGISFTPREVGEHIVSVKRLGKHITNSPFKITVGEREVGDAKKVKVTGAGL<br>TEGKTQTENSFTVDTRSAGYGGLSLSIEGPSKAEIQCKDNEDGTLNISYKPTEPG<br>YYIVNLKFADHHVEGSPFTVKVTGEGSNRQREKIQRQREAVPITEVGSECKLTF<br>KMPGITAFDLAATVTSPGGVTEDAEINEVEDGLYAVHFVPKELGVHTVSVKYK<br>EMHIPGSPFQFTVGPLKDGGAHRVHAGGPGLERGEQGQPCEFNVWTREAGAG<br>SLAISVEGPSKAEIDFKDRKDGSCYVSYVVSEPGEYRVGIKFNDQHIPDSPYKLF<br>ISPAMGDAHKLEVFQFPSSGVQADKPTAFLVRKNGAKGELDAKIVAPSGIEDD<br>CFIQSIDSETYSVRFMARENGIHNIHCKFNGVHIPGSPFRIKVGKEDADPAAVHA<br>HGNGLGEIKTGQKTDFLVDTCNAGAGTLAVTIDGPSKVSMDCTEVEEGYKVR<br>YTPLVPGDYYISIKYNGYHIVGSPFKVLCTGEDLAERGAQETSSVVVETVQKVS<br>KSKQTGPVLPLFKSDASKVTSKGMGLKKAYLQKQNMFTVHAGEAGNNILYV<br>GVYGPKSPCEEVFIKHLGRNNYQVNYVVRERGEYIVIVKWGDEHIPGSPFKVE<br>V	

7	LMI_GLEAN_101878<br>50	microtubule-actin cross-linking factor 1 isoform X6 [Nasonia vitripennis] XP_008203191	MEFDLDSSLKEWAKDKPLSILQLDPADRAVLRIADERDAIQKKTFTKWVNKHL<br>KKASRHVGDLFEDLRDGHNLISLLEVLSGEHLPRERGRMRFHMLQNVQIALDF<br>LRYRKIKLVNIRADDIVDGNPKLILGLIWTIILHFQISDIVVGQETNISARDALLR<br>WARRSTAKYPGVRVSDFTSSWRDGLAFNALIHRNRPDLIDWRSIRSRVVRERL<br>ETAFTVAEREYGVTRLLDPEDVDTPEPDEKSLITYISSLYDVFPEPPPIHPLYDSE<br>SQQRLSEYRELASSLHLWMREKISIMLDRTFPSTLIEMKKLAAESNRFRSDEIPP<br>RQRDKQKLHLIYRELEKYFEAVGEVDIESELQYEVIDKNWNRLMIAHQDRDH<br>AILEEIKRLERLQRLAEKVHREVKQVDSRLGELEVRVEEEARRLDRLHPLDAK<br>HNVDLLETELRGTEDAINSMFTDTQVLREGRYPQAPELHKRVQKLHQRWVSL<br>RQLVHTHLVSPLASLSFPVEERTVTRQTRTVLETRLVDTNVHFRQLQECSEWC<br>RTKLKQLQDAEYGSDLPSVQNELELHQREHRIIEQFQSKVEHCSLAKSNFHGEE<br>LQLYNQHLGQLQKLYNELLVTSNKRLSDLEVLQDFLQSATNELVWLNDKEET<br>EVTRDWSDKSLNVPALEQYYESLMSELEKREIQFSAVQDRGESLVLQHHPAAK<br>CIEAYMAAMQTQWAWLLQLTLCLETHLKHAAHYQQFYRDLHDAEAWIAKR<br>DELLNTIYSKSDFTLDEGERLLKGMQELRDELNQYTTVIQRIAERATEVIPLKQ<br>RRQPVTRPTAVTAVCSYKQVNIIIEKGEKCVLHDNSGRVKWRVSNSSGQESTV<br>PGVCFLIPPPDKDAIDAADRLRRQFDRSTALWQKKQLRMRQNMIFATIKVVKS<br>WDLAQFLAMGQEQRSAIRKALNEDADKLLQEGDPADPQLRRLKREMEEVNR<br>LFDEFERRARDEEDSKSTSRSFNDSASKLTAALDEAERTLNARTAAPLPRDVDS<br>LEHLVIEHKDFETRLQALGPDVEKLQSTFRSISRKTPEQQSRLDKCISKWNNLW<br>STSHLYIERLKCIEIVLSGLEEATNIVTEFELKLASYKELPSDVEALRAVHEDLL<br>NLQNSVAQHQMVIDQLSEDVLNARRLVEKSRPNHRGPHSDLERLEGEVGRLT<br>NRWGNVCSQLVDRLRSCEAAYGLLQTYSNSYEKEVAWVDESYGKLSGLAPIE<br>DRAKEQLEPTRVLYNNVLEKAPAIEQVNVVGGRFIREAKIYDLRLQHYASWLV<br>DEVHPSMDTVIGRSRANGDSRADRIPGATAVAAQLDALNHRYRALLSLLYDR<br>LKQIATLCSNEPSVQVLVSQMEPRELKTFRTEFNIYESAEESQFNSRQYTTKQA	45

			TEVDGGRPGVGGTDVSPNQTVGVSGNRGSSMHFSEIRSLRRIRQADEGTDVSD<br>LLGITDPATGRVLTVGEAIALRVLDVRTGQVAGTPLREAVKCGLVEPTLATRL<br>LGPCGVGQLSLLEAIQRELADAERGVPSAGPEQAVEEKYKHCDETLSKLLLWL<br>SNTENRIASQGNVAENTEDLRNQINTLKQVKDDLEAHSRPIAACLDLVRQLVS<br>TGRDVLSADEVSTVEKNGRSLKTRFDCASERTERLLRKLGTARDELSKFKSELS<br>TFNSWLDKACRQLEDKERSLSNLNKLNSSTDSTKEFVSDVIAHQADLRFITMA<br>AQKFSDESKEYLSILNDFRGGLPERLPHVEHPAKSVVSHEVSEASVRYRDLLSR<br>AEGLSDRLSGVGGRQREHQDALEKARAWMREAEPRAHKTIAEPAGADPRTLE<br>EQLIRARALSSEFVAQGRLIDAAKLSLDALLRSLEGQQTQADVASLEAPVHDLE<br>DKYKQLSEALLEKCQELDTALVQSQSVQDALDSLTQWLNSAENNAKSLQRPA<br>SLHKERLDEQLREHRLLQADVDQHRPSIESVSQSAQELIATASNPRLAKKIDAK<br>LKDVTSRFEKLSERTLRRTELLEDTLAALTSFSTQASKLETWCSQTVEALEACA<br>GRLDAEELQARVDTLAAQKEAHRPEFEETVRAGRTLVGKKDVTDTVALRDRI<br>KYLESQWKELGLQLEEKQRLGKARVDQMNAYERLHEQVMDWLTMMESRVQ<br>RLDPVAVDIDVLRTQIEELKPLHKEYREYVGTVDRLNDLGNAYDNLLRGDRPE<br>SPQRRRSSAYSPTKKTSVTASPQFLRHVSFTDSRFQKLAELSPVQQQLTEVNNR<br>YGLLGVRLSDRQSELDSTREEVRRHMDSLRTLSAFLDKVQRSLPKEVVPQTKE<br>EANKAAKSIKAVMEDMYEKQSLLDNTRGQVADLLKRKPSVQGSDTLHDEVT<br>DVVTRWKSINDRCKDRIKLMDDIKEFYDTHDSLSAWLGAKDRMMTVLGPISS<br>DSRMVQSQVQQVQVLREEFRTQQPQLSHLEEVGSAVLSKLDMSAPDAQRISSK<br>LTSLQQRWAELLARLQERADSLGAAADTSREFDAGLLRLRDALQALSDHLDE<br>VPLDRDPEEQLRKVENLERQLEGQRPLLADAESTGAQLCDVLSDPASRAEIQG<br>KLGAVGRQYNTLQKKMDHRKAEIEGNLRDGRQFEASCARTLGWLSDELGAM<br>SERLLVSADREVLQQQLDQHEPIYKDVMSKEHEVIMLLNKGRDMLARSSQMQ<br>RSDSRNLQRDIDKIQSQWDRLRKDVMERHTRLQTCMEHCRKYYKAQEAFLP<br>WLSQAETKLEALAPASFKRKDIERQLKDLSAFRNEVWKRSGEFENNRTLGDTF	

			VAACDIDKDVVKGELAQMKKRWDRLNNELLERTQSLEDTSRRLTDFSENLRD<br>LTHSLQRCEDRLASHDALGGAAKDPKLLDRIKTLREETTALRKPLSAVRQQAG<br>DLCHEAGELGVDASHLQDEVDGIGDRLDDLQAKLDDRCSELQSAATAVTQFT<br>DQVKGLSHDLSNLEQELENMKPPGRDLKTVHTQQDEITKFIRKLSRVSDNVAD<br>TVSAGERLVDSGFAPDTAATREQADLLRRQLARLEERGRAREEDLAVTLDRLE<br>TFYQTHSGIIEDIRHVSDQLHHLKPVGSEVDTIRAQQEEFRTFRDKEVEPLASRI<br>DECNRLGQGLIQSASGGVSTVNLEKDLEKMNDKWNDLKERLNERDRKLDVG<br>LLQSGKFQEALDGLSKWLTDTEEMVANQKPPSADYKVVKAQLQEQKFLKKM<br>LLDRQNSMSSLFAMGNEVVAGADPAERKAIERQLHELMQRFDNLTENATQR<br>MNALEQAMAVAKDFQDRLVPIVEWLDKTEKKVKDMELVPTDEEKIQQRIKEH<br>DALHTEILRHKTDLSELTDVAGELMGLVGEDEAAGLADRLQVAADRYTALVD<br>ASEAVGALLSQSCSGLRHLVLGYQDLQAWMDGADQRLSKFRILAVHTEKLLA<br>QMEDLADLTEEVANHQSQVDGTVDTGLELMKHISSDEALALKDKLDSLQRRF<br>NDLTSRGADMLKHAQEALPLVQQFHNCHNRLVDWMMGAEAQLQAAEPREE<br>DIQRLELDIQEFRPVLEMINLVGPQLYQISPGEGASTIEGLVTRDNRRFDAIAEQI<br>QRKGERIQLSKQRSLEVIGDIDELLDWFREVEGQIREAEPPSSEPDVIRVQLKEH<br>KALNDDISSQKGRVRDVLSTAKKVLRESAQHEDTSTIREKMEDLREMMDTVS<br>GLSSDRLGVLEQALALAEHFNETHDGLASWLSDTEQQLAVLAAMPTLRPDLIA<br>QQQDRNEMLMQSINEHKPLVEKLNKTGEALVKLVSDDEAAKVQEIVENDNSR<br>YAALRSDLRQRQQALEKALQESSQFSDKLEGMLRALGSTADQVHAAEPVSAH<br>PSRIRDQMEENTALVEDLDKRQEAYAAVKRAADDVISKAGNTSDPAVKDIKR<br>KLERLNSLWNEVQRATGERGRSLEDTLAVAERFWDELHNVMATLRDLQDSL<br>NSQEPAAVQPAAIQQQQEALQEIRHEIDQTKPEVDQCRQTGQELMSLCGEPDK<br>PEVKKHIEDLDNAWDNITALYAKREENLIDAMEKAMEFHETLQNLVEFLDSAE<br>DRFSSMGALGSDIDAVKKQIEQLKDFKAEVDPHMVKVEALNRQAQELTERTS<br>AEQAAAIKEPLSAVNARWDDLLRGVVERQRQLENALLRLGQFQHALAELLV	

			WIDKTDSTLTELKPHAGDPQALEVELAKLKVLVNDIQAHQTSVDTLNDAGRQ<br>LIEQGQGSAEAGATQERLSQLNRRWRELLDRAAERQRELEEALKEAQQFSAEL<br>QDLLSWLGDVDAAIAASKPVGGLPETASEQLERFMEVFNELEQNRPKVETVLQ<br>QGNEYLKKSGANTGHLQHNLKTLKTRWDSVTARANDKKIKLEIALKEATEFH<br>DALQSFVDWLTNAEKTLGGLKPVSRVMETILGQIEEHKAFQKEVGVRRETML<br>NLDKKGTHLKYFSQKQDVILIKNLLISVQHRWERVVSKSAERTRALDLGYKEA<br>REFHDGWSALMTWLEDTERNLDELAEDAAAGNDPEKIKTRLAKHREFQRALA<br>GKQTAYDTTMRAGKSLKERAPKTDEPALRQMLTDLKARWTSVCAKSVDRQR<br>RLEEALLFSGQFKDAVQALLDWLHKVESTLSEQGPVHGDLDTVVALVEQHKT<br>FEEDLRSRQAQMESVHNTGRELQAKATAADAATIQAQLVELTTLWETVTVLS<br>RKKTQRLEEALKEAEKLHKAVNMLLEWLSDAEMRLRFTAALPEDEQDTRNQ<br>LAEHDKFMKEMMDKEKEKNATIALAQEILAKAHPDGAAVIKHWITIIQSRWEE<br>VFSWATQREQRLKDHLAALRDLEGLLEELLAWLAGLEGTLVDLEREPLPDDL<br>PTLEALVADHRQFMENTSKRQTEVDSVCKSRQVKLAEAQRDRKLSRPKTPSR<br>KRGRKEREKRMVRASPGREKAPELQPHIGPRFAAKGSKGAEPQFRNPRVKLL<br>WDRWRNVWLLAWERQRRLQERLAYLQELEKVKNFSWDDWRKRFLKFMNH<br>KKSRLTDLFRKMDKNNDGLIPREDFIDGIMKTKFDTSRLEMNAVADLFDRNSE<br>GFIDWKEFIAALRPDWVEPTKPVTDAEKIHDEVKRLVMLCTCRQKFRVFQVGE<br>GKYRFGDSQKLRLVRILRSTVMVRVGGGWVALDEFLQKNDPCRAKGRTNIEL<br>REQFILAEGVSQSMTAFKPKASPGSGSGSTPQRSQSLAGPITKVRERSARSVPM<br>GRPSGRSSFSGGARTPDSLSDNESTLPRPLRKGSAPPRSSLTPGGSLPGSKAGSR<br>AGSRPGSRQGSKPPSRHGSTLSLDSTDEGTPSRIPVQRRTVGATTTPPARGGTSA<br>GSSRKLTAPVNGTPGGSRPRTPTGGSRPRTPTGLLSPASGVPTSGTKIARRSSGA<br>SDT	
8	LMI_GLEAN_101355<br>16	alpha-actinin, sarcomeric isoform X2<br>[Tribolium castaneum] XP_972324	MEQNGVKSAVVHTASFVTHRKVACGVYKTFTAWCNSHLRKAGTSIENIDEDF<br>RNGLKLMLLLEVISGETLPKPDRGKMRFHKIANVNKALDFIASKGVKLVSIGA	36

			EEIVDGNLKMTLGMIWTIILRFAIQDISVEEMTAKEGLLLWCQRKTAPYKNVNVQNFHLSFKDGLAFCALIHRHRPDLIDYHKLSKDNPLENLNTAFDVAEKYLDIPRMLDPDDLINTPKPDERAIMTYVSCYYHAFQGAQQAETAANRICKVLKVNQENERLMEEYERLASDLLEWIRRTMPWLNSRQTDNSLAGVQKKLEEYRTYRRKHKPPRVEQKAKLETNFNTLQTKLRLSNRPAYMPTEGKMVSDIANAWKGLETAEKAFEEWLLSEMMRLERLEHLAQKFKHKADIHEDWTRGKEEMLQSQDFRQCKLNELKALKKKHEAFESDLAAHQDRVEQIAAIAQELNTLEYHDSASVNARCQRICDQWDRLGSLTQRRRQALDEAERILEKIDILHLEFAKRAAPFNNWLDGTREDLVDMFIVHTMEEIQGLIDAHGQFKATLGEADKEYTAIVGLVREVEATVQKYQVPGGLENPYTTLTANDLTKKWTEVRQLVPQRDQTLQNELRKQQNNEMVRRQFAEKANQVGPWIERQLDAVTAIGMGLQSTELLPFEQQNRGSLEDQLHRLKEYEQGVYAYKPHIEELEKIHQAVQEGMIFENRYTQYTMETLRVGWEQLLTSINRNINEVENQILTRDSKGITQEQLNEFRSSFNHFDKNRTGRLTPEEFKSCLVSLGYSIGKDRQGDIDFQRILAVVDPNNTGYVHFDAFLDFMTRETTDTDTAEQVIDSFRILAGDKPYILPDELRRELPPDQAEYCIHRMPPYKGPNGVPGALDYMSFSTALYGESDL	
9	LMI_GLEAN_10062054	Paramyosin, long form [Zootermopsis nevadensis]KDR08790	RRKFQARIQEQEEHIETLLVKVNNLEKQKSRLQSEVEVLIIDLEKANNTARELQKRTEHLEKINIDIKTRLEETVSLYESSQRELRNKQTEIQRITHELDKTREHKETLIRENKKLSDDLHDAKNSLSDLTRRLHELELELRRLENEREELSAAYKEAEAGRKAEEQRAQRLSAEFGQFRHESERRLAEKDEEIEAIRKQTSIEIEQLNARVVEAETKLKTEVTRIKKKLQIQITELELSLDVANKNNIELQKTIKKQSLQLTELQAHYDEVQRQLAVTLDQYGIAQRKLQSIGAELEEVRGNYESALRAKRTAEQQYEESVSRINELTTINVNISAAKSKIEQELSTLAGDYDEVTRELKASDERYQRVQIELKRTVETLHEEQERIVKIEAIKKSLEIEVKNLTVRLEEVEANAIVGGKRIISKLEARLRDLELELDEEKRRHAETLKILRKKERTVKEVIIQQEEDQKNISLLQESLDKANQRIQIYKRQLQETEGMTQQSVTRVRRFQRELEAAEDRADTAESNLSLIRAKHRTFVTTS	33

			TVPGSQVYLVRETQ	
10	LMI_GLEAN_101374 18	Spectrin beta chain [Zootermopsis nevadensis] KDR16227	MTTDISIVRWDPSLQQEIVEDYEYDGGNSSSRLFERSRIKALADERESVQKKTF QKWVNSHLVRVSCRISDLYVDLRDGKMLLKLLEVLSGERLIQDITIEETDNQET KSAKDALLLWCQMKTAGYHNVNVRNFTTSWRDGLAFNAIIHKHCPDLIQFEK LSKSNAMYNLNNAFNVAEEKLGLTKLLDAEDVFVEQPDEKSIITYVVTYYHYF SKLKQETVQGKRIGKVVGIAMENDRSAQEYESLTSDLLRWIEATIESLGDRNFA NSLAGVQQQLAQFNTYRTVEKPPKFNRKASMRETWLSENQRLVSQDNFGFDL AAVEAAAKKHEAIETDIFAYEERVQAVVAVSQELEAENYHDIDRINARKDNVL RLWNYLLELLRARRLRLELSLQLQQNFQEMLYILDSMEELKLRLLSDDYGKHL MGVEDLLQKHSLVEADINVLGERVKAVVQQSQRFLDQEAAEGYRPCDPAIIVE RVQQLEDAYAELVKLAVERRARLEESRKLWQFYWDMADEENWIKEKEQIVS TGDIGHDLTTINLLLSKHKALENEISSHEPQLMSVVDIGRELVGRQHFGAARVQ ERLDEILDSWNHLLELSAYRRKRLEEAVDFHQFFADADDVDIWMLDTLRLVSS EDVGRDEANVQSLLKKHKDVTDELKNYAGAIEALHQQASQLGEQDRQAPQV VERLASIDHRYKELLEFAKLRKQRLLDALSLYKLLSESDGVGQWISEKDRMLQ TMVPVKDIEDVEIMKHRYDGFEKEMNANASRVAVVNQLARQLLHVEHPNSE QIVARQNQLNHEWAQLREKAEAKKEELNSAHGVQTYHIECRETMSWIEDKKR ILQSTDSLEMDLTGIMTLQRRLSGMERDLAAIQAKLDSLQQEAETIGAEHPEER ELIQERIDQIKLVWEQLTQMLKERDAKLEEAGDLHRFLRDLDHFQAWLTKTQT DVASEDTPASLAEAEQLLSQHQTIREEIDNYRDDYAKMMEYGERITAEPSTQD DPQYMFLRERLKALRDGWEELHQMWENRQQLLSQSLNLQMFNRDAKQAEV LLAQQEHVLSKDEAPTNLEQAENLIKRHEAFLTTMEANDDKINNVVQFASRLC DEGHYAADKVSKKADSINERRHANRNKALEQMEKLKDQLQLHQFLQDCEEL GEWVQEKTVTALDDTYRSAKTVHSKWTRHQAFEAEIASNKDRLFRIQEATAE LMKEKPELAELVEPKVQELGNQFDKLEETTREKGERLFDANREVLLHQTCDDI DTWMDELEKQIEGPDTGSDLASVNILMQKQQMIETQMAVKAKQVSDLETQAE	31

			KLQKTAPEKMGDIVAKKMQVEKRFAQLKQPLLERQRQLEKKKEAFQFRRDVEDEKLWIAEKMPQATSTDYGNSLFNVHMLKKKNQSLRTEVENHEPRINLVCSNGQKLIDEGHEDAEEFAGRIKELQEKWQALKDAMENRRKHLLQSEKAQQLLWERFKEFARDTEAIGSERVSAVNGIADQLIGAGHSDSATIAEWKDGLNEAWQDLLELIETRKQMLEASRELHKFFHDCKDVLSRILEKQTXGRAGPRCRTGFCPAAKAPHVHAGPADTWWPGKVEAIQASSAKLQTAYAGERAREITGREAEVVGAWNALQAHCEDRRQKLGDTGDLFRFFCMVRTLILWMDDVVRQMNTSEKPRDVSGVELLMNNHQSLKAEIDAREDNFTSCISLGKELLARNHYASSEIKEKLLSLTNQHKSLLHRWEERWLNLQLILEVYQFARDAAVAEAWLIAQEPYLMSQELGHTIDEVENLIKKHEAFEKSAAAQEERFSALERLTTLETRDDVDACTVLQLPPGSSFPEMTSVSSISDLDECVTAEVCLGLPRVDGEMSPEAEQVQVNGEDTEAREATPRPRPTKEPVAVASPPATSAGPGATASLDRKKDRSRSKSPFRSLRSWRRSPKAGTSGAHSDDEGTLERAAAERPSPGAEEDHMEGNLVRKHEWENTTKKASNRSWDKVYAVLRGNLLMFYKDQKTYKTSPDSFFKGESPVDLRGGACQVASDYTKRKHIFRVKLSNGAEYLFQARDDDDMNTWVSRLSQQCERDASAGPSRSQTLPASGDRKDEPKRRSFFTLKKKWPVTILYFELHAIFDRHSTLLTLLVA	
11	LMI_GLEAN_10140538	Titin [Harpegnathos saltator]EFN83273	PQDREEIITLVDKPKERKARPDISGKEVAEMTEIVPEFGMGKLEEEKASPAQAKKAHLPFESIIKTEVSVRECEEPLKGDRPSVEDNAVEEALPLMEKPEEEKADEKPEEVEDGMKETTKVKRLKRIVKKKVTPTDEEKPLGEDKKEESIPLHEKPEEEEVYETPEGVLLVAKAESTETAETTKEKKVKRIVRKKKITADEDKKPSVEEEETVPLEEKPIDEEKPELAEGEKEEVPEKTKLKRIVRKKKIMPDEKPQPKDTKKKVKVRKVKPGQEEKPEEEVHEEKKEEIQEIEKEQKEVHEDDKEIDTTDEVEVKEDKVKRVIRKKKTTPTEKEKPSEESKPEEIVTIQEKPEGQKVDEIPETLKEEDKVEEELVEQVGAAKEKKVKRIVKKKKVVTEDSEKPAEEGTTEDTLSLPEKPEEEKAKPNLDIEEKPDTVPTPKDDVPETVEEKEHKKVKRIVKKKKVAAPEEGEKPIVEEVEDEEQDIAPDKPTDGKPIKKKKLPALQLMKVERKEIKATKIKVIDVKEDIPLFAQIKLK	28

			KVTPVKKREIKSVTIPKVLLKSNIKFIDYPPAEQKPIITVLEVHFDKGILSRNYQE<br>ALKVQKRKVKKVRIKDKEATELEEPEKFPTEDTEKPERVEKDDQPEWQSLPKK<br>PAPEEPESKKLDIGKGKVPEQESPIPEEVKLKKIPEKPKPDEEEQKIKPPKKDKD<br>EQKVKKPKESEKVPLAPFEGRDIEKHDTDLEKYERESPEKSDEETPKPEAPKKP<br>KVKKSKPEPETVQIPLEKGIPKKPQPEDDKEIKLRKKMGEPTPDEPESITLKPFR<br>KEKPEDKPEQPEKEIGKPKGEDRPTELPEEERDQLPDGKGKKKIVKKVRKQGV<br>PQEEGEKPVPDVPEGETPEVISPKEEQPGEVQVPESVSEPLKAVDEQPKVSEVE<br>KPDEVGADEKPQPKDTKKKVKVRKGKPVEEEKPEEVVQEEKEEGKQKMVEE<br>PKEVPEDDKEAATTDEVEVKEKKVKRVIRKKKASPTDDEKISDELKEEETLPLL<br>EKPEDIPETVKEAEEVDQEEVTEEKKVKRIVKKKKIVPEPLQEEAAKDNLPVLK<br>GDEKHSPEDSHVHYANVMSSDEGQVTDMVTPSHETDRTVPEQEEIKLKKVPE<br>KPLEEETTEVKKPKKDDTDEEDKKSVPEEKDITVTPFKPSDIEGADLELERYVK<br>EEPEKKERKSPEKQKVKKPKKDIPKPEPETVKIPLEKQPPKKPLPEEEKDIKLKK<br>KTGEPEPEKPDSITLKPFVKEKPDEESDVPRKDSLKLDDKQKPSEEEEILPSEVK<br>KPESPERTIKKTKIKKPKPSKPVEDTEKDERSPQQEEPVLDEKPKQIEEMPEEELI<br>TKRKPKIEDTEEEEVKKEKPDKPEVEEIKEVTIKTKKPRKSSVEKVTEEVAQEV<br>VIKKKKPTEEKVEEVTEEVTIQKKEPEKPKEEASEEVKIKKKKPRKPSVEEVSEE<br>VTIKKVVPVEPKEEEVTEEVTIKKKEPEKPKEEVSEEVKTKKKNPRKPSVEEVS<br>EEVTIKRVVPVEPKEEEVTEEVTVKKEPEKPKEEVSEEVKIKKKKPRKPSVEEV<br>SEEVTIKKVVPVEPKEEEVTEEVTIKKKEPEKPKEEVSEEVKIKKKKPRKPSVEE<br>VSEEVTIKRVVPVEPKEEEVTEEVTIKKKEPEKPKEEVSEEVTIKKKKPRKPSVE<br>EVSEEVTIKKIVPVEPKEEEEVTEEVVIKKRKPKEAKEEEVTEEVTIKKKEPEKP<br>KEEVSEEVKIKKKKPRKPSVEEVSEEVTIKRVVEKKSVEPQEEDVTDEITEFTV<br>KKKAPQRKLSIEELDEEEITIKPLRPRQPSLPEVTEVTDVETVTIQPRITKTKEEIE<br>QEFKIQLHTYEEEDISMTSKVRLKKQRRPTYAEEAGEQTIRIVKEVEDQKSPEII<br>EILESPEAEAVSFDLKRKPSYTVQEAEEEETVVITKPQREREEIEESFQISLRKKD	

			DRKMSYSIENVEEEFSIDKIFVTEDFTLSIVKEKEVFQEGVEEILVTELQEGDVL<br>YSVSAYEAENEDAMNLVEGEKVYIIDMNNQDWWFVKKHLTEEKGWVPAQLL<br>MDEIRYTTFVQKVIQQKIDQLPVFEKPTDKATAPRFVEKLQPKHAPDGTTVEFQ<br>CQISGSPRPQVTWFKQTAILLPSHDFQMYYNEDNVATLVIREVFPEDAGTYTC<br>VAKNTAGFASSSTDLIVEGPLSDHGSDVAVISRKSVSRESSLADILEGIPPMFTQ<br>KPVPLSVDEGSDVKLDCQLVAVPEPVITWYFNKKEIDKTENISVTVDSDVHMY<br>FSKVSIKKVKKSQEGVYEVIARNSEGETSMPIILKVKTGKKEPPTILEPLKPTVV<br>HTGDPVVLRAVITGNPTPKVTWLKDGLPADDLPVSHDGDLHSIFLKDVGEGDS<br>GLYTVRAENPVGSVESTASLVVEAPDDNMEPPLFTERFEEVKVPEKGTLRLVA<br>KVTGKPVPEITWLRNNRPLKPSSNLTLSFDGTTSTLEIPNVDSEVDAGNYKCIA<br>KNPAXXXWSQVSVDVPEVTFVRKLQEITEVEEREALVLECETSHTVSTHWFHE<br>GHELSGMDHRVVVQEGRIHRLQIKNCTSRDAGLYQCRVRDQVTEGHVTVHD<br>KKPEFVRKLQDFEVKERESAILEVEITSETAEVTWHKDGEQLTDTSDKFVFEKQ<br>GTVRKLLIRGVSVHDEGEYTCALGEQECTAEVSVVELPPEITTKMQDVTVAKG<br>EKATFEIELTKGDALVRWFKDGKELQFSEHVQLSIDGKRQRLKVYNSELKDAG<br>VYSCEVGEQTSSARLTVEEPAVQFLKKLPDVTLIPLNMDATFTVELSRPDVEVK<br>WLMKGKEIKASERYQITSEGSVKKLIVKNVTIEDQTDYSCVALNVKSTSKLKV<br>EIIETPPKINIESLKKEYRVKKGDDVTIEVKFTAAPPPQDEWFINGKLVKKSPRIT<br>QTLAENSATLTIKQAEETDAASFTLKLSNTRGEASAEMTLVLMEPPSEPGTPEV<br>VEVTEQSITLHWKPPEFDGHSPITNYILEYHDKTDFTWIQVKEVVTETTHKVSQ<br>LQTDVEYMFRVSAENDVGRGPPSHNTHYIRVAKPPLEGVVCGLHQSISLSCVIG<br>GIPEPTVKWMKDGKVFKSKNLTYENRIAKCTISETTETSSGIYTCQATNTAGFA<br>ETSCELKIQEPPKLEFDESLTTQKLRITNQWKIEVKYSGFPKPEVVWTKNGKSL<br>ASTKHCSMYYDEYSSTIAIYSLAKVDTGSYTFTARSEAGTASVDFSLKVIDKPS<br>KPEGPIIVKDIRKDSVVLEWKPPADDGGLELSKYSVEKCDVEKMVWMKVADV<br>EKDVSSYCVQRLREDSEYMFRIIAENPVGTSEPLESDPVTIRSKLDKPSAPRGPL	

			DISGMTETSFTAAWQPPESDGGAPVLEYVIERREVGKKAWQKVGSTAKDVTHI EVSGLKTNTSHHVRITARNEVGVSAPYAPDDVITVGMRITLVLMTVSTLPKMQ VVVMPSKTTGAPSAPTNLQITDITSKSVTLQWGPPASTGGTELTGYIIEKRELSS KKWSKAVTLEPTVFQYCIENLKDRQEYFFRVYAENAIGLSPPAGTDLVYLKKH ATVPSPPTAPLEIRPIGPNAVVIAWGIPETDGGAPLQGYTIAIRDVRKTMWMEV GRVKADVHKLTIKDLQENHEYLIRIFAKNEVGPSEPLESEEPFKMLRPTEVDQL ELDKLETTAPTLSFSTETTTSWMKEAGMDADIRSYARGSLLRRDEYFFRIWYY ARQLFK	
12	LMI_GLEAN_101347 74	elongation factor 2 [Schistocerca gregaria] AEV89753	MLIYVTIQTLPMPPPAVNFTVDEIRVMMDKKRNIRNMSVIAHVDHGKSTLTDS LVSKAGIIASAKAGETRFTDTRKDEQERCITIKSTAISMFFELEDKDLTFITNPDQ REKGEKGFLINLIDSPGHVDFSSEVTAALRVTDGALVVVDCVSGVCVQTETVL RQAIAERIKPVLFMNKMDRALLELQLEAEELYQTFQRIVENVNVIIATYSDDSG PMGEVRVDPSKGSVGFGSGLHGWAFTLKQFSEMYAEKFKIDVVKLMNRLWG ENFFNPKTKKWSKQKEVDNKRSFCMYVLDPIYKVFDSIMNYKKEEAASLLQK LNIELKPEDREKDGKALLKVVMRTWLPAGEALLQMIAIHLPSPVVAQKYRME MLYEGPHDDEAAIGVKNCDPNAPLMMYISKMVPTSDKGRFYAFGRVFSGKVA TGMKARIMGPNYIPGKKEDLYEKAIQRTILMMGRYVEAIEDVPSGNICGLVGV DQFLVKTGTITTFKDAHNMRVMKFSVSPVVRVAVEPKNPADLPKLVEGLKRL AKSDPMVQCIIEESGEHIIAGAGELHLEICLKDLEEDHACIPIKKSDPVVSYRETV SEESDQMCLSKSPNKHNRLFMKAVPMPDGLAEDIDSGEVNPRDEFKARARYL SEKYEYDVTEARKIWAFGPDGSGPNLLLDCTKGVQYLNEIKDSVVAGFQWAT KEGVLAEENMRGVRFNIYDVTLHTDAIHRGGSQIIPTTRRCLYACVLTAQPRL MEPVYLCEIQCPEVAVGGIYGVLNRRRGHVFEEMQVAGTPMFVVKAYLPVNE SFGFTADLRSNTGGQAFPQCVFDHWQILPGDPMESGTKPFGVVQDTRKRKGL KEGLPDLTQYLDKL	26
13	LMI_GLEAN_101285	Pyruvate kinase [Zootermopsis	MAEQTGLPKMQMQAADAVTHLDHMCALDIDSKASYVRLSGIICTIGPASRDP	26

	85	nevadensis] KDR19430	ATLEKMMETGMNVARLNFSHGSHEYHAETIKNIREAAANYSKKLGMEYPLAI ALDTKGPEIRTGLLEGGGSAEVELVKGQTLKLTTDKAYMERGTAETVYVDYD NILKVVKPGNRIFIDDGLISVICTSVAPPHLVCTVENGGLLGSRKGVNLPGIPVD LPAVSEKDKKDLLFGVEQQVDMVFASFIRNAAALQEIRSVMGEGGKKILVISKI ENHQGMVNLDEIIEASDGIMVARGDLGIEIPPEKVFLAQKAMISRCNKVGKPVI CATQMLESMVKKPRPTRAETSDVANAILDGADCVMLSGETAKGDYPLECVLT MANICKEAEAAIWQRQIFSDLSSKAVPPMDVAQSVAIATVEASMKCLASAIIVI TTSGRSAHLISKYRPRCPIIAVTRYAQVARQCHLYRAILPLHYTAPPLQDWLKD VDVRVQFGLQFGKSRGFIRDGDPVIVVTGWKQGSGFTNTMRIVYVSPVM	
14	LMI_gi_37993866	heat shock protein 70 [Locusta migratoria] AAP57537	KAPAVGIDLGTTYSCVGVFQHGKVEIIANDQGNRTTPSYVAFTDTERLIGDAA KNQVAMNPSNTIFDAKRLIGRRFDDQAVQSDMKHWPFKVINDSGKPKIQVQY KGETKTFFPEEVSSMVLTKMKETAEAYLGKNVSNAVITVPAYFNDSQRQATK DAGAIAGLNVLRIINEPTAAAIAYGLDKKVSGHGERNVLIFDLGGGTFDVSILTI EDGIFEVKATAGDTHLGGEDFDNRMVNHFVQEFKRKYKKDLTTNKRALRRLR TACERAKRTLSSSTQASIEIDSLYEGIDFYTSITRARFEELNADLFRSTMEPVEKA LRDAKMDKAQIHDIVLVGGSTRIPKVQKLLQDFFNGKELNKSINPDEAVAYGA AVQAAILAGDKSEEVQDLLLLDVTPLSLGIETAGGVMTTLIKRNTTIPTKQTQT FTTYSDNQPGVLIQVYEGERAMTKDNNLLGKFELTGIPPAPRGVPQIEVTFDID ANGILNVTAVEKSTGKENKITITNDKGRLSKEEIERMVNEAERYRAEDEKQKA TIAAKNGLESYCFNMKSTVEDEKLKDKISDSDKQTILDKCNEVIRWLDANQLA EKEEFEEKQKELEQICNPIITKLYQGAGGAPGGMPGGFPGGFPGAGGAAAGGA GAGGAGPTIEEVD	25
15	LMI_GLEAN_101609 12	glycogen phosphorylase-like [Apis florea] XP_003690485	MALPQSDIEKRKQISVRGIAQVEDVSTFKKSFNRHLHYTLVKDRNVSTPRDYY FALAHCVRDNLVSRWIRTQQYYYEKDPKRIYYLSLEYYMGRSLQNTMINLGIQ NACDEAMYQLGLDIEELEELEEDAGLGNGGLGRLAACFLDSMATLGLAAYGY GIRYEYGIFAQKIKNGEQVEEPDDWLRFGNPWEKARPEYMLPVNFYGRVEDTP	25

			QGKKWVDTQIVFAMPYDSPIPGYGNNVVNTLRLWSAKSPVEFNLKFFNDGDY<br>IQAVLDRNLAENISRVLYPNDNFFEGKELRLKQEYFMCAATLQDIIRRYKASKF<br>GVRDAVRTSFDSFPEKVAIQLNDTHPSMAIPELQRILVDIEGLSWEKAWEIVVR<br>TCAYTNHTVLPEALERWPVHMIERILPRHMQIIYHINHLHLEEVGKKYPGDLD<br>KLRRMSLIEEHGEKRVNMAHLCIVGSHAVNGVAAIHSEILKKDVFRDFYEMTP<br>HKFQNKTNGITPRRWLLLCNPGLADLIAEKIGEDWIVHLEQLQKLKKFAKDPT<br>FQRAIVKVKQENKLKLAQLLEKEYNVKINPASMFDIQVKRIHEYKRQLLNCLH<br>IITMYNRIKRNPNVNISPRTVMIGGKAAPGYYMAKKIIKLICSVGNIVNNDPVV<br>GDKLKVIFLENYRVTLAERIMPAADLSEQISTAGTEASGTGNMKFMLNGALTI<br>GTLDGANVEMAEEMGRDNIFIFGMTVDEVEELKKRGYNAYDYYNRNPEIRQC<br>IDQIQNGFFSPGNPDEFRDVADMLMKYDRFYLLADYEAYIKCQEKVGEVYQN<br>QSKWTEMAINNIASSGKFSSDRTISEYAREIWGVEPSWEKLPAPHEPREEDVAK<br>EAKK	
16	LMI_GLEAN_101571<br>78	staphylococcal nuclease<br>domain-containing protein 1<br>[Tribolium castaneum] |XP_974879	MTTQQPKVNRGIVKQVLSGDTIIIRGQPKGGPPKEKTLILSNIIAPKLGRRATAN<br>SEETKDEPYAWDAREFLRKKLIGEEVLFTLDKAQNTSREYGCIYLGKDLATAE<br>NVTESLVAEGLADVREAGRSASPEHAKLIELRDHAKTAEKGMWAKTSTQKPA<br>RDIKWVIENPRNFVDKFQQKPIKAIIEHVRDGSTVRAFLLPDFYHITLMISGIRCP<br>GFKLDADGKPDPTAVVPYADEAKFVVESMLLQRDVEIVLESVNNNNFVGSILH<br>PKGNIAEVLLKQGFARCVDWSMAFMTTGADKLRAAEKVAKEKKLRLWKDY<br>QATGPQVTGKEKEFSGIVVEVMNGDALNVRLPDGRVKKVFLASIRPPRQADA<br>KPDEAPQQRNKVFRPLYDIPWMFEAREFLRKKLIGKRVNVVVDYIQTARDNFP<br>EKICCTVTIGGVNVAEAMVSKGLATVVRYRQDDDQRSSHYDELLAAEMKAA<br>KSGNGLHSKKNIPNHRVADVSGDLNKAKQFLPFLQRAQRTEAIVEFVASGSRL<br>RLYIPKETCLVTFLLGGINCPRGSRPGPGNTGIVEGEPYGEEALAFTKEKCLQRE<br>VEIHVESMDKAGNFIGWLWIEGVNLSVALVETGLASVHPTAERSEHYRALKTA<br>EDTAKAKKLKIWKDYVEENEEEKKIEEERVVERKIDYQKVLIIEATPDLHFYAQ	23

			MVDQGLNLEQLMQKMRQEFTTNPPLPGAHTPKKGELCAARFTLDREWYRAK<br>VEKISGSNVSVFYVDYGNRETINVADCAKLPAEYSSEKYFAQEYALACVQLPK<br>NDNDYREEAVKAFISDVENRPVLLNVEYRIGNLPYVTLVEVNDQEEDIVKNLI<br>KDGLLLVDSRREKRLQKLVEEYKAAEEVAKKNHLNVWEYGDIREDDAKEFG<br>LGR	
17	LMI_GLEAN_101530<br>94	coracle, partial [Blattella germanica]<br>emb|CCI09964	MPEETKAAEKGEQQPAVKQSSGGGKTVLARVTLLDGSLLELNVERRAKGQEV<br>LDKVCEHLNLLEKDYFALTYEDRHDPRNWLEMDKRVTKFIKNEPWKFNFEVK<br>FYPPDPAQLQEDITRYQLCLQIRNDILEGKLPCSFVTHALLGSYLVQSELGDYD<br>PDEHGRNYLKDFRFAPNQTAELEEKVMDLHRTHKGQTPAEAELHYLENAKKL<br>AMYGVDLHPAKDSEGVDIMLGVCASGLLVYRDRLRINRFAWPKILKISYKRH<br>NFYIKIRPGEFEQYESTIGFKLANHRAAKKLWKVSVEHHTFFRLMTPEPTQKSG<br>LFPRFGSKFRYSGRTHYETKKALIERPAPRFERSLSGRALTSRSMDALSGPGRL<br>QAENASDANKRHTMSHPPDHYPDMEPARPVKELKEKEEKIKDKKPVGGVAVL<br>PPGALGFGKKQKKKDKDGSDKEKGAAEAEDEEKENRQDNDSLNTSDEKSPTR<br>KREKLKIKTPGFLFTKGSPPKDKEKEEKEKEDGTPEKEKKEKERKEKEKKEKD<br>KKEKEKKDKKGKEEKVAVALTDETAKSGVSGGPPKGSPQLPGYTREYDYVEE<br>EPQTDDQRRFEPQGGFSYEKGRLEATPERSPPSSSGRRATGLAFNYAPGETTPA<br>NKGLRTPGIDYVASAGLKEQAKGVGTAPVVVPQTTQSSKFIIIAGGKPSVVAGC<br>EPPIVKSEDGKLVVDSAWPSPLAKAQVVAGPDEKPAPVRDVYPGLILKDGKLH<br>LPKDIDVIEGYEVLENKDGSGVTVNQCRYLGTYTGRDGRPVQGKKKPASVAG<br>AELLVSRDGKPLLAADSKGKGGGFVVGRSGKIDIPPEEPFSATLSGAMAVKGH<br>DGNPLLVWGQLPNTTTGPDGTLYITGRHSGDLDGAPVIMGNDGKPLPITAVCG<br>SSKHNSLIPGAVVSVSKGKTDVHRTGFLGEIEDDGDRVRGISVTVDRTGQPIEA<br>PPVAVVILGPTGKAALLTGDHQSAIAAGRDGKGTVTCSGEFEGAPTLINKDGK<br>LVLVDGWPAMLKADGGYQAEFSDVIEGARVIRGEDGQPKAVKSKYIGTVVTG<br>DKGRIEKVKLVLDPEGQPLAVTESDLGTVISADGKPIERLQVVPSPKTTPEKKT	23

			SSPIPSLPKITLPKLSTGSPRLPTAEGSKIQDTEALLAAERSHAGILGIAPGEVVAL<br>PETQVLLLTSKDGKPLLATGTIPPPVADQDGKKTIPGKFSGSLDGATVITDRQG<br>QPVATSGGRADVVTGPDGKPAVSAQFTHVVEGAKLLSGKSPQKPDLSYTKYL<br>VNLTGSGGKPIASGEKLEKVEAVTVILGADGKPLEVTESAPGVLIAGDKKPVIN<br>TNSGFSGVLAAGRTSPTDARVLPAEHVLCLIAKDGSPLVVTGDLPTPVTNKDG<br>RKALPGKLAGKLDGATILVDKNGKPVTTVGQPVEVTTGNDGKPIVSAPFSHVV<br>EGAKIRPGKGGKLDATNTKYLTSVIGPGGRTLKPGEKLENIETVRLVVTADGK<br>PVEVTEPSPGVLLSSDGKPIINSVTPFGGERTANLIPKLQITELPVSEALLVISKEG<br>VPVIVTGDLPAPVTAKDGKKTLPGKFSGKLDGAVVTAKPDGQTMTLKGLEIEP<br>VISPNGKPAVGAQYTHVVEGAKIVPKKSGKPEVTLTKYLVTLIGKDGKPITLGS<br>KNVDVAEATVVLGTNGKPLEVSETPKGELCSSDGKPVMKLTSEADAEGIVRA<br>VVSPGHPVSFLAADGKQVSTVPLRYGGLIGTTDPAPYVDGVTVVVDKEGHPVI<br>LTGQYPAPVISKDGKKVLPPGKFPGSLDGAIVLEDEHSKPLIPRGLYTEVVAGP<br>DGKPQKVVHYAYIIDGARIIIGKNGEPVVSREKFLGTLVGKDGELWSPDKSPSD<br>IKGAVVVVEKDGEPVTVVEKLPGIIADKDGQPIISVEKHLGIMPHSRLVISPGSP<br>ATGTGSQPFMLSVPFTQTVAVASGVVAVVGNDGKVSVVAGQFPAPVTKSGK<br>MVLPEDFPGKLAGAPILVDRDGKPVVMKGLQAQQLLSDGKPVLAADHSHIVE<br>GTTVTLNKDGKPLSSPAKFLVTLVGRDGKPLPSEKTLATEVNGLTIVLGPDGQP<br>LPVTEIVAGTVISKDGKPVITTSHSSLVPSVDHRIPSVKPVTSVTTSDSTLTTLVT<br>PQPIHIIEGVIAVVRKDGKPVILTGQYPAPITDKNGKKVLPPKFPGNLEGAVVLV<br>DKDSKPTILRGTEIAQTSGSDGKPLAVADYTQVIEAARVSLGKDGKPIVTADKF<br>VGSLMTKDGKPLPPGRSPLEAEGVTVVLGKDGQPMAVEETPLGIVSAQDRQPL<br>LSVQKFLESVPGSSVSYSSPSSQTSSGTVQTTRVVMGSALSGGPPVVTTHTINIV<br>EGVMAAVKKDGKPVILVGQFPAPVSGKGGKKILPTNFPGGLEGAAVLVGKDS<br>QPILLKGQYAATTSGNDGKPVTVADYTQIVEGVRVTLDKENKPLVSNTKFLGS<br>FVGSDGKPLPPGRPLVDSQGATVVLGQDGQPLPVRVSSFGSITSESGEPILSVQK	

			FLESVPGATVAFEQAGPTTSTTVETTSVILGPDGRPVQLGSPHTTSRTIGIVDGI<br>MAVVRKDGKPVILTGRFPAPVPGKDGKKVLPPKFPANLEGATVLTDKLGKPLT<br>LKGLYAAKTSDNDGKPLAVADYSEVIEGVKLVMDEENKVTASPDKFLATLVT<br>RDGKTWSPGRYPGDMESVTVVLGQDGQPLSITDIGGTILGKDGVPILSVQKFA<br>ETVPGSTVSFMQKGGAPSAGTVQTSSVVLGPDGKPVASVTGKDSIESFTSHTIS<br>TVEGVMAVVKKDGKPAILTGQYPAPVTTKDGRRVLPPKFPSSLEGATVLTNNE<br>GKPLILKGVYAAQTEDPSGKPLYVADYSQIVDGAKVSLGKDGKVTVVNEKFL<br>AKLYGQDGQPILPSAVSPGSVHSVSVILGKDGQPLAVTEKPSGDVVGSDGTSVI<br>TMQRFTESVTTSTVKTASSVGGVVRTETIAIMEGVTALLARNGQPVIITGQYPA<br>PVIGADGRTIIPGQFSGLLDGATVLTGTDGRPLVLKGVPAEAIKSRNGEPALAV<br>DYTHILEGAKLSVGRDGKPSVVPKHYLAKLLGPGGEKVPSGSLPSQVTGVVVV<br>LGEDGEPVAVSGTDRGRIRVEGNDLKIFRRGSDGELSDEEDSGDELAAYAEDTI<br>LGPKVVKTTTKRTVVKDSGGLRRNVEEKVEDLGAGTVSLTTHEDEAEVGSDD<br>GRSPHVTATAVTTRTATTHEDKETNAKTSQVEEKTVAHTTTTSGARQEQRTVT<br>QEMRATSTVVTTDQQFSRRSSTSSDDSGTPVDLLDESEDGLFYANGGARLQPI<br>VPTESKVYSQPGSPSTTSVTSTTKVPVVATETRKVTVEEGPYSASGEIVSSQTIS<br>SKTRTVETITYKTEKDGVVETRVEQKITIQSDGDPIDHDRALAEAIQEATAMNP<br>DMTVEKIEIQQQQQQQGAPVQGEEIKSLQFADDIVILSETAKDLEEQLNRIDIVL<br>KGAYKMNINKSKTSVVECTLRNTGNGEGIKLGNETINIIMESPGWN	
18	LMI_gi_93278396	heat shock protein 90 [Locusta migratoria] AAS45246|	MQDAAEVETFAFQAEIAQLMSLIINTFYSNKEIFLRELISNSSDALDKIRYESLTD<br>PSKLDSGKDLWIKIVPNKSERTLTIIDTGIGMTKADLVNNLGTIAKSGTKAFME<br>ALQAGADISMIGQFGVGFYSAYLVADKVTVASKHNDDEQYLWESSAGGSFTI<br>RPDPGEPLGRGTKITLYVKEDQTEFLEERKIKEIVKKHSQFIGYPIKLVVEKERD<br>KELSEEEEEEEKKEGGEEGDNESKPKIEDVGEDEEDESGDKKKKKKKTIKEKY<br>LEDEELNKTKPIWTRNPDDISQEEYGEFYKSLTNDWEEHLAVKHFSVEGQLEF<br>RALLFIPRRAPFDLFENKKRKNNIKLYVRRVFIMDNCEDLIPEYLNFIKGVVDSE	21

			DLPLNISREMLQQNKILKVIRKNLVKKCLELFEELTEDADTYKKFYEQFSKNLK<br>LGIHEDSTNRKKLSDLLRYATSASGDETCSLKDYVARMKENQKHIYYITGENK<br>DQVANSSFVERVKKRGFEVVYMTEPIDEYVVQQMKEYDGKQLVSVTKEGLEL<br>PEDEEEKKKREEDKAKFENLCKVMKDILDKKVEKVVVSNRLVESPCCIVTSQY<br>GWTANMERIMKAQALRDTSTMGYMAAKKHLEINPDHPVMETLRQKAEADK<br>NDKAVKDLVMLLFETALLSSGFTLEEPQVHASRIYRMIKLGLGIDEEEPQAAEE<br>EKVDAEMPPLEGDNEDASRMEEVD	
19	LMI_gi_99867354	arginine kinase [Locusta migratoria manilensis] ABF68036	MVDAAVLEKLEAGFKKLEASDSKSLLKKYLTREVFDQLKTKKTSFGSTLLDCI<br>QSGLENHDSGVGIYAPDAEAYTVFADLFDPIIEDYHGGFKKTDKHPPKNFGDV<br>DTLGNLDPNGEYVISTRVRCGRSMQGYPFNPCLTEAQYKEMEQKVSTTLSGLE<br>GELKGQFYPLTGMSKEVQQKLIDDHFLFKEGDRFLQAANACRYWPSGRGIYH<br>NDNKTFLVWCNEEDHLRIISMQPGGDLGQVYRRLVHAVNEIEKRIPFSHDDRL<br>GFLTFCPTNLGTTLRASVHIKLPKLAADRAKLEEVAGKFNLQVRGTRGEHTEA<br>EGGVYDISNKRRMGLTEYDAVKEMNDGILELIKIEGTL	20
20	LMI_GLEAN_10097368	ATP-citrate synthase [Zootermopsis nevadensis] KDR07798	MSAKAIREATGKHLINNHLQSGTAASFCRFASVDENTNWDELRSKHPWLLTE<br>KLVAKPDQLIKRRGKLGLITVNKDFEEVKEWITARLGVDQVIGRATGKLRTFII<br>EPFIPHKPEEEVYVCIYSHRYADTILFHHEGGVDIGDVDAKALKLDIPVNEEVTL<br>DDIKRTLLTNVTQEKKELIAQFIVSLYKLYVELYFTYLEINPLVVTEKSIYILDLA<br>AKLDATADYICRAKWGEIDYPPPFGRDAFPEEAYIADLDAKSGASLKLTILNKT<br>GRIWTMVAGGGASVIYSDTICDLGGASELANYGEYSGAPSEQQTYEYAKTILS<br>LMTQTKHPKGKILIIGGGIANFTNVASTFKGIVTALQEFQPKLVEYNVSIFVRRA<br>GPNYQEGLRIMRDVGKNLGIPLHVFGPETHMTAIVSMALGKKPIPSVTEVEFAT<br>ANFLLPGGQKDSPQQSRKGSLASSTGNSSQRETPVRSFSSSGGEDVQDGSQRGI<br>LFTKSTKAIVWGMQSRAVQSMLDFDFVCRRSEPSVVAMVYPFTGDHKQKFY<br>WGHKEILIPVYKKMEDAMTKHRSADVLVNFASLRSAYESTLEAMNFPQIRTIAI<br>IAEGIPENLTRKLIVLAHKKGVSIIGPATVGGLKPGCFKIGNTGGMMDNILHSKL	20

			YRPGSVAYVSRSGGMSNELNNIISKSTNGVLEGVAIGGDRYPGTNFMDHILRY QADPEVKMIVLLGEVGGVEEYAVCKALKDKRITKPLVAWCIGTCAGMFTSEV QFGHAGSCANSDMETAVAKNKALSEAGAHVPTSFDSFGDVIAEVYESLVESG VIVPAPEVPPPTVPMDYSWARELGLIRKPASFMTSICDERGQELIYAGMPISSVL RQNLGIGGVISLLWFQRCLPAYVCKYFEMCLMITADHGPAVSGAHNTIVCARA GKDLVSSVVSGLLTIVLRQNLGIGGVISLLWFQRCLPAYVCKYFEMCLMITAD HGPAVSGAHNTIVCARAGKDLVSSVVSGLLTIGDRFGGALDGAARQFSEAYDT GLIPMEFVNTMRSKGQLIMGIGHRVKSINNPDMRVKIIKEFVMEHFPNKPLTQY ALEVEKITTSKKPNLILNVDGIIATSFVDMLRHCGSFTREEAQEYIEMGAINSLF VLGRSIGFIGKYTSLIAEVPFKKESSTVANYFLFTVSQYTSHFMDQKRLKQGLY RHPWDDISYILPEQYN	
21	LMI_GLEAN_100438 24	clathrin, partial [Locusta migratoria] AHC70342	MVRSGRIRSAPTELTSVGINQASVSFNTLTMESDKFICVREKVGDTAQVVIIDM ADPSNPIRRPISADSAIMNPASKVIALKGKAGVEAQKTLQIFNIEMKSKMKAHT MTEDVIFWKWISLNTLALVTETSVYHWSMEGDSVPQKMFDRHSSLNGCQIINY RTDPKQSWLLLIGISAQQSRVVGAMQLYSVERKCSQPIEGHAASFAQFKMEGN AEPSTLFCFAVRTLQGGKLHIIEVGHPPAGNQPFPKKAVDVFFPPEAQNDFPVA MQVSAKYDVIYLITKYGYIHLYDIETAVCIYMNRISGDTIFVTAPHESSGGIIGV NRKGQVLSVSVEEDNIIPYINSVLQNPDLALRMAVRNNLAGAEDLFVRKFNLL FQNGQYAEAAKVAANAPKGILRTPQTIQRFQQVPTPQGQTSPLLQYFGILLDQ GQLNKYESLELCRPVLQQGRKQLLEKWLKEDKLECSEELGDLVKQADPTLAL SVYLRANVPNKIATKYHEQLTTKALIDLFESFKSYEGLFYFLGSIVNFSQDQEV HFKYIQAACKTGQIKEVERICRESNCYNAERVKNFLKEAKLTDQLPLIIVCDRF DFVHDLVLYLYRNNLQKYIEIYVQKVNPSRLPVVVGGLLDVDCSEDIIKNLILV VRGQFSTDELVEEVEKRNRLKLLLPWLESRVHEGCVEPATHNALAKIYIDSNN NPERFLKENQFYDSRVVGKYCEKRDPHLACVAYERGQCDRELINVCNENSLF KSEARYLVRRRDPELWAEVLNENNPYKRPLIDQVVQTALSETQDPEDISVTVK	19

			AFMTADLPNELIELLEKIVLDNSVFSDHRNLQNLLILTAIKADRSRVMEYINRL<br>DNYDAPDIANIAINNQLYEEAFAIFKKFDVNTSAIQVLIDNVQNLDRAYEFAER<br>CNEPAVWSQLAKAQLQQGMVKEAIDSFIKADDPSAYIDVVETAHKTGSWEDL<br>VRYLQMARKKARESYIESELIYAYARTNRLADLEEFISGPNHADIQKIGDRCFD<br>DGMYEAAKLLYNNVSNFARLAITLVHLKEFQGAVDGARKANSTRTWKEVCF<br>ACVDSEEFRLAQMCGLHIVVHADELEDLINYYQARGYFEELINLLEAALGLER<br>AHMGMFTELAILYSKYKPAKMREHLELFWSRVNIPKVLRAAEQAHLWAELVF<br>LYDKYEEYDNAVIAMMNHPTEAWREGHFKDIITKVANIELYYKAIQFYLDYKP<br>LLLNDVLLVLAPRMDHTRSVNFFTKVNHLQLVKPYLRSVQSLNNKAINEALN<br>NLLIEEEDYQGLRTSIDAFDNFDNIALAQKLEKHELIEFRRIAAYLYKGNNRWK<br>QSVELCKKDRLFKDAMEYAAESKNAEVAEELLAWFLEKGNHDCFAACLFQC<br>YDLLHPDVILELAWRHNILDFAMPYLIQVVREYITKVNMYYS	
22	LMI_GLEAN_101640<br>84	Heat shock 70 kDa protein cognate 5 [Zootermopsis nevadensis] KDR08641	MLFVCKYVGRKALDSTYYVTETASKNQFSTLLSKVATPALGTNQNVTQFRHK<br>SEGVKGAVVGIDLGTTNSCVAVMEGKQAKVIENSEGARTTPSVVAFTKDGER<br>LVGMPAKRQAVTNSANTFYATKRLIGRRYNDAEVQKDMKTVSYKIVKASNG<br>DAWVEGSDKKMYSPSQIGAFVLMKMKETAESYLNTSVKNAVITVPAYFNDSQ<br>RQATKDAGQIAGLNVLRVINEPTAAALAYGMDKTEDKVVAVYDLGGGTFDIS<br>ILEIQKGVFEVKSTNGDTFLGGEDFDNVLVNHLVAEFKKDQGIDITKDPMAMQ<br>RLKEAAEKAKIELSSSLQTDINLPYLTMDSSGPKHMNLKLTRSKFESLVGDLIK<br>RTIQPCQKAVQDAEVNKRDIGEVLLVGGMTRMPKVQSTVQEIFGRQPSRSVNP<br>DEAVAVGAAIQGGVLAGDVTDVLLLDVTPLSLGIETLGGVFTRLISRNTTIPTK<br>KSQASINLTVFSTAADGQTQVEIKVHQGEREMAADNKLLGQFTLVGIPPAPRG<br>VPQIEVTFDIDANGIVHVSARDKGTGKEQQIVIQSSGGLSNDEIENMIRNAEAFA<br>AQDKKKKDRVEAVNQAESITHDIESKMEEFKDQLPKEECDKMKEEIQKVREL<br>VAKKDETDPEEIRKAAGSLQQASLKLFEMAYKKKLDLSLLQIIDVECADNSSN<br>KSSFIILNFKEVLQINKKASGGWEVPNGFGDEAAVKVKGVLLRCMLHHRIVHI	18

			ALGHSLTVAIHPDREPMAAERDSSSGSSGSSTEKPEEEEEKKAKKE	
23	LMI_gi_241997152	ER protein gp78 [Locusta migratoria] ACS75353	MRVLSLSAFLAVIIGISLAKEEKNKDVGTVIGIDLGTTYSCVGVYKNGRVEIIANDQGNRITPSYVAFTPDGERLIGDAAKNQLTTNPENTVFDAKRLIGREWTDPTVQHDIKFFPFKVKEKNSKPHIQVATSQGEKMFAPEEISAMVLGKMKETAEAYLGKKVTHAVVTVPAYFNDAQRQATKDAGTISGLVVMRIINEPTAAAIAYGLDKREGEKNVLVFDLGGGTFDVSLLTIDNGVFEVVSTNGDTHLGGEDFDQRVMDHFIKLYKKKKGKDIRKDNRAVQKLRREVEKAKRALSSGHQVRIEIESFFEGDDFSETLTRAKFEELNMDLFRSTMKPVQKVLEDADMNKNDVDEIVLVGGSTRIPKVQQLVKEFFGGKEPSRGINPDEAVAYGAAVQAGVLSGEQDTDAIVLLDVNPLTMGIETVGGVMTKLIPRNTVIPTKKSQIFSTASDNQHTVTIQVYEGERPMTKDNHLLGKFDLTGIPPAPRGVPQIEVTFEIDANGILQVSAEDKGTGNREKIVITNDQNRLTPDDIERMIKDAEKFADDDKKLKERVEARNELESYAYSLKNQLSDKEKLGAKVSGSDKTTMEEAIEEKIKWLEANQDASTEEFKKQKKELEDVVQPIIAKLYQSSGGPPPSGSSEDDDLKDEL	18
24	LMI_gi_256368118	hexamerin-like protein 2 [Locusta migratoria]ACU78069	MRTATVVVLSLLAALAAAAVVPHSEAGKELLEKQDKLLRLLYHVQQTTLVKEEQEIAKTYKPIEHVDNYQYKDKVELFWKYYVDVGFLPKGEVFSVYYQKHFYQARALFNLFFFAKDFETFYKTAVWAREHLNEALFVYSYTVAVLHREDTKDVTLPAPYEVYPQLFVNAEVIQQAYDAYLRGEVGTKEAPYVFYSNYSGYPVASNPEELVSYFTEDIGLNSYFAYLSYKYPYWLNPKNYSLPEYQYRGESFFFVLQQLLARYYLERLSNHLPDVKAVDYNHPVLVGYYPELRLQNGREAPARPEGIFARNVDILYVEEIRNYERRIRDGIDYGYLAGYNYEKYNVREKDYTNVLGNILEGNEDSINKEYYGAFYRNLISLFGHIVDPVHRYGVPASVLEQPETQLRDPLFYRIGKRVLSIFYHYKNLLRPYTHEDLYLPGVTVEDITFDKLVTFFDTFDFEINNALTLSKPEEGAGFSYVARQYRLNHKPFFYHLKVKSEKEVDSVVRVFIGPKYDALGREYSLEERKQYYVLLDTFNYKLVAGENDIKRSSNDFPFYAKEAPSWYDLYKATSSAVKGEDKFFLDKFRSHFGFPQRLALPRGTRSGLPLSVFTIVTQASPDAKNPILEHGDLHAAG	18

			FPFDRRVVEFEFDVPNAHFDETFVVHRRVEDLNATA	
25	LMI_GLEAN_101095 13	tropomyosin-1 [Nasonia vitripennis] XP_001599003	MDAIKKKMQAMKLEKDNAMDKADTCEQQAKDANARADKVNEEVQDLKKK LVQVEEDLVNTKNKLEQANKDLEEKEKALQNAESEMAALNRKLQLIEEDLER SEERLATASTKLAEASEAAEESKRMCKVLENRSLQDEERMDQLTNQLKEARL LAEDADGKSDEVSRKLAFVEDELEVAEDRVKSGDAKIMELEEELKVVGNSLK SLEVSEEKANQRVEEYKKQIKTLTVKLKEAEARAEFAEKTVKKLQKEVDRLE DELGINKDRYKSLADEMDSTFAELAGY	17
26	LMI_GLEAN_100658 78	60 kDa heat shock protein, mitochondrial [Zootermopsis nevadensis] KDR14060	MALIASQYSNYIPRYCDAKLTLEFAKRACPEQHGVLASVSGRALATRFAYFKR QLRVAISRDGRNVILEQSWGSPKITKDGVTVAKGVELKDKFQNIGAKLVQDVA NNTNEEAGDGTTTATVLARAIAKEGFEKISKGANPIEIRRGVMMAVEAIIDHLK TLSKPVTTPEEIAQVATISANGDQKVGELISEAMKKVGKEGVITVKDGKTLQD ELEVIEGMKFDRGYISPYFINTSKGAKVEFQDALLLLSEKKISSVQSIIPALELAN SQRKPLVIIAEDVDGEALSTLVVNRLKIGLQVAAVKAPGFGDNRKATLQDIAIA TGGIVFGDEGNPVKLEDLQPSDLGQVGEIVITKDDTLMLKGKGNKTDIDRRAE QLRDQIDSTTSEYEKEKLQERLARLASGVAVLKVGGSSEVEVNEKKDRVNDA LNATRAAVEEGIVPGGGTALLRCGPILAKLQPNNVDQATGIDIVKKALRMPCM QIAKNAGVDASVVVSKVEDATGDMGYDALNNEYVNLIERGIIDPTKVVRTAL TDAAGVASLLTTAEAVVTEIPKEETAPAGMGGMGGMGGMGGMGGMM	17
27	LMI_GLEAN_100019 37	myosin heavy chain, non-muscle-like isoform 2 [Bombus terrestris] XP_003394420	MADSDTKLDRSDPELKYLSVDRNMFNDPATQAEWTQKRLVWVPHETHGFVA AGIKGERGDEVEVEIQDTGKRILVAKDDIQKMNPPKFDKVEDMAELTCLNEAS VLHNLKDRYYSGLIYVSTYIYVLGSQSRCPALTQSVFEKLMYVVVSACCTVLF YNVVHDFLFSVSDREDQSILCTGESGAGKTENTKKVIQYLAYVAASKPKSSAP HTGELEQQLLQANPILEAFGNAKTVKNDNSSRFGKFIRINFDASGYIAGANIET YLLEKSRAIRQAKDERTFHIFYQLLMGASPEQRKEFILEDPKLYTFLSSGGHLPV PGVDDVAEFQATCKAMSIMGLTPEDFSAIFRIVSATLMFGNMRFKQERNSDQA TLPDNTVAQKVAHLLGLSVTDMTKAFLKPRIKVGRDFVTKAQTKEQVEFAVE	17

			AIAKACYERMFRWLVNRINRSLDRTKRQGASFIGILDMAGFEIFELNSFEQLCIN<br>YTNEKLQQLFNHTMFILEQEEYQREGIEWKFIDFGLDLQPTIDLIDKPMGIMAL<br>LDEECWFPKATDKSFVEKLVSAHSVHPKFMKTDFRGSADFAVIHYAGRVDYS<br>AHKWLMKNMDPLNENVVSLLQASQDSFVVQIWKDAEIVGMAQQALTDTQFG<br>ARTRKGMFRTVSQLYKEQLARLMVTLRNTNPNFVRCIIPNHEKRAGKIDAPLV<br>LDQLRCNGVLEGIRICRQGFPNRIPFQEFRQRYELLTPNVIPKGFMDGKKACEK<br>MIQALELDPNLFRIGQSKIFFRAGVLAHLEEERDYKITDLIVNFQAFCRGYLARR<br>NFQKRTQQLNAIRIIQRNCAAYLKLRNWQWWRLYTKVKPLLEVTKQEEKLSQ<br>KEDELRQVRDKLDQQLRAAQEYERKFQQAVEEKIALSEQLQAETELCAEAEE<br>MRARLLARKQELEELLQDMEARMEEEEERVTQLNNEKKKLQLNIQDLEEQLE<br>EEEAARQKLQLEKVTCDAKIKKLEEDLALSDDTNQKLLKEKKVLEERAADLS<br>QTLAEEEEKAKHLSKLKAKHEATIAELEERLLKDHQQRQEMDRSKRKVETEV<br>SDLREQLSEKRIQLEEMQLQLGKREEELTQALMKIDEEAASKAQSQKALRELE<br>SQLAELQEDLEAEKAARSKAEKQKRDLNEELEALKNELLDSLDTTAAQQELRT<br>KREQELATLKKTLEEEASLHEVTVQEMRHKYSQEIAAINEQMESVKKTKASLE<br>KAKQQLEAENADLTQELRTAGASRQECERRRKQAEAALAELQAKAAEAERTR<br>AELAERATRLAQEAEAAQQQLEQAELRASAAAKSAATVESQLADVQGQLEEE<br>TRQKLAISSRLRQLESEREALQEQVEEEEEARRALDKQLTAAVTQLQEAKKRQ<br>EEDSEQIQQLEEAKKKMAKDIEALQRQVEELQAANDKLDKSKKKLQAELEDA<br>NIDLEAQRAKVLELEKKQRNFDKVLAEEKAVSEQIAQERDAAEREAREKETK<br>VLSLTRELDDMSEKVEELERGRRQLQAELDELVNSQGTADKNVHELEKAKRA<br>LESQLAEHKAQYEELEDELQLTEDAKLRLEVNMQALRAQFERDLQAREEQAE<br>EKRRALVKQLRDLEAELEDERKQRAATTQQRKKLEADMKDLEQQLEMHDKV<br>KEDALKQLRKLQAQAKDSARDAEEARAARDELAAAAKEAERRLKSLEAELA<br>QVSEELSAAERARRAAEAERDELQEEMGSSASKGSLLLDDKRRLEARIATLEE<br>ELEEEQGNSEILMDRARKAQLSIEQLTTDLAAERSTTQKLETQRMLLERQNKE	

			LKAKLVELETAQRTKTKATIASLEAKIANLEEQMDTEVKERLAQKLXXQEKKI<br>KEISLQLEDERRQTDQYKEQLDKSNARVKVLKRQLDEAEEEISREKAQKRKAQ<br>REMEDMMESQETMTREINNLKNKLR	
28	LMI_gi_225194719	pro-phenoloxidase 2 [Locusta migratoria]ACN81829	MAQQTPVAESLLYFYDRPKEPVFFPKGNNDVIFQLPEDYLSSRSKTIVDELPSRF<br>GRVKEEIQIRKIDLPDLSEPMKLGKRENFSVFIPYHRRLAARVIEVLMGMRTTE<br>DFRSAVAYCRENLNPLLFIYAFSVAMLHRPDTRNVNIPPLSETFPDKFIGGGLLN<br>RAREEANIFQTDGTRVPLRLPLDYTASDVEEEHRVAYFREDLGINLHHWHWH<br>LVYPFDGPDVVVRKDRRGELFYYMHQQIVARYNFERFCNKLGRVKRLQSLYE<br>PIKEAYFPKLDSFVASRVWPPRNSNVMLSDVDREQEQLKFDIQDLERWRDRMF<br>EAIHRGTVLQENGQEMQLTEDGGIDILGNIMESSTISINRRYYGNLHNLGHVAL<br>ALCHDPDNRHLETFGVMGDSATAMRDPVFYRWHAFVDDVFQEHKATLPPYT<br>LQKATQFDNVNIDSFTVQTDGSKAPNLLETTYDQNDVNLTRGMDFTPRGNVF<br>ARITHLNHKPFTYTINVNNRGPQRLGTVRIFMAPKFDERGITLLLRDQRLLFIEL<br>DRFTTNLKSGNNAIKRRSTESSVTIPFEQTFRDLTTNRPTDNEGLSRFNYCGCG<br>WPQHMLIPKGTPEGFPCELFVMISNYNDDKVDQAADTESCDDASSYCGIRNKK<br>YPDKRAMGFPFDRPPRPGANTLADFRRNLPNMRVIDVQIRFIPPASNPNIVD	17
29	LMI_GLEAN_10141796	vinculin-like isoform 1 [Bombus impatiens]XP_003493644	MPDLERPVMAVSRAVANLVKVGRETINSSDDPILKQDMPAALMRVEGASKLL<br>EEASAMLKQDPLSDFNVFSTSSUKKLIEGSRGILQGTSALLLCFDESEVRKIIRE<br>CKRVLDYLAVAEVIETMEDLVQFLKDLSPCLSKVSREVGGREKELTHQVHREI<br>LIRCLDQVKTLAPILICSMKIFIHIIAQGGKGAEEAAENRNYLAQRMTDEINEIIR<br>VLQLTTYDEEEWDADNLTVMKKAQNAIEGKIRAAHDWLEDPLALRGGVGEK<br>SVRQVLEHANRVADRALPGDSEALHRLCGEVTTMTDALCELRQDGKGATPQ<br>AECLARGIQEKLSALQSLVGRAVQSVERSGVQQPAHTVTGRLEQARRWLANP<br>ERDDRGLGQRAIALIVDEGKKVAEGLPGVHKAEILQLCDEVDMLSHQLSDLCH<br>QGLGNSARAQDIARALSQKLYELKNRIQDAVVSRVVEDFVDISTPLKQFTDAV<br>HLPEDTPGAEYNFAEKAKQLQNFSSRASKTARMVAAGGSGGNKKLAEALLSS	17

			ASQVESLTPQLINAGRIRKSYPESKAADEHFENLVSQYSDSIQRVRALCDEATD SADFIKMSEEQIQKHTVLCEDAIHTNQRQKMVDNTSSIARLANRVLMVAKQES DNSEDPQFVDRVNRASVAVQGSLPPMVHGAKAVAMNTQDSVAVARWRESN RALLTAVGQVRQAVTVSPDLPPPPDMSSLNINDEIVEPFQPSLQYNYFTEKVGQ PLREQFGADLGATTSASPVGSRPHSSAALSPLPKWARGDNADLLYQELSPDDF SGRGILHQRLDYAPPRPPLPGGEVPPPRPPPPETDDEDDMFSHAPLPNQPIMVA AHGLHQEVRQWSSKDNDIIAAAKKMAVLMGRLSKLVRGEGGSGSKRDLIACA KSIAEASEEVTRLAKELARECTDKRMRTNLLQVCERIPTIGTQLKILSTVKATM LGAQGTEEDQEATDMLVGNAQNLMQSVKETVRAAESASIKIRTDAGIRLRWV RKQPWYQY	
30	LMI_GLEAN_101435 64	hexamerin-like protein 2 [Locusta migratoria] ACU78069	MRTATVVVLSLLAALAAAAVVPHSEAGKELLEKQDKLLRLLYHVQQTTLVKE EQEIAKTYKPIEHVDNYQYKDKVELFWKYYVDVGFLPKGEVFSVYYQKHFYQ ARALFELFFFAKDFETFYKIIWSPVQRKNEIRREVGTKEAPYVFYSNYSGYPVA SNPEELVSYFTEDIGLNSYFAYLSYKYPYWLNPKNYSLPEYQYRGESFFFVLQQ LLARYYLERLSNHLPDVKAVDYNHPVLVGYYPELRLQNGREAPARPEGIFARN VDILYVEEIRNYERRIRDGIDYGYLAGYNYEKYNVREKDYTNVLGNILEGNED SINKEYYGAFYRNLISLFGHIVDPVHRYGVPASVLEQPETQLRDPLFYRIGKRV LSIFYHYKNLLRPYTHEDLYLPGVTVEDITFDKLVTFFDTFDFEINNALTLSKPE EGAGFSYVARQYRLNHKPFFYHLKVKSEKEVDSVVRVFIGPKYDALGREYSLE ERKQYYVLLDTFNYKLVAGENDIKRSSNDFPFYAKEAPSWYDLYKATSSAVK GEDKFFLDKFRSHFGFPQRLALPRGTRSGLPLSVFPPVPQASPDAKNPILEHGDL HAAGFPFDRRVVEFEFNVPNAHFDETFVVHRRVEDLNTTA	17
31	LMI_GLEAN_101962 47	beta-actin [Diabolocatantops pinguis] ACV32627	MCDDDVAALVVDNGSGMCKAGFAGDDAPRAVFPSIVGRPRHQGVMVGMGQ KDSYVGDEAQSKRGILTLKYPIEHGIITNWDDMEKIWHHTFYNELRVAPEEHPI LLTEAPLNPKANREKMTQIMFETFNAPAMYVAIQAVLSLYASGRTTGIVLDSG DGVSHTVPIYEGYALPHAILRLDLAGRDLTDYLMKILTERGYSFTTTAEREIVR	16

			DIKEKLCYVALDFEQEMATAAASTSLEKSYELPDGQVITIGNERFRCPEALFQP SFLGMESCGIHETVYNSIMKCDVDIRKDLYANNVLSGGTTMYPGIADRMQKEI TALAPSTIKIKIIAPPERKYSVWIGGSILASLSTFQQMWISKQEYDESGPGIVHRK CF	
32	LMI_GLEAN_100971 73	Tropomyosin-1, isoforms 9A/A/B [Camponotus floridanus] EFN72212	MDAIKKKMQAMKLEKDNALDRALLCEQQARDANLRAEKAEEEARALQKKIQ TIENDLDQTQESLGQVMAKLEEKEKALQNVKALANANSASSTSVSVAAESVT DRLHGNISNRDDSTGTRQQQQDSTSATHPTTPPSEDDPNVDIRNIERPAAEGSS QSDPEEAHDPETEELSRLRCTSERTEVIAEREIIRQRRCADYPGFAFGSSIFGSDT MMKFNIIRNELQNIKNSQLKRVSMYVLKGRDYFRRVNRRSRFFLSSAFGREFSF GNMTTNVQQGTLLDVLKKKMRQTKEEMEKYKDECEEYHKRLQVEIRRREEA ESEVAALNRRIQLLEEDLERSEERLATATAKLAEASQAADESERIRKALENRTN MEDDRVAILEAQLAQAKLIAEEADKKYEEVRFRDLIADIIIVFFRARKILENRSL ADEERMDALENQLKEARFLAEEADKKYDEVARKLVMMEQDLERAEERAEQS DSKIVELEEELRVVGNNLKSLEVSEEKANQREEEYKQQIKNLTTRLKEATQREE TFEEQVKALSGSLKEAEARAEFAERSVQKLQKEVDRLEDELVIEKEKYKIIGDD LDSAFVELIL	16
33	LMI_GLEAN_100219 33	tubulin beta-1 chain [Tribolium castaneum] XP_967267	MREIVHIQAGQCGNQIGAKFWEIISDEHGIDPTGAYHGDSDLQLERINVYYNEA SGGKYVPRAILVDLEPGTMDSVRSGPFGQLFRPDNFVFGQSGAGNNWAKGHY TEGAELVDSVLDVVRKEAESCDCLQGFQLTHSLGGGTGSGMGTLLISKIREEY PDRIMNTYSVVPSPKVSDTVVEPYNATLSVHQLVENTDETYVIDNEALYDICFR TLKLATPTYGDLNHLVSLTMSGVTTCLRFPGQLNSDLRKLAVNLVPFPRLHFF MPGFAPLTSRGSQQYRALTVPELTQQMFDAKNMMAACDPRHGRYLTVAAMF RGRMSMKEVDEQMLNIQNKNSSYFVEWIPNNVKTAVCDIPPRGLKMAATFVG NSTAIQELFKRISEQFTAMFRRKAFLHWYTGEGMDEMEFTEAESNMNDLISEY QQYQEATADEDAEFDEEQEGEVEEN	16
34	LMI_GLEAN_101707	fructose 1,6-bisphosphate aldolase	MTTYFNYPPPELQQELRQIAQAIVAPGKGILAADESTATIGKRFSEIGVENTEEN	16

	91	[Schistocerca gregaria] AEV89754|	RRLYRQLLFTADPSVGENISGVILFHETLYQKADDGTPFVELLKQRNIIPGIKVDKGVVPLFGSEDECTTQGLDDLAQRCAQYKKDGCHFAKWRCVLKIKRNTPSYQAIMENANVLARYASICQCNRIVPIVEPEVLPDGEHDLDRAQKVTETVLAAVYKALNDHHVYLEGTLLKPNMVTPGQSCPKKAQPMEIAQATVTALNRTVPAAVAGIVFLSGGQSEEEASVNLDAINKFPGKKPWPLTFSYGRALQASVLRAWSGKPENVAAAQEELKKRAKANGDAAQGVYAGGVSGAAADVGLFIKDHAY	
35	LMI_GLEAN_10123052	malate dehydrogenase, mitochondrial [Nasonia vitripennis] XP_001600547	MFSRVVRPQTVSVLQNGVRSISTSSQRHTKVAVLGASGGIGQPLSLLLKQSPLVSDLSLYDIVNTPGVAADLSHIETPAKVKGYDGSNQLKDALKGCEVVIIPAGVPRKPGMTRDDLFNTNASIVRDLAQACAEVCPKAIIGIISNPVNSTVPIASEVLQKAGVYDPNRVFGVSTLDVVRANTFIAEAKIRKAGEGGKDPDGLGSEAAVKAQGVGDRCMLRYRIVHIALGHSLMVAIHPDEEWGLDPTKVNIPVVGGHSGVTIIPLISQATPSVEFPQDQLKALTERIQEAGTEVVKAKAGAGSATLSMAYAGARFAFAVIRALKGEQNIVECAYVRSNVTEAKYFATPLLLGPSGIQKNLGLGKLSDFESQLLKAAIPELKKNIQKGEDFVNKK	16
36	LMI_GLEAN_10097518	alpha tubulin [Schistocerca gregaria] AEV89775	MRECISIHVGQAGTQIGNACWELYCLEHGIQPDGQMPSDKTIGGGDDSFNTFFSETGAGKHVPRAVFVDLEPTVVDEVRTGTYRQLFHPEQLITGKEDAANNYARGHYTIGKEIVDLVLDRIRKLADQCTGLQGFLIFHSFGGGTGSGFTSLLMERLSVDYGKKSKLEFAIYPAPQVSTAVVEPYNSILTTHTTLEHSDCAFMCDNEAIYDICRRNLDIERPTYTNLNRLIGQIVSSITASLRFDGALNVDLTEFQTNLVPYPRIHFPLVTYAPVISAEKAYHEQLSVAEITNACFEPANQMVKCDPRHGKYMACCMLYRGDVVPKDVNAAIATIKTKRTIQFVDWCPTGFKVGINYQPPTVVPGGDLAKVQRAVCMLSNTTAIAEAWARLDHKFDLMYAKRAFVHWYVGEGMEEGEFSEAREDLAALEKDYEEVGMDSIEGEGEGAEEY	16
37	LMI_GLEAN_10142607	alpha tubulin [Schistocerca gregaria] AEV89775	MEGEVKIMKAEGGGGSTYDDCLGFIWVYGKERLVTPFRELDEHECISIHVGQAGTQIGNACWELYCLEHGSPMGGQMPSDKTIGGGDDSFNTFFSETGAGKHVPRAVFVDLEPTVVDEVRTGTYRQLFHPEQLITGKEDAANNYARGHYTIGKEIVDL	16

			VLDRIRKLADQCTGLQGFLIFHCFGGGTGSGFTSLLMERLSVDYGKKSKLEFAI YPAPQVSTAVVEPYNSILTTHTTLEHSDCAFMSDNEAIYDICRRNLDIERPTYTN LNRLIGQIVSSITASLRFDGALNVDLTEFQTNLVPYPRIHFPLVTYAPVISAEKAY HEQLSVAEITNACFEPANQMVKCDPRHGKYMACCMLYRGDVVPKDVNAAIA TIKTKRTIQFVDWCPTGFKVGINYQPPTVVPGGDLAKVQRAVCMLSNTTAIAE AWARLDHKFDLMYAKRAFVHWYVGEGMEEGEFSEAREDLAALEKDYEEVG MDSIEGEGEGAEEY	
38	LMI_GLEAN_100964 50	alpha tubulin [Schistocerca gregaria] AEV89775	MRYDRPQLNCSIRRVSRAATLSDIGLVPVDAIDRWDFTRHGLHLNRKGKGKL AGLIAKSLRGDTAIHEGFVFQRECISIHVGQAGTQIGNACWELYCLEHGIQPDG QMPSDKTIGGGDDSFNTFFSETGAGKHVPRAVFVDLEPTVVDEIRTGTYRQLF HPEQLITGKEDAANNYARGHYTIGKEIVDIVLDRIRKLADQCTGLQGFLIFHSF GGGTGSGFTSLLMERLSVDYGKKSKLEFAIYPAPQVSTAVVEPYNSILTTHTTL EHSDCAFMCDNEAIYDICRRNLDIERPTYTNLNRLIGQIVSSITASLRFDGALNV DLTEFQTNLVPYPRIHFPLVTYAPVISAEKAYHEQLSVAEITNACFEPANQMVK CDPRHGKYMACCMLYRGDVVPKDVNAAIATIKTKRTIQFVDWCPTGFKVGIN YQPPTVVPGGDLAKVQRAVCMLSNTTAIAEAWARLDHKFDLMYAKRAFVH WYVGEGMEEGEFSEAREDLAALEKDYEEVGMDSVEGEGEGAEEY	16
39	LMI_GLEAN_101112 05	aconitate hydratase, mitochondrial-like [Megachile rotundata] XP_003705474	MSRFDKDTYLPYEKLEQNLGIVKKRWGTPWLVAAQEIQRRCFHVSPLAAAAR KVAMSRFDKDTYLPYEKLEQNLGIVKKRLGRPLTLSEKVLYSHLDEPDKQEIV RGTSYLRLRPDRVAMQDATAQMAMLQFISSGLPKVAVPSTIHCDHLIEAQVGG DKDLARAKNINKEVYDFLSSAGAKYGVGFWKPGSGIIHQIILENYAFPGLLMIG TDSHTPNGGGLGCLCIGVGGADAVDVMANIPWELKCPKVIGVKLTGKLKGWT SPKDIILKVAGILTVKGGTGAIVEYHGPGVDSISCTGMATICNMGAEIGATTSIF PYNRRMRDYLVATNRKEIAEEADKYVKSLFSADPGAPYDQTIELNLDTLEPHI NGPFTPDLCHPISKVGQTAKEKGWPVDIRVGMATICNMGAEIGATTSIFPYNRR MRDYLVATNRKEIAEEADKYVKSLFSADPGAPYDQTIELNLDTLEPHINGPFTP	16

			DLCHPISKVGQTAKEKGWPVDIRVGLIGSCTNSSYEDMGRCASIVKQALKHGL KSKIPFNVTPGSEQVRATIERDGIAETLRQFGGTVLANACGPCIGQWDRKDVK KGEKNTIVSSYNRNFTGRNDANPATHAFVTSPEIVTALSIAGTLDFDPTRDTLK GADGKEFKLDDPFADELPTKGFDPGEDTYQAPPSEGSKLKVNVDPKSQRLQLL EPFDVWDGKDLTDMTILIKVKGKCTTDHISAAGPWLKYRGHLDNISNNMFIGA VNSENGEMNKVKNQLTGEWGPVPDVARAYKAKGVKWVAVGDENYGEGSSR EHAALEPRHLGGRAIIVKSFARIHETNLKKQGLLPLTFANPADYDKIQPTDKISL LGLKDLAPGKPVTAEVKHADGKKEQIQLNHTMNEQQIQWFKAGSALNHMKR VAAGK	
40	LMI_GLEAN_101708 35	Nesprin-1 [Zootermopsis nevadensis] KDR09330	KEEILRQFIPLDETFWANKWKFHDSEVQWQVLLALSRPSLTGQSSEVGEYNDD DHRRDKDDGGSSGNSSPGSRTPRGAGGEAHSDATTEVLSADLPGGMCSWPAE STYLSLEEHTSRLAHRVAEDVFTNLDEEPHVSLAKDDWVSQLKASIASDAENL QKKIEISERDLSNLPTDNLLNMLNSITKIIEDLEERDQDLTRLERLLRELPQDVEC HSLTASLCSLRTHLLTLVSNAEQNRATVESAWQLQQRRQEEVKQYQILLSELE QWLKSAQAVLSADIQEASAPVLREHIKELKLREAQLAELAQLCRNLQDYPDV HHLATVLFQQLSQMELAIREASEQINIRLGALKVQIQKIISNASSMPYEEVVLTT PVPCKDETTVLHAESQTGPSLLRDLSEKAPSEVYDRTVGKTQPVDMEIQTDLLS PVLTLKPDSDVAVQVQPFPSLDGVEKDSIKVLQKTEGNEETIEISTKKIEIPKPVS EVSDTDLLVQLKSIGRKGEESKDDIEPGSSELKITHTVPQSFETVVVEPGESTTE VIVDADGTKRVIVRKTHRTVVMRQHRDIIQEHQSTMLPSDHTAVPAAEFITLSG ITTDSQQKLPEKIEEFPEGPVTDYVQTSSSVVPVTEMRVVEPIGSQNIEVQVDSS EFPAQFHSSTDTSRVPVVQHVTRKIIRRIRRRIIRKVVVINGKEQVTEEVIEEPEEI EVFEDGGPKIAIEGQNNHSVFVGEPGMDIHIQKEISYKTVPAGTALSATQLFINA ERSPGLPLETEHQQEGSILPEQKQAFEETLMIQPLETDTQQRETDIDQYLYSSVS SVSDQHKEHGEVDVPKEIIPEVLPKAPVDDAKVEQLPEQSQKPEPVFDTSPLAG KTTDETTVKSLPEPETSQFETLASETKLTIGKETDTHISQLDDDSLPKPTLHELE	16

			HDGTKEEDKSTPLQDSSETSLAESTNITVSLTGSSGPSKMTEEEIVFLDTSLHSL<br>DSTHKVETDDTGYEPEDRTYDEPSLSGDEKKQIKKKKRRRKPKEKLKEPSSPD<br>SIAAEVEGVESLVDTVATDKTDYEPQSKPDMSGMMDRKGTKRKRDKKDDEE<br>MMPEMSMEQIEDYSPRTSLEDFLQAERGSAQMAIPVEKTAVTDTVMDTIHTVE<br>TPTLDSSEQLDKTEPHKLLDKPEYISEEPDHGTIVDVTIKEAEPETTSMPSNDET<br>LPIVTKVPVETVAEEVIVELKEGQLIQDEESVKTSDLATQTVIADLTPTQEGEM<br>QTSPDLFKMPLSETIETSAQTVPDSIPVQEVSSQTETLLAGDQSVTETETLPDGK<br>GDHLAPTAEISMQTSPEAVTLCDEESQTVTERSQPVESSEFSMQTVTKDLIPTQE<br>GVVQTSPDLIEVPQTETVVTSVQTNIVPTQEISMQTLPQETVDDVMDTSVQTQK<br>EDLVPTQEVQDDSIQTTPVIEAETAEIHIQTNKEDLTPTVEKTIQTVTPELDTVV<br>APDTLEISVQTVKEETVPTAETSMQTVTPEPKHEPVVTVTVTQQTSPDLITITEIT<br>KPEEYEVSQMTSESVEPPEESQKNIAQLSEVSVQTKIPESPEKFEVAVEAVPSKE<br>TDMTETSIQTSPVQIRSPEESTSSSVSDEPYEIQIQASITYTSSGLPTVETKMTKPG<br>DSETVHDQESNITKESYEQKYTSDTGGYDQIYKKDSAVKDVVSKKQSDVSHK<br>KPVLSQQMKTEHLPSDVPEITRSSLQSKDPKAVSGTVNEALSLEAPEISVYTVPT<br>AYSSEEGSEPAPEILVGADEKKEEKTPEKQKKSEKEEKGDELKSHVADTPSEYA<br>SMIKETGVPSYKDIVELPDTQEKILKSGDQVSPKKDGAVVKEKHALPVQDVSS<br>PLEKLIDDREKSITVSKDTDTHLEKPIATVIPSTVQTDPEKPIEKLQDSNLKETTE<br>SFPDISKDPLIDVTTVSLLDDRPLKHTELQQFIMEEKQKSSPKNRAQSVVEGAK<br>WKQASNLVVERVKNLQNARRTTHLSGMLCLASLHEVVAEESVEQRNTALQH<br>NLNLLRTAAKSRDVIVIQRTVITTIETISTWLETVEYRVFLNRQKHVTASPNQEE<br>IKKQFITLKDEISTIEENVGELENILEQTEGICNEDDKARMKECVMSLQEQVKA<br>VEEVAQETEERAAREHGRWEEFLNAVNNISVLVEELRNQVEELSESDLPAREK<br>LCQLDEAETMNQSHRERTSQLLLTARQLLRDFPGREIPPETYAAHETTRIIELAV<br>ACQRDKVLQQLALADEYEQTLREFAQITDVADALVENDTAVQDLNHLQEEM<br>QKHRKFFVNLSHCRSILESLEGNLDSETRAAHHALHQSLHRRATAILDKAAAR	

			AQQMALAASRWTVLEQGMRDECGWLAVAQQRLPDLTAVTTSDFHQYISLFQ<br>SLAADCATHQARIAHLLETAQRLQELVTCVGLETRYDETVSVLLQLQDDVAA<br>NLRRLVAFREHWTAYDALATRLDNWIISADKELDQISNSTGALTGSMRQFWE<br>LKAQYELHNGLRSEAGNCFDAAMRTLPVADEMLQRQLHAQLEERWQRLSGR<br>VITVRDAATRGIEDESVPLADRLAVLAGELKELQAALDDMHGVLRSPDELDLY<br>IQRLQVMKERLDGIQEELGRLGLLSAEQSDMVGSLLGGARQLELQVSEELEAA<br>EQVREQLRTVSRGLTRATTAQGRTADVLADCERVHDQGGDVISRAIDRCIVAG<br>QDLAQQWQQLMALRQQLHAVPASRLRLSLSPQGVERQMSALQDTHAALEQQ<br>QGQLLQMLRARFALWQNFERRLETVRQQVAEAGYMMELLTVQGSVDLDRLV<br>KATERLQGLSGSLEEREQLLTELHEAAAALCAPEVSRQVAAAVEEAVASWQE<br>TRRSLGELCSRYRHAVDLWRRYRDASDAVRAWADQQLLEAADAPHVDADA<br>VQVCRGRQRAFTQYTTLTAKAVRTLDLGIVSRLNVSLLDSGFGQFAERCQEVR<br>EVRLALDRLAQAAEQRAAGHHELQAARRFLDSVQQSLAAVEEATEQDAEQQ<br>LGALRSHLLALGKTESQLQQLRGTAETSVDDAPVVEVLALWQQVFRETFQQY<br>HRLSARLVRGQDGAAALRLWQEYLVHVQAFLSAGVPGDYKALAEHQHLCEV<br>HQNLLTNQQNVLLSKNDSDGLLGSGLIESSVVEQFASLTNLHNETLARIMDRH<br>AEVRARLDAWDRYRQDQAVLLAWLRDTERERARLQLRYIHLRCLPKILLRIQ<br>ALIEKIPSGESQAESLQQQQSSLLSFCDEALATSIRMEHAAIVQRISNLGAALET<br>WREFLERITNLATTFEQQAETVTTAFNEVRSTIEQSGADTPVSVNAVTERLERL<br>KELRTQLADRTADLESLGVTLEQLKECAAPQDMKAATQRVWLLWQAQADLE<br>HQLTLLCHRLEERIGLRTLFDTRQSRFMTWASEVEARLERSSTADAEEVLRRLE<br>TELQAEVALKRREAEWLTRTGRELLAELDTNADPELKERVERVETRWRELQD<br>LGRSRAARLAELLQTMSQLELRLAELRAWLHQVESKLATPLVFENCSKETVD<br>QKLREHEELQKAIEKQSSNVGEVLNLCELLLSNCEACKATINTESITIATDMLE<br>QRWKNVCGQSAERKRRIIAVCQLIQDLQKLCNEQEDWLTAQELVLKELDSRQ<br>DLRSQSEIQSFVSRIDGVLKEVETRAPALRILDQSYSRLHKESTLLEPDNMRQLT	

			SGARSVLRRWHDLAPAAAAAQQRLFRELQRYRDFAAAHSRALVALTQFDISL<br>TQAQHLTEQLDMAQMTQLETDLEALSPLVQEVEEVGNKVLQRCSPEETSELR<br>GMLNECQLLWTDIRERFWQLHEKCSSQVVDSAIQVETLRFEQDTAVQVDTLK<br>LTSDDAYRYELESAMRECSVNLDQLEDVSSRTKITPRSSEISKALAAARSSIELV<br>RHLSSLLPENRAAADRVRVLGERFDRMVANIQAREQQEKELSESTRLTCPLCS<br>RRNWQQLDNDLWRLEQWLQYAEATQSAQSSPPNNIEKLEDTIQDHREFLMDL<br>DSHKSIVVSLNIVGSHLADHTEDIEHAEQLRARLSTTNSRWDAVCRAAARWQ<br>AQLQVALMENSEFHSIIEELVEWLEKTENIIRQTEPVDLADDVTVIEAKYNKFR<br>ELRSDLERCEPRVMSLQEAADQLLRQPEEEGGVNTGSNTWARLTDLRLRLQSL<br>RRLTGVYVLKLGAVLGREPSEMGTPVPSATAATSLAKTIHSLKSNVSSVCTVD<br>LVLHFLLFFQLVNQTSSSQADVSLHGDQQQASDTDVDTSVLTRGYRFLGRVIR<br>ASLPIQALMLLLLGVASLVPTGEEDYACNLSNTFARGLEPMLRYPNGPPPV	
41	LMI_GLEAN_101045<br>18	Pyruvate carboxylase, mitochondrial [Zootermopsis nevadensis]KDR22588	WNIATRLYSSHVEYKPIRSVMCANRGEIAIRVFRACTELGIRSVAIYSEQDKMH<br>MHRQKADESYLVGKGLPPVQAYLNIPEIIRIAKENEVDAIHPGYGFLSERADFA<br>QACIDAGLRFIGPSPKVVQQMGDKVAARQAAIEAGVAVVPGTPGPIDKLEEAK<br>AFCLKHGLPIIFKAAYGGGGRGMRVVRTMEELEESFQRATSEAKAAFGNGALF<br>IEKFIERPRHIEVQLLGDRGGNVVHLYERDCSVQRRHQKVVEIAPAPNLDPKVR<br>DKMTTQAVNLAKHVGYENAGTVEYLLDEQGNFYFIEVNARLQVEHTVTEEIT<br>GIDLVQSQIRIAEGMTLPELGMSQDKIHPQGCAIQCRVTTEDPAKNFQPDTGRIE<br>ASKISGEGMGIRLDGASAFAGAIISPYYDSLLVKVISHAHDLQSSSAKMDRALR<br>EFRVRGVKTNIPFPLNVLENQKFLNGTVDTNFIDDHPQLFRFRPAKNRAQKLLN<br>YIGTVLVNGPSTPLATQLKPAEVNPQVPEIPLGNNVKRQEPEKSELAPPPGLRDI<br>LKKAGPEGFAKYVRQHKGLLLMDTTFRDAHQSLLATRVRSHDLLRISPYVAH<br>RFSQLFATENWGGATFDVALRFLHECPWERLQDMRAAMPNIPFQMLLRGANA<br>VGYTNYPDNVVYKFCELSVQSGMDIFRVFDSLNYLPNLILGMEAVGTAGGVIE<br>AAISYTGDVSDPSRTKYNLDYYLKLSDELVKAGTHILSIKDMAGLLKPAAAKL	16

			LVTALRDRHPDVPIHIHTHDTAGAGVASMLECAKAGADIVDVAVDSMSGMTSQPSMGAIVAALQGSDLDTGLDLSNVSEYSAYWEQTRTLYGPFECATTMKSGNADVYLNEIPGGQYTNLQFQAYSLGLGDFFEDVKKAYRDANLLLGDIIKVTPSSKVVGDLAQFMVQNKLTAKEVEEKAEELSFPKSVVEFLQGYIGEPHGGFPEPFRSKVLKGMPRVDGRPGASMPPMDLDSLKNKLKEKFPDVTDNDVMSAALYPQVTEDFLTFRKHFGPVDKLDTRIFLIGPKVGEEFEASTQFPVTIEKGKTLGIKTLAMAEDLTPAGEREVFFEMNGQLRSVFIKDKEAVKELHIHPKADKSVKGQVGAPMPGTVIDIRVKVGEKVEKGAALVVLSAMKMEMVVQAPISGVIKKLDIEPNMKLEGDDLLMTIE	
42	LMI_GLEAN_10168000	Annexin-B9 [Zootermopsis nevadensis] KDR08631	MADIKSEAKIAGVEDVTIPLWNYQLDLGRLDLEPHIVSGVIACFRLGNSASAFRDLVGRKRKERITRNGMGGVEGRLEVVGVVPCDSDAGRQGSLITRDASLVLPVSPQQSPSGGVCCAVFAATFKSIRIYQQMTMVRSKIVLVFLSISLGSVRTILVSRLVKWLYDLLLPRNTTMSSRQYYPVHCTPTIYPADPFDAKADAETLRAAMKGFGTDEQAIIDVLAKRSIVQRLEISEEFKTLFGKDLLSELKSELSGNFEDAIVALMTSLPNLYAKELHDAICGIGTDEEALVEILCTLSNYGIKTIAAVYEETYGTSLESDIKGDTSGHFKRLLVSLLMANREESQDVDPSAAKADAEALLAAGEGQWGTDESTFNKILVAQSYAQLRRVFHEYEQLAGHDIEEAIKRECEGSLEDGFLSIVKCVKNKTGYFAERLHDAMAGMGTKDKTLIRIIVTRSEIDLGDIAEEYAKKYEKSLAERVQEDCAGDYKSLLVGILN	15
43	LMI_GLEAN_10143558	hexamerin-like protein 1 [Locusta migratoria] ACU78068	ADKNFLLRQKKLLEVFWHVGQPTIDPEQRKISETFNLEENVNNFKDPELVKKFVNYYNHGYFKQRGEQFSIYNKLDRIQAKALVDLLYQANDFETFYKTSVWARDHLNEGLFVYALNVAKLHREDLFDVVLPPFYELYPQLYVSPEVIKEAWEATLEGKTFSKENPYVIRVNYSGQPFARNADELVSYYTEDVGLNAYLDFMHYRYPFWAKMPEYNQANYTRRGDHFYYGIKAALARYNLERLSNGLPDVEVIDYTKPINGVVPPAPLVQYNLERLSNGLPDVEAIDYTKPINGVAPQGQLVQYVNTLEKRLREAVDAGYVFDSNFTKYSLRDPLSIEILGRIVEGNADSINDDYYGSFYRSLLSLAAAP	15

			GVSIVFVPACTLRNNNVFSERVFHFQSVTPASSFSNPTYYRVAKRVASIFDQFK DKLGPYSRDQLLLPGVNVESLTVDKLVTFFDDYDFELNNAIPVGSVEEGAKLN VLARVQRLTHKPFNYHVKVTSDKDIDVFVRFFFGPRYDVYGKELTPNEKRHN MLYTDSFVFKLKKGENELVRNSRQFNYYGQLPLGYSKLYHRTEQAIKSGSDV YVDDFVKKYGDPEGLLLPRGTRSGLPLSAYVILTPFTHTTISRINPYTDNVGAKI SFFPFDRPINEIEFDVPNSYFADAVVVHRSGDDIN	
44	LMI_GLEAN_10126189	citrate synthase 2, mitochondrial [Zootermopsis nevadensis] KDR22581	MIIDSCSRMKEESMTGWRYLIETNKGLEPSCREAPYCSARTKKVALPLLSAVAS RSASTDSTDLKAVLAEKAVRELERVKAFRKQYGGTKVGEVTVDMMYGGMR GIKGLVCETSVLDPEEGIRFRGYSIPECQKLLPKAPGGNEPLPEGLFWLLITGDIP NEAQVKYLSKEWANRAGLPSHVVTMLNNFPTNLHPMSQFSSAITALNTESKFL KAYSEGVNKAKYWEYVYEDAMDLIAKIPVVAAMIYRNTYKESKGIGAIDPKK DWSANFCQMLGYDNPEFTELMRLYLTIHSDHEGGNVSAHTVHLVGSALSDPY LSFAAGLNGLAGPLHGLANQEVLIFLKKVQAALGDNPSDDKLKEFIWQTLKSG QVVPGYGHAVLRKTDPRYTCQREFALKHLPDDPMFKLVAQVYKVVPPILMET GKVKNPWPNVDAHSGILLQYYGLKEMNYYTVLFGVSRALGVMASLVWDRIF GLPIERPKSMSTDGLMKMVKGAAKAA	15
45	LMI_GLEAN_10189307	Ubiquitin-like modifier-activating enzyme 1 [Zootermopsis nevadensis] KDR20513	MSSSRVLDSSVDPAAKKRKVATGDSVVASSCTSRSSEMEQPGSSNCAPEIDEG LYSRQLYVLGHDAMRRMASSNVLISGLGGLGVEVAKNVILGGVRAVTLHDEA SCTMSDLGSQFYLSEASLGKNRAEASLKQLSELNNYVRTQAYTGPLTEEFLKQ FRVIVLTNSKLEEQLRIAETARAFNIALIIADNKGLFSQVFCDFGDEFTVVDTNG EAPLTAMIASVSNDVEGVVTCLDDTRHGLEDGDYVTFSEIVGMTELNNCDPIKI KVLGPYTFSIGDTSNYSKYVSGGIVTQVKVPTVMQFKPLKEALKEPEFVITDFS KFERAPQLHLAFQALHKYHEQNGDYPRLWNNDDATAFRALCKKLSVNGGSD YEVGNDVLDIFPKICRGDVSPLNAATGGIVAQEVMKACSGKFTPIYQWLYFDA LECLPKDLTAENPENYMPTGSRYDGQIAVFGKEFQEKLGKLNYFIVGAGAIGC ELLKNFAMMGVGAGEGGKVIVTDMDLIEKSNLNRQFLFRPHDVQRPKSTTAA	15

			RVVKQMNPHVNIEAHENRVGPETERVYDDTFFEALDGVANALDNVDARIYM DRRCVYYRKPLLESGTLGTKGNTQVVVPFLTESYSSSQDPPEKSIPICTLKNFPN AIEHTLQWARDSFEGLFHQAAENAAQYLSDPSFIDRTMKLPGVQPLEILESLKK ALVDQRPLSFEDCVAWARCYWQEQYNDQIRQLLFNFPPDQLTSSGQPFWSGP KRCPKPLEFSVENSLHLDYIVAAANLKAEVYGIPQNRDREAVARIAAKVEVPV FVPKQGVKISVTDSQDQMSNGTGAVDHDRIIQLQKDLPSVSDLGNLQIKPLEFE KDDDTNMHMDFIVAASNLRAENYGIPIADRHKSKLIAGKIIPAIATTTSVVAGL VCLELYKLAQGFRNIDVFKNGFINLALPFISFSEPISAPKNKYYDTEWTLWDRF EVSGELTLKEFLDYFRTKYNLEITMLSQGVCMLYSFFMSRSKCAERMGLPMSE VVMKVSKKKIEPHVKALIFELCCNDDSGNDVEVPYVRYTLPN	
46	LMI_GLEAN_10113091	ATPase [Homo sapiens] AAA35578	MTSTLIKTSDEDRESKFGFVFAVSGPVVTAERMAGSAMYELVRVGYYELVGEI IRLEGDMATIQVYEDTSGVTVGDPVLRTGKPLSVELGPGIMGSIFDGIQRPLKDI NELSNSIYIPKGVNVPALSRTAQWDFSPVSVKVGSHITGGDLYGLVHENTLVK HKLLLPPRAKGTVTYIAEPGNYTVDDVVLETEFDGERSKFTMLQVWPVRQPRP VTEKLPANYPLLTGQRVLDSLFPCVQGGTTAIPGAFGCGKTVISQSLSKYSNSD VIIYVGCGERGNEMSEVLRDFPELSVEIDGVTESIMKRTALVANTSNMPVAARE ASIYTGITLSEYFRDMGYNVSMMADSTSRWAEALREISGRLAEMPADSGYPAY LGARLASFYERAGRVKCLGNPDREGSVSIVGAVSPPGGDFSDPVTTATLGIVQ VFWGLDKKLAQRKHFPSINWLISYSKYMRALDDFYDKNFPEFVPLRTKVKEIL QEEEDLSEIVQLVGKASLAETDKITLEVAKLLKDDFLQQNSYSPYDRFCPFYKT VGMLKNMIAFYDMSRHAVESTAQSENKITWNVIRDSMGNILYQLSSMKFKDP VKDGEAKIKADFEQLHEDIQQAFRNLED	15
47	LMI_GLEAN_10165425	Glycogen debranching enzyme, partial [Zootermopsis nevadensis] KDR16306	MLDAKLAIMKLMVCLGTFFKKVQDNISFLTVYLLEGFCYKKIKKCKFEKMPH KQSETNIGSILGFINKNPEHNQDQIRVLTLNDGEHQDSTLYRLQKGWRLQFRLG PTLFGRHVVIYCNHPDSIEEEFDRNQYRPLHWQCDSGTPSGDDTSSYAEVCIRR AGSFHYYFIYEGSDVPGPQGSGFFTVDPVLTYGCDEVLPQDCIQCQTVLAKCL	15

			GPFHTWEKKLQVARESGYNMIHFTPIQELGGSNSSYSISDQLKLNPLFNEENKTI<br>SINDVESLITKMRDEWKVVSICDVVLNHTANESQWLKEHPECTYNLVNSPHLR<br>PAYLLDALLHQLTVEVAEGKWQFSGIPAEVNCEEHLSAIRSALHGFFLPQIKVS<br>ELYMVDVNALVSEFQSLVRKRLPPVQVVSQENMKLKIIQDPQYRRLKSNVDM<br>ELALQLYNVYRNDCFDEETRIRRCTEDFKMCLDKLNNAIHLEVEAHLQAAVN<br>NTIAGIRYFRVDMNGPRIKQVSLKNPLVPRYFTDYGTPNSLKEFEDVMYSADA<br>CYIMAHNGWVMNDDPLRNFAAPENNVYLRRELIAWGDSVKLRYGERPEDCP<br>FLWQRMQEYVEQTARVFDGIRLDNCHSTPIPVAEYLLDAARRVRPDLYVVAE<br>LFTNSDQKDNIFVNRLGISSLIREAMSAWDSHEEGRLVYRYGGEPVGAFHQPSL<br>RPLVPSVAHALFLDLTHDNPSPIDKRSVFDLLPSTALVSMACCASGSNRGYDEL<br>VPHHIHVVDEYRQYAAWTEDDDAAGVGIRSGIISAKKALNKLHFDLGMSGFN<br>QVFVDQMDADIVAVTRHCPATHQSVVLVSYTAFQHPDAYFSRGFVKPLRVEG<br>TVDEVILEASLFHKKVKSGGARFQKFDKFEKNEKIINGLQDYEVELKEHIPLSES<br>QMLQIGDSGDPNVTQINFKNFQPGSVVAIKVSLHNKAAEAIKKLRNFVWLSRL<br>PQQDASAHDLATELSNIVSSLSLSDLNAALYRCDSEELDYGKGIGVYNIPGFGS<br>LVYCGLQGFMSLLCDITPINDLGHPFCNNLREGNWMIDYVSHRLKQYSGTIEL<br>GLWLEKSMSPLKEIPRYLVPSYFAVIIALTYLRLLNQAWSLMSLFVKDGSTFLR<br>GLALGSVQCASVVHSAPLPQLSPNLAPPLPLTRLDSEGKEVQACVTLSAGLPHF<br>ATGYVRNWGRDTFIALRGIFILTGRYQDARYHILAYAACLRHGLIPNLLDGGS<br>KARFNCRDAVWWWMHCIHSYVNEAPDGYNILKDKVSRIFPTDDSPALPAGQV<br>DQPLHDVMQEALSVHFQGLCFRERNAGRSIDAHMTDKGFNNQIGVHPETGFV<br>FGGNSYNCGTWMDKMGSSDKAGNRGKPATPRDGSAVEIVALCKSTLRWLAS<br>LHEQGRFPYNSVQRSSKNGTLITWTYKQWEEKIAANFEKCFYIDVEPSPNEPRP<br>DLVHRRGIYKDTYKASQPWADYQLRCNFPVAMVVAPEMFDPKHAWIALKKV<br>EELLLGPLGMKTLDPADWNYCGDYDNANDSADPKVAHGFNYHQGPEWVWP<br>LGYFLRACLHFAREVGGIAELERTAARVRSILAKHFTVLQTSPWRGLPELTNK	

			DGAYCHHSCQTQAWSMASIIEVLHDLNQLQESHPIILDSSSN	
48	LMI_GLEAN_101367 78	Neither inactivation nor afterpotential protein C [Zootermopsis nevadensis] KDR20620	RFELLPDPGDRYQLGDRIGRGVYSEVFEAVDTQAGGKRVAVKVQPVSEDLLQ DIREEFRVLRDLSTHPNLPDFYGVYMKRATNRVDPDKLWFVMELCDGGPVTD LVQGLHKQNRKMNEDHIAYILKESVKALAHLHEHNVMHRDVKGSNILLTKEG EVKIVDFGLAKELESAEGRRSTCLGSPCWMAPELISSCAGVAAEVGEQAGYDN RVDVWAIGITAIELGDGKPPFQDMHPTRTLWNPPPTLYRPANWSKLYNDFITE CLEKNPENRPYMVEILEHPFLTALPENDFHLSQELKKLLSELHLTEGRRGREVA VRGGALVAEGASDPEPMRVEDLAALEPVHEDAVLAELHERMRRGHCYTFVG DVLLLLNPNEQQNIYGPEVHTKYQFKSRSDNAPHIFAVADRAYQDAMHHEEP QTILLAGETASGKTTSLSHLVRHLAFLGKSGNGQGDRVEKALSVLRAFGNAAT PLNGNSTRHVFQVQLTFGLTGKLSGAIFWIYQLEKWRVTTKYRQQANFHVFY YWYDGSAAEGKLKQHGLNDGRQYRYLRAAXXXXXXXPREDAAGNARRFKE LRAALRDLGVDHEQEETLWRTLAAIVQLGEVRFQDDGHGQADLRDHDLAAN VAKMLGVDEKKFAWALTNYCVIQQGTAVRRRHTVDEADEARDVLARGIYSR LVDWLVNLINFKMSFARAVFGDHYCVNILDLFGFECFKTNSLEQLFVNTLNEQ MQYHYNQRVFAWEMQEAADEDIQLASLQFYDNKPTVDALMAKPSGLFYILD DASRNPLGSDFIAETLRGSSKGPHLRATGPFEFSVAHYTGKVTYNATDMAEKN RDFLPPEMIETLGQSTDPVIRQLFTNQLSRPGNLTFTPEEQEAATTPSTDQKKSK WGRALVAENTRMRRFNTESRGQYSQTRRMRTAAAVFRAVSLELLKNLSVGA GSGGTHFVRCLRADLTGAPRGFQPEVVRQQLRDTARARQSGYPHRITFAEFIR RYKFLAFDFDENVEVTKDNSRLLLVRLKMEGWKIGRTKVFLKYYNEEYLART YEIQVKKIIKVQTMMRAFLAKRNVAGKLKNKKDVKNGGKSKTETEKKESAM SQEEAAVVIQKQFRGYQVRKQMGHKEKSKPALFIRQYCRKWKAKSMFQVLL LYRAARHQDLVYFSQQVHLYNQNAMSTIANTAMAVKMNHIDPGSRATIELGK PQPTVWKLPF	15
49	LMI_GLEAN_100560	Full=Actin, cytoplasmic A3a; Flags:	MCDEEVAALVVDNGSGMCKAGFAGDDAPRAVFPSIVGRPRHQGVMVGMGQ	14

	04	Precursor [Helicoverpa armigera] Q25010	KDSYVGDEAQSKRGILTLKYPIEHGIVTNWDDMEKIWHHTFYNELRVAPEEHP VLLTEAPLNPKANREKMTQIMFETFNTPAMYVAIQAVLSLYASGRTTGIVLDS GDGVSHTVPIYEGYALPHAILRLDLAGRDLTDYLMKILTERGYSFTTTAEREIV RDIKEKLCYVALDFEQEMATAASSSSLEKSYELPDGQVITIGNERFRCPEALFQP SFLGMEANGIHETTYNSIMKCDVDIRKDLYANTVLSGGTTMYPGIADRMQKEI TALAPSTMKIKIIAPPERKYSVWIGGSILASLSTFQQMWISKQEYDESGPSIVHR KCF	
50	LMI_GLEAN_101562 23	Transitional endoplasmic reticulum ATPase TER94 [Zootermopsis nevadensis] KDR08983	MADGRNPDDLATAILRKKDRPNRLIVEEAVSDDNSVVALSQAKMDELQLFRG DSVLLKGKRRKETVCIVLSDDSCPDEKIRMNRVVRNNLRVRLSDIVSVQSCPD VKYGKRIHVLPIDDTVEGLTGNLFEVYLKPYFLEAYRPIHKDDTFIVRGGMRA VEFKVVETDPSPYCIVAPDTVIHCEGDPIKREEEEEALNAVGYDDIGGCRKQLA QIKEMVELPLRHPSLFKAIGVKPPRGILLYGPPGTGKTLIARAVANETGAFFFLI NGPEIMSKLAGESESNLRKAFEEADKNAPAIIFIDELDAIAPKREKTHGEVERRI VSQLLTLMDGLKQSSHVIVMAATNRPNSIDGALRRFGRFDREIDIGIPDATGRL EILRIHTKNMKLADDVDLEQIAAETHGHVGADLASLCSEAALQQIREKMDLID LEDDQIDAEVLSSLAVTMDDFRYAMSKSSPSALRETVVEVPNVTWEDIGGLEN VKRELQELVQYPVEHPDKFLKFGMQPSRGVLFYGPPGCGKTLLAKAIANECQ ANFISVKGPELLTMWFGESEANVRDVFDKARAAAPCVLFFDELDSIAKSRGGN VGDAGGAADRVINQILTEMDGMGAKKNVFIIGATNRPDIIDPAILRPGRLDQLI YIPLPDEKSREAIFRANLRKSPVAKDVDLTYIAKVTHGYSGADLTEICQRACKL AIRQSIEAEVRRERERANNPNLAMDMDEEDPVPEITRAHFEEAMRFARRSVSD NDIRKYEMFAQTLQQSRGFGTNFRFPSSNPGNPSQGGNSGSQPGTFQEDGDDD LYS	14
51	LMI_GLEAN_100852 05	transferrin [Romalea microptera] AAQ62963	DKICVPETVLDECNNLAKQDGVHLTCVPARDRLECLDKVHTHKADFVPVDPE DMYIAANNGDNHFAVFKEIRTKEEPNEEFRYEAVAVIHKNQPLRSVQDLKGLK SCHTGVGRNVGYKIPLTKLSNMHVIGAMNDKSLTARENELRELSNLFSKACLV	14

			GNWAADPELNKRLKKEYANLCALCEHPDVCNYPDYYSGYDGALRCLSDNGG EVAWTKVYYVKKHFGTGYNPNDYAYFCPDGSKKPILGRACRWAARPWQGY LASDQLLSEVPQLRQQLKLANTLGEQQDAKWLDKVLLILKGKTAAVDNAQPL SPQAYLNKANYSDVIGRNFGPNDPIRFCVTSTAALAKCRSLSAGAFSRDIRPRL GCVVKGGVRDCLAAIRDGEADVISLDGGGTVTPGIHFVCREYDLKPILSEVYG PLQDVYYAVAVIKKNSNYQSFADLRGAKSCHTGIGRTAGWVVPVHTLLRLGL VQRSECPAAKAVSDFFSGGSCAPGALLAENNPTGQNPSKLCDLCVGNSDKND ASTKCYPDVGEDYFGYTGAFRCLAAGAGDVAFVKHSTVLNNTDGHNSEAWA RDLKSSDYELLCPDGGRKPLDQFESCNLAAVPGHLVVTSKTKNEHELEAIRQA LLAAANLYSNRPDLFRLFGPYEGKHDLLFKDSATGLKPVYEESQALRDYEQVL TSTRSC	
52	LMI_GLEAN_100713 72	14-3-3 protein zeta [Zootermopsis nevadensis] KDR15025	MSVDKEELVQRAKLAEQAERYDDMAAAMKAVTETGVELSNEERNLLSVAYK NVVGARRSSWRVISSIEQKTEGSERKQQMAKEYREKVEKELREICYDVLGLLD KYLIPKASNAESKVFYLKMKGDYYRYLAEVATGENRNTVVDDSQKAYQDAF EISKAKMQPTHPIRLGLALNFSVFYYEILNSPDKACQLAKQVAVVDDSQKAYQ EAFDIAKSKMQPTHPIRLGLALNFSVFYYEIINSPARACHLAKQAFDDAIAELDT LNEDSYKDSTLIMQLLRDNLTLWTSDTQGDGDEPQEGGDN	13
53	LMI_GLEAN_100813 07	Glutamate dehydrogenase, mitochondrial, partial [Zootermopsis nevadensis] KDR15400	MFHLKAVSRTVGALKQSTQFDAGALLKVCPAAFQLRNSRSYSDHQIPERLKD VPTAENPRFFDMVEYFFHRACQVAEDKLIEDMKERRMSVEDRKKKVKGILML MQGCDHIIEVSFPIKRDSGDYEMITGYRAQHSTHRTPCKGGIRYSEDVTRDEVK ALSALMTFKCACVDVPFGGGKAGLKLNPQKYSEHELEKITRRFTLELAKKGFI GPGVDVPAPDMGTGEREMSWIADTYAKTIGHLDINAHACVTGKPINQGGIHG RISATGRGVFHGLENFIMEANYMSMIGTTPGWGGKTFIVQGFGNVGLHTMRY LHRAGAVCIGIIERDGSIYNPEGIDPK	13
54	LMI_GLEAN_101278 81	Tubulin alpha-3 chain, partial [Anas platyrhynchos] EOA98266	MDRAASRVGDSNPPSGIDVFVVVSSWMGNSSCFPVDLMTSWVVGCPVGNSVE LAQYFDYVARDLLQIRPETARRESINTGPDLFEKQSQRECISIHVGQAGVQIGNS	13

			CWELYCLEHGISPDGHMPSDKVMGGDDSFNTFFSETGAGKHVPRAVFVDLEP TVIDEVRAGPYRQLFHPEQLITGKEDAANNYARGHYTIGKEFVDIVLDRVRKL ADQCTGLQGFLIFHSFGGGTGSGFTSLLMERLSVDYGKKSKLEFAIYPAPQVST AVVEPYNSILTTHTTLEHSDCAFMCDNEAIFDICRRNLDIERPTYTNLNRLIGQI VSSITASLRFDGALNVDLTEFQTNLVPYPRIHFPLVTYAPVVSAEKAYHEQLSV AEITNSCFEPANQMVKCDPRNGKYMACCMLYRGDVVPKDVNAAIATIKTKRS IQFVDWCPTGFKVGINYQPPTVVPGGDLAKVQRAVCMLSNTTAIAEAWARLD HKFDLMYAKRAFVHWYVGEGMEEGEFAEAREDLAALEKDYEEVGLDSVEVE EGLEEY	
55	LMI_GLEAN_101833 79	14-3-3 protein epsilon [Schistocerca gregaria] AEV8977	MSERDDNVYKAKLAEQAERYDEMVEAMKKVASLDVELTVEERNLLSVAYK NVIGARRASWRIISSIEQKEENKGAEEKLEMIRGYRSQVEKELKDICSDILGVLD KHLIPCASTGESKVFYYKMKGDYHRYLAEFATGNDRKEAAENSLVAYKAASD IAMTELPPTHPIRLGLALNFSVFYYEILNSPERACRLAKAAFDDAIAELDTLSEE SYKDSTLIMQLLRDNLTLWTSDMQGDGETEQKEQLQDVEDQDVS	13
56	LMI_GLEAN_101814 26	2-oxoglutarate dehydrogenase E1 component, mitochondrial [Zootermopsis nevadensis] KDR11185	MQLWTSTIRSDSRPEKHGNSYTPGEPQFSYDLNITSEDDVDESLLSAQPKMHR ARSVLTNLLPLTPLIPHAAGPERFAAWLLRSGSATSSLSVTSTRNYIASVAAEPF LNGSSSAYVEEMYNAWLADPKSVHVSWDTFFRSSSAGAAPGQAYQPPPSLAP PGKNQVPITSLAPYLGASVPALDGQVNEKIIDDHLAVQAIIRSYQARGHLVADL DPLGIMYADLHSKYKDRQGAPPEIVVRQYMLVFLISNTCSGLLPSKLCCQIRGH HIAKLDPLGISSADLDDKTPPELLYSHYSFGKRASTTYSQDLQRKVASMMEKE TDMDRVFKLPSTTFIGGKEKALSLREILRRLENAYCRHIGVEFMFINSLEQCNWI RQKLESPGVMEITNDEKRLILARLTRATGFEAFLARKWSSEKRFGLEGCEILIPA MKQIIDSSTEFGVESIVMGMPHRGRLNVLANVCRKPLEQIFTQFAALEAADDG SGDVKYHLGTYIERLNRVTNKNIRLAVVANPSHLEAVDPVVQGKTRAEQFYR GDGEGKKVMSMLLHGDAAFCGQGIVYETFHLSDLPDYTTHGTIHIVVNNQIGF TTDPRHSRSSPYCTDVARVVNAPIFHVNSDDPEAVMHVCKIAAEWRATFHKD	13

			VVIDIVCYRRNGHNEIDEPMFTQPLMYRKIRKTPPALDKYADKLIKEGVVTAEE VKDVRDKYEKICEDALENARKETHIKYKDWLDSPWSGFFEGKDPLKVSPTGV KEDTLVHIGKKMSSPPPNAAEFEIHRGLERILKARMEMVEARTVDWALGEAM AFGSLLKEGIHVRLSGQDVERGTFSHRHHVLHHQKVDKATYRPLCHIYPDQGP YIVCNSSLSEFGVLGFELGYSMTNPNALVCWEAQFGDFNNTAQCIIDQFISSGQ AKWVRQSGLVMLLPHGLEGMGPEHSSARLERFLQMSSDDPDYFPPESEEFAV RQLHDINWIVANCSTPANYFHILRRQIALPFRKPLILMTPKSLLRHPEARSSFDE MTEETQFLRVIPENGPASQNPGGVKKLVFCTGKVFYDLRKAREEKNLTNDVAI TRIEQISPFPYDLVKKECAKYPNALLCWAQEEHKNQGSWTYVQPRFHTAINGT RDFVYAGRPTAASPATGSKVHHLRELAALLDEAMAI	
57	LMI_GLEAN_10042900	mitochondrial F1-ATP synthase alpha subunit [Locusta migratoria manilensis] AGO59887	MALLSFRLASAVAKHLPAATPQVFKSIPNGRTKCLIAYWKTARTVDCVEVRLV IDCTEISGLTWPAAQITNRNIHVSCSQRAAEISSILEERILGAAPKADLEETGRVL SIGDGIARVYGLKNIQADEMVEFSSGLKGMALNLEPDNVGVVVFGNDRLIKEG DIVKRTGAIVDVPVGEELLGRVVDALGNPIDGKGPLKASKRFRVGIKAPGIIPRI SVREPMQTGIKAVDSLVPIGRGQRELIIGDRQTGKTALAIDTIINQKRFNDGEDE KKKLYCIYVAIGQKRSTVAQIVKRLTDSGAIGYSVIVSATASDAAPLQYLAPYS GCAMGEFFRDNGKHALIIYDDLSKQAVAYRQMSLLLRRPPGREAYPGDVFYL HSRLLERAAKMNDAHGGGSLTALPVIETQAGDVSAYIPTNVISITDGQIFLETEL FYKGIRPAINVAGSMKLELAQYREVAAFAQFGSDLDAATQQLLNRGVRLTELL KQGQYVPMAIEEQVAVIYCGVRGHLDKLDPSKITAFEKEFLQHIKTSEAALLA NIAKEGKITDEIDVKLKKIVTDFVANFQG	13
58	LMI_GLEAN_10122197	Bifunctional purine biosynthesis protein PURH [Zootermopsis nevadensis] KDR19778	MQSGIEPGPPRSPGRRLATASPWSLVVQWSGQETSACQNFVLAALLSVSDKTG LVSLAEKLHSLGLTLVASGGTAKALRDAKLAVKDVAEITGAPEMLGGRVKTL HPAVHGGILARLTDSDQADMKKQGYNLIRVVVCNLYPFVKTVSKPDVTVPDA VENIDIGGVTLLRAAAKNHDRVTVLCDPADYDSVVAEIQKSPGKDTELATRQR LALKAFTHTAQYDDAISDYFRRQFSGGSGQLTLRYGXALGLPAATSFKHVSPA	13

			GAAVGVPLTKDEARLCMVEDLVEQLTPLAAAYARARGADRMSSFGDFVALS RTCDAVTARIISREVSDGIIAPGYTPEALEILSKKKGGAYCVLEMDPDYEPDPVE RRTLFGLTLEQKRNDAVVDESLFEGRTVSQRKELPAEAVRDLIVATIALKYTQS NSVCYAKNGQVIGVGAGQQSRIHCTRLAGDKADNWWLRQHPRVIGMKFKKG VKRAEISNAIDNYVGGSVGKDMEYDAWASVYEEVPPFLTQQERQEWIKQLKS VALSSDAFFPFRDNVDRAYQSGVEYIVSPAGSANDAEVIKACDERKITLVHTNL RLFHH	
59	LMI_GLEAN_101540 78	hexamerin-like protein 2 [Locusta migratoria] ACU78069	MSRGRASPTAGADSRLISHLSHYKRPSGRRTIRTSSASNMRTATVVVLSLLAAL AAAAVVPHSEAGKELLEKQDKLLRLLYHVQQTTLVKEEQEIAKTYKPIEHVDN YQYKDKVELFWKYYVDVGFLPKGEVFSVFYQKHFYQARALFELFYFAKDFET FYKTAVWAREHLNEALFVYSYTVAVLHREDTKDVTLPAPYEVYPQLFVNAEV IQQAYDAYLRGEVGTKEAPYVFYSNYSGYPVASNPEELVSYFKDVGLNSYFA YLSYKYPYWLNPKNYSLPEYKYRGESFFFVLQQLLARYYLERLSNHLPDVKAI DYNHPVLVGYYPELRLQNGREAPARPEGIFARNVDILYVEEIKNYERRIRDGID YGYLAGYNYEKYNVREKDYTNVLGNILEGNDESINKEFYGAFFRNLISLFGHI VDPVHRYGVPASVLEQPETQLRDPLFYSIAKRVLSIFYHYKNLLKPYTYEDLYL PGVTVDDITFDKLVTYFDNFDFEINNALTISKPEEGAEFSYVARQYRLNHKPFF YHLKVKSEKEVDSVVRVFIGPKYDALGREYSLEERKQYYVLLDTFNYKLVAG ENDIKRSSIDFPLYAKEAPSWYDLYKATSSAVKGEDKFYLNKFRSHFGFPQRLA LPRGTRSGLPLSVFTIVTQASPDAKNPILEHGDLHAAGFPFDRRVVEFEFDVPN AHFDETFVVHRRAEDINATV	13
60	LMI_GLEAN_101737 35	Phosphoglycerate mutase 2 [Zootermopsis nevadensis] KDR20387	MSCPCSGKTDPKQAKYTIVMVRHGESEWNQKNLFCGWYDANLSDKGRQEAA NAGKALKDAGYKFDIAHTSVLTRAQKTLEAILKEIGQTDLPVEKTWRLNERHY GGLTGLNKAETAAKYGEEQVQIWRRSFDVPPPPMDEENPYYKTITEDPRYADG PKKEEFPMFESLKLTIQRTLPYWNDVIVPQLKAGKRILIAAHGNSLRGIVKHLD EMSDEAIMGLNLPTGIPFVYELDATLKPVVSMKFLGDEATVKAAMEAVAAQG	13

			KTK	
61	LMI_GLEAN_10053226	Rab GDP dissociation inhibitor alpha [Zootermopsis nevadensis] KDR21130	MDEEYDAIVLGTGLKECIISGMLSVSGKKVLHVDRNKYYGGESASITPLEELFA RFGAPAPGETYGRGRDWNVDLIPKFLMANGSLVKLLIHTGVTRYLEFKSVEGS YVYKGGKISKVPVDQKEALASDLMGMFEKRRFRNFLVYVQDLKEDDPKTWK ETDPNTCTMQQLYDKFGLDKNTQDFTGHALALYRDDDYLNRSAIETIRRIKLY SDSLSRYGKSPYLYPMYGLGELPQGFARLSAIYGGTYMLDKPIDEIVLENGRV VGVRSGEEVARCKQVYCDPSYVPDRVKKTGQVIRCICLMDHPVPNTRDALST QIIIPQKQVGRKSDIYVSLVSFTHQVAAKGWFIAMVSTTVETDNPEAEIRPGLDL LGPIRQKFVSVSDYYEPTDLGSESQIFISTSYDATTHFETTCLDVLDIFRRGTGED FDFSKVKLDLGDEEQ	13
62	LMI_GLEAN_10105168	titin [Tribolium castaneum]XP_008191512	QSKQSPVEELLRQADQVISTQRPRAEVYAAMAQSLGRAWADVNAHLDLRRQ LLDLNVSYHRHASSCTEAMRVLEVACDEASGLPTDAESTKEALSALHEARRVS LESLMPALQDGRALLDKLRELVALGSLDSRPNAIRVAASYANSQVEHWMEAL HDRRQQLEMAFTRRRERLEQCLALMLVARDLRALEDAVDRRRQELADVLGD SAASAEILLTEHRRLLPEAKEFQDRGVKITKATEQLVSSNHFAAEEAVAQAYK VLQQCAEYSEALDNRESLLQRAISFFRTAHNAMQKLDQLEQQLKMNELPVTSP QLAKIHAAAVQTIEDVTDGPLKEGYALLDVAGHGMPGSEGIKRAVEELENRKI SLNGLCIAHKEEHLRVSLAMQTYKEKEEEIFSWIVQIAEAFLQGHQDMGSVLA MAQDFLQLHQQLLGDVQVNEERVKIDEKVETLHNLWLKLKNVVEARIFLAQI YVKFHTVAVDLANEFDALDDDLKKSPEKISDETIRNVEQKWLYIQQLYTQLQS IGKSVKDECSKIGDPYLDTKRAALCVDTLLEHFGGRQLTLTQSWQTWQSTVTV ERETQILWERSVVDSSKTYDWASKLDGHLYPVMTSDSSSSKVIVRELEDKLQT VLPELKKAQTEIELRVKTAENLALKGESQGQKEEITAKLIDLHGKLQVVATDY QILLQMLIAFFRNLAELEKTIENLESQYRHTRLPSSVSDIELLLKEHEASRQAVL ELFKFTQNEMEQIVIRIKQQEPDAAAKHDIDSLTRLLEIKRIAWESAWTDRKLQI EQHRQLCQFDSDLHQINATLNDLSRQLDAIKGQYGESLVSAKATSQAFVYFEK	13

			TIETFVSTAENMLSTERVSSPHVEHELKQLQTRWQQFHKQVVESRRLIDLSMQ<br>YFTLVEEAEEWFREGSKLLVTIARKSTSVKKPEEATQLLAEVDTFLKPGELRQD<br>ERIRKISTLAIQLYGEERSKQVSLVLSENKEMLDSFNVISEELNTLATNLKAAEE<br>QREKQRKVRNTLRDLRTWHSCRNDDDIFMKDRPWKGTNNRIQGYNIISFGHH<br>GLCWLEKHIHTHIHTDTTAFSGHFDQFTGLSMAREQQAVEASLAAARAEAAA<br>AKAAAAAAEEARKAAESAAKLLKETSVTVHKVDVEMQVAMPEAKQLERPQK<br>SPSPPLKKAKIVTVTPKNVAPVFSVPLNDAVIQEGEKFTFCCCVTGTPMPEVIW<br>YKDGISIQNNPDYQTTFKDGNCSLTIEETFTEDSAKFTCKATNAAGTAETSATL<br>SVKEAEPEEQLSPPQFTQPLQNSTAREGSSHELRCHVEGVPLPTVQWFKDGTC<br>VDASPDYVITFNNGEALLRFEEVFLEDQAEYSCRATNPLGSDSCCARLTVEPLE<br>PTETPAFTVPLTNVMARAGQKIKLECEVTGLPSPQISWSHNNKPVKETRDLKV<br>QYENNKATLVITEAFPKDAGIYVVTAKNIAGEASSSCNVSVKGRLPTETSDSEL<br>ASDMEPTKPSIQVPLKDIIVFEGKRARLDCVIVGQPEPEVIWYHDDRPVKESTDI<br>QLLFQGDRCSLIIQEAYLDDAGEYKVVAINSAGEANSKCSLSVKUPKIQPKAPE<br>VMEEGSGKAPKFTKLLTDVMVSETESAVLDCSVEGDPLPEIKWFLNNSEIKPD<br>AHRVFQHDKDGNVNLTIKSATPEDKGVYTVKATNKHGEAKCFSNLIIKSSGPA<br>EVRSKVDKQVFPAFKELFADRTAYVGDSTKFECIVTGKPTPKVKWYFNDEPVS<br>GKDFLVSTSGERQVLTIPCVANNHAGKVACVAENEVGKATCLALLTVQEAPT<br>LPSLLNSNISEQMESSSFSVKRSVFMQSSTSQVTSSTLTTGAEPKVEVHSYSSES<br>QESFKQSGDKPPVQVESHKTVEVHQVNKDKPDIHQTSFLKVSNAAEESQESST<br>SSYSTPVASPKPVRKSVGPRFISPLNGVIVDQGADVVLEGIIDGYPQPEVNWTK<br>NGQPLIAKEGSVDISWKLNKARVELKNVGVKDAGRYTCTATNKAGSASSTAD<br>VVVKKTVFPPVFGRRLQAQVVKVGDRVVMEVEVTGTPEPVITWMKDGEVIKS<br>GDQYRLKSQGNCHSLIIEKASLPYSGKYTVKAKNSGGEAQCIADFVVYQPEPT<br>VEVVKNVIFEDIIQSETQVLLITVEQKSHVPPATPENKGGVFPCAEDKETSTLKK<br>LTEETVQPHKATKEIESDKIIEKESIFSSTKIEKSVHVNETALTEIKSEETGIEDAPI	

			SKKSAFDFFVSKLKDSEEQPVKPKQPAKEDELPTRADGFDLIPEPPPEIGYIPKTE TSKKAKEDVTTRVKKLEEGHRILSDVEIPAGERATSPKPSMEGLAMDKLWAH KHPDAALKKSWPPPPAAEDKPIIPWVSKGTTEKQWPPPQTTVVSEKVETKSQK TELKEHKVISSKQPVMTTATKEQFNEVVEENILKPSEVKKTWPPGFKEDFEMK APPKRKSTPSKEVPSRPTPMPDTTVEPLLEPGPPPEIGFARPPPLERRQSYVETIE KDLEKDLEKEPTRHLAGAVRTIPPPPQKERSLPPHPKQKIDAQSDTSKISILQTD KYESTTESKMFERFPDLEPFPFKPDPPKPKQKVPPPPKPSKFVKGEFGGSDYESD FETTHIPPKWRPYESDTEDIQYRRVKPPVISSLPKRPKSTEPEPLPPSKFDKPPQF QGPPRPDVKVIKDSKIESVSKTETKIETKMEKQRKIVSAETKKISPPPLRQGSPPV FVQAVPPEHQEMKPTIDPKKPDSPKSKSKVVRTEFRESGYMADTDEPRQLNQIS QKSSSVKQEEFMVMKEVPAAPRQPSKDDRDSSLEPFPFEQEVSRTVSRRVTKV PPPPSPSKFVKGEFRESDYESDYEGRIPAKWRPADSDAEEPTYRPVRPILTPRAS SRTSAGGRTPTPPTEFDNPPQIGGPPRPKFEPIDKVTQPIKSDKISKSSEKVQVIFK PKPVTPKTVASMEVITATPAVRTTEHVVLEPGSPPEIGYIPPPKTGPKMSKSIFQ NATQTETSKVMNFAEETETSRRVMSVQQTTRVIKFGENDRKKSEPKLEPFPFRP DPERPRRSSAPPPPKPKKFVPGEFRESDYESDYEGRIKPKWTPANSDTDEPKYR RVRPPQVTRSVSVPSKSSTQVPTPMEFDTQQPYVPSPSESPCSTLERQSKTSVQK FQYETEQRRLRRVEEMKKRFSVTPPSTTTVKRTTSLQDLGVKPGEPPEYGFVSP STAANKSILKSATPAVTNGDKEPQAYREESRVAQYVFKTYLHILKGNYCNLKT ILYLCLGTKHIDPDTGLIYFKYDFGYEFGIVLPGEGGGKKESHEPKLKATGKQP GDIDVPVIHERTDGQKGTQKQTPVTPQFRPRKFVHSKAVKWDPGASESEMSEA ECDSGAPRRRLGSMADTPSPVSLSPSLPSLSPHHVGDASAASPGGSWLGKSPRG RGSTPTPPSTPGSTPTPTQQHQLRRPPVFITPLRDIAVVSGQTARFECIVQAEPAP NILWSKNGRIIESSEDYQLHYRNGVCRLTIPQAFPEDAGSYSCTATNMLGSSCT TATLQVPGEKR	
63	LMI_gi_329564865	glutathione S-transferase delta [Locusta	MPSVDLYYVPGSAPCRAVQMVAKAVGVDLNLKLVNLMEGEQMKPEYLKMN	12

		migratoria] ADR30117	PQHTVPTIDDNGLYLWESRAIIGYLVEQYAKDDSLYPKEAKKRALVNQRLYFD IGTLYARFADYYYPVMFGGASYDPEKLKKLEEAYEFLNKFLEGSDWVAGNSIT IADYSIMASVSTAEIIGFDIKKFPKVAAWFEKAKKEIPSYEETNQAGALEFKKLF DSMTAKK	
64	LMI_GLEAN_100961 04	calcium-transporting ATPase sarcoplasmic/endoplasmic reticulum type-like [Megachile rotundata] XP_003707160	MEDAHTKTVEEVVNYFNVDIDKGLTLDQVKKYQEKYGPNGKSIWQLVLEQF DDLLVKILLLAAIISFVLALFEEHEDSVTAFVEPFVILLILIANAVVGVWQERNA ESAIEALKEYEPEMGKVIRGDKSGVQKIRAKEIVPGDIVEVSVGDKIPADIRLVK IYSTTLRIDQSILTGESVSVIKHTDPVPDPRAVNQDKKNILFSGTNVAAGKARG VVIGTGLSTAIGKIRTEMSETEEIKTPLQQKLDEFGEQLSKVISVICVAVWAINIG HFNDPAHGGSWIKGAVYYFKIAVALAVAAIPEGLPAVITTCLALGTRRMAKKN AIVRSLPSVETLGCTSVICSDKTGTLTTNQMSVSRMFIFEKIEGNDSSFHEFEITG STYEPIGDVFLHGQKIKAAEYDTLHELATICVMCNDSAIDFNEFKQAFEKVGEA TETALIVLAEKLNPFNVSKIGLDRRAAAICVRQEIETKWKKEFTLEFSRDRKSM SSYCVPLKASRLGSGPKLFCKGAPEGVLDRCTHARVGTQKVPLTATLKSRILEL TRQYGTGRDTLRCLALATADSPMKPEDMDLGDSTKFYTYEVNFTFVGVVGM LDPPRKEVFDSIQRCRAAGIRVIVITGDNKSTAEAICRRIGVFTEDEDTTGKSYS GREFDDLSPSEQKAAVARARLFSRVEPAHKSKIVEYLQSMNEISAMTGDGVND APALKKAVVGIAMGSGTAVAKSASEMVLADDNFSTIVAAVEEGRAIYNNMKQ FIRYLISSNIGEVVSIFLTAALGLPEALIPVQLLWVNLVTDGLPATALGFNPPDLD IMNKPPRKSDEGLISGWLFFRYMAIGGYVGAATVGAAAWWFMFSPYGPQLSY YQVTHHLGCLGGSDDFKGVDCKIFSDPHPMTMALSVLVTIEMLNAMNSLSEN QSLIVMPPWSNIWLMASMALSFTLHFVILYVEILSTVFQVTPLTVEEWITVLKFS IPVVLLDETLKFVARKFADVGEVVVDKWA	12
65	LMI_GLEAN_101099 19	Hrp65 protein [Zootermopsis nevadensis] KDR15347	MENAANEMNIKTEEEHDRKGQNHAGQNNHPGGGRGEGRGNFDGRGGGFGG GRGRFSNRPRDDNRGRNPDDRLNERLAALMAPTHDLPPMDTTEKKFSGRCRL YIGNLTADVTDDEIREMFTPYGEVAEQFVNKEKNFAFIRMDYRANAEKAKREL	12

			DGSLRKGRSIKVRFAPPGAAIKVKNLTPWVSNELLEKAFSVFGDIERAVVIVDD RGKSMEEGIIEFARKPAAQMALRRCAEGCFFLTASLRPVVVEPYEPLDDTDGY QEKNLPKKNPEYYKMREVGPRFATPGSFEYEYGTRWKQLYDLHKQKEDSLRR EMKMEEEKLEAQMEYARYEHETEMLREQLRQRELDRERQKQEWEMKERQA HQLNSLLEQQEQALRANPTDYDAAAADPVIRDYDAVGAEAGTIPPDPKAFMD AYDRGASRYGEGARGDLREDLGGPAAGSRSRWGPGGADRRGEISVQLIRFTT DETLMQVLDKGLDDNGEWLHSRPSFLELQNFCYKDKTPNGDPGT	
66	LMI_GLEAN_10123367	protein disulfide-isomerase [Schistocerca gregaria] AEV89748	MNEDMYLNLLYVIMHVIKKQDTIKREAITPHERLTAVLRLLATGRTYEDFRYY FAKALGEIFQIHASFKPVACGGLQLESAGVVAGDLASPISVDWANTTSGRSGPV LAAEEDVLELTDEDFSTRIQEHDTMLVMFYAPWCGHCKKLKPEYAKAAGIIK DNDPPVTLAKVDCTEAGKETCNKFSVTGYPTLKIFRRGELSMDYSGPREAAGI VKYLKSQVGPSSKDLLTETAFEDFISKDDVAVVGFFEEESDLKLAFLRVADKL REKARFGHTSNRDLLKKGVSDGIILYRPKHLHNKFEPNSITYDGEAKKEAIESW INKEYHGLVGHRQRDNTQDFKNPLVVAYYGVDYVKNPKGTNYWRNRILKVA KSFASVFNFAISAKDDFQHELNEFGFDYVKGDKPVIFARNAKNQKFVLSDEFS METFEKFLNDLKDEKLEPYLKSEPIPEDNDGPVKIAVAKNFDEIVTNNGQDTLI EFYAPWCGHCKKLAPVYEELGEKMKGEDVAIVKMDASNNDVPEPYEVRGFP TLYWASKDGKSNPVRYDGGRELDDFIKYIAKQSTNELKGWDRKGKTKKQEL	12
67	LMI_GLEAN_10192650	moesin/ezrin/radixin homolog 1 [Riptortus pedestris] BAN21261	MIEASGFIEEKDSLCNEDGAVGMNDFEGDMSGDNVRDGLLEKGGSVGNDFRA MERGRMNVRVTTMDAELEFSIQHTTTGKQLFDQVVKTIGLREVWFFGLQYTD SKGDLTWIKLYKKVMSQDVKKENPLQFKFRAKFYPEDVAEELIQDITLRLFYL QVKNAILSDEIYCPPETSVLLASYAVQARHGDYNKAIHTPGFLANDRLLPQRV MDQHKMTKEEWEQSVTTWWNEHKGMLTEDAMMEYLKIAQDLEMYGVNYF EIRNKKGTDLWLGVDALGLNIYEQDDKLTPKIGFPWSEIRNISFNDRKFIIKPID KKAPDFVFFAPRVRINKRILALCMGNHELYMRRRKPDTIDVQQMKSQAREEK LAKQQQREKLQMEIAARERAEKKQQEYEERLRTMQEEMAKRQQDLEAAQD	12

			MIRRLEEQLKQLQKAKEDLEMRQNELQLMMERLEESKNMEAAERAKLEEEIA SKQEEVRRIQNEVEAKDEETRRLQEEVEEARRKQEEVKQALMAATTTPQHHH VVENENDEDDETENGDVSRDLATDDNIVDPVEERRTLAERNERLHDQLKALK QDLAQSRDETKETALDKIHRENVRQGRDKYKTLREIRKGNTKRRVDQFENM	
68	LMI_GLEAN_100512 80	malate dehydrogenase, putative [Pediculus humanus corporis] XP_002424808	MSSPKFHLFSISTFVEIGSDLLSANVRIFKVQGEALDKYAKKSVKVLVVGNPAN TNALICSKYAPSIPRQNFSAMTRLDQNRAKSQIAARLGIGVSAVRNVIIWGNHS STQFPDARNATADIGTGAPVPVYEAVKDDAWLRGDFVSTVQKRGAAVIAARK MSSAMSAAKAAADHMHDLWFGTAPGEVVSMGVISDGSYGAPPDVVFSFPVTI KNKQWEIAQGLPLDDFAKEKLEITGKELLEERTEALAVCESCNL	12
69	LMI_GLEAN_101540 80	hexamerin-like protein 2 [Locusta migratoria] ACU78069	MRTATVVVLSLLAALAAATAVPPSEADKELLEKQNKIIRLFYQVQQPTIIPEEQ EIAKSYKPIENIDNYQYKDKVEVFWKYYTEYGFVPRDEVFSIYYKKHFSQAKG LFELFYYAKDFDTFYKTAVWAREYLNPGLFVYSFTVAVLHREDTKFVTLPAPY EVYPQLFVNAEVIQKAYDARLRDVVSTRKEPYVFYANYSGFPVANNPEELVSY FTEDVGLNSFFAYLHYKSPFWLNPANYSLPAFKRRGDSFFFILQQLLARYYLER LSNRLPDVKPVDYANPVLVGYYPELRLQNGIEAPARPEGVYPSNFDLLFVERIQ NYERRIRDAVDFGYLYGYDFKTFNLNEKDLTDILGNVIEGNAESVNYEFYGSIY RYLISLFGHIADPYHKYGAPASVLEQPETQLRDPLFYRIAKRVISIFYQYKNQLK PYTKNQLEFPGVAIEGITFDKLVTFFDDFDIELNNALSFSKPEQGDNFNFIARQY RLNHKPFYYQLKVKSEKEVDAVVRVFVGPKYDVYGREFTLDEKKQYYFLLD VFNQKLNAGENEIKRSSKEFALFAKEAPSYYDLYQTTYRALKGEDKFSLDNVF AIVTPAVQGSEHPVLPYYDNQAAGFPFDRRVVEFEFDVPNVYFGETYVVHRRV EDINTTA	12
70	LMI_GLEAN_101089 70	succinyl-CoA ligase [ADP-forming] subunit beta, mitochondrial [Tribolium castaneum] XP_970725	MAKMLRQGCAVFEAISRKTPPKFLGGAAPVLQKQQQRNLNVHEYISYTLLRD AGIPVPKFGVAKTKQEAGKIAQELNVKDIVLKAQVLAGGRGKGSFKGGLKGG VRMVYTPAEAEDIAGKMLGDYLVTKQTGEKGRICNAVMVTERKFPRKEYYF AIMMERAFNGPVLIASSQGGVNIEEVAAENPEAIVYEPISIEKGLLKEQAEKIVE	12

			AVGLGDKKEETTQILLRLYNLFKSKDALLIEVNPYAEDASGNFYCLDAKMRFD DNADFRQKELFALRDWTQEDEKEVEAAKYNLNYIALDGDIGCLVNGAGLAM ATMDIIKLHGGEPANFLDVGGGATSQQVKEAFKIITADPKVHAILVNIFGGIMR CDVIAEGIIAAAKELNLRLPIICRLQGTNVDDAKVLIAASGLKILACDNLDEAAR LAVKLSNIVSLARSAKLDVNFEIPL	

Note: The proteins involved in TCA are lightened with blue background, and the structure proteins and molecular chaperones are lightened in yellow and red, respectively.

Supplementary Table S3 Detailed information about the 90 differentially expressed proteins in the locust head

Group ID	Hit Number	Accession No. in the locust genome database	Description	Score	Mass	Coverage	Sequence	Spectrum	Unique Spectrum	Peptide	Unique Peptide	Coverage	Ratio (G/S)	QuantNumber	Significance	BlastP
Up-regulated in the gregaria (as compared with the solitaria)																

1	138	LMI_GLEAN_10124366	[mRNA] locus=scaffold23180:328362:552548:+ [translate_table: standard]	381	102827	13.6	MSFNGGHERDSSRGR<br>SWLTSPNASPHAVRKE<br>RGQHRALPAAVSVFLP<br>EPLLLNQKVSQFASQEL<br>SAYRSPTAVGMPERIIE<br>KLDRNPALPNASPHAV<br>RKERGQHRALPAAVSV<br>FLPEPLLLNQKVSQFAS<br>QELSAYRSPTAVGMPE<br>RIIEKLDRNPALGDSAP<br>AGDPEFIKRQEQKKSDL<br>DEQLKEYILEWRKQRA<br>KEEEELKRLKEKQAKR<br>KVMRADEEKRLAERKK<br>QEEERRQREIEEKKQR<br>DIEEKRRRLEEAEKKRQ<br>AMMAALKSFGRHIKLKL<br>FLEGLPQQIKGRATCDS<br>THVIYQLTCLHCEAFYV<br>GMTSNKLSIRMNGHRQ<br>TAFVGNEDRPVAKHAL<br>VHGQHILAQFYDQSKN<br>KGPNFTITKKDASFNLS<br>SAQIERNKTKEQLEEEK<br>RISLSIRIKPLEIDGLSLD<br>KLRTKATELWETIVKLE<br>TEKYDLEERQKRQDYD<br><br>PKIQVASKYERRVDTRS<br>YDDKKKLFEGGWATLS<br>[illegible]	24	24	10	10	13.6	1.652	23	*	troponin t, invertebrate [Aedes aegypti].XP_001655223

2	428	LMI_GLEAN_10120585	[mRNA] locus=scaffold5556:268440:294506:- [translate table: standard]	124	25222	8	AARRIRQVSIDWAKMAE RVPEDQKSHFVAFKAK SDSYVRRMLANPENPP KIDWAYYKTRVPLKAM VDDFQKQYEALKIPYPP DNVTPQIEAQEKEVKKA IEAFVKASNERIAEYEAE ANKLRAVLPFEQMTMD DFRDAFPEQALDPVNR PTFWPHNPEEQLDYKR DAVATEGNGH	3	3	1	1	8	1.547	3	*	XP_002096273.1 mitochondrial F0 ATP synthase D chain, putative [Aedes aegypti], XP_001649125 ATP synthase, subunit d, isoform A [Drosophila melanogaster]
3	904	LMI_GLEAN_10019610	locus=scaffold[illegible] 128492:24787 :24999:-	50	8727	14.3	MAPLFIRYRTSNGISQQ EQGTIENQGSEDEAIAV RGSYSFIGADGKTYTIT YVADRNGFQPQGDFLP KRR	2	2	1	1	14.3	16.62	2	*	cuticle protein 1, Tribolium castaneum, XP_970381.1

4	433	LMI_GLEAN_10147206	[mRNA] locus=scaffold284:608189:695524:- [translate_table: standard]	122	109347	8	MVFLVFTHDPCDALGG NTMHSKMSGFHDPTD MLHYSAIFRTDENSTDG LQHKAIDLEKLFTPASD SGEVTPSRHRRMYASS SFYSPHHPTVEDQVEL ARRISNSLSDISNQQSK GQSMYVNRKKRSVKW VHEGQGTGKLSSSEEY SETTDENLSFKIPNVGP QEMVFKEPSKTPLKLV MDPRGQVQDLTSLRQQ GYNIETGALSPDICLDLV RDLNATKGKGAELFAK RRKKSEKWVVDETTVK KSSTSSTVTDYVSSSST LQRQSSIGAPSQLPPIV PSYLDTSRVQHAQKLN EIQDWSKWWWEDAYD RFYIRGSYMGGSWKVG KMVWGFHRDELGGHR ELLTNGSLDNFPMTKVF RDELESDIPEDIRFTLPR LRLVKSPWEAALETGS VDAAFQELKPLQSWSF SGAPTPAAPAPVLSSTF DSYPPTTPQPVPSEPA FTPLTPSIGVSQPPNQR VSGQEKDFLYKPKAPR GWSGTQQSQQNYGLK ESNLSSGQSTTTTATDA AATNILGTESNVIPTDST [illegible]	5	5	5	5	8	1.998	5	*	hypothetical protein LOC100160882 [Acyrthosiphon pisum],XP_001951692.2

5	367	LMI_GLEAN_10002441	locus=C189179892:18:439:+ [translate table:	147	12508	22.6	QAVLGAEAKEGEVNVVQVEALGCRQSIKVPVCVLKAGSIQQVLLDLTFPDAPVTFSLVQGNGPVHLIGNHAIGGPVDEEIDDEMDEEELGEEEEGEDGETAVSNIF	5	5	2	2	22.6	2.016	5	*	isoform 1-like protein [Maconellicoccus hirsutus],ABM55590.1 ;similar to nucleoplasmin [Tribolium castaneum]
6	876	LMI_GLEAN_10170245	[mRNA] locus=scaffold837:1540845:1541372:- [translate table: standard]	52	19605	18.9	MLSVAVFACLLAAAQAGLVAPAHLPAAHAVAYDHYAPPSYKFDYAVADSHTGDAKTQFEHRDGDKVTGAYSLIEADGTTRIVEYTADDHNGFQAVVKRLGPPTPAPPKAVLVAAPLSVAPPPAPVAHAPVYAAPAPAAHGPVYAAPAAHSAVLAAPAAQAYAYPAPAHGPAPYHA	4	4	3	3	18.9	4.181	4	*	similar to Cuticular protein 62Bc CG1919-PA [Tribolium castaneum],XP_967979.1

7	184	LMI_GLEAN_10106737	[mRNA] locus=scaffold7960:151509:258756:- [translate_table: standard]	298	56974	22.8	MPRTRQGDQIPFKKPEQSRDNYNRADDQTSTAEVGRNTINGPKVQIFIAQFENVFLTAKVLGGLGLKNGAALKPEGGFEQPPEEAEPRTKCTLIPGDGVGPELMYSVQEVFKAADVPIDFETFFFSEVHHMMSAPLEVVSKSIEKNGICLKGILATPDYSRTGELQTLNMKLRRSLDLYANVVHVKSLPGVKCRHDGIDAVIIREQTEGEYSALEHESVKGVVECLKIITREKSMRIAKFAFDYATKNKRRKVTAVHKANIMKLGDGLFLESCKKMAKLYPKIEFETMIVDNCTMQMVSNPKQFDVMVTPNLYGSIVDNLASGLVGGAGVVAGASYSANCVVFEPGARHTFSEAVGKNVANPTAMLLCAAKMLSHVNLHTYSEMIRNAVNRVLQDGKVKTKDIGGQSTTNEFTYAVIANLG	12	12	7	7	22.8	2.257	12	*	probable isocitrate dehydrogenase [NAD] subunit beta, mitochondrial-like [Nasonia vitripennis],XP_001607423.2

8	177	LMI_GLEAN_10097536	locus=scaffold29745:87587:90963:+ [translate table:	308	15076	50.9	MSNKSELACIYSALILVDDEVAVTGEKIQTILKAASVDVDPYWPGLFAKALEGINVKDLITNVGAGVGAGPAVGAAPAAAGAAPEAAPAEKKEEAKKKEESEESDDDMGFGLFD	5	5	3	3	50.9	2.088	5	*	protein [Ceratitis capitata],CAA72658.1;60S acidic ribosomal protein P1-like [Bombus terrestris],XP_003403

9	147	LMI_gi_1710156	[mRNA] locus=scaffold8098:194539:301329:- [translate_table: standard]	360	78096	22.6	LLLLLAAAATAAPNPEG EKVFLTRQREVLRLFVK IQQPAVIPEHIEIIKSFKW EEIEPKLQDKETVERFL QLHKEGSLLSREDDFSI LYAKHLWQAVDLFKILY RIPDYETFHKVAVWLRY NVNAGLFEYVARVVLLH RDDTRELLLPPPYEVLP QFFVSAPALQQATDAC LRGLCADENRPFVIRAN YTGSHALRSVESKLSYF REDVGLASYMAFMGAQ YIVPWVNASECPWPAL RMRGDLYYFLMRNLLA RYDLERLSNHMLPVTP VDLWEPVSEGYDPQLR LLSGKEAAARPEGLRPT HADVISLDDVISWERRV RDAAATALFLNEKKLES LEESDAVNRLASIVMGG PSSPAPNYYRSVSWGL QTLYGHIADPQHQYGM APSALDMHLTMYRDPL YYRIIKRIYGIFEVYKNNL PPYHAQDLTWGGVKIE ELKVVDKLVTFFDDFDI RLDNAIDVGRVEDIRKT NVVARQQRLNHKPFSY SLSVSSDKEQLALVRVF LGPADGAVPVEDLRHH FLVVDGFHTKLKPGNNT [illegible]	13	13	10	10	22.6	1.656	12	*	juvenile hormone binding protein [Locusta migratoria],AAC47391.1;methionine-rich hexamerin-like protei[illegible] micropetera],AAX14950.1

10	317	LMI_GLEAN_10123370	[mRNA] locus=scaffold413:330871:345940:+ [translate_table: standard]	167	44908	14.9	MESNVYKLIVKFDVAALFIISYALSNHLKRLQSTLVLAEHNNEKLIPITQNAISAAKKIGGDISVLVAGTKCAPVAAQLSKASGVSKILLAENDAFLGFTPETLTPLVLATHKQFNFSHIIAGASAFGKAVLPRIAAKLNVSPITDIIDVKAPDTFVRTIYAGNAILTLKSKDPIKVVTVRGTSFEPAPLEGGSAATEQAAAGDYKTDLVQFIGQELSKSDRPELTSAKVVVSGGRGMKSGDNFKLLYDLADKLNAAVGASRAAVDAGFVPNDLQVGQTGKIVAPELYIAVGISGAIQHLAGMKDSKTIVAINKDPEAPIFQVADYGLVADLFKAVPELTSKL	4	4	3	3	14.9	4.612	4	*	cxpwmw03 [Periplaneta americana],ABC61672.1;electron transfer flavoprotein subunit alpha, mitochondrial-like [Megachile rotundata],XP_003700429.1

11	6	LMI_GLEAN_10196247	[mRNA] locus=scaffold943:1627014:1628144:+ [translate_table: standard]	2826	48225	54	MCDDDVAALVVDNGSG MCKAGFAGDDAPRAVF PSIVGRPRHQGVMVGM GQKDSYVGDEAQSKR GILTLKYPIEHGIITNWD DMEKIWHHTFYNELRV APEEHPILLTEAPLNPK ANREKMTQIMFETFNAP AMYVAIQAVLSLYASGR TTGIVLDSGDGVSHTVP IYEGYALPHAILRLDLAG RDLTDYLMKILTERGYS FTTTAEREIVRDIKEKLC YVALDFEQEMATAAAS TSLEKSYELPDGQVITIG NERFRCPEALFQPSFL GMESCGIHETVYNSIMK CDVDIRKDLYANNVLSG GTTMYPGIADRMQKEIT ALAPSTIKIKIIAPPERKY SVWIGGSILASLSTFQQ MWISKQEYDESGPGIV HRKCF	107	50	16	7	54	4.3	30	*	beta-actin [Diabolocatantops pinguis],ACV32627.1;actin, clone 205-like isoform 1 [Apis mellifera],XP_623619.1

12	523	LMI_GLEAN_10130423	[mRNA] locus=scaffold3090:443554:514891:+ [translate_table: standard]	96	59618	8.5	MKKLAQDRIVWRTASNQSNQTEDHNNRRVMVSTTVTENGTESGKTVDLSAGGEYRVIGWDVDATGRTLIDEICQVAAYTPTDKFSQYIMPFGDLNFASRSRHNMAVVTIGRYRMLKDIKAGKVLKTKSEISALTDFVHWLEQISGKTKQSIILVSHENQKLASSLLLEALRKYNLLDRFCAVVKGFVNGFSVAEVKCAKTVKAFTLRTLSRMLLDKDEDLSSAVDRARLAYQIVHHLLDERQENKGSGDASVDIGESLKEFIWTLDKEEEHIASVKKFIERQNSLRPIFRPMMRIDRKESARASVLRRALAQAGVNYEDLSAAHSKDGSNGINELITSKVTNVKPNELEELKKIVNGHFNPETKKKTDESPQKVAPEKSVSEGASASSTPDTNNSPVKEPSTGANNTSATASSQSNDTSPQKSVNTGASSPASPESN	5	5	3	3	8.5	1.723	5	*	maternal protein exuperantia-like [Apis florea],XP_003697468.1;

13	102	LMI_GLEAN_10140130	locus=scaffold10215:85160:89430:- [translate table:	474	17556	73	MSWQDYVDKQLLASKCVTKAAIAGHDGNVWAKSDGFEVSKEEIAKLVQGFEKQDILTSSGVTLAGNRYIYLSGTDRVIRAKLGKVGVHCMKTQQAVVVSLYEDPIQPQQAASVVEKLGDYLVSCGY	15	15	7	7	73	2.307	15	*	profilin [Apis mellifera] ,NP_001011626.2

14	343	LMI_GLEAN_10116472	[mRNA] locus=scaffold627:165964:237097:- [translate_table: standard]	155	70422	9.2	MQGRVLLALCAVVAAA<br>TAQKDPYWLPGRNAMV<br>HLFEWKWADIAAECEN<br>FLAPKGFAGVQVSPPT<br>ENLAITQRNRPWWERY<br>QPVSYKLVTRSGDEAA<br>FADMVKRCNDVGIRIYA<br>DVILNHMTGTWSGEVG<br>TGDSVPDTAAYSYPDV<br>PYTKDHFHPYCLVSNY<br>QDANNVRNCELVELHD<br>LDQSQEYVREKLVEYL<br>NHLVELGVAGFRVDSA<br>KHMTPSELKLIYDAVND<br>LNTSFGFAEGSRPFIFQ<br>EVIDFGNEAISESEYTD<br>MGRVIEFRYSAEVGRG<br>FRQNNPLKWFYNFGEA<br>WAMEADENVLVFVDNH<br>DNQRGHGAGGDQILTY<br>KQPRQYKMAVAFMMA<br>WPFGVPRLMSSFAFTD<br>SEAGPPMDENQDIISPT<br>FNEDGSCDENWVCEH<br>RWRPIYNMIAFRTAVEG<br>TNATNWWDNGNNQIAF<br>CREDKGFIVINDDNVDL<br>KETLTTCLPAGDYCDVI<br>SGEPTETGCSGKTITVN<br>SDGTASFQILTTDDEGTI<br>AIHVNIQDVHAQQIVTTS<br>SVGTVGRERSEDRKGG<br>[illegible]	4	4	3	3	9.2	1.563	4	*	alpha-amylase [Blattella germanica],ABC68516.1

15	618	LMI_GLEAN_10172557	[mRNA] locus=scaffold249:700602:738570:+ [translate_table: standard]	81	43855	13.1	MASPNVKNEKIGIVGSG<br>LIGRSWAMLFAAAGYE<br>VCLYDILPEQVNSALADI<br>KTQLKTLEKEKLLRGKL<br>SADQQMSLIKGTSNLSE<br>AVKGAKLVQENVPERL<br>DLKCKVYQELDKVVDS<br>NTILSSSTSTFLPSLFSK<br>DLKHRSQIIVSHPVNPP<br>YYVPLVEIVPAPWTDKA<br>IIPRTRAIMEEIGQAPVC<br>LAKEIPGFALNRIQYCIL<br>NEVLRLVADGVLSVSDI<br>DKVMSEGLGMRYAFLG<br>PLETAHLNAEGMESYC<br>ERYAQTIYDVSMTMGP<br>VPKFEGPIVKELNKQLC<br>EMTPVEKLPERRAWRD<br>LCLTHLSTLKKKMSNTE	4	4	4	4	13.1	1.966	4	*	lambda-crystallin homolog [Nasonia vitripennis],XP_001601340.1

16	304	LMI_GLEAN_10098768	[mRNA] locus=scaffold4462:251565:275709:- [translate table: standard]	175	32067	21.8	MAHQLAANPLHFGRHIFWEFTQRLKDIGSKLTSTVEQTVESKKSELERIGQEKKQAAAALLEEQSKKTGDLLWQTKSELESAVIGTATSAATAAAASATTATKTAKQQAVDAFDATMADAEKAVDSTIKTASTTIDSKLDEAGKALDETRQGVQKAVQDTTKLASDKVSETTGTASKKASGLFNSTDQLYSDHPVSPWPGEAPRHPLTAFRVPFFTSQDKFKIGHPAAASCFGE	5	5	3	3	21.8	5.303	5	*	hypothetical protein Phum_PHUM011760 [Pediculus humanus corporis],XP_002422765.1

17	399	LMI_GLEAN_10164303	[mRNA] locus=scaffold140:728064:792094:+ [translate_table: standard]	135	100358	8.1	MKPKRRVRIFIVAAKRTPFGTMGGKFVQKKAVELQTVAAKAAMEAGNVKPEQIDSTVIGHVLAISSADGILIARHTALYSGVPIDRHAMSINRLCGSGFQSIVSGAQSILVGESKVVLTGGAENMSQTPYFVRNVRFGSPLGTKIEFEDSFWAGLTDTYCNLNMAMTAENLAVKYKLTRENVDKFALRSQMLWKNANEKGYFKEELAPVTVKTRKGEEIVNVDEHPRPFSTLEGLGALPPVFKKDGVVTAGSASGICDGAGAVIIASEEACKEYNYKPLARLVGYSVAGVEPSIMGIGPAPAIRNLLKVSGKSLTDIDLVDINEAFGAQVMACQEELKLDIEKLNVNGGAIALGHPLAASGARITSHLVHELSCRFLAKKEMAALTKGIFIVAAKRTPFGTMGGKFVQKSAMELQEVAGRAALAAGRVSPDQVDSVVIGNVLSVSSPDAIFIARHVSLKCGIPIERPALAVNRLCGSGFQAIVSGAHNILVGDSKVVLTGGTDNMSQAPYAVRNIRFGAPLGARIEFEDTLWVGLTDTY[illegible]	4	4	3	3	8.1	3.284	4	*	3-ketoacyl-CoA thiolase, mitochondrial-like [Bombus impatiens],XP_003488365.1

18	12	LMI_GLEAN_10021933	[mRNA] locus=scaffold16018:15229:18920:+ [translate_table: standard]	2003	55374	47	MREIVHIQAGQCGNQIGAKFWEIISDEHGIDPTGAYHGDSDLQLERINVYYNEASGGKYVPRAILVDLEPGTMDSVRSGPFGQLFRPDNFVFGQSGAGNNWAKGHYTEGAELVDSVLDVVRKEAESCDCLQGFQLTHSLGGGTGSGMGTLLISKIREEYPDRIMNTYSVVPSPKVSDTVVEPYNATLSVHQLVENTDETYVIDNEALYDICFRTLKLATPTYGDLNHLVSLTMSGVTTCLRFPGQLNSDLRKLAVNLVPFPRLHFFMPGFAPLTSRGSQQYRALTVPELTQQMFDAKNMMAACDPRHGRYLTVAAMFRGRMSMKEVDEQMLNIQNKNSSYFVEWIPNNVKTAVCDIPPRGLKMAATFVGNSTAIQELFKRISEQFTAMFRRKAFLHWYTGEGMDEMEFTEAESNMNDLISEYQQYQEATADEDAEFDEEQEGEVEEN	75	13	16	4	47	4.484	13	*	similar to beta1-tubulin [Tribolium castaneum],XP_967267.1

19	349	LMI_GLEAN_10113524	[mRNA] locus=scaffold21257:167871:292928:+ [translate_table: standard]	153	121334	10.3	MANPRILRFAFANESAH TNWKIATMTSVGGSDS YGNLLSGTQVAKEIRES LAKDVANIKNELPHFKP GLAIVQVGGREDSNVYI RMKIKAATEIGIEATHIKL PKSITEVELLNKITKLNN DPNIHGIIVQMPLDSDN PIDSHRVTDAVSPDKDV DGSGVTIAGSNAVVLG RSKIVGTPVSELLKWHH ATVTVCHSKTKNLDKLT ANADILVVAIGRAEMVK GSWIKRGAVVIDCGINAI PDPTKKSNQRLVGDVA FEEAKQIASYITPVPGG VGPMTVAMLMKNTVLS AQKAASKILNVQWNLSI LPLTLKKPVPSDIEIARS QEPKDISDLAREIGLHP AEVEQYGHKKAKITLDV LKRLQHQKDGKYIVVA GITPTPLGEGKSTTTIGL VQALSGHKKKNAFACV RQPSQGPTFGIKGGAA GGGYSQVIPMEEFNLH LTGDIHAVTAANNLLAA QLDARMFHEATQKDDS LYMRLVPRVKGSRAFS EIQLRRLQRLGISKTDP DSLTPEEIRKFVRLDIDP ETVTWTRVIDTNDRFLR [illegible]	10	10	9	9	10.3	2.055	10	*	c-1-tetrahydrofolate synthase, cytoplasmic-like [Megachile rotundata],XP_003700207.1

20	128	LMI_GLEAN_10160841	[mRNA] locus=scaffold4989:131164:145437:- [translate table: standard]	408	31633	29.5	MELYGSRGERTKRRATTRANSCGVTGRRRRHRLKKLLRRCTKKMEYKTRQWHEKCFCCCVCKTPIGTKSFIPREQEIYCATCYEEKFATRCVKCNKIITSGGVTYKNEPWHRECFTCTNCNTSLAGQRFTSRDEKPYCADCFGELFAKRCTACSKPITGIGGTRFISFEDRHWHNDCFICAMCKTSLVGRGFITDADDIICPDCAKQKLM	10	10	5	5	29.5	1.927	9	*	Four and a half LIM domains protein 2 [Acromyrmex echinatior],EGI61543.1

21	208	LMI_GLEAN_10088301	[mRNA] locus=scaffold10516:103543:184315:- [translate_table: standard]	259	51462	24.4	MLPRGARILCNKASQREKALSSFFGSKSNYATKADFETKPFRLHRLDSGPSTQVSVTSEEALQLYKQMHVIRRMESAAGNLYKEKIVRGFCHLYSGQEAVAVGMRAALRPQDSVITAYRAHGWTYLMGVSPAGVLAELTGRKVGCARGKGGSMHMYANNFYGGNGIVGAQVPLGVGIAFANKYKGSDGVCVTLYGDGAANQGQIFEVYNIAKLQNLPCIFVCENNGYGMGTSVERASASTAYYTRGDYVPGLWVDGMDVLATREATRFAIAHCLSGKGPIVIEAATYRYSGHSMSDPGTSYRTREEIQEVRQTRDPITSFKEKIINAELATPEELKKLENEIRAEIDEAVKLAKAEKECNLDELTADIYAASLESSVRGALPWYDLKHKNIGKPINL	8	7	7	6	24.4	1.532	6	*	probable pyruvate dehydrogenase E1 component subunit alpha, mitochondrial-like isoform 1 [Bombus terrestris],XP_003399781.1

22	98	LMI_GLEAN_10118445	[mRNA] locus=scaffold17165:8807:31848:+ [translate_table: standard]	490	106795	9.4	MEKEIVGPFEKNQPGIG QMKTIFILFAVLGITAAK AVPTPVVSVHSRDLQD DLNDFLALIPIDDIVNIVL DHMANDPEVQAAIEYV LSDEFKNIVLAIDALPEYI DFLNYLQESGLDVYKY VNMLHDLLGLPQLTPPS KLRHTRSIRSMVDEILAI LPVDELRALFNEKLETS AEFKELYDRIRSPEFQSI VETLNSLPEYQELLQKL RDAGIDVDAFIDLIRGIF GLPTNKIMKLHVSAKKA NRDLQDDLNDFLALIPV DDIANIVFDHVSNDPEV HAAIEYVLSDDFKNIVLA IDALPEYIDFLNYLQESG LDVYKYVNMLHDLLGLP QLTPPSKLRHTRSIRSM VDEILAILPVDELKALFN EKLETSPEFKELYDRIR SPEFQSIVETLNSLPEY QELLQKLRDAGIDVDAF IDLIRGIFGLPTSKSTKFI HVLVTAKKANRDLQDD LNDFLALIPVDDIANIVF DHVSNDPEVHAAIEYVL SDDFKNIVLAIDALPEYI DFLNYLQESGLDVYKY VNMLHDLLGLPQLTPPS KLRHTRSIRSMVDEILAI [illegible]	14	14	5	5	9.4	2.076	14	*	major allergen Bla g 1.02 [Blattella germanica],AAD13531.1

23	70	LMI_GLEAN_10042129	[mRNA] locus=scaffold9555:69878:87667:+ [translate_table: standard]	629	25461	43.4	VGSFETGNSIVREEQSEVLNPGQPNQQRVVRGRYSYVDPEGRDVVVSYVVGEDGGFRAEGNVLPKEPEIPEAIQIALARNAAEEAQLNEQQRAEVGCVLALATAGSCAPQQPVGPTTPPVPILRSALDTNHDGNYAFSFETGNSIVREEQSEVLNPGQPNQQRVVRGRYSYVDPEGRDVVVSYVVGEDGGFRAEGNVLPKEPEIPEAIQIALARNAAEEAQLNEQQ	22	22	8	8	43.4	3.503	20	*	similar to conserved hypothetical protein [Tribolium castaneum],XP_970222.2

24	907	LMI_GLEAN_10119040	[mRNA] locus=scaffold23000:400254:501928:- [translate_table: standard]	49	67895	4.1	MQEYIDNLLKSRIHVFDKPNDSALQYFDKQWVPVHKRWMGAFKYKSIRTLYSAAALGVDNYSKTRERVQNQFTNMADRFRTKMAEISNQESKNLVFTEDLKNMVHLAGENDVELVLKMLRRFNSQNKDLRFGTFVFGPVVMRMLHYLNKPDEALNAFKDPALDGIFDQLITYQLLLDLLYKNEKYQDMLTVFEMIKDKQIQSARFPKNVVVLTMAACYKLNSEDSFKYATDLWKSLLEVGHQPTRRAATFIAALALNQNAPHIAAEIISCVNTQGYVTIRNIKVAALADLNRLEDALAVLRQAVQVDNPNQVKATFTKDVVEKVKAASEREERKEVAHEAQALLKRLAENRHTVDQLRVDVAASVSFFMPATFCVTVRGSTGTRASRGVALSEPVQGGSGGTEVVTASASGARKCSTKAGDGSEVVVAAEESRDDEVVVAPPASAHEGSTAATKLVWQPSGVQVGKIEVSDSCSTNRAGEQETEEDHSVGEGREMWELATRVEAALAMHLSETLA	2	2	2	2	4.1	1.566	2	*	pentatricopeptide repeat-containing protein 2-like [Acyrthosiphon pisum],XP_001946785.1

25	1338	LMI_GLEAN_10187958	[mRNA] locus=scaffold2981:2252450:2348288:- [translate_table: standard]	25	75038	1.1	SDQNFFGKSQYSNKITGYCKLISKDRFVIDVGYHKRMIEIFKTIGSAKYDAPSKKWSFHIKDHNLLLKGLDAEPDLSISKLPDIILKTFLSAPPTPVNYDIDLSRIDKMLVENLMPFQREGICFGISRKGRCLIADDMGLGKTVQGLGIAHYYYSDWPLLIVTPSSVRYLWLEAVLSWLPTVPLHHVEVMNTGKDYVGDAKVVIISYDLLARHHKDLNLSRFGFVILDESHFLKNIKTARTKAALFVARACRRVVLLSGTPALSRPCELYTQIAAIEHSAFPSFQTYGIRYCAGKQTKWGWDFSGSSNMEELQLLLEHRFMIRRLKSDVITQLPAKMRQVVVLNPSSINMKSEEVENWLQRLHSKQLSGMQRRGALLSFFAATGNVKLKAVKDYIADLLEAGKKFICFVHHKNVMDGIAKTIEEKKAMYIKIDGSTAASERKALCDKFQFDDNYSVALLSITAANAGITLTAAKLVVFAELFWNPGILTQAEDRAHRIGQIDSVLVQYLIAKGTADDHLWPMIQSKLDVLNKAGLSKD	2	2	1	1	1.1	1.509	2	*	conserved hypothetical protein [Ixodes scapularis] ，XP_002416013.1

26	62	LMI_GLEAN_10042900	[mRNA] locus=scaffold1163:41882:52486:- [translate_table: standard]	678	73348	26.6	MALLSFRLASAVAKHLP AATPQVFKSIPNGRTKC LIAYWKTARTVDCVEVR LVIDCTEISGLTWPAAQI TNRNIHVSCSQRAAEIS SILEERILGAAPKADLEE TGRVLSIGDGIARVYGL KNIQADEMVEFSSGLK GMALNLEPDNVGVVVF GNDRLIKEGDIVKRTGAI VDVPVGEELLGRVVDA LGNPIDGKGPLKASKRF RVGIKAPGIIPRISVREP MQTGIKAVDSLVPIGRG QRELIIGDRQTGKTALAI DTIINQKRFNDGEDEKK KLYCIYVAIGQKRSTVA QIVKRLTDSGAIGYSVIV SATASDAAPLQYLAPYS GCAMGEFFRDNGKHAL IIYDDLSKQAVAYRQMS LLLRRPPGREAYPGDV FYLHSRLLERAAKMND AHGGGSLTALPVIETQA GDVSAYIPTNVISITDGQ IFLETELFYKGIRPAINV AGSMKLELAQYREVAA FAQFGSDLDAATQQLL NRGVRLTELLKQGQYV PMAIEEQVAVIYCGVRG HLDKLDPSKITAFEKEFL QHIKTSEAALLANIAKEG [illegible]	26	26	13	13	26.6	2.799	26	*	similar to AGAP005134-PA isoform 1 [Tribolium castaneum] >gb|EFA07428.1| hypothetical pro TcasGA2_TC008728 [Tribolium castaneum]` XP_966334.1`` putative mitochondrial ATP synthase alpha sub precursor [Toxoptera citricida]` AAU84946.1

27	462	LMI_GLEAN_10061914	[mRNA] locus=scaffold27830:15859:128950:+ [translate_table: standard]	114	83548	7.3	MRRSSDEDEFLLLLTVA SKKRRKWVHDINTKRE EYALVLEPGKKYAQTVS KPFHISMASLDVVHSEN ELVTVNVNFQNAEFILC NLQKNKILQTSLDLNFE AGDRIAFYTSGKGRVHL TGYLIDDDDVDDDLDID AEAEESEEDVTPQKNAI KANKQEKRKSVGQTPG KPVLKKSKMDSEDEDG DDNGDDFDDDGDSDLE SLGDSDEEMEVESEED DGEEDEDSELEETPPQ KHQQGKKKEKQSAQK QLQDKQTNTPNEMKKK KNKGHDAATPNTPTVQ ANGTPETQSGKKNKKK GGETPGDKGANTPKPP QAGSPQTPQKKLLEGG VAVEDTVVGSGPVAKP GRFVTVYYTGRLKQNN KKFDETVQGPGFKFRL GKGEVIKGWDIGVTGM KVGGKRKLIIPPHMADG KIFNRDGLAGQYFRAVL LIVSQRKKKVKKVAQIS EQDSRYGAKGSPPVIP PNSALVFEVELKNDPKP ACRRASGSSTITDATSG KPSQPLTPKGQREGQK KPKEEEDANEVSSVPR [illegible]	4	4	3	3	7.3	2.155	4	*	46 kDa FK506-binding nuclear protein, putative [Pediculus humanus corporis] >gb|EEB13519.1| 46 kDa FK506 [illegible] nuclear protein, putative [Pediculus humanus corporis]` XP_002426257.1 `` hypothetical protein LOC100 [illegible] [Acyrthosiphon pisum]` XP_001951061.1

28	409	LMI_GLEAN_10110738	[mRNA] locus=scaffold50570:566297:570020:+ [translate_table: standard]	131	71286	10.6	MASFPGTLAFDEFGRP FIIIRDQQNQQRLTGVE ALKSHMLAGKTIANILRT SLGPKGLDKMMVSADG DVTVTNDGATILKQMDV DHEIAKLMVQLSQSQD DEIGDGTTGVVVLAGAL LEQAEQLLDKGIHPIRIA DGFELAAQHAVKHLDSI ADSFPFDINNLEPLIQTA MTTLGSKIVNKCHRQM AEIAVNAVLAVADMEKK DVNFELIKVIGKVGGRL EDTMLVKGVVVDKDFS HPQMPKVLKDVKLAILT CPFEPPKPKTKHKLDVT SVEDYRALRAYEAEKFT EMVQQVKDTGATLAIC QWGFDDEANHLLLQRE LPAVRWVGGPEIELIAIA TGGRIVPRFEELTPDKL GHAGLVREISFGTTKDR MLVIEECKNSRAVTIFIR GGNKMIIEEAKRSIHDA LCVVRNLVVDNRIVYGG GAAEISCALSVSTEADK YSSLEQYAFRAFAEALE SVPLALAENSGLSPIHT VTEVKARQAVEGNSAL GIDCMLNGTADMRQQH VIETLRSKKQQIVLATQL VKMILKIDDIRCPNDQG [illegible]	9	9	6	6	10.6	1.543	9	*	heat shock protein 60 [Schistocerca gregaria]ˆ AEV89752.1¨ chaperonin containing TCP1, subunit 5 (epsilon [Acyrthosiphon pisumˆ NP_001153860.1

29	903	LMI_GLEAN_10187224	[mRNA] locus=scaffold160:960582:1058210:- [translate_table: standard]	50	40513	2.2	DNSGVLAAVVKGAKQAIVECQYQFRNRRWNCSTRNFVRGKNLFGKIVDRGCRETAFIYAITSAGVTHAIARACSEGSVSACSCDHSYAHRASAKHPLPGAAAAAAADWEWGGCSDNIDYGFKFSRDFVDTGERGRSLREKMNLHNNEAGRAHVVAEMRRECKCHGMSGSCTVRTCWMRLPSFRVVGDNLKDRFDGASRVMLSNAAAPGGGKRXLQLRPYNPDHKPPGVKDLVYLEPSPGFCERNPHLGIQGTHGRQCNDTSIGVDGCDLMCCGRGYRTQEITVVERCACTFHWCCEVKCKLCRTVKTIHTCL	2	2	1	1	2.2	2.021	2	*	wingless protein [Culex quinquefasciatus] >gb|EDS27053.1| wingless protein [Culex quinquefasciatus]^XP_001847858.1^protein Wnt-1-like [Bombus terrestris]^ XP_003393164.1

30	245	LMI_GLEAN_10065877	[mRNA] locus=scaffold31298:55238:79387:+ [translate table: standard]	214	20430	13.2	MASNKRKLSDVKIEQLANITELVWNEFMDEFSGGESHHFHVSTQVRIQMQTTVIEEEEQMYRLPRVLRSNALQQIYQVRSFAKDVRFGPEVRALMLQGVDILADAVAVTMGPKKAYTRNYATEFDIQGVPTILSTQNISGTITAIGKRLAPVSMYGWDP	2	2	1	1	13.2	3.368	2	*	heat shock protein 60 [Pteromalus puparum] , ACO57619.1
31	327	LMI_GLEAN_10025235	[mRNA] locus=scaffold65233:15826:30841:- [translate table: standard]	163	22781	16.7	MGLKVGDKLPSVELYENNPTNKVNLEKLLSNKKAVVFAVPGAFTPGCSKTHLPGYVEKADELKKKGIDEIICVSVNDPFVMDAWGKEHKATGKVRMLADPDASFTKALELETNLPPLGGTRSKRYSMIIENGVVKSLNVEPDGTGLSCSLADKLPL	5	5	2	2	16.7	3.633	4	*	hypothetical protein AND_16974 [Anopheles darlingi] , EFR21501.1", peroxiredoxin-like protein [Phlebotomus papatasi]", ABV44727.1

32	94	LMI_GLEAN_10105042	[mRNA] locus=scaffold3524:1744:126661:- [translate_table: standard]	502	30368	45.8	CLQVAVVCLAAAAMALPQRPVAGGRGKDAVIVSATNDVNFDGSYRYRFESADGTIAEQEGTLVKSQDPNEPDTIAVRGSVSYTAPDGTPIKLTYTADKEGFKPEGDHIPVAPPVPEAIARALQYIAEHPLVLSALVAVTMGAVAEIPKEVVPIVKQESEISPEGNFRYSFESGDGTRAEQEGTLVKSQDPKEPDTIAVRGTVTYTAPDGTPIQLTYTADKEGFKPEGDHIPVAPPVPEAIARALQYIAEHP	29	22	10	7	45.8	3.003	22	*	RecName: Full=Endocuticle structural glycoprotein SgAbd-3’ Q7M4E9.1” endocuticle structural glycoprotein SgAbd-1-like [Apis florea]’ XP_003697135.1

Full=Putative beta-carotene-binding protein ^ P82886.1 ^^ GK14019 [Drosophila willistoni] >gb|EDW84222.1| GK14019 [Drosophila willistoni] ^ XP_002073236.1
*
2
1.748
8.1
1
2
2
3
MDRRLLLAFAALAAHAA AYIGIPQIGADPIDPLAQ LPPLTWNSNSRGLDLQ FRLDNARHTGLGRSVV DNLQVDTVNKRIRVVTH VDGLNNIEGDYTLSGKV LGVPLDGSGIFKLSMYG SSVDVTYAGHIENGKQ GHSYLKIDSVSVKLQLG QSYYELTGLFGDYEPLV HAGNMFINSVAGKVVE PEMLPTLEKWLAQGYL QHAQAVFSTVPYDQLF PKTSAYTGFYRAVFPQ YF
8.1
28719
76
[mRNA] locus=scaffold1779:765435:779043:+ [translate_table: standard]
LMI_GLEAN_10172624
650
33

34	629	LMI_GLEAN_10164915	[mRNA] locus=scaffold223:395995:521356:- [translate_table: standard]	79	29066	4.9	MVYLQHLLLHVAMSLPRERRDADETTEGAANILEGIERLASDISKKTQEFFTADSFRAIGANATELANKCTLVTMLLLRAMFQVCCGAPLSPEEALRVLDVERRLLLEASGLLVQWRRSAADRPALAQRLSGLGERRDXXXXXXXXXXXXXXXXXXXXXXXXXXXXXXXXXXXXXXXXXXXXXXXXXXXXXXXXXXXXXXXATGEVADRLRQEAPSAEELLRLADEFRRIWRRFRRLDAHADASGQQ	4	4	2	2	4.9	5.574	3	*	predicted protein [Micromonas pusilla CCMP1545] >gb|EEH59002.1| predicted protein [Micromonas pusilla CCMP1545]ˆ XP_003057357.1

35	394	LMI_GLEAN_10131973	[mRNA] locus=scaffold431:622673:665285:- [translate_table: standard]	135	34295	6.4	MANVIEQTSVHVFGELL DMPNVIELENGPHAPY YRALNLFAYGTYRQYLK NKAQLLDLTPMQKKKL QHLTIVTLATKSKCIPYS VLLQELDMKNVRDLED LIIEAIYADIIHGKLDLKN SQLEVDYAIGRDIQPAD LGSIISTLQEWCDSCET VLSCVEMQINRANSEK NRRLKHKEAIEQEIHNIK KTLKTQSQDTDEAMAT DTRDTAAQTGDKGKKP FKGKGIRSSSGKFWQK S	2	2	1	1	6.4	1.683	2	*	COP9 signalosome complex subunit 7A, putative [Pediculus humanus corporis] >gb|EEB19483.1| COP9 signalosome complex subunit 7A, putative [Pediculus humanus corporis]ˆ XP_002432221.1
36	57	LMI_gi_85816368	[mRNA] locus=scaffold1153:2754721:2862495:+ [translate_table: standard]	747	25531	56	MADGVKRNIPIKLGDFS VIDTEFSSIRERFDAEM RKMEDEMARFRSELMN RESNFFTKSTTSTTSST DVASSPRTWLDGFNSP LIQDEGQSKVLKLRFDV SQYQPEEIVVKTVDNKL LVHAKHEEKTESKSVY REYNREFMLPKGTNPE SIKSSLSKDGVLTVEAP LPALAEGEKLIPIAQH	23	23	9	9	56	6.205	21	*	heat shock protein 20.6 [Locusta migratoria] ˆ ABC84493.1 ˆˆ heat shock protein beta-1-like isoform 4 [Bombus impatiens] ˆ XP_003491574.1

37	158	LMI_GLEAN_10107594	locus=scaffold14021:341551:347710:+ [translate table:	334	14942	33.1	MVAVSAVVARPQAPGGSNTSPIPIISFVNEGVNFDGSYKWSYETGNEIKAQEEGFLKNAGNPETEGQAAQGSYSYTAPDGTRIEVTYTADENGFVPQGAHLPTPPPIPPAIQRALEFIASQPPQPEDNSS	5	5	3	3	33.1	4.452	5	*	motif 45 precursor [Bombyx mori] >dbj|BAB32485.1| cuticle protein [Bombyx mori] >tpd|FAA00548.1| TPA: putative cuticle protein [Bombyx mori]`

38	337	LMI_GLEAN_10126245	[mRNA] locus=scaffold8098:18809:32633:- [translate_table: standard]	156	90548	12	MRTSTLAVLSLLVALAA PVVPQKYADKELLVKQ QKIYNLLYLLDRPIFDEE QKSIVQSYKLEENADKF VKPEVVEKFLKYYKYGY FKNQGKAFSPFYKIDMY QVIDMFEVLFYAKDFDT FYKTAVWMRQNLNVG QFVYAFTVAVLHREDTK NVTLPPPYEIYPELFVK SEAIQKAYDARFQGLA GTKEQPYVIVSNYTGYP EPYNPDDMLSYFTEDV GLNSFFAYLHYRYPFW MKYDFPRKGEMFYFTL RQLLARYYLERLSHKLP DVTPVDYYKPVQVGYY PEIRLQNGLETPVRAEG VIPRDIDFLKVEEIENYE RRIRSAIDFGFFFNEKA ELYSVRDKDATDIIGNIV EGSATSIHKNYYGSFYR GLISIFGHISDPKHQYGV VPSVLEKPETMLRDPLY YKIAKRVLSVFDHYKNQ LAPYTQQELVMPGVKV DSVTFDKLVTFFDEFNI DVSNVVGEAYDKGYVV VRQPRLNHKPFNYHIKV TSDKEVDAVVRVFYGP RFDAYGREYSLEESKN YYVLMDIFPYKLQAGEN [illegible]	10	9	8	7	12	4.818	9	*	arylphorin hexamerin-like protein 2 [Romalea microptera]` AAX14951.1

39	59	LMI_GLEAN_10095175	[mRNA] locus=scaffold17982:244370:282467:+ [translate_table: standard]	722	59490	19.6	STSHRVERDTFGELQVPGDRYYGAQTLRSVMNFPIGGETERMPKPVIVAMGILKKAAALVNKEYGLDPKIADAIAKAADEVISGELYDKHFPLVIWQTGSGTQSNMNTNEVISNRAIEILGGVLGSKNPVHPNDHVNKSQSSNDTFPTAMHIAVATEINKSLIPALKLLHDALDDKAKAFEDIIKIGRTHLMDAVPLTLGQEFSGYVTQMANSIERVKTALPRLYELALGGTAVGTGLNTRIGFAEKCAAKISELTGLPFVSAPNKFEALAAHDALVEVSGALNVVACSIMKIANDIRFLASGPRCGLGEISLPENEPGSSIMPGKVNPTQCEAITMVAAQVMGNHVAVTVGGSNGHFELNVFKPMMVSNVLRSVRLLADSSRAFTNNCVVGIKANQERIAKLMNESLMLVTALNPHIGYDKAAVIAKTAHKEGTTLKEAALKLGFLTEADFESWVKPQDMLGPK	21	21	8	8	19.6	1.8	21	*	aspartate ammonia lyase [Aedes aegypti] >gb|EAT40064.1| aspartate " XP_001658987.1" fumarase [Glossina morsitans morsitans]" ADD20350.1

40	123	LMI_GLEAN_10154080	[mRNA] locus=scaffold1255:83959:94554:+ [translate_table: standard]	417	88866	21	MRTATVVVLSLLAALAA<br>ATAVPPSEADKELLEKQ<br>NKIIRLFYQVQQPTIIPE<br>EQEIAKSYKPIENIDNYQ<br>YKDKVEVFWKYYTEYG<br>FVPRDEVFSIYYKKHFS<br>QAKGLFELFYYAKDFDT<br>FYKTAVWAREYLNPGL<br>FVYSFTVAVLHREDTKF<br>VTLPAPYEVYPQLFVNA<br>EVIQKAYDARLRDVVST<br>RKEPYVFYANYSGFPV<br>ANNPEELVSYFTEDVGL<br>NSFFAYLHYKSPFWLN<br>PANYSLPAFKRRGDSF<br>FFILQQLLARYYLERLS<br>NRLPDVKPVDYANPVL<br>VGYYPELRLQNGIEAPA<br>RPEGVYPSNFDLLFVE<br>RIQNYERRIRDAVDFGY<br>LYGYDFKTFNLNEKDLT<br>DILGNVIEGNAESVNYE<br>FYGSIYRYLISLFGHIAD<br>PYHKYGAPASVLEQPE<br>TQLRDPLFYRIAKRVISI<br>FYQYKNQLKPYTKNQL<br>EFPGVAIEGITFDKLVTF<br>FDDFDIELNNALSFSKP<br>EQGDNFNFIARQYRLN<br>HKPFYYQLKVKSEKEV<br>DAVVRVFVGPKYDVYG<br>REFTLDEKKQYYFLLDV<br>[illegible]	19	17	12	10	21	4.09	16	*	hexamerin-like protein 2 [Locusta migratoria]` ACU78069.1

41	749	LMI_GLEAN_10175054	[illegible] 51632:115 1829:+	63	8977	32.3	MDKPVVLARVMKVLGR TGSQGQCTQVKVEFIG EQNRQIIRNVKGPVREG DILTLLESEREARRLR	2	2	2	2	32.3	5.316	2	*	protein S28e [Mycetophagus quadripustulatus] >gb|ACY71303
42	587	LMI_GLEAN_10073596	locus=scaffold699:80 480:89313:+ [translate table:	85	17757	22.5	QDKYPDTFDKLDLQEL QGDKERVQAAIKCLVQ EEDTECKPAAKLLKSVL AEIVQTDCGKCTEAQKT KVAGFFAFVSQNYPQQ MQQVLEKYDPSKEYRE KYAQSWAAKGIKV	3	3	2	2	22.5	1.56	3	*	hypothetical protein [Locusta migratoria]' CAJ01469.1

43	270	LMI_GLEAN_10154525	[mRNA] locus=scaffold5199:868974:909019:- [translate_table: standard]	195	82039	10.9	MKETYVKPNVAELQDIA<br>NKLRVHSIEATNTAKSG<br>HPTSCASMAEIMSVLFF<br>NTMRYKISEPRDPSSD<br>RFILSKGHAAPILYAAW<br>AEAGLFPVKDLQNLRKI<br>DSDLEGHPTPRLNFIDV<br>GTGSLGQGLSIACGMA<br>YVGKNYDKASYRVYCL<br>VGDGESAEGSVWEALN<br>FASYYKLDNLCVIFDVN<br>RLGQSDPTVLQHNMET<br>YRKRLDAFGLNAIVVDG<br>HDVEELSKAFHEAATTK<br>GKPTAIVAKTYKGKNFP<br>TIEDQENWHGKPLGGQ<br>ADTVLEHLKSLIKNKGP<br>LQLHPQKPLKEDAPIVDI<br>TNVKLSSPPNYKIGESV<br>ATRLAYGTALAKIAASN<br>SRVIALDGDTKNSTYSD<br>KLRKVYPDRYIECFIAE<br>QNLVGIAIGAACRDRTV<br>AFVSTFATFFTRAFDQI<br>RMGAISQTNVNFVGSH<br>CGVSIGEDGPSQMGLE<br>DIAMFRTIPGSTVFYPS<br>DAVSCERAVELAANTK<br>GVCFIRTSRPATSILYKN<br>EENFQVGKAHIVKQSN<br>SDKVLIIGAGVTLYEALK<br>AADELAKSGIHVRVLDP<br>[illegible]	7	7	5	5	10.9	1.606	6	*	transketolase-like protein 2-like isoform 1 [Bombus impatiens]ˆ XP_003493512.1

44	141	LMI_GLEAN_10095481	[mRNA] locus=scaffold3133:30163:79562:- [translate table: standard]	376	24451	67.1	MRPGIEPEPPCILGRRA NFAFSVYDFDGSGTVD AIYLGDLLRALNLNPTLA IVEKMGGTKKKNEKKLK IEDFLPIYSQVKKEKEV GCYEDFLECLKLYDKAE DGKMLAAELSHTLLSLG ERLTDDECESILKECLD PEDEDGFVPYAPFLKRL IAGPPEEKPAEEAK	15	15	8	8	67.1	1.984	14	*	myosin 1 light chain [Lonomia obliqua] >gb|AAV91412.1| myosin 2 light chain [Lonomia obliqua]^ AAV91411.1

45	838	LMI_GLEAN_10181062	[mRNA] locus=scaffold1755:2066428:2107122:+ [translate_table: standard]	56	50189	10	MTSDVQMFVYLTALRGRQSLNLQTTYELANTKDGTNIHARGDIRTPDRSGRGDPTCLVAICDSQRRPPSYSLDDMPETSFDCRDKILGGYYADPETDCQMFHVCVKVPGVGVQNYRFLCPNDTAFDQESQICASWYDVDCEAALLYADNFDLYRIGYVPSSPAIVASKATALPLGPSTAAPARARQPFNSRQPQLDDEYYLQAGDTADRRLQPKDVLRGSSSGNFFNQKKGREEEEEAVISKKKVAVRKFRKRPSQQAEDNGEAASTPAPTTYFSTAGAGSSRSTSGNTYXRRPPLPPPPRRSSRRAARASSRGPPPPPPQPPPAQPSATRTSPGTTTTPSPTTTTTNRASSSRITTTTCRPRHRRGLTTTTTRRKRDPTTTHPRRRLPRPLTSTATKPCRGTATASPPLL	4	4	3	3	10	1.546	4	*	hypothetical protein LOC100169018 [Acyrthosiphon pisum]` XP_001946070.2

46	1380	LMI_GLEAN_10080939	locus=scaffold24831:46155: 74657:+ [translate_table:	20	21051	4	MAAIAQGLRCVFARNG FWQKENVRHFADAKPT MDQIRERVLKVCAAYD KVTADKITPKRFYSDKE PLTYELIRDRVLLVLRLY DKVDPDKLTLESHFIND LGLDSLDHVEVIMAMED EFGFEIPDSDAEKLVRP ADIVRYIADKEDVYD	2	2	1	1	4	2.428	2	*	willistoni] >gb|EDW79295.1| GK13357 [Drosophila , XP_002068309.1; mitochondrial acyl carrier protein 1 isoform A NP_001164229 [Tribolium
47	101	LMI_GLEAN_10050722	[mRNA] locus=scaffold28250:33611:59197:+ [translate table: standard]	481	27284	27.9	MTRSFRNSAHGFRSSA VLPSRGATGADLLTSVC DEVLQRPPSLVPSSGR NGYFQFFIACVSRPCNS TPYFEMSLTTQLSLQLV LSALVAVAVGAPADVPK EVVPIVKQESDISPEGG FRYSFESADGTIAEQEG TLVKSQDPNEPDTIAVR GSVSYTAPDGTPIKLTY TADKEGFKPEGDHIPVA PPNTAADASVTNIVRLP SDKPLFVEPFSSATVAL VLGVSVMRPNITYA	15	8	5	2	27.9	5.555	8	*	[Drosophila simulans] >gb|AAL29063.1| LD46766p [Drosophila melanogaster] >gb|AAF58518.2| cuticular protein 49Ae [Drosophila melanogaster] >gb|EDW47559.1| GM21380 [Drosophila sechellia] >gb|EDX06761.1| GD10877 [Drosophila

48	808	LMI_GLEAN_10061897	[mRNA] locus=scaffold14256:69811:121428:+ [translate_table: standard]	58	41762	12.1	MTPSMISECSKVIEEAIC NAILDLLAEKRIVCKDEL ITIDEKDELQEYTACDS DALSSSPIEKRVEDGEK HFDKWKHIDEEMYDRFI RARRNNEQVHLVKYAL YVMSEENWFIRTVSFCT CTEYIFASLPRTQRGQP IVLGGDPKGKNFLYTNG NSVIIRNIENPAISDIYTE HSCTTNVAKYSPSGFYI ASGDQSGKIRIWDTVNK EHLLKNEFQPFGGPIKD ISWSPDNQRIVVVGEG RERFGHVFMAETGTSV GEISGQSKPINSCDFKP SRPFRIITGSEDNTIGVF EGPPFKFKMTKQSE	3	3	3	3	12.1	2.111	3	*	actin-interacting protein 1-like isoform 1 [Acyrthosiphon pisum]ˆ XP_001943831.2¨
49	423	LMI_GLEAN_ 10137288	[illegible] 26256:921364 :937729:- [illegible]	125	11812	19.3	MADLPPPVRVSPLIKFG RWSFLLAGILYGASRNR TLSKREAALREIEEKER PIREAKLKEEKAMLNKI QMAELARAAGVEPEP	5	5	2	2	19.3	1.713	3	*	[Bombyx mori] >gb|ABF 51335.1| H+ transporting ATP synthase subunit e

50	825	LMI_GLEAN_10135691	[mRNA] locus=scaffold4224:7922:173493:+ [translate_table: standard]	57	86414	3.9	MTKEGLSSGDPNSYSN<br>PDEVAVTHLDVSLNVDF<br>EHKVLIGEVNLTIEKKKD<br>DAHTLVLDAREIKIKEIS<br>DKESGTKLDYNIEKDDP<br>VFGSKLTVKLPSKLGKN<br>GIVKIEYETSPSASALQ<br>WLSPQQTAGGKHPYLF<br>SQCQAIHCRSVIPCQDS<br>PAVKVTYSAQIRAPEDL<br>VVLMSAVREGEPSLVG<br>NGLKLHKFHQKVPIPSY<br>LFAIAVGALESHRLSPR<br>CQVWSEKEFVDQAAYE<br>FAETEKMLSIAEDICGE<br>YVWGVYDLLVLPPSFP<br>FGGMENPCLTFVTPTVL<br>AGDRSLADVVAHEISHS<br>WTGNLVTNKNFEHFWL<br>NEGFTMFVERKILARLIH<br>PDQRKFSAFCGLKDLR<br>DAINTRGPNDPMTCLVP<br>DLRSVSPDDSFSVVPY<br>EKGHTFLYYLEELAGGP<br>EKFDPFLRAYLQKFKYK<br>SIGTDDFKEYLYEYFPQ<br>NENLKKVDWDTWLKKP<br>GMPPVIPDYKTSLEETC<br>TDLCKKWVKWAECESC<br>PFSATDLKNLSSLQLKE<br>FLAQLLEEKPLSLEKLK<br>TMERIYKLNSVKNSEIR<br>[illegible]	3	3	2	2	3.9	1.538	3	*	putative leukotriene A4 hydrolase [Daphnia pulex]` EFX86132.1

51	597	LMI_GLEAN_10074080	[mRNA] locus=scaffold9349:34205:115668:- [translate_table: standard]	83	51512	7.2	KKAKQAEIDRKRAEVRKRLEEASKAKKAKKGFMTPERKKKLRLLLRKKAAEELKKEQERKAAERRRIIEERCGKPKNVDEANEATLKSIINTYHKRIASLEGEKFDLEYEVAKKRAELKAIVKEYYDRVYLCEGQKWDLEREIRNKDYEIADLNSQVNDLRGKFMKPTLKKVSKYENKFAKLQKKAAEFNFRNQLKVVKKKEFTLEEEDKEKKPDWSKKGDKQKDVAAEGGAPPAEGAAPPAEGAPPAEGAAPPAEGAPPAEGAAPPAEGAPPAEGAAPPAEGAPPAEGAAPPAEGAPPAEGAGPPAEGXGAAPPAEGVAPPAEGAAPAAEGAAP	4	4	3	3	7.2	1.513	4	*	Troponin I [Camponotus floridanus]` EFN61242.1

52	709	LMI_GLEAN_10102266	[mRNA] locus=scaffold32375:54578:85240:+ [translate_table: standard]	68	48097	2.7	MASTGVVEEHIGEKPNQMQPIISKIEGSVLDSFSKLTSSVEKQIINGAIYILNYLHLKEAEIEEETCLETTYTLNRFVRGLASPRLDSRAGFFSILVGFLSTFSSIKTDDVFAAIEKELQSTGSKTKWEIGDIYSGRVLAYGSVIESGLFLKGTDEQQQQIVKKLLEAAELNHFQSIIGSFGPEEPIHSMQKQLALVEHLGPWHHGIYVPLEPYRDQYVPNQNSSDGAIVYQSSRVQLPYRTTPLVLPGAKVKKDSPVATQSYLRHHPNPHFRAPPAPSATEVLMKQKVADTVIQKVVGEEAGAGKQVVHKQFNSPIGLYSDQNIAETIQKQTGVTPKSIVRAPTLDRRNQVPVRQVGNGIFFSPGCR	3	3	1	1	2.7	3.242	3	*	AGAP006260-PD [Anopheles gambiae str. PEST] >gb|EDO63843.1| AGAP006260-PD [Anopheles gambiae str. PEST]^ XP_001688837.1

53	431	LMI_GLEAN_10099907	[mRNA] locus=scaffold14288:99559:167942:+ [translate_table: standard]	123	91829	9.1	MYLETARTEYIKSLHSQI NSSSGTELPGEDPCGV VSMSDNMGPYSRIHSII QMPDSVLGYVVALQVA TNVTEQKHHVSRDKRG QILGNLRGFRGCTVWF TGLSGAGKTSISFELEA YLISRGIPSYGLDGDNV RTGLNKDLGFSKEDRE ENIRRVAEVAKLFADSG VVALCSFVSPFAADRE MARKIHKESDLPFFEVF VDTPLNVCEQRDVKGL YKKARQGSIKGFTGVD QAYEKPENPDLVVKTV NASVEESTMQVVEFLQ EHDIIPQTLQASDEVHE LFVPENRLAAAKEEAEH LPALEITTLDLQWLQVL SEGWASPLEGFMHED QFLQAIHFNCLPEKAGI NQSVVIVLPVHTEDKER LDGMSAIALSYEGRRAI HFNCLPEKAGINQSVVI VLPVHTEDKERLDGMS AIALSYEGRRVAILRKPE FYGHRKEERVCRQFGT SHRGHPYIKMIYESGD WLVGGDLEVLERIRWN DGLDSYRLTPLELRAKF REMGADAVFAFQLRNPI HNGHALLMQDTKRRLL [illegible]	5	5	5	5	9.1	1.773	4	*	adenylsulfate kinase, putative [Pediculus humanus corporis] >gb|EEB20057.1| adenylsulfate kinase, puta XP_002432795.1" bifunctional 3'-phosphoadenosine 5'-phosphosulfate synthase-like [Acyrthosiphon pisum]" XP

54	53	LMI_GLEAN_10190806	[mRNA] locus=scaffold3326:2088159:2135572:+ [translate_table: standard]	806	62104	27.3	MSVRINYVSKTHIGTEK NLPANDNLTQLFSVFAY RFAFFTARRQLPIAFFIIF IGSWWSHVEMGPPDAI LGVTEAYKRDTNPKKIN LGVGAYRDDNGKPFVL PSVREAEEKIRSKNMD KEYAPIAGSAEYCKLSIL LALGDDSEVVKNGCNA TVQGISGTGSLRIGAAF LEKFFPGNKEVYLPTPS WGNHTPIFKHAGLNVK QYRYYDPKTCGFDFQG AMQDIAKIPEKSIILLHA CAHNPTGVDPKPEQWK EISNVVKQKKLFPFFDM AYQGFASGDVARDAFA VRYFIQEGHQMALAQS YAKNMGLYGERAGAFS LITGSKQEADTTMSQLK ILVRPMYSNPPINGARIV TEILSDPTLKAKWLQDV KGMADRIISVRTQLRDN LKKEGSSRDWSHITDQI GMFCFTGMNPQQSERL TKEFSIYLTKDGRISMA GVTSKNVEYLAHGIHQV TK	37	37	11	11	27.3	3.434	37	*	aspartate aminotransferase, putative [Pediculus humanus corporis] >gb|EEB15916.1| aspartate aminotransferase, putative [Pediculus humanus corporis]^ XP_002428654.1

55	118	LMI_GLEAN_10043969	[mRNA] locus=scaffold13590:6421:18700:+ [translate_table: standard]	427	22226	43.6	AYRTNEGKPWVLPVVRKTEKQMAEDETSNHEYLPVLGMESFTSAATKMLLGEDSPAIVQGRAIGVQTLSGTGALRVGAEFLNRHLNYTTFYYSKPTWENHRLIFTNAGFTDPREYRYWDPNKRAIDFEGLIADLQEAPENSVIILHACAHNPTGSDPTQEQWEKIAAVMKVR	12	12	5	5	43.6	1.542	12	*	aspartate aminotransferase, partial [Allonemobius socius]ˆ AEX97005.1
56	395	LMI_GLEAN_10078839	[mRNA] locus=scaffold757:91846:95948:- [translate_table:	135	19454	36	MKSCALALLLVGLVAAAAAYTTKYDNIDLDEILHNDRLLNKYHECLLSDTDTPCTADGKELKAAIPDALTNECAKCNEKQKNGAEKVIRFLIKEKPDLWTPLEKKYDPNGTYRQKYGEELKKVSS	5	3	3	2	36	1.649	3	*	chemosensory protein [Locusta migratoria] >emb|CAJ01464.1| hypothetical protein [Locusta migratoria] ˆ CAB65179.1

57	209	LMI_GLEAN_10071089	[mRNA] locus=scaffold30987:44059:91161:- [translate_table: standard]	258	36174	22.2	MSFQVAEEKTDAGSTAETTADAAAPQADSSSVDAKSEVPSSPEAKEPEIPAPSTEASAQEPSATENTSEVKAEKEEPAKVEEVAPAVPSSPPPAEEQKSPQPSAEEAKVEPLAVEVPAPVVAAAAPVESVESPKETAPPVEPSLPAEPASPAAASQSLTDATETPQTPAEAVASPVSVAQPESAESPAPEANVSSEVPDVPAAVEPQTVVPPVPTTPEPQSAAQEEKVENSPAPEVPVAAPTTPTPAADTSITDTVVQSPENVTVVAVDSAAVPAETDSAVTSADSGTETQLILGPDCRSTIHMTPEERNSCEGVSQVCSLSQC	8	8	4	4	22.2	1.513	8	*	**hexamerin 4 precursor [Tribolium castaneum] NP_001164245**

58	86	LMI_GLEAN_10154078	[mRNA] locus=scaffold1255:5425:17038:+ [translate_table: standard]	534	96494	21.7	MSRGRASPTAGADSRL ISHLSHYKRPSGRRTIR TSSASNMRTATVVVLSL LAALAAAAVVPHSEAGK ELLEKQDKLLRLLYHVQ QTTLVKEEQEIAKTYKPI EHVDNYQYKDKVELFW KYYVDVGFLPKGEVFS VFYQKHFYQARALFELF YFAKDFETFYKTAVWA REHLNEALFVYSYTVAV LHREDTKDVTLPAPYEV YPQLFVNAEVIQQAYDA YLRGEVGTKEAPYVFY SNYSGYPVASNPEELV SYFKDVGLNSYFAYLSY KYPYWLNPKNYSLPEY KYRGESFFFVLQQLLAR YYLERLSNHLPDVKAID YNHPVLVGYYPELRLQ NGREAPARPEGIFARN VDILYVEEIKNYERRIRD GIDYGYLAGYNYEKYN VREKDYTNVLGNILEGN DESINKEFYGAFFRNLIS LFGHIVDPVHRYGVPAS VLEQPETQLRDPLFYSI AKRVLSIFYHYKNLLKP YTYEDLYLPGVTVDDIT FDKLVTYFDNFDFEINN ALTISKPEEGAEFSYVA RQYRLNHKPFFYHLKV [illegible]	23	7	13	3	21.7	9.961	5	*	hexamerin-like protein 2 [Locusta migratoria]ˆ ACU78069.1

59	93	LMI_gi_484000	[mRNA] locus=scaffold223:120163:137211:- [translate table: standard]	504	21909	38.3	MNTLLAVLMLAVAAQARPDAAGQVNIAEAVQQLNHTIVNAAHELHETLGLPTPDEALNLLTEQANAFKTKIAEVTTSLKQEAEKHQGSVAEQLNAFARNLNNSIHDAATSLNLQDQLNSLQSALTNVGHQWQDIATKTQASAQEAWAPVQSALQEAAEKTKQAAANLQNSIQSAVQKPAN	19	19	5	5	38.3	4.229	19	*	choline transporter-like protein 4 [Xenopus laevis].NP_001086000

60	1325	LMI_GLEAN_10057778	[mRNA] locus=scaffold3937:38480:141927:+ [translate_table: standard]	25	99871	0.7	GEKFQHDPDFRGPLHH RSCTDIICLLLWIVFIAA WIAIGCVAFSNGDPNKL LHPKDSSGLRCGLDEG VKDKPYLFYFDLTKCVL GSSLLSPSGCPTKKVC VSECPKKYFRFNGLKR YDETINDLYCTHGTKEK LLSMESIKNAVNNGECV DWYVPSQRDLQSQLH CKDLWVWSSEWVGLT EPNKLHVVKPSTIAWKL SLRASCRELGCPLLAPD WSYLADAWPPSTLRGP SSVSLRYTSHSCHELAA QQKNFNIPDTLTLVLAD NTSMVDTILNFILRTKSD NFIYFQIGETILANLEKN WKWIAGFMGVTEVVCII YIILMQCLAGIMVWLSLL AAVGLNAYCVYACYMK YDELRNTSPEESSSAIL ANQNTWLGLLIISSAVLL IVLLMLLFLRKRINLAIGL IREASRAVASLKSTLAF PVLSWILQCIVIAWAIVV TFYLLSCGDKTYNCNG GCDPSNSQSNCGNCTE EPFKYAEYVTYFHVVNI FGFLWGMCFVSGFSEM ILAGAFATWYWTFKKD NVPFFASLWSIGRTVRY [illegible]	2	2	1	1	0.7	8.503	2	*	hypothetical protein TcasGA2_TC001323 [Tribolium castaneum]^ EEZ98759.1

61	23	LMI_GLEAN_10056004	[mRNA] locus=scaffold77503:52378:54035:- [translate_table: standard]	1503	48232	53.2	MCDEEVAALVVDNGSG MCKAGFAGDDAPRAVF PSIVGRPRHQGVMVGM GQKDSYVGDEAQSKR GILTLKYPIEHGIVTNWD DMEKIWHHTFYNELRV APEEHPVLLTEAPLNPK ANREKMTQIMFETFNTP AMYVAIQAVLSLYASGR TTGIVLDSGDGVSHTVP IYEGYALPHAILRLDLAG RDLTDYLMKILTERGYS FTTTAEREIVRDIKEKLC YVALDFEQEMATAASS SSLEKSYELPDGQVITI GNERFRCPEALFQPSF LGMEANGIHETTYNSIM KCDVDIRKDLYANTVLS GGTTMYPGIADRMQKEI TALAPSTMKIKIIAPPER KYSVWIGGSILASLSTF QQMWISKQEYDESGPS IVHRKCF	67	7	14	3	53.2	3.527	4	*	[illegible] cytoplasmic actin A3b [Helicoverpa zea] >gb|AAL89658.1|AF286060_1 cytoplasmic actin A3a1 [Helicoverpa zea] >emb|CAA66218.1| Cytoplasmin actin A3a [Helicoverpa armigera] >emb|CAD58315.1| non-muscle actin [Manduca sexta] >gb|ABS57458.1| actin 1 [Heliconius erato] >gb|ADN84932.1| actin [Helicoverpa armigera] >gb|ADO32988.1| cytoplasmic actin A3 [Biston betularia] >gb|AEA29698.1| actin [Trichoplusia ni] >gb|AEV89776.1| actin

62	307	LMI_GLEAN_10124558	[mRNA] locus=scaffold2014:539279:628696:- [translate_table: standard]	174	68680	14.6	MLRSFVRAGICQIQKAS MATAAAIPAPNPNPEVL YTGIFINNEWHKSSSSK TFKTINPATEEVIAEIQE GDKADVDKAVKAAQDA FRFGSPWRTINASERG LLLNRLADLIERDRQYL ASLETLDNGKPYTASYA GDLDLSVKTLRYYAGW ADKIHGKTVPYDGDYFT YTRHEPVGVCGQIIPWN FPLLMMAWKLGPALAT GNTIVLKPAEQTPLTAL YVAQLVKEAGFPPGVV NVIPGDGKTGSFIAEHM DVDKVAFTGSTEVGKLI KQAAGRTNLKRVTLEL GGKSPNIVFSDADLEYA VEQAHFGLFFNMGQCC CAGSRTFIEDSIYDKFV EMSAERAKKRATGNPF DMSVESGPLVDDQQFN KVLGLIKKGQSEGAKML VGGGRIGDKGYFVQPT VFADVKDNMTIAREEIF GPVQQLIRFKKLDELIE RANKTDYGLAAAIFTKDI DKANYLLQGIRAGTVW VNCYNVLNVQAPFGGY KMSGNGRELGEYGLEA YTEVKTVIVKIPQKNN	10	8	7	6	14.6	1.815	8	*	GF22728 [Drosophila ananassae] >gb|EDV33005.1| GF22728 [Drosophila ananassae] XP_001964871.1 aldehyde dehydrogenase, mitochondrial-like [Nasonia vitripennis] XP_001604192.1

63	1331	LMI_GLEAN_10127658	[mRNA] locus=scaffold1949:72975:83056:- [translate table: standard]	25	27166	3.6	MLDQLSGGMGRDINAGEVPTAKDSMKRLEGRNSGSVMCGMADTCREQAILAFTDNINMRRKTTKIIMMMSQLGKYKGDVNNNQLSRKDISRTVKVGFRQVNFGKDLATSEHKGMVIGTEKGILSPGGRHVPPIVSAMAMYTASKHAVKILLEGLRKDLVAKGSNIRVGVSPFNTQRGPGFLLHSYKDWLKSKEN	2	2	1	1	3.6	3.525	2	*	dehydrogenase/reductase SDR family member 11-like [Acyrthosiphon pisum]' XP_001947617.2

64	263	LMI_GLEAN_10124634	[mRNA] locus=scaffold4762:262864:276733:- [translate_table: standard]	200	63551	19.2	MSYNKVISPNQANKEHVLAVSRDFISQPRLTYKTVSGVNGPLVILDEVKFPKFAEIVQLKLADGTIRSGQVLEVSGSKAVVQVFEGTSGIDAKNTLCEFTGDILRTPVSEDMLGRVFNGSGKPIDKGPPILAEDYLDIQGQPINPWSRIYPEEMIQTGISAIDVMNSIARGQKIPIFSAAGLPHNEIAAQICRQAGLVKLPGKSVLDDHEDNFAIVFAAMGVNMETARFFKQDFEENGSMENVCLFLNLANDPTIERIITPRLALTAAEFLAYQCEKHVLVILTDMSSYAEALREVSAAREEVPGRRGFPGYMYTDLATIYERAGRVEGRSGSITQIPILTMPNDDITHPIPDLTGYITEGQIYVDRQLHNRQIYPPVNVLPSLSRLMKSAIGENMTRKDHADVSNQLYACYAIGKDVQAMKAVVGEEALTPDDLLYLEFLTKFEKNFISQGNYENRTVFESLDIGWQLLRIFPKEMLKRIPASILAEFYPRDSRHPQTK	7	7	7	7	19.2	2.803	7	*	Full=V-type proton ATPase subunit B; Short=V-ATPase subunit B; AltName: Full=Vacuolar proton pump subunit B >emb|CAA45706.1| H(+)-transporting ATPase [Manduca sexta]^ P31401.1

Down-regulated in the gregaria (as compared with the solitaria)

65	726	LMI_GLEAN_10109880	[mRNA] locus=scaffold21393:23522:80363:- [translate_table: standard]	99	47921	5.4	MPHTTAHWERRNRHTA ATAAEGTSPAGEAVST PRKRIAHQLKREGRGP TGKGPSANKKLTPREQ LEKKILTPSAGRNFPEE TPVKHLKRLMGVHPDS YLFQPPVQRHAVEDLDI PEEFDSREKWSFCPTIK EIRDQGSCGSCWAFGA VEAMSDRVCIHSKGEK NFHFSAEDLVSCCRSC GFGCNGGFPGAAWAY WKRKGIVSGGAYNSSQ GCQPYEIPPCEHHVNG TRLPCTGEGGNTPRCE KQCEDGYPVSYTEDLH YGETAYSIEGVSKQIQA EIMKNGPVEGAFTVYE DFVHYKSGVYQHVTGT ALGGHAIKIIGWGVLDD TPFWLVANSWNSDWG DGGFFRIKRGNNECGIE SQINAGLPRIN	3	3	2	2	5.4	0.617	3	*	similar to cathepsin b [Tribolium castaneum].XP_974220

66	286	LMI_GLEAN_10133889	[mRNA] locus=scaffold3811:338056:347622:+ [translate_table: standard]	183	33723	14.4	MRLLAVFSLLVVAAVDA RIGDDPTPGGVGVRTC PSSDPEVVTCRRNALQ SALSLLAGGIPSIGARPI DPLTQIPPLILETESPFK LKFKLDDISMIGHSSSVI DDLDIDVQNHTIHVATH TPGALILKGIYTLDEEVI KGIPMGGNGRFKLTML DSTADVTYKGHPVTGR DGETYLKLDSAKTKYTY GKTNYELTGLFGGFGP LEAAGNALINTMTSPIVE REMLGPMEAWMEQVY KGQAQNVFDTIPFSKLF PDSVSTFSDKSFSNSIN	6	6	3	3	14.4	0.464	6	*	takeout-like [Acyrthosiphon pisum] >dbj|BAH71589.1| ACYPI006265 [Acyrthosiphon pisum] , NP_001191952.1" protein takeout-like [Acyrthosiphon pisum]' XP_001950706.1

67	46	LMI_GLEAN_10143558	[mRNA] locus=scaffold12735:230256:325611:+ [translate_table: standard]	908	89593	26.2	ADKNFLLRQKKLLEVF<br>WHVGQPTIDPEQRKISE<br>TFNLEENVNNFKDPELV<br>KKFVNYYNHGYFKQRG<br>EQFSIYNKLDRIQAKAL<br>VDLLYQANDFETFYKTS<br>VWARDHLNEGLFVYAL<br>NVAKLHREDLFDVVLPP<br>FYELYPQLYVSPEVIKE<br>AWEATLEGKTFSKENP<br>YVIRVNYSGQPFARNA<br>DELVSYYTEDVGLNAYL<br>DFMHYRYPFWAKMPEY<br>NQANYTRRGDHFYYGI<br>KAALARYNLERLSNGLP<br>DVEVIDYTKPINGVVPP<br>APLVQYNLERLSNGLP<br>DVEAIDYTKPINGVAPQ<br>GQLVQYVNTLEKRLRE<br>AVDAGYVFDSNFTKYS<br>LRDPLSIEILGRIVEGNA<br>DSINDDYYGSFYRSLLS<br>LAAAPGVSIVFVPACTL<br>RNNNVFSERVFHFQSV<br>TPASSFSNPTYYRVAK<br>RVASIFDQFKDKLGPYS<br>RDQLLLPGVNVESLTVD<br>KLVTFFDDYDFELNNAI<br>PVGSVEEGAKLNVLAR<br>VQRLTHKPFNYHVKVT<br>SDKDIDVFVRFFFGPRY<br>DVYGKELTPNEKRHNM<br>[illegible]	41	39	15	14	26.2	0.106	37	*	hexamerin-like protein 1 [Locusta migratoria]` ACU78068.1

68	318	LMI_GLEAN_10138023	[mRNA] locus=scaffold3867:168504:241806:- [translate_table: standard]	167	120039	3.1	MNPEVTMELIQVLEKTV SLDKTELEQAQNYLEQ AAQTNLGEFLKTLSDIL HHGGNSPVARMAAGL QLKNTLTSKDTALKTQY QQRWLGFPEETRSYIK KNILAALGTENNRPSSA AQCVAYVAVAELPVGQ WPELIGIMVNNVISATST EMMKEATLEAIGYICQD IEHEVLVSQSNHILTAIIH GMRQNEPSNRVRLAAT TALLNSLEFTRANFDKE SERNFIMEVVCEATQS QDTQVRVAALQCLVKI MSLYYQYMEPYMGQAL FPITLEAMRSDIDEVAL QGIEFWSNVSDEEVDL AIEDSEAAEIGRPPQRT SRFYAKGALQFLVPVL MQKLTKQEEYDDEDD WNPSKAAGVCLMLLAT CCEDDIVPFVLPFVKDN IKSTDWRYRDAALMAF GSILGGLEPKTLKPLVE QAMPTLIELMYDSSVVV RDTAAWTFGRVCEIIPE AAINETYLKPLLEALVN GLKAEPRVAANVCWAF TGLAEASYEQAVGKEE QTQPETYCLSHYFEFIV QRLLETTDRADGAQAN [illegible]	4	4	2	2	3.1	0.663	4	*	importin subunit beta-1-like isoform 1 [Nasonia vitripennis]ˆ XP_001599381.2

69	604	LMI_gi_159434	[mRNA] locus=scaffold326:498497:506722: + [translate table: standard]	82	20460	20.1	MKLVVAAVLAMAASAV AAPLSAHGHVPSSTCA DMLPVHGNAVPSTKPS PYTITVSPTSVNGGDTV RVHISGTEEFRGVYLQA GPRASRRVPAARRREQ QDRPVRLPAGTQPALL SNFSSLSQNAFSYISRT PLDTLDIDWKAPDTSDE IVFRATFVKSFSEFWVG VESPKITIG	3	3	3	3	20.1	0.37	3	*	conserved hypothetical protein [Pediculus humanus corporis] >gb|EEB10389.1| conserved hypothetical protein [Pediculus humanus corporis] , XP_002423127.1

70	152	LMI_GLEAN_10133888	[mRNA] locus=scaffold3811:304216:308862:+ [translate_table: standard]	342	36114	26.4	MKLAAVLALIVVAAVDGRIGDDPGSLGVKVCPSSEPDVVSCRRNALQSALSLLAGGIPSIGARPIDPLTQIPPLILETESPFKLKFKLDDIVMIGHARSVLDNLEIDVQNHTIHVVTHTPGALVMKGIYTLDEEIIKGIPMRGNGRFKLTMIDSTADVTYKGHPVAGRGGETYLKLDSAKTKYTYGKTSYELTGLFGGFPPLEAAGNALINMMTSPIVEREMLGPMEDWMEQVYKGQAQYVFDTIPYSKLFPESVSAVPKSSKTVSKESSFFYNLK	14	14	6	6	26.4	0.562	14	*	protein takeout-like [Acyrthosiphon pisum], XP_001950706.1

71	212	LMI_GLEAN_10002265	[mRNA] locus=scaffold329667:159:779:- [translate table: standard]	254	19442	28.5	MEAISHAGTCLGILANDGILLAAERRNTNKLLDEVFFSEKIYKLNDDMVCSVAGITSDANVLTNELRMIAQRYLIQYGESIPCEQLVSWLCDVKQAYTQYGGKRPFGVSILYMGWDKHYGYQLYQSDPSGNYGGWKATCIGNNSAVSADNP	7	7	3	3	28.5	0.66	7	*	Proteasome subunit alpha type-4 [Harpegnathos saltator]", EFN87452.1

72	159	LMI_GLEAN_10054296	[mRNA] locus=scaffold40832:75736:102170:- [translate_table: standard]	333	58868	17.5	MILVVKCPNRKRAAFAGATYDYSDVIQKSLLFYQAQRSGKLSGMDPLVSWRKDSCLDDKGLFGEDLTQGYFDAGDFVKFTFPAASAFTVLSWGAIEFEQGYQQAGALDGVRDAIKWGTDWLLKAHPQPNQLYLQVGDGTADHNYWGRPEDINFARPAYLLNDSYPGTDAAGEAAAALASASIVFQSVDSQYSATLLNAAKELLSFADNYRALCTDSIPEMAEFYTSSGYTDELVWGYVWVYKATGDNSYLQRALELFEEFDLQYISSYFSFDQKTIGIVGLLAEITGEQRFITQLTDHCYNMVHQQQRTPKGLLYISDWGNLRLAANVALTCLKFPHIWFGVALMKNSGCQRPDDSDHGSNESDGRKRDNGRVAGHQHGGEPGAGQGADRLGDTGRSFVVGYGTNPPVRPHHRAASCPDAPASCDWTQYSTSEPNPHVLYGALVGGPDSSDAYSDDRTDYQHNEVALDFNAGFQGVLAALVQLGL	12	11	7	6	17.5	0.567	11	*	beta-1,4-endoglucanase 1 [Panesthia cribrata]` AAF80584.1

73	681	LMI_GLEAN_10066930	[mRNA] locus=scaffold30572:44076:141777:+ [translate_table: standard]	72	75609	3.8	MDSGDLGELNAAYESY QYGGLEYDPHTAEAPA EYDYEQYSQMDKTVVR DLLYFAECVNERMLFEL QSLYETHFPKTMEDYY GKNAWPSEEMVVRVV GQDPVFLTLYKELYYRH IYEITDKPGPSLEMRVR SFYNYCDLFTYILGAEH PVPLELPDQWLWEIIDE FVYQFQSFARYQASKAI GPEAIAFLQSDKSVWN VINVLNVLHSMIEKSNIK RQLEVYASGGDPNSVA GEFGRHSLYKMLGYFS LVGLLRLHSLLGDYYQA IKVLKNIELHKKSQYAHV PACQISTSYYVGFSYM MMRRYSDAIRTFSSSLL YIQRTKQMYQSTSYQH DQIKKQTDQMYHLLAIC LVLHPQYIDESLQHALR DKNFLETMNKMQRGEL EEFQQCFLYACPKFVP ATPPLSDSDTDLVEETI KHQKDVFMDEVRQQK NLPTIRSYLKLYTTLPLS KLATFMSQGQRDSNE WDLNKEMQDLIINLLCF KHKMKNVVWTKGTSGL DGKFQSGSELDFYIDRD MIHIADTKVSHRYGDFFI [illegible]	4	4	2	2	3.8	0.565	4	*	similar to eukaryotic translation initiation factor 3 [Tribolium castaneum] >gb|EFA00209.1| hypothetical protei[illegible] TcasGA2_TC003034 [Tribolium castaneum]^ XP_971137.1

74	532	LMI_GLEAN_10175495	[mRNA] locus=scaffold1962:1203384:1276222:- [translate_table: standard]	93	45792	15.4	MADGAGNWCLIESDPGVFTELIKEFGVKGVQVEELWSLDAEQFENLKPIHGLIFLFKWVQDDEPSGSVVQDSRLDKIFFARQVINNACATQAILSILFNCKHEDISLGGTLTEFRDFCQSFDANMKGLALTNSQTIRTVHNSFARQTLFEYESKLASKNDEVYHFVGYVPIEGRLYELDGLKEGPIDLGPVASDADWLDVVRPIIEKRIKKYSEGEIHFNLMAIVSDRRMIYERKIQDIQKQVEENGMETDAQQSEIARLKVLIEEEEAKIKRYQAENIRRKHNYLPLIVEILKILAKEGQLLPLYEKAKERALEKETKREEVKT	3	3	3	3	15.4	0.459	3	*	ubiquitin carboxyl-terminal hydrolase isozyme L5, putative [Pediculus humanus corporis]ˆ XP_002431967.1

75	443	LMI_GLEAN_10082914	[mRNA] locus=scaffold26261:105188:217137:- [translate_table: standard]	119	118835	1.6	MDFLKSGLKSVLGGPN<br>EGSQPSGAETVERLVD<br>RVQSSTLLDDRRDACR<br>ALKALSRKYRVEVGAQ<br>GMDALRQVLEMDRADA<br>EIVGYALDTLCNITSGE<br>NFEEEEHINLNSALVATI<br>GEQFTEIFIKQPDNVGL<br>VLGFLEEYDFHVRWPA<br>VKLLTNLLANKPKEIQEII<br>LVSPMGVSRLMDLLGD<br>SREVIRNDALLLLIQLTK<br>GNANIQKIVAFENAFDR<br>LFDVISKEGYSDGGIVV<br>EDCLLLMLNLLRNNTSN<br>QNFFKEGSYIQRLAPMF<br>NLPAEGDDAIWSPQKV<br>SNVHCMLQVLRTLVTP<br>GNPAQVTTSSQKTMKS<br>CGLLENLCNILMASGVP<br>ADILTETINTVGEVIRGN<br>MSNQEYFANVLAPSAR<br>PAIVVLLMSMVNEKQPF<br>ILRCSVLYCFQCFLFKN<br>EVGQAQLVQTLLPSTS<br>EMSTLTTGQLLCGGMF<br>SADPLSNWFSAVALAH<br>ALVENPAQKEQLLRVM<br>LATSIGNPPVSLLNQCT<br>SLLQQCGKVQSKLGILM<br>LLSTWLAHCPLAVKQFV<br>SIPTSIPYLTAQVGSNE<br>[illegible]	2	2	1	1	1.6	0.663	2	*	similar to vesicle docking protein P115 [Tribolium castaneum] >gb|EFA08682.1| hypothetical protein TcasGA2_T[illegible]

76	763	LMI_GLEAN_10192264	[mRNA] locus=scaffold4192:2926256:3015937:+ [translate_table: standard]	62	92108	5.6	MGGKYSSLDPMEVEVPNLDELLNRDPYLKPYEREFRKRYAVFKDCLEKIEAYEGGLQKFTQGYKSYGIHVNPDNSVTCKEWAPGAVELYLYGDFNNWNRQSHPYARCEYGKWELQIPPKADGSCPIDHMSEVKIIVKTHSGETVDRLSPWAPYVLQPPSSEGYTFKQRLWNPPSDQRYHFKHPEPKKPESMRIYECHVGIATKEPKVGSYKEFITNVIPRIVKNGYNTIQLMAVMEHTYYASFGYLVTNFYAASSRFGTPEELKELVDAAHANGLYVIIDLVHSHASKNVLDGLNQFDGTNSCYFHDGPRGDHCHWGSRLFNYTEYEVLRFLLSNLRWWKEEYKFDGFRFDGVTSMLYHSHGANKGFSGSYDDYFNLDVDTESVMYIMMANHILHDLYPEITTIAEDVSGMPASCRPVSEGGLGFDYRLAMAIPDMWIKILKEIKDEDWDMGKIVYTLTNRRWMEKTVAYAESHDQALVGDKTLAFWLMDKEMYTHMSVLSEPNNVIDRGLALHKMIRLITHGLGGEAY[illegible]	3	3	3	3	5.6	0.654	3	*	1,4-alpha-glucan-branching enzyme-like [Megachile rotundata]` XP_003707245.1

77	683	LMI_GLEAN_10168539	[mRNA] locus=scaffold1455:1120177:1184172:- [translate_table: standard]	71	34245	4	MGRRPKQQLSQGQGAEDYVDRGPVVELIYWRDPKKSGIVFGSVLGILLSLTYFSLISVIAYLSLAILTGTISFRVYKTVLQAVQKTTSDGHPFKDILELDLTLPQEKVREMTDLGVAHLNAAACELRRLFLVEDLVDSIKFGVLLWCLTYVGAWFNGMTLIIIAFVALFTLPKVYETNKVQIDQNIDLVRSKIAEISSNLEFASRLSAFGKGVHCSGVGISVRVTLEVEGEATHTVAYSPGEGVSQLAVEAVGCSVTSDGLKSSVMIIALQVM	2	2	1	1	4	0.653	2	*	Reticulon-1 [Camponotus floridanus]", EFN73447.1 "; similar to reticulon/nogo [Tribolium castaneum] ", XP_967166.2

78	402	LMI_GLEAN_10196136	[mRNA] locus=scaffold1972:2488450:2540115:+ [translate table: standard]	134	22230	11.4	MSQGKPKKEKATVVERHQRPEMRQSAVSAAEIVPRNFRLLEELEQGQKGVGDGTISWGLENDDDMTLTHWTGMIIGPPRTPYENRMYSLKIECGARYPDECPSARFISRINMTCVNSTNGVVDSRAVPVLARWQRDYTIKTVLQELRRIMTLKENMKLSQPPEGSSY	6	6	2	2	11.4	0.599	6	*	enzyme E2 variant 2-like [Bombus terrestris] >ref|XP_003488993.1| PREDICTED: ubiquitin-conjugating enzyme E2 variant 2-like [Bombus impatiens] >ref|XP_003691588.1| PREDICTED: ubiquitin-conjugating enzyme E2 variant 2-like [Apis

79	779	LMI_GLEAN_10099118	[mRNA] locus=scaffold23377:24384:115275:- [translate_table: standard]	61	285999	1.6	MADAAARQLQYEYKAN SNLVLQADVRLIERRSR DEATGEVMSLVGKLDG TRMGDRHQRTKPVKAA ERKAKRQKRDEAQYDF TRMKGATLLSEGVDEM VGILYRPKTQETRQTYE VLLSFIQEALGDQPRDIL CGAADEVLAVLKNDRL KEREKKKETESLLGNLT DERFALLVNLGKKITDF GTEEKTQTSEENIDETY GINVQFEESEEEDDED MYGEIREEEDDEEGEE AKLDRAIHAENLGGAEE TRKEKTLHPLDIDAYWL QRRLSKFYDDAMVSQA RAAEVLAVLRDAGDDR DCENQLVLLLGYSCFDF IKLLKRHRQMILYCTLLA SSQSESERQKIREKMS EDPSLVKILKALDTGGG DDEEIVGGGGGGRGSS GRSRRDQGGEGSGDV GAASTANVPGQRVIVDL EDLVFAQGSHFMANKR CQLPDGSFRKQRKGYE EVHVPALKPKPYGADE TNVPIEKLPKYVQPAFE DCKTLNRIQSRLYKVAL ESDENLLLCAPTGAGKT NVALLCMMREIGKHINP [illegible]	2	2	2	2	1.6	0.513	2	*	similar to pre-mRNA-splicing helicase BRR2 [Tribolium castaneum]` XP_970554.1`` putative U5 small nuclear ri

80	124	LMI_GLEAN_10120545	[mRNA] locus=scaffold10840:91255:98543:- [translate table: standard]	415	22274	28.1	MMQRAALLVFLLASSALAKPTPARQWIRPNGRIIGGTTASIANYPWQLSFQYSGSHICGASIISSDWVLTAVSGSLLGTNAQTVSLPSSGYDPAGGLAVTVTGWGVTSTNGNLPTNLMKVDTSIVARSTCQSIFSGINTVTARMVCAGAAGKSVCNGDSGGPLVSGTTQVGIVSWGNSRCESSPGVFSNVGNLRSWIQQATGI	17	17	5	5	28.1	0.557	17	*	serine protease-like protein [Schistocerca gregaria]`, CAA70820.1`,`trypsin-1-like [Apis florea]`, XP_003689751.1

81	125	LMI_gi_311063281-D1	[mRNA] locus=scaffold1923:1520655:1554566:- [translate table: standard]	412	29300	27.6	SYIKACKMNDPNLNEC SLKNGREAIPSILKGDP KYRVPSFDPLVLTEVSV ADRGLNITLRNVRLLGIP DIDFRSSVIDLTGMKVI WGFFLPRLEVLSDYTV DGKILVLPLKGNGPANI TLLDVPIILTFDYELMKK NDGKEYLNPTKVTPFFN STQVYIQLDNLFNGDKL LGDNTNKFLNENWREV VADVGPVLAQGLAEAV RQILAGIALQVPFNLLFT Q	12	12	5	5	27.6	0.49	12	*	protein takeout-like [Acyrthosiphon pisum], XP_001947537.1
82	1008	LMI_GLEAN_100 51974	locus=scaffold299 67:8258:13256:+ [translate table:	42	11260	8.9	MEIGGKAVDALRQRYG TEYEYGTIYDIIYPASGG SIDWVKKALGTRFVYE WELRDTGRDXXADQIV PTALETIDSMVVILQEAV ARTAAH	2	2	1	1	8.9	0.468	2	*	A-like [Tribolium castaneum] >gb|EFA05749.1| hypothetical protein TcasGA2_TC015 782 [Tribolium

83	262	LMI_GLEAN_10117209	[mRNA] locus=scaffold16880:156430:158190:+ [translate table: standard]	200	30507	26.5	MAAKYKLTYFPIMALGE PIRFLLSYGKIEFEDERC EREKWPSVKESMPFG QMPVLEIDGKKTWQSL AICRYLGKQMGLAGAN DWEDLQIDMAIETVTDL RLKIANYWYETDEAQK EKKKEPLFNETLPFLLP RLDNLVKENGGYLANG KLSWADLYFIAVLDYLN TMVGFDLTKDYPNLAEL KNKVLEIPAIKEWVDKR PKTDL	10	5	5	3	26.5	0.593	5	*	glutathione S-transferase sigma 1 [Locusta migratoria]` AEB91973.1
84	436	LMI_GLEAN_10002280	locus=C189020578:351:862:+ [translate table:	121	14295	20.8	MPSYVWWCAFYWHSR TLCSISCASCYRFVTHL MKRLRHSQVRGISIKLQ EEERERRDNYVPEVSA LEQDIIEVDPETKDMLK MLDFNNITGLQLTQPAT QGFGRRP	2	2	1	1	20.8	0.558	2	*	S17, putative [Pediculus humanus corporis] >gb|EEB10115.1| 40S ribosomal protein S17, putative [Pediculus humanus corporis] `

85	723	LMI_GLEAN_10109545	[mRNA] locus=scaffold1141:13996:101260:+ [translate_table: standard]	66	94472	4.9	MLYRRAGVAEWSGAP SCGSRGLPRRKLNRKE KEDNVVGKANQRLLFIG RTLKRCNHRTTKETAYT TFARPFLEYCWLVWDP YQVGLTKDNRKRPKKG TIQRYGNDKKSGMGIIT TTALIAAALEELTSLKDI PNSLAVFCMATYGEGD PTDNAMEFYEWLQNGD PDLSGLNYAVFGLGNK TYEHYNEVAKYIDKRLE DLGATRVYEMGLGDDD ANIEDDFITWKDQFWPA VCEFFGIESTGEDVSVR QYRLTEHDSISSDKIYT GEVARLHSLANQRPPY DAKNPFLAPVKVNKELH KAGDRSCMHIEFDIEGS KMRYDSGDHVAVYPM NDEALVNRIGELLNIDLD TVITLTNTDEESTKKHPF PCPCSYRTALRHYLDIT SNPRTHILKELIEYTKDP QEQEKLKLMSSTSPEG KSLYNQWIIKDNRNIVHI LEDLPSCKPALDHLCEL LPRLQARYYSISSSPKV YPNSVHVTAVLVDYTTP TGRHNKGVATSWLKKK QPKEDFTPTTPIYIRRS QFRLPTRTQTPIIMIGPG [illegible]	2	2	2	2	4.9	0.479	2	*	NADPH--cytochrome P450, putative [Pediculus humanus corporis] >gb|EEB11242.1| NADPH--cytochrome P450, corporis]ˆ XP_002423980.1

86	500	LMI_GLEAN_10042954	[mRNA] locus=scaffold51358:58549:180221:+ [translate_table: standard]	102	54645	10.8	MRVTVTTLSDEIFVLDVSEDLELENFKALCEMESGFPSREILIVYSGRPLTDDRKSMKEHGIADGDVLILQRMQGSSTSLSAAAAGLHLLDFSSIQVPPEALAATDTASDDTSMEDVAGPGPTYAAEEDPRVIRDMFLANPDQLALLKQNNPRLADALLSGNLDRFAAVLREQVEIRAERERQRIRMMNADPFDLEAQRLIAEEIRRKNIEANLEAAIEYNPEIFGTVTMLYINCRVNGHPVKAFIDSGAQTTIMSAACAERCNIMRLVDTRWAGVAKGVGVQRIIGRIHMVQVQIETDHLTTSFSVLQEQPMDMLLGLDMLRGHQCCIDLQKNVLRIGTTGTETPFLPESELPECARLSGPGESASAEAVGRSMREAEDRELARALQDSEASAAAHRSSSMDSQDGGGRAASLPRDPSLTILPTDKFGENEVRDLVSLGFTREQVIAELRRFDGDKNRATASV	3	3	3	3	10.8	0.638	3	*	similar to DNA-damage inducible protein [Tribolium castaneum]^ XP_969775.2

87	647	LMI_GLEAN_10170704	[mRNA] locus=scaffold351:1721876:1758756:+ [translate_table: standard]	76	153512	2.6	MHLYNLTLQRATGITHA VHGNFSGSKTQEILVSR GKSLELMRPDPNTGKV HTLLTVEVFGIIRSLMAF RLTGGTKDYIVVGSDS GRIVILEYSPSKNIFEKV HQETFGKSGCRRIVPG QYLAIDPKGRAVMIGAI EKQKLVYILNRDAEARL TISSPLEAHKSNTLVYH MVGVDVGFENPMFACL EIDYEEADTDPTGEAAH KTQQTLTFYELDLGLNH VVRKYSEPLEEHANFLV SVPGGNDGPSGVLICS ENYLTYKNLGDQHDIRC PIPRRRNDLDDPERGMI FVCSATHKTKSMFFFLA QTEQGDIFKITLETDED MVTEIKLKYFDTVPVAT AMCVMKTGFLFVASEF GNHYLYQIAHLGDDDD EPEFSSAMPLEEGDTF FFAPRHLRNLVLVDELD SLSPIMACQVADLANED TPQLYMLCGRGPRSTV RVLRHGLEVSEMAVSE LPGNPNAVWTVKRRVD EEYDAYIIVAFVNATLVL SIGETVEEVTDSGFLGT TPTLSCSALGEDALVQV YPNGIRHIRADKRVNEW [illegible]	2	2	2	2	2.6	0.588	2	*	Splicing factor 3B subunit, putative [Pediculus humanus corporis] >gb|EEB16979.1| Splicing factor 3B subunit, pu[illegible]

88	328	LMI_GLEAN_10081266	[mRNA] locus=scaffold9813:59159:81270:+ [translate_table: standard]	163	37236	23.5	MPGFSSVGTFKIFVGNLADKTTVDDIKPLFEKYGKVVECDVVKNYGFVHMEHEEAGRDAIQNLNGYLVHGQAIKVEAATSRKGPQTPTTKIFVGNLTDNTKAPQVRALFAKYGTVLECDIVRNYGFVHIESNDNVNLAIKELNGYIVDGQPMKVQVSTSRVRQRPGMGDPEQCYRCGRGGHWSKECPKAGVFVFISRLGPDRNGFRGPIFGREPYPPPPPPPFLRDRMMGRFGMRDPLYDGFYDRNRFEDSPRDMFERRFPPLPPRDLGPSLRGREFLPPPPLPP	9	9	6	6	23.5	0.666	8	*	Arginine/serine-rich-splicing factor RSP31, putative [Pediculus humanus corporis] >gb|EEB11604.1| Arginine/serine-rich-splicing factor RSP31, putative [Pediculus humanus corporis]ˆ XP_002424342.1

89	419	LMI_GLEAN_10127412	[mRNA] locus=scaffold3841:6547:36767:- [translate_table: standard]	126	37758	18.8	MAAKEGKKANVHSVCIL GGVLPDVERVLPDAIKG TGCSQLQVVAHIGTKD VCRFGSEEILRLISTSGT HQDSHESWFSKLLVRR IEPTKESHSRLLSDKEVI YELQTHNMRPDSVDNY LKNYKENVDLIQSRNLS CELVGSWTVTVGDLDQ ALHLWRYTGGFTSIDKA RKELLQDPAFVRLQQE RGKMLRSRHLQYVLAF SYWPPIQLRKGPNLYEI RSYRLKPGTMIEWGNN WARAINYRRNNDEPFA GFFSQIGRLYNVHHIWC MYTVVTICIHSMIFVF	6	6	5	5	18.8	0.665	6	*	nipsnap [Aedes aegypti] >gb|EAT48786.1| nipsnap [Aedes aegypti]` XP_001658894.1

90	484	LMI_GLEAN_10131870	[mRNA] locus=scaffold1839:709122:800003:- [translate_table: standard]	107	62884	6.1	MAADTGMDTCPSPEIPDSRKRPLDGDVENGVTKKSHYAGGGDGTYHFKLLVPSTAAGAIIGKGGETIAQLQKDTGARVKMSKANDFYPGTTERICLITGTVDAIMLVLIFIMEKIREKPDLNAKQAIDFDSKTAAERDKQVKILVPNSTAGMIIGKGGNYIKQIKEESGSYVQISQKAKDQTLQERCITVIGDMESNKTACRMILVKIVEDPQSGSCLNVSYADVSGPVANYNPTGSPYANSGQHTPGTFSSTASLNTTVGGMLLNGLNLSLNLGSPTAGTSLTTQLLEHIQVVLRGSGFSEQATGEITAAMSTLARYGILGMGLGLAGAGTAAGGSGPAGYLGADAPASAANGGGAFGPIGTVSAIGSPSPTPRSTDRFGCPTDGYDPFRRQASSPGVASPASLNSNSFGLGTGGAVSPLLKGAGVDLKADHKKVDVEISESIVGAILGPGGRALVEIQHLSGASIQISKKGTFAPGTRNRIVTISGSPNAITNARYLIEQRVSEEEAKRARHGSLGPILQ	5	5	3	3	6.1	0.602	5	*	RNA-binding protein Nova-2-like [Bombus terrestris] >ref|XP_003494948.1| PREDICTED: RNA-binding protein Nova-2-like [Bombus impatiens]ˆ XP_003395567.1

Note: The resulting MS/MS spectra were searched against the locust protein database generated from the newly assembled genome with MASCOT software, and a score will be generated by the software. The higher the score is, the more accurate the identification is.
